# Regional and educational disparities in inaccurately coded deaths in Sweden, 1997–2023: a systematic analysis

**DOI:** 10.1186/s12963-026-00471-8

**Published:** 2026-03-18

**Authors:** Authia Gray, Peter Allebeck, Mohsen Naghavi, Brita Zilg, Vincent Mougin, Emmanuela Gakidou, Matthew Cunningham, Emilie E. Agardh

**Affiliations:** 1 Institute for Health Metrics and Evaluation, University of Washington, 3980 15th Ave. NE, Washington 98195 Seattle, United States; 2 Department of Global Public Health, Karolinska Institutet, Solnavägen 1, 113 65 Stockholm, Sweden

**Keywords:** Cause of death, Global Burden of Disease, Education, Socioeconomic disparity

## Abstract

**Background:**

Accurate underlying cause of death (CoD) data is critical for informing public health policy, but inaccurate CoD assignment, here called garbage code (GC) deaths, compromise CoD research and monitoring. Since 1997, GCs have consistently made up over 20% of all underlying CoDs in Sweden, but the distribution of GC deaths by sociodemographic status of the deceased remains poorly understood.

**Methods:**

We used the Swedish Cause of Death Register containing 2.50 million death records from 1997 to 2023. We mapped each record to the Global Burden of Disease (GBD) project cause list and categorized GC deaths by disease groups. We calculated the fraction of deaths that were GCs by individual age, sex, region of death, and highest educational attainment. We performed redistribution of GCs onto well-defined CoDs and assessed the odds of GC assignment with a binomial logistic regression.

**Results:**

Since 1997, Sweden has coded at least 23% of deaths to GCs each year with 25.5% coded to GCs in 2023. The lowest educated consistently received more GC deaths, with 45.8% more GC deaths relative to non-GC deaths between ages 20 and 39 compared to the highest educated, and there were more GC deaths in (1) infections, (2) blood and endocrine diseases, (3) injuries, (4) cancers, and (5) maternal, neonatal, and congenital (MNC) diseases in 2023. GC deaths among the highest educated have continued to increase in infections, injuries, cardiovascular, digestive, and MNC diseases. After redistribution, well-defined death counts among the lowest educated increased by over 20% in 13 of the leading 20 CoDs in Sweden. Our model suggested low education increased the likelihood of having a GC by 12.8% (11.4%-14.2%) compared to the highest educated. This was second to point estimates of standardized age at death (25.2% [24.8%-25.6%]) and exceeded sex (12.1% [11.4%-12.8%] increase for males) and region (at most 7.3% [6.6%-8.1%] decrease for death outside of Stockholm).

**Conclusions:**

We found consistent trends of high GC level in Sweden with doctors assigning more GCs to the lowest educated. Our results reveal stark sociodemographic disparities in CoD coding in Sweden and it is probable that similar disparities would be found elsewhere. This underscores the need for improving procedures and national guidelines in CoD assignment to correctly represent all social groups in research.

**Supplementary Information:**

The online version contains supplementary material available at 10.1186/s12963-026-00471-8.

## Background

Standardized cause of death (CoD) data is critical for informing public health policy. To promote standardization, the World Health Organization (WHO) issued rules known as the International Classification of Disease (ICD) system for classifying underlying causes of death (UCoDs) [1]. Unfortunately, errors and improper coding and misassignment of CoDs are common. Examples of inaccuracies are non-specificity (e.g. R99, other ill-defined and unspecified causes of death), impossibility (e.g. M54.5, low back pain), or inaccuracy through the misuse of an intermediate or immediate CoD (e.g. I46, cardiac arrest) [2, 3]. Without correction, such problematic CoDs may be excluded or misclassified in mortality research because the well-defined UCoD cannot be determined with confidence. This uncertainty can have significant implications for health planning and priority setting. As a result, misassigned and inaccurate deaths are called “garbage codes” (GCs) in the Global Burden of Disease (GBD) project and the GBD has developed methods to redistribute GCs onto well-defined CoDs to improve comparability [4].

Ideally, GCs would be eradicated, making it important to understand the drivers of GC assignments. Known drivers include insufficient physician training on CoD certification, deaths complicated by multiple comorbidities making UCoD assignment challenging, low levels of autopsies reducing the information available to health authorities, and/or inadequate healthcare access and quality [3, 5, 6]. Variation in GC levels across European countries has been documented, indicating that characteristics of the healthcare system may influence the share of GCs [8]. In addition to these drivers, there is evidence that sociodemographic inequalities are important drivers of GC levels. In a study conducted across 16 European countries, higher proportions of GC deaths were assigned to people with lower education [7]. Understanding the influence of sociodemographic inequalities on GC level is necessary for improving national data and for examining the effect of correcting GC deaths on national mortality trends. Still, there has yet to be a comprehensive assessment of GC level by sociodemographic factors of the deceased.

In Sweden, the healthcare system is decentralized into 21 regions that manage local hospitals and clinics [9]. Death certificates are primarily electronic and for every death in Sweden, a cause-of-death certificate with a UCoD and CoD chain must be completed by a physician [10]. Death certificates are sent to the National Board of Health and Welfare where the ICD codes are assigned using the software Institutional Repository for Information Sharing (IRIS) [10]. Even so, Sweden had a consistent trend of over 20% of age-standardized deaths coded to GCs from 1990 to 2017 while the rest of Western Europe saw a steady decline during a similar period [8].

Given the ubiquitous use of CoD data for public health monitoring and research, it is important that all groups in society are proportionally represented in national health data sources. Therefore, we present a comprehensive assessment of GC level and the effect of GC redistribution on mortality trends by highest attained education, sex, age, and region in Sweden from 1997 to 2023. Through this analysis, we aim to better understand the educational and regional drivers of GC coding as well as the effect of GC death correction.

## Methods

### Data sources

In total, we obtained cause of death (CoD) data on 2.50 million deaths from the Swedish National Cause-of-Death register. These records represented all available ICD-10 certified deaths in Sweden from 1997 to 2023. We retrieved information on subjects’ highest educational attainment by individual linkage to the “The Longitudinal Integrated Database for Health Insurance and Labour Market Studies” (LISA) which provided information on educational attainment by person and year. From the LISA database we also obtained information on the subjects’ region of residence at time of death.

### Data processing

We mapped all ICD-coded deaths to the comprehensive and mutually exclusive list of underlying CoDs and GCs used in the GBD 2023 project (Appendix Table S1, S2). All GC deaths were further categorized by disease groups: blood and endocrine diseases (Blood/Endo), cardiovascular diseases (CVD), cancers (Cancer), digestive diseases (Digestive), genitourinary diseases (Genitourinary), infectious diseases (Infectious), injuries (Inj), maternal, neonatal, and congenital diseases (MNC), mental and neurological conditions (Mental/Neuro), respiratory diseases (Respiratory), all special signs and symptoms (referring to select GCs from ICD-10 chapter R), and other (referring to GCs that cannot otherwise be categorized). Appendix Tables S3 and S4 describe the GCs and the ICD codes associated with them.

To assign each person’s highest educational attainment, we used the highest value assigned in LISA. For persons aged 25 and younger, we assigned the highest educational attainment as the parent’s highest level of education, unless the individual’s education was higher. For adopted persons, we used the highest educational attainment of adoptive parents. We categorized educational attainment into three categories: high (having completed post-secondary or tertiary education with more than 12 years of study), intermediate (completed upper secondary education with 9–12 years of study), and low (completing primary or lower secondary education with overall 9 years of study or less). For more information about educational assignment, see Appendix Sect. 4.1. Data summary statistics and handling of persons with unknown education are described in Appendix Sect. 4. By region, we assessed all 21 regions both in detail and in aggregated categories (Stockholm, Skåne, Västra Götaland, and other) to reduce stochasticity from small populations by grouping the smallest regions together.

### Redistribution

We performed redistribution to correct the GCs in the data and assess mortality trends by education and region before and after redistribution. This redistribution method follows the data processing procedure performed in the GBD, where all deaths recorded as GCs are corrected through redistribution onto specific well-defined underlying causes of death (UCoDs) at the population level by age, sex, year, and location. We summarize here the methodology that has been described previously in detail [11]. There are four primary types of redistribution methods: (i) multiple cause of death (MCoD), (ii) negative correlation, (iii) impairment, and (iv) proportional.

1. The MCoD method was used to redistribute GCs where specific intermediate and immediate CoDs (e.g. I50.1 left ventricular failure) were erroneously assigned to the UCoD position. We used MCoD data to find instances where these GCs were appropriately coded to the intermediate and immediate CoD position. We used these correctly coded death records to determine potential well-defined UCoDs that the GC deaths could have been coded to. The associated well-defined CoDs became the targets for GC redistribution. Final proportions for redistribution were generated through a binomial logistic regression.
2. Negative correlation was used to redistribute specific GCs that correlate inversely with well-defined UCoDs, e.g. when unspecified cancer is high, specified cancer is low. GCs that relate to specific diseases that are obscurred by unspecificity (e.g. chronic kidney disease due to unspecified type diabetes or abdomen and pelvis cancer) are redistributed using negative correlation. This method leverages a negative correlation model to assess the well-defined CoDs (e.g. breast cancer) that significantly decrease with increases in select GCs (e.g. unspecified site cancer). The result from the model is the amount of the GC that gets redistributed onto each well-defined CoD.
3. For GCs that are impairments (e.g. D64, other anemias) with many possible well-defined CoDs, we utilized cause-specific prevalence calculated for each impairment in the GBD. The percentage of years lived with disability (YLDs) due to each cause was then the proportion that the GC was redistributed onto each well-defined cause.
4. For non-specific GCs that have many possible well-defined CoDs (e.g. R99, other ill-defined and unspecified causes of mortality), the deaths were fractionally redistributed onto the well-defined CoDs based on the relative proportion of the CoDs within the dataset.

Please see Appendix Sect. 2 and Table S13 for more detailed information about redistribution.

### Modelling

To isolate the effect of education on the proportion of GC death certification, we ran a fixed effects binomial logistic regression of the form:

$$\begin{aligned}\mathrm{log}\left(GCfraction\right)&={\beta}_{0}+{\beta}_{1} male+{\beta}_{2}age+{\beta}_{3}Regio{n}_{Sk{\overset\circ{a}}ne}\\ & \quad+{\beta}_{4}Regio{n}_{V\ddot{a}straG\ddot{o}taland}+{\beta}_{4}Regio{n}_{other}\\ & \quad+{\beta}_{5}educatio{n}_{intermediate}+{\beta}_{6}educatio{n}_{low}\end{aligned}$$

Where sex, region, and education are categorical variables and age at death is continuous at 5-, 10-, or 15-year intervals, the latter serving as the age covariate standardized by 1 standard deviation. We ran a separate model for each of the three age categorizations for comparability. The reference for the categorical variables in the model is deceased females in Stockholm County with highest possible educational attainment. To simplify our analysis and reduce the number of covariates to avoid overfitting, we kept the three most populous regions distinct (Stockholm, Skåne, and Västra Götaland) and grouped the remaining regions together. Uncertainty we calculated as 1.96 times above and below the standard error.

## Results

### Level of garbage codes

In Sweden, the mean level of garbage coding remained relatively constant with 29.0% (95% CI: [28.7% to 29.3%]) of deaths assigned to GCs in 1997 and 25.5% (25.2% to 25.8%) in 2023 with a minimum of 22.8% (22.5% to 23.1%) in 2020 (Table 1). From 1997 to 2023, GC deaths have dropped from 27 000 (26 700–27 400) deaths to 24 100 (23 800–24 400) deaths. By age, GCs made up the largest fraction of deaths in those aged 90 years and older with 48.0% (47.2% to 48.8%) in 1997 and 32.5% (31.9% to 33.1%) in 2023. The second-most affected in recent years were those under the age of 40 with 19.1% (17.5% to 20.8%) of deaths assigned to GCs in 1997 and 31.2% (29.1% to 33.2%) in 2023. GCs were more common in females than in males with females having 8.1% more GCs in 1997 and 1.2% more GCs in 2023.

**Table 1:** Percentage and counts of deaths that are Garbage Coded by Variable, 1997 and 2023

Variable	Category	1997		2023	
		Percentage	Counts	Percentage	Counts
Overall	All	29.0 (28.7–29.3)	27 000 (26 700 − 27 400)	25.5 (25.2–25.8)	24 100 (23 800 − 24 400)
Education	High	19.6 (18.2–21.1)	561 (515–607)	24.0 (23.2–24.8)	2 600 (2 500–2 700)
	Intermediate	20.5 (19.9–21.1)	3 890 (3 770–4 010)	24.5 (24.1–24.8)	11 300 (11 100 − 11 500)
	Low	24.7 (24.3–25.1)	10 500 (10 300 − 10 700)	26.9 (26.5–27.4)	9 680 (9 480–9 870)
Region	Skåne	28.6 (27.8–29.4)	3 430 (3 310–3 540)	27.5 (26.7–28.3)	3 480 (3 370- 3 600)
	Stockholm	29.5 (28.8–30.2)	4 630 (4 500–4 770)	26.7 (26.0–27.4)	4 290 (4 160–4 420)
	Västra Götaland	29.5 (28.5–30.5)	2 390 (2 290–2 480)	26.0 (25.4–26.7)	4 090 (3 970–4 220)
	Other	28.8 (28.4–29.2)	14 300 (14 000–14 500)	24.5 (24.1–24.9)	12 200 (12 000–12 500)
Age	Under 40	19.1 (17.5–20.8)	415 (375–455)	31.2 (29.1–33.2)	585 (538–632)
	40–69	16.0 (15.5–16.6)	2 710 (2 610–2 810)	21.7 (21.0–22.4)	2 910 (2 800–3 020)
	70–79	21.3 (20.8–21.9)	5 230 (5 090 − 5 370)	20.9 (20.4–21.4)	4 700 (4 560–4 830)
	80–89	33.5 (33.0–34.0)	11 900 (11 700 − 12 100)	25.0 (24.6–25.5)	8 370 (8 190–8 550)
	90+	48.0 (47.2–48.8)	6 770 (6 610–6 930)	32.5 (31.9–33.1)	7 550 (7 380–7 720)
Sex	Male	25.0 (24.6–25.4)	11 700 (11 400 − 11 900)	24.9 (24.5–25.3)	11 900 (11 600 − 12 100)
	Female	33.0 (32.6–33.4)	15 400 (15 100 − 15 600)	26.1 (25.7–26.5)	12 300 (12 000–12 500)

### Educational disparities

**Fig. 1:**
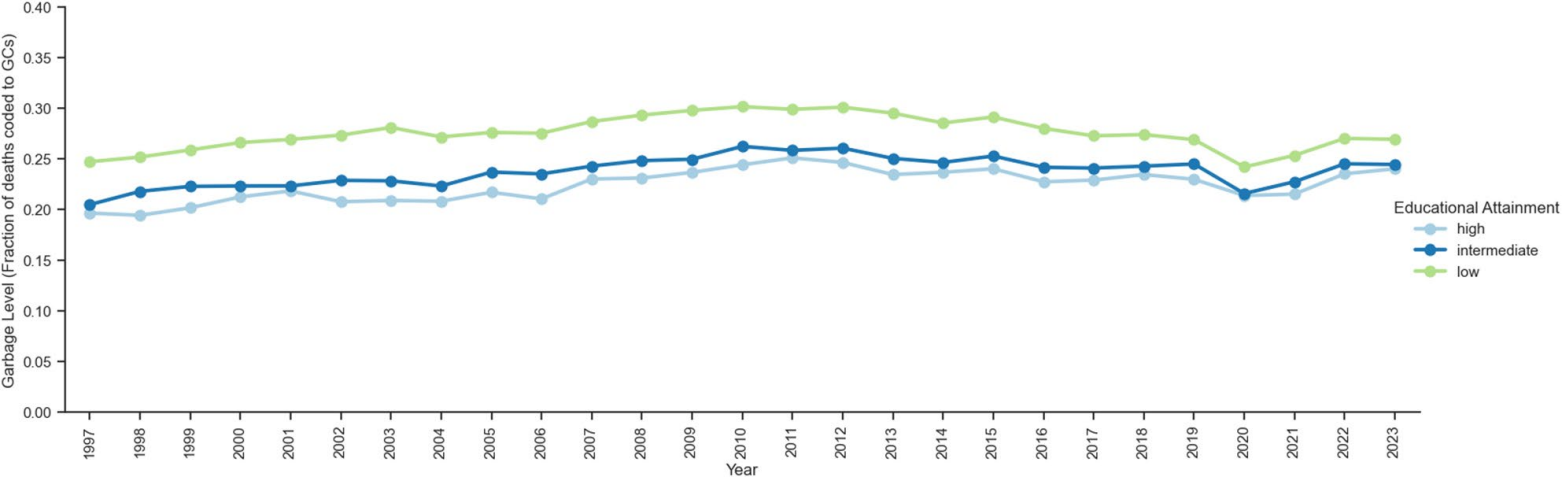
Level of GCs in Sweden by Educational Attainment, 1997 to 2023

**Fig. 2:**
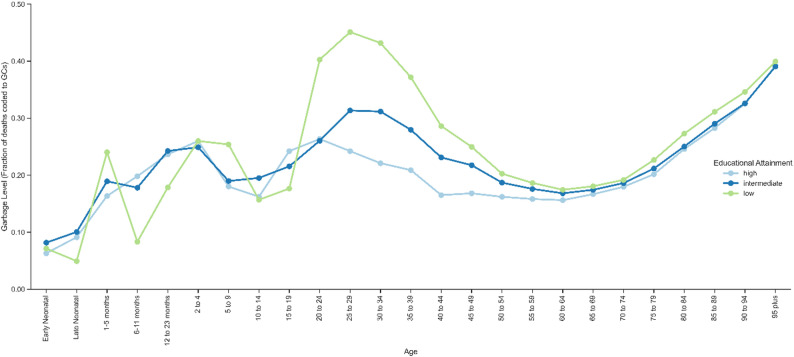
Level of GCs in Sweden by Age, 1997 to 2023

From 1997 to 2023, the highest number of GC coded deaths were assigned to those with the lowest educational level, with between 24.2% (in 2020) and 30.2% (in 2010) of deaths coded to GCs throughout the years (Fig. 1). In comparison, only between 19.4% (in 1998) and 25.1% (in 2011) of deaths among the highest educated were coded to GCs. While there was a 7.8% reduction in GC deaths assigned to the lowest educated reducing from 10 500 (10 300 − 10 700) deaths in 1997 to 9 680 (9 480-9 870) deaths in 2023, GCs assigned to the highest educated more than quadrupled from 561 (515–607) deaths to 2 600 (2 500-2 700) deaths despite less than 20% increase in the highest educated population in Sweden over the same time period (Table 1, Appendix Figure S10). For those who died before the age of 20, the educational group with the highest GC level varied with the lowest educated accruing the most GC deaths only at 1–5 months and 2 to 9 years (Fig. 2). After the age of 20, more GC deaths were assigned to those with lower education. This trend was most extreme among those who died between the ages of 20 to 24 (percent change of 89.4% more deaths than the highest educated), 25 to 29 (85.2% more), 30 to 34 (77.4% more), and 35 to 39 (68.7% more).

**Fig. 3:**
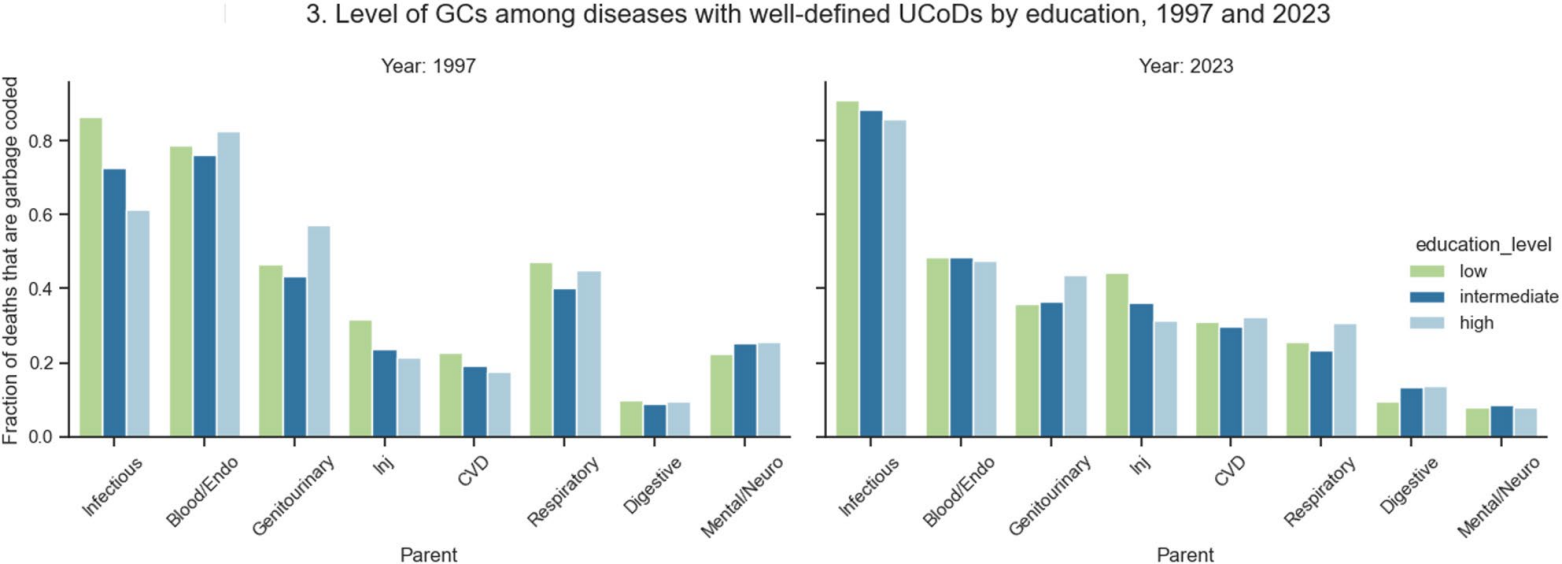
Level of GCs among diseases with well-defined UCoDs by education, 1997 and 2023

Across all years, GCs originated predominantly from ICD codes relating to cardiovascular diseases (CVD), all other ill-defined garbage, cancers, and respiratory diseases (Table 2, Appendix Table S5). Despite higher death counts among the lowest educated across all disease groups except for maternal, neonatal, and congenital (MNC) diseases, the distribution of GCs was similar across the education groups. Chi-squared testing indicated a statistically significant relationship between education and GC disease groups with increasing trends of GC deaths with decreasing educational attainment particularly visible among CVD, blood and endocrine, genitourinary, and respiratory diseases (Appendix Sect. 3.2). Of the disease groups with possible well-defined CoDs, the following had increasing trends of GC coding across all education groups from 1997 to 2023: CVD (37.0% increase among the low educated and 84.9% increase among the high educated), injuries (38.9% increase among the low educated and 47.5% among the high), and infections (5.6% increase among the low educated and 39.9% among the high) (Appendix Figure S2). The lowest educated had the highest percentage of deaths coded to GCs than well-defined CoDs in the following categories in 1997: infections, injuries, CVD, cancers, respiratory, digestive, and MNC diseases (Fig. 3). Despite reductions in the relative percentage of GC deaths in 2023, the lowest educated still had higher relative percentages of GCs in infections, injuries, cancers, blood and endocrine, and MNC diseases. As for the highest educated, there was a higher percentage of GCs in blood and endocrine, genitourinary, and mental and neurological diseases compared to the other education groups in 1997. By 2023, the percentage of infection, injury, CVD, digestive, and MNC disease deaths coded to GCs among the highest educated had all grown. In the same year, the highest educated had the greatest relative percentage of deaths coded to GCs in genitourinary, CVD, respiratory, and digestive diseases.

At the most detailed level, the following GCs were most assigned in deaths among the lowest educated in 2023: (i) heart failure unspecified right or left (1 470 deaths), (ii) all ill-defined codes for causes of death (916 deaths), and (iii) unspecified type of stroke (914 deaths) (Appendix Table S9). For deaths among the highest educated in 2023, the leading GCs were the same but were each assigned to 378 deaths or fewer. Additional results by age are available in Appendix Table S7.

**Table 2:** GC death counts and percent of all deaths by disease groups and highest educational attainment

**Disease group**	**High educated**		**Intermediate educated**		**Low educated**	
	**GC deaths**	**Percent of all deaths**	**GC deaths**	**Percent of all deaths**	**GC deaths**	**Percent of all deaths**
Blood/Endo	1 740 (1 660 - 1 820)	1.0 (1.0 - 1.1)	12 400 (12 200 - 12 600)	1.4 (1.4 - 1.4)	21 800 (21 500 - 22 100)	1.8 (1.7 - 1.8)
CVD	13 000 (12 800 - 13 200)	7.6 (7.5 - 7.8)	75 000 (74 400 - 75 500)	8.5 (8.5 - 8.6)	143 000 (142 000 - 143 000)	11.6 (11.6 - 11.7)
Cancer	5 980 (5 830 - 6 130)	3.5 (3.4 - 3.6)	29 600 (29 300 - 30 000)	3.4 (3.3 - 3.4)	39 300 (38 900 - 39 700)	3.2 (3.2 - 3.2)
Digestive	572 (525 - 619)	0.3 (0.3 - 0.4)	3 500 (3 390 - 3 620)	0.4 (0.4 - 0.4)	3 790 (3 670 - 3 910)	0.3 (0.3 - 0.3)
Genitourinary	547 (501 - 593)	0.3 (0.3 - 0.3)	3 210 (3 100 - 3 320)	0.4 (0.4 - 0.4)	6 040 (5 890 - 6 190)	0.5 (0.5 - 0.5)
Infectious	1 040 (980 - 1 110)	0.6 (0.6 - 0.6)	5 400 (5 250 - 5 540)	0.6 (0.6 - 0.6)	8 470 (8 290 - 8 650)	0.7 (0.7 - 0.7)
Inj	2 910 (2 810 - 3 020)	1.7 (1.6 - 1.8)	18 500 (18 200 - 18 700)	2.1 (2.1 - 2.1)	20 400 (20 200 - 20 700)	1.7 (1.6 - 1.7)
MNC	31 (20.1 - 41.9)	0.0 (0.0 - 0.0)	60 (44.8 - 75.2)	0.0 (0.0 - 0.0)	8 (2.46 - 13.5)	0.0 (0.0 - 0.0)
Mental/Neuro	1 880 (1 790 - 1 960)	1.1 (1.1 - 1.1)	9 380 (9 200 - 9 570)	1.1 (1.0 - 1.1)	13 000 (12 800 - 13 200)	1.1 (1.0 - 1.1)
Other	7 540 (7 370 - 7 720)	4.4 (4.3 - 4.5)	35 400 (35 000 - 35 700)	4.0 (4.0 - 4.1)	50 000 (49 500 - 50 400)	4.1 (4.0 - 4.1)
Respiratory	3 490 (3 380 - 3 610)	2.0 (2.0 - 2.1)	18 400 (18 100 - 18 600)	2.1 (2.1 - 2.1)	34 400 (34 000 - 34 700)	2.8 (2.8 - 2.8)

This table depicts counts and percents across all years, ages, and sexes. Blood/Endo = blood and endocrine diseases. CVD = cardiovascular diseases. Cancer = cancers. Digestive = digestive diseases. Genitourinary = genitourinary diseases. Infectious = infectious diseases. Inj = injuries. MNC = maternal, neonatal, and congenital diseases. Mental/Neuro = mental and neurological conditions. Respiratory = respiratory diseases

### Regional disparities

**Fig. 4:**
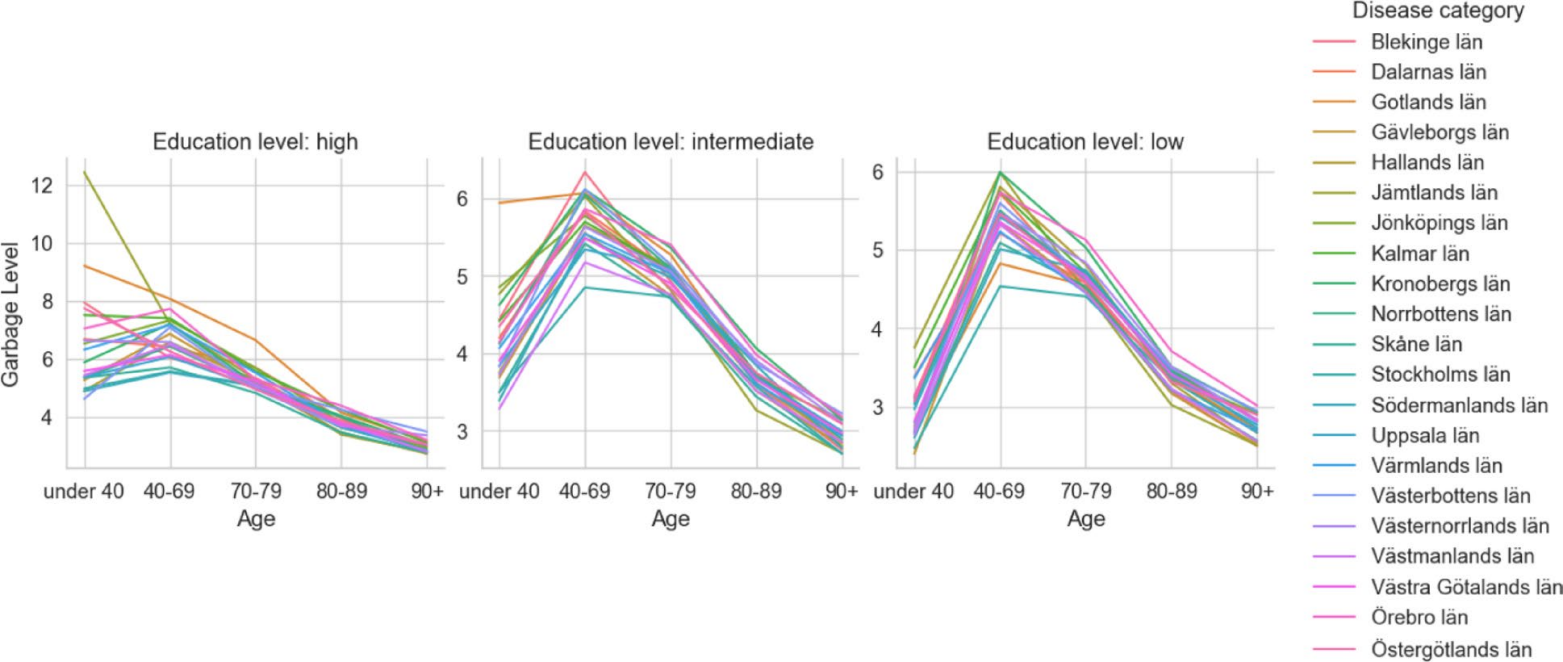
Garbage level by region and age, 1997 to 2023

All regions had at least 26.0% (95% CI: 24.3% to 27.6%) of deaths coded to GCs in 1997 (Appendix Table S12, figure S3). As of 2023, Skåne had the highest percentage of GCs with 27.5% (26.7% to 28.3%) followed by Stockholm (26.7% [26.0% to 27.4%]), Västra Götaland (26.0% [25.4% to 26.7%]), and the remaining regions of Sweden combined (24.5% [24.1% to 24.9%]) (Table 1). Similarly, the variation by age within the regions followed a consistent pattern (Fig. 4). By disease groups, the trend across all regions followed a similar pattern to the education trend with GCs predominantly in CVD, other garbage, cancers, and respiratory diseases (Appendix Table S6).

### Redistribution

After correction of GCs by redistribution, the underlying trend of well-defined CoD mortality differed more for the lowest educated groups compared to the highest educated. Across all years, the lowest educated experienced over 20% increases to the total number of deaths in 13 of the overall leading 20 CoDs in Sweden (as ranked by burden in 2023). The most affected CoDs were lower respiratory infections (increase of 924.7%, adding an additional 33 700 deaths after redistribution), diabetes (218.4% increase, adding 18 500 deaths), stroke (132.2% increase adding 70 600 deaths), falls (128.6% increase, adding 14 400 deaths), and chronic kidney disease (91.2% increase, adding 11 400 deaths) (Table 3). In 2023 alone, correction of GCs with redistribution changed the ranking of CoD burden among the lowest educated. Ischaemic heart disease surpassed Alzheimer’s disease and other dementias as highest rank, stroke surpassed chronic obstructive pulmonary disease and lung cancer to become third in rank, and lower respiratory infections were elevated from rank 31 st to 10th (Appendix Figure S9). The ranking changes were more severe in 1997 bringing falls, chronic kidney disease, diabetes mellitus, lower respiratory infections, and non-hodgkins lymphoma into the top 20 CoDs (Appendix Figure S8). The intermediate and highest educated groups experienced comparatively fewer rank adjustments (< 20) after redistribution, particularly in 1997 (Appendix Figures S4-5). Across all education groups, the ranking of CoDs remained similar in 2023 with CoDs changing rank at most by one to four places except for lower respiratory infections (rising into the top 20) and parkinson’s disease (falling out of the top 20 for all but the highest educated). For the full comparison of redistributed deaths, CoD rankings, and redistribution effects by year see Appendix Tables S8, S10-11.

**Table 3:** Cumulative Deaths from 1997–2023 before and after redistribution in the top 20 CoDs by highest educational attainment

Rank in 2023	Cause of death	Deaths pre-redistribution			Deaths post-redistribution		
		High education	Intermediate education	Low education	High education	Intermediate education	Low education
1	Ischemic heart disease	19 500 (19 300 − 19 800)	126 000 (125 000–127 000)	221 000 (220 000–222 000)	25 500 (25 200 − 25 800)	159 000 (158 000–159 000)	280 000 (279 000–281 000)
2	Alzheimer’s disease and other dementias	12 100 (11 900 − 12 400)	59 800 (59 300 − 60 300)	96 600 (96 000–97 200)	12 200 (12 000–12 400)	60 100 (59 600 − 60 500)	97 100 (96 500 − 97 700)
3	Tracheal, bronchus, and lung cancer	6 620 (6 460–6 780)	42 000 (41 600 − 42 400)	43 900 (43 500 − 44 300)	7 450 (7 290–7 620)	46 200 (45 800 − 46 600)	49 200 (48 700 − 49 600)
4	Stroke	6 220 (6 070 − 6 380)	33 900 (33 500 − 34 300)	53 400 (52 900 − 53 800)	12 000 (11 800 − 12 200)	67 900 (67 400 − 68 400)	124 000 (124 000–125 000)
5	Colon and rectum cancer	6 840 (6 680–7 010)	30 000 (29 600 − 30 300)	33 000 (32 700 − 33 400)	7 970 (7 800–8 150)	36 100 (35 800 − 36 500)	42 100 (41 700 − 42 600)
6	Chronic obstructive pulmonary disease	2 980 (2 870–3 080)	26 000 (25 700 − 26 400)	39 400 (39 000–39 800)	3 530 (3 420–3 650)	28 900 (28 600 − 29 200)	44 400 (44 000–44 900)
7	Severe acute respiratory syndrome coronavirus 2	2 030 (1 940–2 120)	9 140 (8 950–9 330)	8 690 (8 510–8 870)	2 400 (2 300–2 500)	10 900 (10 700 − 11 100)	10 500 (10 300 − 10 700)
8	Chronic kidney disease	1 440 (1 360–1 510)	7 740 (7 570–7 920)	12 500 (12 300 − 12 700)	2 640 (2 540–2 740)	14 300 (14 100 − 14 600)	23 900 (23 600 − 24 200)
9	Atrial fibrillation and flutter	3 370 (3 250–3 480)	17 600 (17 400 − 17 900)	31 500 (31 100 − 31 800)	3 370 (3 250–3 480)	17 700 (17 400 − 17 900)	31 500 (31 200 − 31 900)
10	Prostate cancer	6 140 (5 990–6 290)	24 500 (24 200 − 24 800)	30 800 (30 500 − 31 100)	6 910 (6 750–7 070)	28 100 (27 800 − 28 400)	36 000 (35 600 − 36 400)
11	Lower respiratory infections	449 (407–491)	2 270 (2 170–2 360)	3 640 (3 520–3 760)	3 710 (3 590–3 830)	19 700 (19 400 − 19 900)	37 300 (37 000–37 700)
12	Falls	1 780 (1 690–1 860)	8 550 (8 370–8 730)	11 200 (11 000–11 400)	3 330 (3 210–3 440)	16 900 (16 600 − 17 100)	25 600 (25 300 − 25 900)
13	Pancreatic cancer	4 740 (4 600–4 870)	19 900 (19 600 − 20 200)	18 700 (18 400 − 19 000)	5 300 (5 160–5 440)	22 900 (22 600 − 23 200)	22 600 (22 300 − 22 900)
14	Hypertensive heart disease	1 200 (1 140–1 270)	7 110 (6 940–7 270)	12 800 (12 600 − 13 000)	1 930 (1 840–2 010)	11 100 (10 900 − 11 300)	20 500 (20 300 − 20 800)
15	Self-harm	3 330 (3 220–3 450)	15 700 (15 500 − 16 000)	9 040 (8 850–9 220)	4 340 (4 210–4 470)	21 100 (20 800 − 21 400)	12 100 (11 900 − 12 300)
16	Breast cancer	5 090 (4 950–5 230)	17 300 (17 000–17 500)	15 200 (15 000–15 400)	5 460 (5 320–5 610)	19 500 (19 200 − 19 700)	18 400 (18 100 − 18 700)
17	Diabetes mellitus	862 (804–920)	5 530 (5 390–5 680)	8 480 (8 300–8 660)	2 250 (2 150–2 340)	15 500 (15 200 − 15 700)	27 000 (26 700 − 27 300)
18	Non-rheumatic valvular heart disease	1 270 (1 200–1 340)	6 940 (6 770–7 100)	12 200 (12 000–12 400)	1 810 (1 730–1 900)	9 860 (9 660 − 10 100)	17 800 (17 600 − 18 100)
19	Aortic aneurysm	1 940 (1 860–2 030)	10 400 (10 200 − 10 600)	13 400 (13 200 − 13 600)	2 210 (2 110–2 300)	11 800 (11 600 − 12 100)	15 600 (15 300 − 15 800)
20	Cirrhosis and other chronic liver diseases	1 210 (1 150–1 280)	8 930 (8 740–9 110)	7 230 (7 060 − 7 390)	1 470 (1 400–1 550)	10 600 (10 400 − 10 900)	8 700 (8 510–8 880)

The leading 20 CoDs were defined as the CoDs with the greatest fatal burden in 2023, all ages and both sexes

### Model results

After validation there was significant evidence of an education effect on GC death coding after controlling for age at death, sex, and region of death. After standardization, the most influential factor was age at death with 25.2% (24.8%−25.6%) higher odds of GC assignment. That said, education was the second-most influential factor. We estimated 12.8% (11.4%−14.2%) higher odds of assigning a GC coded death among deceased with lower education compared to those with higher education taking into account sex, age at death, and region of death (Table 4). This point estimate exceeded the effect by age at death at 5-year intervals (1.5% [1.5%−1.5%] greater for each additional year of age), sex (12.1% [11.4%−12.8%] greater for males compared to females), and region (7.3% [6.6%−8.1%] lower for dying in the other regions compared to Stockholm, less for Skåne and Västra Götaland). Data summary statistics and model validation are available in Appendix Sects. 3 and 4.

**Table 4:** Model validation results

Covariate	Odds Ratio	CI Lower (95%)	CI Upper (95%)	Significant (*p* < = 0.05)
Intercept	0.084	0.082	0.086	*
Age at death (5-year)	1.078	1.076	1.079	*
Age at death (10-year)	1.161	1.159	1.164	*
Age at death (15-year)**	1.252	1.248	1.256	*
Female	Reference			
Male	1.121	1.114	1.128	*
High education	Reference			
Intermediate education	1.043	1.03	1.056	*
Low education	1.128	1.114	1.142	*
Stockholm	Reference			
Skåne	1.011	1.00	1.022	
Västra Götaland	0.932	0.923	0.942	*
Other regions	0.927	0.919	0.934	*

We ran three models separating the 3 age intervals (5 years, 10 years, and 15 years). The effect of the other covariates remained the same and were thus not repeated in the table. **The age effect for the 15-year interval reflects standardization by 1 standard deviation for covariate comparison

## Discussion

To our knowledge, we present the first comprehensive assessment of GC level by highest educational attainment, sex, age, and region, from 1997 to 2023 in Sweden. Overall, GC level has decreased by 3.5% since 1997, standing at 25.5% in 2023. This is motivated by modest improvements in GC level among the lowest educated, but progress has been curbed by worsening GC coding among the highest educated. Even still, GCs are assigned predominantly to the lowest educated throughout the timeseries, an effect further exacerbated by age where those with low education had over 45.8% more GCs relative to well-defined deaths than the highest educated among those who died between the ages of 20 to 39. After redistribution, the number of deaths among the lowest educated increased by over 20% in 13 of the leading 20 CoDs in Sweden (as ranked in 2023). This raises concerns about both equitable representation and overall death coding quality in Sweden, in spite of substantial improvements in healthcare quality over the last 26 years [12].

Past explanations of GC deaths have focused primarily on the challenges of CoD coding, such as the difficulty of assigning a single UCoD in the presence of multiple comorbidities, lack of or insufficient detail in patient history records, declining number of autopsies, and/or insufficient physician training in CoD certification [3, 5, 6]. However, after controlling for age at death, sex, and region of death, educational attainment was the second-most influential factor in whether a death was coded to a GC with a 12.8% (11.4%−14.2%) higher likelihood of having a doctor assign a GC to those with lower education. This suggests that comorbidities (which tend to increase with patient age), healthcare quality and physician training in completing death certificates (which could differ by location) are not alone sufficient to explain GC levels in Sweden [ 13, 14, 15]. Lower educated individuals have been shown to have a higher likelihood of behavioral factors that may lower health, increasing the risk of additional comorbidities, and poorer health literacy leading to inadequate care seeking and/or poor communication with their physician [16–19]. These factors likely interact with the prior drivers of GC coding and may prove to be points of intervention.

Our findings show an unexpectedly high proportion of GCs in the 20–39 age group, especially among the lowest educated. One potential explanation is the high proportion of drug overdose in this age group, as substance abuse tends to be more prevalent in this age and among individuals with lower education [20, 21]. Overdose often presents challenges in classifying the manner of death (accident, suicide or homicide) and could explain the high number of GCs [22]. Another reason for the high proportion of GCs in younger individuals may be the frequent use of the R99 code (unknown cause of death, e.g., other ill-defined and unspecified causes of death) in Swedish death certificates, as demonstrated by other garbage being the second-most common across all education groups. Although this code is not more prevalent in young adults compared to older adults, its relative impact is more pronounced due to the higher proportion of forensic autopsies performed in this age group. It is plausible that forensic pathologists are more inclined to assign R99 in cases where decomposition or other factors prevent a meaningful autopsy result [23]. In contrast, physicians certifying deaths without autopsy may prefer to assign a presumed natural cause, such as heart disease, even in the absence of definitive evidence [24]. Unlike Sweden, Finland has the lowest garbage coding in the world as of 2023, likely due its centralized system for death certificate review [25]. Unlike many countries, Finland employs forensic pathologists at the national health institute (THL) who review all death certificates, including those not involving forensic autopsies [26]. This systematic oversight allows for the correction of vague or inconsistent entries prior to ICD coding, thereby improving data quality. The Finnish model demonstrates how structured medical review can significantly reduce the prevalence of GCs in national mortality statistics. It is true that in some cases, a well-defined CoD is impossible to determine for reasons such as extensive decomposition or undetermined intent. In these instances, it is important that physicians are not pressured to invent a well-defined CoD to avoid assigning a GC. However, GC reduction is still possible through systematic improvements in physician awareness and standardizing access of patient information to death certifiers.

While prior literature has identified more modest effects of education on assignment of ill-defined causes-of-death [7], such literature focused predominantly on exclusively ill-defined GCs limited to R00-R99 codes. Our study takes a broader definition of GCs which incorporates ill-defined codes alongside GCs originating from disease-specific ICD chapters. The leading disease groups for GCs in Sweden are cardiovascular disease, cancer, injury, respiratory, and blood and endocrine disease related GCs. All of these groups predominantly have GCs related to (i) intermediate and immediate CoDs that become GCs when they are misassigned as the UCoD and (ii) unspecified type GCs which are too non-specific to be considered well-defined causes of death. When we consider the percentage of all deaths in each group coded to GCs instead of well-defined UCoDs, we find modest improvements over time among the lowest educated but worsening assignment among many groups for the higher educated. This is particularly evident among infectious diseases, which may have been impacted in recent years from the COVID-19 pandemic. The high prevalence of GCs arising from misassigned intermediate and immediate CoDs could be addressed through improved physician training in CoD certification since these GCs can be avoided by not attributing intermediate and immediate causes to UCoDs [27]. Additional safeguards to CoD assignment could be introduced through fine-tuning Sweden’s electronic coding system. In Norway, since the introduction of an electronic cause of death coding system in 2021 with mandatory online reporting and drop-down menus with diagnoses from ICD-10, death register quality has shown significant improvement in reducing coding of unspecified disease [28]. As for addressing unspecified type GCs, it is important to ensure adequate data collection for physicians and healthcare systems to be able to provide information on a more specific CoD. This can be remedied through improvements in provision of data to forensic pathologists, increase in autopsies, increased patient information gathering such as through surveys, and thorough review of patient health records [29–33]. Promisingly, our results showing regional homogeneity in Sweden indicate that nationally implemented interventions could prove beneficial in reducing death coding inequality across all regions. This would mitigate the need for initial region-based tailoring of national cause-of-death coding practice improvements. That said, implementation and guidance of interventions would benefit from regional expertise.

Redistribution of GC deaths is a necessary step for inclusion and comparability of data, otherwise the lower educated would have disproportionately misclassified or excluded deaths in CoD analyses in Sweden. Of course, due to the greater number of GCs among the lowest educated, this education group also had the greatest change in mortality trends after redistribution. This significantly affected lower respiratory infections which led to this cause being considered one of the top 20 causes rather than 31st. When we consider the ranking of causes overall across all education groups, there were many minimal changes in ranking with ranks often increasing or decreasing by no more than 4 places. Given that CoD ranking and mortality trends are critical for public health as well as clinical planning and research [4], it is important that they are correct and representative. In an ideal setting, redistribution of GCs would not be needed, and the raw data could be utilized without concern for systematic educational bias impacting public health planning.

This study builds upon the work performed by the Institute for Health Metrics and Evaluation in production of the GBD. As a result, our study has several strengths and limitations. We utilized the GBD classification of GCs which counts the ICD-10 code X42 as a GC when it is not considered one by the World Health Organization (WHO). We also utilized intermediate and immediate cause GBD redistribution methods which do not include Swedish data due to the requirements of line-level, patient identifiable data accessible to the GBD. Furthermore, GBD redistribution methods often incorporate age and sex in redistribution percentages which may account for the reduced effect of these covariates compared to education. It is also important to note that mild overlap in the confidence intervals between our education and sex covariates suggest that the difference in GC coding between the two covariates may not be of strong importance. Future iterations of this work should strive for incorporation of Swedish data as well as education strata in these redistribution methods to improve fit and applicability. Additionally, not all GCs can be redistributed using the most robust method of redistribution due to data limitations and it is important to acknowledge that redistribution represents a statistical correction rather than a method for identifying the “true” UCoD. With regard to sociodemographic inequalities, it is important to note that we assess education and not income, country of birth, and other known factors that could influence the results if considered separately. For the region-based analysis, we did not incorporate covariates to address variations in urbanicity and economic status and we combined the 18 least populous regions together as a simplification to reduce overfitting. Future analyses by region would benefit from more granular analysis to address these potential confounders. That said, a strength is that the GBD cause list is extensively peer reviewed and comprehensive and mutually exclusive. It further provides a means to summarize ICD-10 codes by disease group. Another strength of this study is that it was conducted in Sweden where there is universal health coverage and, in principle, good access to care [34]. This diminishes systemic barriers to healthcare that may otherwise confound a study of this nature. Given that we still see stark disparities by education in Sweden, a country well regarded for its healthcare quality, we believe it is of global interest for countries to assess their GC levels for sociodemographic disparities where barriers to healthcare may exacerbate the problem further.

## Conclusion

Quality cause of death coding is necessary for mortality research, but inaccurately coded deaths lead to systematic biases in reporting particularly affecting the lowest educated. Since 1997, at least 23% of deaths per year have been misassigned in Sweden. These misassigned deaths were consistently more common among the lowest educated after accounting for region, sex, and age. This disproportionately miss-classifies deaths from the lowest educated, directly affecting public health as well as clinical research and monitoring. Moreover, these inaccuracies and biases are occurring in Sweden, a country well-regarded for healthcare quality, indicating that this problem is globally widespread. Addressing sociodemographic inequalities in GCs is necessary as a part of existing efforts to improve data quality through improvements in guidelines and systems for death certification as well as ensuring equal access to health services and appropriate care. Our study strives to raise awareness of educational disparities in garbage coded deaths with the goal of promoting equitable representation of all socioeconomic groups in health research.

## Supplementary Information

Supplementary Material 1

## Data Availability

Due to ethical restrictions, the individual level data used in this study are not publicly available. However, the data supporting the findings of this study can be obtained from the following sources: Statistics Sweden ([www.scb.se](http:/www.scb.se)) and The Swedish National Board of Health and Welfare ([www.socialstyrelsen.se](http:/www.socialstyrelsen.se))

## Abbreviations

CoD: Cause of death
GBD: Global Burden of Disease
ICD: International Classification of Diseases
UCoD: Underlying cause of death
GC: Garbage code
YLD: Years lived with disability
