# Supplementary material for: Regional and educational disparities in inaccurately coded deaths in Sweden, 1997–2023: a systematic analysis
Source: Popul Health Metr. 2026 Mar 18;24:25. doi: 10.1186/s12963-026-00471-8 (PMC13063463; doi:10.1186/s12963-026-00471-8)
Supplement: Supplementary file 1 — Supplementary Material 1 [file 12963_2026_471_MOESM1_ESM.docx]

Appendix 1: supplementary methods and results to “Regional and Educational Disparities in Misassigned and Inaccurately Coded Deaths in Sweden, 1997-2023: A systematic analysis”

[**Section 1: List of abbreviations** 1](#_Toc205299869)

[**Section 2: Redistribution** 1](#_Toc205299870)

[**Section 2.1.1: Multiple cause analysis** 2](#_Toc205299871)

[**Section 2.1.2: Handling unspecified injuries: X59 & Y:34** 2](#_Toc205299872)

[**Section 2.2: Negative Correlation** 3](#_Toc205299873)

[**Section 2.3: Impairments** 3](#_Toc205299874)

[**Section 2.4: Proportional redistribution** 3](#_Toc205299875)

[**Section 3: Model validation** 3](#_Toc205299876)

[**Section 3.2: Model results** 4](#_Toc205299877)

[**Table 3.2.1 Odds ratios and model results** 4](#_Toc205299878)

[**Section 4: Summary statistics** 4](#_Toc205299879)

[**Section 5: References** 4](#_Toc205299880)

[**Section 6: Tables and figures** 4](#_Toc205299881)

# **Section 1: List of abbreviations**

| **Abbreviation** | **Full phrase** |
| --- | --- |
| CoD | Cause of Death |
| GBD | Global Burden of Diseases, Injuries, and Risk Factors Study |
| ICD | International Classification of Diseases |
| MCoD | multiple causes of death data |
| UCoD | underlying cause of death |
| VR | vital registration |
| YLD | Years lived with disability |

# **Section 2: Redistribution**

The following redistribution methods adhere to the methods previously published and described in-detail^1^. The methods described here are paraphrased adaptations of those published in 2021.

Redistribution is a method of fractionally reallocating garbage coded deaths to well-defined causes of death (CoD) compatible with the Global Burden of Disease (GBD) project cause hierarchy. The important components of redistribution analysis are: (i) identifying the target well-defined CoDs and (ii) determining the fraction of the garbage coded deaths to redistribute onto each target CoD.

## **Section 2.1.1: Multiple cause analysis**

We inform the multiple cause analysis with multiple cause of death (MCoD) data which provides information on both the assigned underlying cause of death (UCoD) and intermediate and immediate causes of death in the death record. In our framework, an ICD code is a GC when coded to the UCoD position. Therefore, MCoD data helps us identify useful patterns of the other ICD codes that co-occur with GCs. These patterns can then be used to reassign GCs to well-defined UCoDs.

The first step of the analysis is to map the data and identify all GC coded deaths. We collate all GC ICD codes improperly assigned to the UCoD position. Then we gather the rows of data where the same GC ICD codes occur properly in the intermediate and immediate cause positions of the record rather than in the UCoD position. This forms the set of all possible well-defined UCoD targets that the GC deaths could have been assigned to. To avoid outliers, we trim the list of possible well-defined UCoDs to those that comprised 80% deaths and use a least absolute shrinking and selection operator (LASSO) regression to apply a penalty to the possible UCoDs in the bottom 20% of deaths. This process leaves us with our target UCoDs for GC redistribution.

Next, we run a mixed effects binomial logistic regression for each GC to estimate the fraction of well-defined UCoD deaths that co-occur with the GC ICD code. As an example, if sepsis is our GC of interest, and its well-defined UCoD target is ischaemic heart disease, we run this model to determine what fraction of ischaemic heart disease deaths that also include sepsis in the intermediate and/or immediate cause position. This model helps us understand the distribution of the GC ICD code among its well-defined UCoD targets. We specify this model as:

$$Y_{i} \sim B\left( n_{i}, \pi_{i} \right)$$

$$logit \left( \pi_{i} \right)=\beta_{0}+\beta_{1}\cdot X+\beta_{age}+\beta_{sex}+\gamma_{underlying cause}$$

Where $Y_{i}$ is the proportion of deaths co-occurring with the GC of interest. $n_{i}$ is the number of observations, $\pi_{i}$ is the probability of a GC-related death for each age, sex, location, year, and underlying cause group *i*. $\beta_{0}$ is the intercept, $\beta_{1}$ the effect of X covariates, $\beta_{age}$ and $\beta_{sex}$ are categorical covariates for age group and sex, and $\gamma_{underlying cause}$ is a random effect by UCoD where UCoDs with the same parent can borrow strength across each other.

We then determine the number of GC deaths that should have been categorized as each possible target. To do this, we multiply the resulting fraction from the model by the GBD calculation of the well-defined UCoD deaths by age, sex, location, and year. We sum all of these deaths to create a total number of GC deaths. The final fraction that is used for redistribution is the cause-specific deaths miscategorized as a GC over the total GC deaths. Below is an example:

$$sepsis deaths_{a, s, l, y, ucod}=modeled sepsis fraction_{a, s, l, y, ucod}\cdot GBD deaths_{a, s, l, y, ucod}$$

$$total sepsis deaths = \sum_{ucod_{i}}^{n} sepsis deaths_{a, s, l, y, ucod}$$

$$proportion of sepsis to redistribute = \frac{sepsis deaths_{a, s, l, y, ucod}}{total sepsis deaths_{a, s, l, y}}$$

## **Section 2.1.2: Handling unspecified injuries: X59 & Y:34**

X59 and Y34 are common non-specific ICD codes that describe exposure to “unspecified factors” and “unspecified event of undetermined intent” respectively. The first step to handling these causes is to collect deaths with X59 or Y34 listed as the UCoD as well as well-defined injury deaths. For each death, the combinations of nature of injury codes appearing in the chain are related to 37 custom groups of diagnostically related ICD codes. We used the top 95% combinations to create cause, age, sex, year, and location-specific redistribution proportions.

## **Section 2.2: Negative Correlation**

The most specific garbage-coded deaths do not typically co-occur with less-detailed codes, making them near impossible to perform the mcause analysis on. Therefore, we use negative correlation. This method takes advantage of a negative trend wherein there is an inverse relationship between the number of garbage coded deaths and its target UCoDs. In other words, if garbage coded deaths are higher, the target UCoD deaths appear lower in number.

First, the target UCoDs are assumed *a priori*. We logit transformed the proportion of each individual target UCoD out of all the possible target UCoD deaths. We modelled this proportion linearly as a function of covariates predictive of the target UCoD mortality. This function takes the form:

$$logit X_{i}\sim N \left( \mu_{i}, \sigma^{2} \right)$$

$$\mu_{i}=\beta_{0}+\beta_{1}X_{i}$$

Where $\beta_{1}X_{i}$ are the covariates predictive of the target UCoD mortality. We ran unique models by age, sex, location, and year. In cases of bias in the residuals, additional adjustments are applied.

## **Section 2.3: Impairments**

As defined by the GBD, impairments are domains of health loss that are the consequence of multiple underlying causes rather than the underlying causes themselves. A routine part of the GBD is estimating the years lived with disability (YLDs) for these impairments. All UCoDs are plausible target underlying causes. We calculate these redistribution proportions as the number of cause-specific YLDs for a given impairment over the sum of YLDs across all causes for each age group, sex, location, and year. Then, deaths incorrectly assigned to impairments are redistributed onto target UCoDs using these redistribution proportions.

## **Section 2.4: Proportional redistribution**

This method calculates the distribution of the target UCoDs in the death data directly. The proportion of the deaths that are each target UCoD are then used as the redistribution proportions for the garbage coded deaths. We only use this method for the least specific class of garbage coded deaths such as “all ill-defined causes of death”.

## **Section 2.5: Mental Disroders and HIV correction packages**

There are two minor redistribution packages with limited use-cases compared to the prior four methods. The first is the Linkage package which is only used to redistribute mental disorders. This package functions by redistributing according to the proportions of causes of death found among patients in the Brazilian public health system between 2000 and 2015. The proportions were taken:

1. By age and sex. The age groups used were 15-29, 30-59, and 60+
2. Among patients diagnosed with ICD codes F04-F09, F20-F49, or F51-F99.
3. Among patients with at least 1+ months of exposure
4. Among level 3 causes
5. Among causes where the relative risk of dying was statistically significant and above 1.001
6. Among causes where at least 100 people died of the cause

The second is the suite of HIV correction packages which do not feature significantly in Sweden as they are employed to capture HIV hidden in causes thought to have hidden HIV burden. This is limited to neglected tropical disease and immunodeficiency-based ICD codes and was used to correct HIV in GBD 2023.^7^

# **Section 3: Validation**

## **Section 3.1: Binomial logistic regression validation**

Our models had an accuracy of 73.6% but an AUC score of 0.58 indicating better than random binary assignment of garbage coded deaths to well-defined deaths. However, this is still less than optimal discernment. Future analyses, especially those that rely on modelling for imputation, should explore additional interaction terms and/or machine learning methods to improve discernment.

### **Section 3.2: Chi-square testing**

We conducted a chi-square test between the highest educational attainment and the GC categories to determine if there was a significant relationship between these variables. Our chi-square test returned with a value of 5 908.8 (p-value=0.0), indicating a significant relationship and a trend of growing garbage code accumulation as highest educational attainment drops particularly among the following GC categories: blood/endo, CVD, genitourinary, and respiratory. Below is our contingency table showcasing row percentages of deaths.

**Table 3.2.1** Percentages of GC deaths by GC category and education level, All years, all ages, all regions, and all sexes

|  | GC category | | | | | | | | | | |
| --- | --- | --- | --- | --- | --- | --- | --- | --- | --- | --- | --- |
| Education | Blood/Endo | CVD | Cancer | Digest | Genito  urinary | Infectious | Inj | MNC | Mental/Neuro | Other | Respiratory |
| high | 4.48 | 33.60 | 15.42 | 1.48 | 1.41 | 2.69 | 7.52 | 0.08 | 4.84 | 19.47 | 9.01 |
| intermediate | 5.87 | 35.58 | 14.05 | 1.66 | 1.52 | 2.56 | 8.76 | 0.03 | 4.45 | 16.79 | 8.71 |
| low | 6.40 | 42.00 | 11.56 | 1.11 | 1.78 | 2.49 | 6.01 | 0.00 | 3.82 | 14.70 | 10.11 |

# **Section 4: Summary statistics**

Our data included 2.50 million deaths across a 26 year time period from 1997 to 2023. 49.6% of the deaths were female. By age, 98.5% of deaths occurred in those that died at the age of 50 or older. By location, 17.2% of the deaths were in Stockholm, 13.0% were in Skåne, 16.4% of deaths in Västra Götaland, and 53.4% in the other regions. By education, 53.9% died belonging to the group with the lowest educational attainment followed by 38.6% with intermediate educational attainment and 7.5% with highest educational attainment. There were 214 000 persons with unknown educational attainment making up a total of 92 400 GCs from 1997 to 2023. 89.0% of these persons were born in or prior to 1940. Due to limited data quality during the early 1900s, we chose to exclude the persons with unknown education from the education-focused analyses.

## **Section 4.1: Assignment of education**

We assigned each individual’s education as the highest attained education for those over the age of 25 and assigned the highest attained parental education for those <= 25 if the individual education was not higher. The assignment of parental education was motivated by research indicating that higher parental education improves aspects of child health outcomes both in Sweden and other countries.^2,3,4^ The cut off of 25 was established because: (i) In a 2024 report of educational attainment in Sweden, those aged 25-34 made up the largest percentage of the population with post-secondary education.^5^ (ii) Prior research set a precedent of assigning parental education to those aged 28 and younger.^6^ (iii) In our data, there was minimal difference between adjusting for those <= 28 years of age (leading to a correction of 20.7% of the total population data) and adjusting for those <= 25 years of age (correcting 19.2%). We opted for a cut-off of 25 years of age which accounted for the lower cut-off of the population most educated and led to the minimum number of corrections in our dataset.

# **Section 5: References**

1. Johnson, S.C., Cunningham, M., Dippenaar, I.N. *et al.* Public health utility of cause of death data: applying empirical algorithms to improve data quality. *BMC Med Inform Decis Mak* 21, 175 (2021). <https://doi.org/10.1186/s12911-021-01501-1>
2. Schäfer Elinder, L., Heinemans, N., Zeebari, Z., & Patterson, E. (2014). Longitudinal changes in health behaviours and body weight among Swedish school children – associations with age, gender and parental education: The SCIP school cohort. BMC Public Health, 14, 640. [https://bmcpublichealth.biomedcentral.com/articles/10.1186/1471-2458-14-640](https://bmcpublichealth.biomedcentral.com/articles/10.1186/1471-2458-14-640?utm_source=chatgpt.com)
3. Rasciute, S. (2024). Parental education and child health: The exploration of the cross-gender intergenerational transmission mechanism. Kyklos, 77(2), [page range]. <https://doi.org/10.1111/kykl.12344>
4. Balaj, M., York, H. W., Sripada, K., Besnier, E., Vonen, H. D., Aravkin, A., Friedman, J., Griswold, M., Jensen, M. R., Mohammad, T., Mullany, E. C., Solhaug, S., Sørensen, R., Stonkute, D., Tallaksen, A., Whisnant, J., Zheng, P., Gakidou, E., & Eikemo, T. A. (2021). Parental education and inequalities in child mortality: A global systematic review and meta-analysis. The Lancet, 398(10300), 608-620. <https://doi.org/10.1016/S0140-6736(21)00534-1>
5. Statistics Sweden. (2025). Befolkningens utbildning 2024: Temarapport 2025:3. Statistiska centralbyrån. [https://www.scb.se/contentassets/29b6a64dcf684f1887d6f64ec7a0b479/uf0506_2024a01_br_a40br2504.pdf](https://www.scb.se/contentassets/29b6a64dcf684f1887d6f64ec7a0b479/uf0506_2024a01_br_a40br2504.pdf?utm_source=chatgpt.com)
6. Li, B., Allebeck, P., Burstöm, B., Danielsson, A. K., Degenhardt, L., Eikemo, T. A., Ferrari, A., Knudsen, A. K., Lundin, A., Manhica, H., Newton, J., Whiteford, H., Flodin, P., Sjöqvist, H., & Agardh, E. E. (2023). Educational level and the risk of mental disorders, substance use disorders and self-harm in different age-groups: A cohort study covering 1,6 million subjects in the Stockholm region. International journal of methods in psychiatric research, 32(4), e1964. <https://doi.org/10.1002/mpr.1964>
7. Hay, S. I., Ong, K. L., Santomauro, D. F., Aalipour, M. A., Aalruz, H., Ababneh, H. S., ... & Ajose, A. O. (2025). Burden of 375 diseases and injuries, risk-attributable burden of 88 risk factors, and healthy life expectancy in 204 countries and territories, including 660 subnational locations, 1990–2023: a systematic analysis for the Global Burden of Disease Study 2023. The Lancet.

# **Section 6: Tables and figures**

Table S1: GBD cause hierarchy with levels

|  |  |
| --- | --- |
| GBD Cause Name | Level |
| All causes | 0 |
| Communicable, maternal, neonatal, and nutritional diseases | 1 |
| HIV/AIDS and sexually transmitted infections | 2 |
| HIV/AIDS | 3 |
| HIV/AIDS - Drug-susceptible Tuberculosis | 4 |
| extensive drug resistance | 4 |
| HIV/AIDS - Extensively drug-resistant Tuberculosis | 4 |
| HIV/AIDS resulting in other diseases | 4 |
| Sexually transmitted infections excluding HIV | 3 |
| Syphilis | 4 |
| Chlamydial infection | 4 |
| Gonococcal infection | 4 |
| Trichomoniasis | 4 |
| Genital herpes | 4 |
| Other sexually transmitted infections | 4 |
| Respiratory infections and tuberculosis | 2 |
| Tuberculosis | 3 |
| Latent tuberculosis infection | 4 |
| Drug-susceptible tuberculosis | 4 |
| resistance | 4 |
| Extensively drug-resistant tuberculosis | 4 |
| Lower respiratory infections | 3 |
| Upper respiratory infections | 3 |
| Otitis media | 3 |
| COVID-19 | 3 |
| Enteric infections | 2 |
| Diarrheal diseases | 3 |
| Typhoid and paratyphoid | 3 |
| Typhoid fever | 4 |
| Paratyphoid fever | 4 |
| Invasive Non-typhoidal Salmonella (iNTS) | 3 |
| Other intestinal infectious diseases | 3 |
| Neglected tropical diseases and malaria | 2 |
| Malaria | 3 |
| Chagas disease | 3 |
| Leishmaniasis | 3 |
| Visceral leishmaniasis | 4 |
| Cutaneous and mucocutaneous leishmaniasis | 4 |
| African trypanosomiasis | 3 |
| Schistosomiasis | 3 |
| Cysticercosis | 3 |
| Cystic echinococcosis | 3 |
| Lymphatic filariasis | 3 |
| Onchocerciasis | 3 |
| Trachoma | 3 |
| Dengue | 3 |
| Yellow fever | 3 |
| Rabies | 3 |
| Intestinal nematode infections | 3 |
| Ascariasis | 4 |
| Trichuriasis | 4 |
| Hookworm disease | 4 |
| Food-borne trematodiases | 3 |
| Leprosy | 3 |
| Ebola | 3 |
| Zika virus | 3 |
| Guinea worm disease | 3 |
| Other neglected tropical diseases | 3 |
| Other infectious diseases | 2 |
| Meningitis | 3 |
| Encephalitis | 3 |
| Diphtheria | 3 |
| Pertussis | 3 |
| Tetanus | 3 |
| Measles | 3 |
| Varicella and herpes zoster | 3 |
| Acute hepatitis | 3 |
| Acute hepatitis A | 4 |
| Acute hepatitis B | 4 |
| Acute hepatitis C | 4 |
| Acute hepatitis E | 4 |
| Other unspecified infectious diseases | 3 |
| Maternal and neonatal disorders | 2 |
| Maternal disorders | 3 |
| Maternal hemorrhage | 4 |
| Maternal sepsis and other maternal infections | 4 |
| Maternal hypertensive disorders | 4 |
| Maternal obstructed labor and uterine rupture | 4 |
| Maternal abortion and miscarriage | 4 |
| Ectopic pregnancy | 4 |
| Indirect maternal deaths | 4 |
| Late maternal deaths | 4 |
| Maternal deaths aggravated by HIV/AIDS | 4 |
| Other direct maternal disorders | 4 |
| Neonatal disorders | 3 |
| Neonatal preterm birth | 4 |
| Neonatal encephalopathy due to birth asphyxia and trauma | 4 |
| Neonatal sepsis and other neonatal infections | 4 |
| Hemolytic disease and other neonatal jaundice | 4 |
| Other neonatal disorders | 4 |
| Nutritional deficiencies | 2 |
| Protein-energy malnutrition | 3 |
| Iodine deficiency | 3 |
| Vitamin A deficiency | 3 |
| Dietary iron deficiency | 3 |
| Other nutritional deficiencies | 3 |
| Non-communicable diseases | 1 |
| Neoplasms | 2 |
| Lip and oral cavity cancer | 3 |
| Nasopharynx cancer | 3 |
| Other pharynx cancer | 3 |
| Esophageal cancer | 3 |
| Stomach cancer | 3 |
| Colon and rectum cancer | 3 |
| Liver cancer | 3 |
| Liver cancer due to hepatitis B | 4 |
| Liver cancer due to hepatitis C | 4 |
| Liver cancer due to alcohol use | 4 |
| Liver cancer due to NASH | 4 |
| Hepatoblastoma | 4 |
| Liver cancer due to other causes | 4 |
| Gallbladder and biliary tract cancer | 3 |
| Pancreatic cancer | 3 |
| Larynx cancer | 3 |
| Tracheal, bronchus, and lung cancer | 3 |
| Malignant skin melanoma | 3 |
| Non-melanoma skin cancer | 3 |
| Non-melanoma skin cancer (squamous-cell carcinoma) | 4 |
| Non-melanoma skin cancer (basal-cell carcinoma) | 4 |
| Soft tissue and other extraosseous sarcomas | 3 |
| Malignant neoplasm of bone and articular cartilage | 3 |
| Breast cancer | 3 |
| Cervical cancer | 3 |
| Uterine cancer | 3 |
| Ovarian cancer | 3 |
| Prostate cancer | 3 |
| Testicular cancer | 3 |
| Kidney cancer | 3 |
| Bladder cancer | 3 |
| Brain and central nervous system cancer | 3 |
| Eye cancer | 3 |
| Retinoblastoma | 4 |
| Other eye cancers | 4 |
| Neuroblastoma and other peripheral nervous cell tumors | 3 |
| Thyroid cancer | 3 |
| Mesothelioma | 3 |
| Hodgkin lymphoma | 3 |
| Non-Hodgkin lymphoma | 3 |
| Burkitt lymphoma | 4 |
| Other non-Hodgkin lymphoma | 4 |
| Multiple myeloma | 3 |
| Leukemia | 3 |
| Acute lymphoid leukemia | 4 |
| Chronic lymphoid leukemia | 4 |
| Acute myeloid leukemia | 4 |
| Chronic myeloid leukemia | 4 |
| Other leukemia | 4 |
| Other malignant neoplasms | 3 |
| Other neoplasms | 3 |
| Myelodysplastic, myeloproliferative, and other hematopoietic neoplasms | 4 |
| Benign and in situ intestinal neoplasms | 4 |
| Benign and in situ cervical and uterine neoplasms | 4 |
| Other benign and in situ neoplasms | 4 |
| Cardiovascular diseases | 2 |
| Rheumatic heart disease | 3 |
| Ischemic heart disease | 3 |
| Stroke | 3 |
| Ischemic stroke | 4 |
| Intracerebral hemorrhage | 4 |
| Subarachnoid hemorrhage | 4 |
| Hypertensive heart disease | 3 |
| Non-rheumatic valvular heart disease | 3 |
| Non-rheumatic calcific aortic valve disease | 4 |
| Non-rheumatic degenerative mitral valve disease | 4 |
| Other non-rheumatic valve diseases | 4 |
| Cardiomyopathy and myocarditis | 3 |
| Myocarditis | 4 |
| Alcoholic cardiomyopathy | 4 |
| Other cardiomyopathy | 4 |
| Pulmonary Arterial Hypertension | 3 |
| Atrial fibrillation and flutter | 3 |
| Aortic aneurysm | 3 |
| Lower extremity peripheral arterial disease | 3 |
| Endocarditis | 3 |
| Other cardiovascular and circulatory diseases | 3 |
| Chronic respiratory diseases | 2 |
| Chronic obstructive pulmonary disease | 3 |
| Pneumoconiosis | 3 |
| Silicosis | 4 |
| Asbestosis | 4 |
| Coal workers pneumoconiosis | 4 |
| Other pneumoconiosis | 4 |
| Asthma | 3 |
| Interstitial lung disease and pulmonary sarcoidosis | 3 |
| Other chronic respiratory diseases | 3 |
| Digestive diseases | 2 |
| Cirrhosis and other chronic liver diseases | 3 |
| Chronic hepatitis B including cirrhosis | 4 |
| Chronic hepatitis C including cirrhosis | 4 |
| Cirrhosis due to alcohol | 4 |
| Nonalcoholic fatty liver disease including cirrhosis | 4 |
| Cirrhosis due to other causes | 4 |
| Upper digestive system diseases | 3 |
| Peptic ulcer disease | 4 |
| Gastritis and duodenitis | 4 |
| Gastroesophageal reflux disease | 4 |
| Appendicitis | 3 |
| Paralytic ileus and intestinal obstruction | 3 |
| Inguinal, femoral, and abdominal hernia | 3 |
| Inflammatory bowel disease | 3 |
| Vascular intestinal disorders | 3 |
| Gallbladder and biliary diseases | 3 |
| Pancreatitis | 3 |
| Other digestive diseases | 3 |
| Neurological disorders | 2 |
| Alzheimer's disease and other dementias | 3 |
| Parkinson's disease | 3 |
| Idiopathic epilepsy | 3 |
| Multiple sclerosis | 3 |
| Motor neuron disease | 3 |
| Headache disorders | 3 |
| Migraine | 4 |
| Tension-type headache | 4 |
| Other neurological disorders | 3 |
| Mental disorders | 2 |
| Schizophrenia | 3 |
| Depressive disorders | 3 |
| Major depressive disorder | 4 |
| Dysthymia | 4 |
| Bipolar disorder | 3 |
| Anxiety disorders | 3 |
| Eating disorders | 3 |
| Anorexia nervosa | 4 |
| Bulimia nervosa | 4 |
| Autism spectrum disorders | 3 |
| Attention-deficit/hyperactivity disorder | 3 |
| Conduct disorder | 3 |
| Idiopathic developmental intellectual disability | 3 |
| Other mental disorders | 3 |
| Substance use disorders | 2 |
| Alcohol use disorders | 3 |
| Drug use disorders | 3 |
| Opioid use disorders | 4 |
| Cocaine use disorders | 4 |
| Amphetamine use disorders | 4 |
| Cannabis use disorders | 4 |
| Other drug use disorders | 4 |
| Diabetes and kidney diseases | 2 |
| Diabetes mellitus | 3 |
| Diabetes mellitus type 1 | 4 |
| Diabetes mellitus type 2 | 4 |
| Chronic kidney disease | 3 |
| Chronic kidney disease due to diabetes mellitus type 1 | 4 |
| Chronic kidney disease due to diabetes mellitus type 2 | 4 |
| Chronic kidney disease due to hypertension | 4 |
| Chronic kidney disease due to glomerulonephritis | 4 |
| Chronic kidney disease due to other and unspecified causes | 4 |
| Acute glomerulonephritis | 3 |
| Skin and subcutaneous diseases | 2 |
| Dermatitis | 3 |
| Atopic dermatitis | 4 |
| Contact dermatitis | 4 |
| Seborrhoeic dermatitis | 4 |
| Psoriasis | 3 |
| Bacterial skin diseases | 3 |
| Cellulitis | 4 |
| Pyoderma | 4 |
| Scabies | 3 |
| Fungal skin diseases | 3 |
| Viral skin diseases | 3 |
| Acne vulgaris | 3 |
| Alopecia areata | 3 |
| Pruritus | 3 |
| Urticaria | 3 |
| Decubitus ulcer | 3 |
| Other skin and subcutaneous diseases | 3 |
| Sense organ diseases | 2 |
| Blindness and vision loss | 3 |
| Glaucoma | 4 |
| Cataract | 4 |
| Age-related macular degeneration | 4 |
| Refraction disorders | 4 |
| Near vision loss | 4 |
| Other vision loss | 4 |
| Age-related and other hearing loss | 3 |
| Other sense organ diseases | 3 |
| Musculoskeletal disorders | 2 |
| Rheumatoid arthritis | 3 |
| Osteoarthritis | 3 |
| Osteoarthritis hip | 4 |
| Osteoarthritis knee | 4 |
| Osteoarthritis hand | 4 |
| Osteoarthritis other | 4 |
| Low back pain | 3 |
| Neck pain | 3 |
| Gout | 3 |
| Other musculoskeletal disorders | 3 |
| Other non-communicable diseases | 2 |
| Congenital birth defects | 3 |
| Neural tube defects | 4 |
| Congenital heart anomalies | 4 |
| Orofacial clefts | 4 |
| Down syndrome | 4 |
| Turner syndrome | 4 |
| Klinefelter syndrome | 4 |
| Other chromosomal abnormalities | 4 |
| Congenital musculoskeletal and limb anomalies | 4 |
| Urogenital congenital anomalies | 4 |
| Digestive congenital anomalies | 4 |
| Other congenital birth defects | 4 |
| Urinary diseases and male infertility | 3 |
| Urinary tract infections and interstitial nephritis | 4 |
| Urolithiasis | 4 |
| Benign prostatic hyperplasia | 4 |
| Male infertility | 4 |
| Other urinary diseases | 4 |
| Gynecological diseases | 3 |
| Uterine fibroids | 4 |
| Polycystic ovarian syndrome | 4 |
| Female infertility | 4 |
| Endometriosis | 4 |
| Genital prolapse | 4 |
| Premenstrual syndrome | 4 |
| Other gynecological diseases | 4 |
| Hemoglobinopathies and hemolytic anemias | 3 |
| Thalassemias | 4 |
| Thalassemias trait | 4 |
| Sickle cell disorders | 4 |
| Sickle cell trait | 4 |
| G6PD deficiency | 4 |
| G6PD trait | 4 |
| Other hemoglobinopathies and hemolytic anemias | 4 |
| Endocrine, metabolic, blood, and immune disorders | 3 |
| Oral disorders | 3 |
| Caries of deciduous teeth | 4 |
| Caries of permanent teeth | 4 |
| Periodontal diseases | 4 |
| Edentulism | 4 |
| Other oral disorders | 4 |
| Sudden infant death syndrome | 3 |
| Injuries | 1 |
| Transport injuries | 2 |
| Road injuries | 3 |
| Pedestrian road injuries | 4 |
| Cyclist road injuries | 4 |
| Motorcyclist road injuries | 4 |
| Motor vehicle road injuries | 4 |
| Other road injuries | 4 |
| Other transport injuries | 3 |
| Unintentional injuries | 2 |
| Falls | 3 |
| Drowning | 3 |
| Fire, heat, and hot substances | 3 |
| Poisonings | 3 |
| Poisoning by carbon monoxide | 4 |
| Poisoning by other means | 4 |
| Exposure to mechanical forces | 3 |
| Unintentional firearm injuries | 4 |
| Other exposure to mechanical forces | 4 |
| Adverse effects of medical treatment | 3 |
| Animal contact | 3 |
| Venomous animal contact | 4 |
| Non-venomous animal contact | 4 |
| Foreign body | 3 |
| Pulmonary aspiration and foreign body in airway | 4 |
| Foreign body in eyes | 4 |
| Foreign body in other body part | 4 |
| Environmental heat and cold exposure | 3 |
| Exposure to forces of nature | 3 |
| Other unintentional injuries | 3 |
| Self-harm and interpersonal violence | 2 |
| Self-harm | 3 |
| Self-harm by firearm | 4 |
| Self-harm by other specified means | 4 |
| Interpersonal violence | 3 |
| Physical violence by firearm | 4 |
| Physical violence by sharp object | 4 |
| Sexual violence | 4 |
| Physical violence by other means | 4 |
| Conflict and terrorism | 3 |
| Police conflict and executions | 3 |
| Other COVID-19 pandemic-related outcomes | 1 |
| Total cancers | 1 |
| Total burden related to hepatitis B | 1 |
| Total burden related to hepatitis C | 1 |
| Total burden related to Non-alcoholic fatty liver disease (NAFLD) | 1 |
| Total Cancers excluding Non-melanoma skin cancer | 1 |

Table S2: List of International Classification of Diseases (ICD-10) codes mapped to GBD causes

| **Cause** | **ICD10** |
| --- | --- |
| HIV/AIDS | B20-B24.9, F02.4 |
| Syphilis | A50-A53.9, I98.0, K67.2, M03.1, M73.1 |
| Chlamydial infection | A55-A56.8, K67.0 |
| Gonococcal infection | A54-A54.9, K67.1, M73.0 |
| Other sexually transmitted infections | A57-A58, A63-A63.8, B63 |
| Tuberculosis | A10-A14, A15-A19.9, B90-B90.9, K67.3, K93.0, M49.0, N74.1, P37.0, U84.3 |
| Lower respiratory infections | A48.1, A70, B34.2, B97.2, B97.4-B97.6, J09-J15.8, J16-J16.9, J20-J21.9, J91.0,  P23.0-P23.4, U04-U04.9 |
| Upper respiratory infections | J00-J02.8, J03-J03.8, J04-J04.2, J05-J05.1, J06.0-J06.8, J36-J36.0 |
| Otitis media | H70-H70.9 |
| COVID-19 | U07-U07.2 |
| Diarrheal diseases | A00-A00.9, A02-A02.0, A02.8-A07, A07.2-A07.4, A08-A09.9, K52.1-K52.3, R19.7 |
| Typhoid fever | A01.0 |
| Paratyphoid fever | A01.1-A01.4 |
| Invasive Non-typhoidal Salmonella (iNTS) | A02.1-A02.2 |
| Other intestinal infectious diseases | A07.0-A07.1, A07.8-A07.9, A80-A80.9 |
| Malaria | B50-B53.8 |
| Chagas disease | B57-B57.5, K93.1 |
| Leishmaniasis | B55.0 |
| African trypanosomiasis | B56-B56.9 |
| Schistosomiasis | B65-B65.9 |
| Cysticercosis | B69-B69.9 |
| Cystic echinococcosis | B67-B67.4, B67.8-B67.9 |
| Dengue | A90-A91.9 |
| Yellow fever | A95-A95.9 |
| Rabies | A82-A82.9 |
| Intestinal nematode infections | B77-B77.9 |
| Ebola | A98.4 |
| Zika virus | U06-U06.9 |
| Other neglected tropical diseases | A68-A68.9, A69.2-A69.9, A75-A75.9, A77-A79.9, A92-A94.0, A96-A96.9, A98-  A98.3, A98.5-A98.8, B33.0-B33.1, B60-B60.8, B67.5-B67.7, B70-B71.9, B74.3-B75,  B83-B83.8, P37.1 |
| Meningitis | A39-A39.9, A87-A87.9, G00.0-G00.8, G03-G03.8 |
| Encephalitis | A83-A86.4, B94.1, F07.1, G04-G05.8, G21.3 |
| Diphtheria | A36-A36.9 |
| Pertussis | A37-A37.9 |
| Tetanus | A33-A35.0 |
| Measles | B05-B05.9 |
| Ebola | A98.4 |
| **Cause** | **ICD10** |
| Zika virus | U06-U06.9 |
| Other neglected tropical diseases | A68-A68.9, A69.2-A69.9, A75-A75.9, A77-A79.9, A92-A94.0, A96-A96.9, A98-  A98.3, A98.5-A98.8, B33.0-B33.1, B60-B60.8, B67.5-B67.7, B70-B71.9, B74.3-B75,  B83-B83.8, P37.1 |
| Other infectious diseases | A20-A28.9, A32-A39.9, A48.2, A48.4-A48.5, A65-A65.0, A69-A69.1, A74, A74.8A74.9, A81-A81.9, A83-A89.9, B00-B06.9, B10-B10.8, B15-B16.2, B17.0, B17.2,  B19.1, B25-B27.9, B29.4, B33, B33.3-B33.8, B47-B48.8, B91, B94.1, B95-B95.5,  D70.3, D89.3, F02.1, F07.1, G00.0-G00.8, G03-G03.8, G04-G05.8, G14-G14.6,  G21.3, I00, I02, I02.9, I98.1, K67.8, K75.3, K76.3, K77.0, M49.1, M89.6, P35-P35.9, P37, P37.2, P37.5-P37.9, U82-U84, U85-U89, Z16-Z16.3 |
| Acute hepatitis A | B15-B15.9 |
| Acute hepatitis B | B16-B16.2, B17.0, B19.1, P35.3 |
| Acute hepatitis C | NA |
| Acute hepatitis E | B17.2 |
| Other unspecified infectious diseases | A20-A28.9, A32-A32.9, A38-A38.9, A48.2, A48.4-A48.5, A65-A65.0, A69-A69.1,  A74, A74.8-A74.9, A81-A81.9, A88-A89.9, B00-B00.9, B03-B04, B06-B06.9, B10-  B10.8, B25-B27.9, B29.4, B33, B33.3-B33.8, B47-B48.8, B91, B95-B95.5, D70.3,  D89.3, F02.1, G14-G14.6, I00, I02, I02.9, I98.1, K67.8, K75.3, K76.3, K77.0, M49.1, M89.6, P35-P35.2, P35.9, P37, P37.2, P37.5-P37.9, U82-U84, U85-U89, Z16-Z16.3 |
| Maternal hemorrhage | O20-O20.9, O43.2, O44-O46.9, O62-O62.9, O67-O67.9, O70, O72-O72.3 |
| Maternal sepsis and other maternal infections | O23-O23.9, O85-O86.8, O91-O91.2 |
| Maternal hypertensive disorders | O10-O16.9 |
| Maternal obstructed labor and uterine rupture | O32-O33.9, O64-O66.9, O71-O71.9 |
| Maternal abortion and miscarriage | N96, O01-O07.9 |
| Ectopic pregnancy | O00-O00.9 |
| Late maternal deaths | O96-O97.9 |
| Other direct maternal disorders | C58-C58.0, N98-N98.9, O09-O09.9, O21-O22.9, O26-O26.9, O28-O31.8, O34-  O36.9, O40-O43.1, O43.8-O43.9, O47-O48.1, O60-O61.9, O63-O63.9, O68-O69.9, O70.0-O70.9, O73-O77.9, O80-O84.9, O87-O90.9, O92-O92.7 |
| Neonatal preterm birth | P01.0-P01.1, P05-P05.9, P07-P07.3, P22-P22.9, P25-P28.9, P52-P52.9, P61.2, P77-  P77.9, P78.0-P78.9 |
| Neonatal encephalopathy due to birth asphyxia and trauma | P01.7, P02-P03.9, P10-P15.9, P20-P21.9, P24-P24.9, P90-P91.9 |
| Neonatal sepsis and other neonatal infections | P36-P36.9, P38-P39.9 |
| Hemolytic disease and other neonatal jaundice | P55-P59.9 |
| Other neonatal disorders | P00-P01, P01.2-P01.6, P01.8-P01.9, P04-P04.2, P04.5-P04.9, P08-P09, P19-P19.9,  P29-P29.9, P50-P51.9, P53-P54.9, P60-P61.1, P61.3-P61.9, P70-P70.1, P70.3-P72.9,  P74-P76.9, P78, P80-P81.9, P83-P84, P92-P92.9, P94-P94.9, P96, P96.3-P96.4, P96.8 |
| **Cause** | **ICD10** |
| Nutritional deficiencies | D50.1-D50.8, D51-D52.0, D52.8-D53.9, E00-E02, E40-E46.9, E51-E61.9, E63-E64.0,  E64.2-E64.9, M12.1 |
| Protein-energy malnutrition | E40-E46.9, E64.0 |
| Other nutritional deficiencies | D51-D52.0, D52.8-D53.9, E00-E02, E51-E61.9, E63-E64, E64.2-E64.9, M12.1 |
| Lip and oral cavity cancer | C00-C08.9, D10.0-D10.5, D11-D11.9 |
| Nasopharynx cancer | C11-C11.9, D10.6 |
| Other pharynx cancer | C09-C10.9, C12-C13.9, D10.7 |
| Esophageal cancer | C15-C15.9, D00.1, D13.0 |
| Stomach cancer | C16-C16.9, D00.2, D13.1, D37.1 |
| Colon and rectum cancer | C18-C21.9, D01.0-D01.3, D12-D12.9, D37.3-D37.5 |
| Liver cancer | C22-C22.8, D13.4 |
| Gallbladder and biliary tract cancer | C23-C24.9, D13.5 |
| Pancreatic cancer | C25-C25.9, D13.6-D13.7 |
| Larynx cancer | C32-C32.9, D02.0, D14.1, D38.0 |
| Tracheal, bronchus, and lung cancer | C33-C34.9, D02.1-D02.3, D14.2-D14.3, D38.1 |
| Malignant skin melanoma | C43-C43.9, D03-D03.9, D22-D23.9, D48.5 |
| Non-melanoma skin cancer | C44-C44.9, D04-D04.9, D49.2 |
| Soft tissue and other extraosseous sarcomas | C49-C49.9 |
| Malignant neoplasm of bone and articular cartilage | C40-C41.9 |
| Breast cancer | C50-C50.9, D05-D05.9, D24-D24.9, D48.6, D49.3 |
| Cervical cancer | C53-C53.9, D06-D06.9, D26.0 |
| Uterine cancer | C54-C54.9, D07.0-D07.2, D26.1-D26.9 |
| Ovarian cancer | C56-C56.9, D27-D27.9, D39.1 |
| Prostate cancer | C61-C61.9, D07.5, D29.1, D40.0 |
| Testicular cancer | C62-C62.9, D29.2-D29.8, D40.1-D40.8 |
| Kidney cancer | C64-C65.9, D30.0-D30.1, D41.0-D41.1 |
| Bladder cancer | C67-C67.9, D09.0, D30.3, D41.4-D41.8, D49.4 |
| Brain and central nervous system cancer | C70-C72.9, C75.1-C75.3 |
| Retinoblastoma | C69.2 |
| Other eye cancers | C69.0-C69.1, C69.3-C69.8 |
| Neuroblastoma and other peripheral nervous cell tumors | C47-C47.9 |
| Thyroid cancer | C73-C73.9, D09.3, D09.8, D34-D34.9, D44.0 |
| Mesothelioma | C45-C45.9 |
| Hodgkin lymphoma | C81-C81.9 |
| Non-Hodgkin lymphoma | C82-C82.9, C83.0-C83.8, C84-C85.0, C85.2-C85.8, C86-C86.6, C96-C96.9 |
| Multiple myeloma | C88-C90.9 |
| **Cause** | **ICD10** |
| Nutritional deficiencies | D50.1-D50.8, D51-D52.0, D52.8-D53.9, E00-E02, E40-E46.9, E51-E61.9, E63-E64.0,  E64.2-E64.9, M12.1 |
| Protein-energy malnutrition | E40-E46.9, E64.0 |
| Other nutritional deficiencies | D51-D52.0, D52.8-D53.9, E00-E02, E51-E61.9, E63-E64, E64.2-E64.9, M12.1 |
| Lip and oral cavity cancer | C00-C08.9, D10.0-D10.5, D11-D11.9 |
| Nasopharynx cancer | C11-C11.9, D10.6 |
| Other pharynx cancer | C09-C10.9, C12-C13.9, D10.7 |
| Esophageal cancer | C15-C15.9, D00.1, D13.0 |
| Stomach cancer | C16-C16.9, D00.2, D13.1, D37.1 |
| Colon and rectum cancer | C18-C21.9, D01.0-D01.3, D12-D12.9, D37.3-D37.5 |
| Liver cancer | C22-C22.8, D13.4 |
| Gallbladder and biliary tract cancer | C23-C24.9, D13.5 |
| Pancreatic cancer | C25-C25.9, D13.6-D13.7 |
| Larynx cancer | C32-C32.9, D02.0, D14.1, D38.0 |
| Tracheal, bronchus, and lung cancer | C33-C34.9, D02.1-D02.3, D14.2-D14.3, D38.1 |
| Malignant skin melanoma | C43-C43.9, D03-D03.9, D22-D23.9, D48.5 |
| Non-melanoma skin cancer | C44-C44.9, D04-D04.9, D49.2 |
| Soft tissue and other extraosseous sarcomas | C49-C49.9 |
| Malignant neoplasm of bone and articular cartilage | C40-C41.9 |
| Breast cancer | C50-C50.9, D05-D05.9, D24-D24.9, D48.6, D49.3 |
| Cervical cancer | C53-C53.9, D06-D06.9, D26.0 |
| Uterine cancer | C54-C54.9, D07.0-D07.2, D26.1-D26.9 |
| Ovarian cancer | C56-C56.9, D27-D27.9, D39.1 |
| Prostate cancer | C61-C61.9, D07.5, D29.1, D40.0 |
| Testicular cancer | C62-C62.9, D29.2-D29.8, D40.1-D40.8 |
| Kidney cancer | C64-C65.9, D30.0-D30.1, D41.0-D41.1 |
| Bladder cancer | C67-C67.9, D09.0, D30.3, D41.4-D41.8, D49.4 |
| Brain and central nervous system cancer | C70-C72.9, C75.1-C75.3 |
| Retinoblastoma | C69.2 |
| Other eye cancers | C69.0-C69.1, C69.3-C69.8 |
| Neuroblastoma and other peripheral nervous cell tumors | C47-C47.9 |
| Thyroid cancer | C73-C73.9, D09.3, D09.8, D34-D34.9, D44.0 |
| Mesothelioma | C45-C45.9 |
| Hodgkin lymphoma | C81-C81.9 |
| Non-Hodgkin lymphoma | C82-C82.9, C83.0-C83.8, C84-C85.0, C85.2-C85.8, C86-C86.6, C96-C96.9 |
| Multiple myeloma | C88-C90.9 |
| **Cause** | **ICD10** |
| Other musculoskeletal disorders | I27.1, I67.7, L93-L93.2, M00-M03.0, M03.2-M03.6, M07-M08, M08.9-M09.0, M09.2-  M09.8, M30-M32.9, M34-M36.8, M40-M43.1, M65-M65.0, M71.0-M71.1, M80-  M82.8, M86.3-M86.4, M87-M87.0, M88-M89.0, M89.5, M89.7-M89.9 |
| Neural tube defects | Q00-Q01.9, Q05-Q05.9 |
| Congenital heart anomalies | Q20-Q28.9 |
| Orofacial clefts | Q35-Q36, Q37-Q37.9 |
| Down syndrome | Q90-Q90.9 |
| Other chromosomal abnormalities | Q87-Q87.8, Q91-Q93.9, Q95-Q95.9, Q97-Q97.9, Q99-Q99.8 |
| Congenital musculoskeletal and limb anomalies | Q65-Q79, Q79.6-Q79.9 |
| Urogenital congenital anomalies | P96.0, Q50-Q60.6, Q63-Q64.9 |
| Digestive congenital anomalies | Q38-Q45.9, Q79.0-Q79.5 |
| Other congenital birth defects | G71.2, Q02-Q04.9, Q06-Q07.9, Q10.4-Q18.9, Q30-Q34.9, Q80-Q86, Q86.1-Q86.8,  Q89-Q89.8 |
| Urinary tract infections and interstitial nephritis | N10-N12.9, N13.6, N15, N15.1-N16.8, N30-N30.3, N30.8-N30.9, N34-N34.3, N39.0-  N39.2 |
| Urolithiasis | N20-N23.0 |
| Other urinary diseases | N25-N28.1, N29-N29.8, N31-N32.0, N32.3-N32.4, N36-N36.9, N39, N41-N41.9,  N44-N44.0, N45-N45.9, N49-N49.9 |
| Uterine fibroids | D25-D26, D28.2 |
| Endometriosis | N80-N80.9 |
| Genital prolapse | N81-N81.9 |
| Other gynecological diseases | N72-N72.0, N75-N77.8, N83-N83.9 |
| Thalassemias | D56-D56.9 |
| Sickle cell disorders | D57-D57.8 |
| G6PD deficiency | D55-D55.2 |
| Other hemoglobinopathies and hemolytic anemias | D55.3-D55.9, D58-D58.9, D59.1, D59.3, D59.5, D60-D61.9, D64.0 |
| Thyroid diseases | E03-E03.1, E03.3-E06.3, E06.5-E07, E07.1 |
| Other endocrine, metabolic, blood, and immune disorders | D66-D67, D68.0-D69.4, D69.6-D69.8, D70-D70.0, D70.4-D75.8, D76-D77, D86.8,  D89-D89.2, E07.0, E16.1-E16.9, E20-E23.0, E23.2-E24.1, E24.3, E24.8-E27.2, E27.4-  E28.1, E28.3-E34, E34.1-E34.8, E67-E68, E70-E77.9, E79-E83.9, E85-E85.2, E88-  E88.2, E88.4-E88.9 |
| Road injuries | V01-V04.9, V06-V80.9, V82-V82.9, V87.2-V87.3 |
| Falls | W00-W19.9 |
| Drowning | W65-W70.9, W73-W74.9 |
| Fire, heat, and hot substances | X00-X06.9, X08-X19.9 |
| Poisoning by carbon monoxide | X47-X47.9 |
| Poisoning by other means | X48-X48.9 |
| Unintentional firearm injuries | W32-W34.9 |
| Other exposure to mechanical forces | W20-W31.9, W35-W38.9, W40-W43.9, W45.0-W45.2, W46-W46.2, W49-W52 |
| **Cause** | **ICD10** |
| Adverse effects of medical treatment | D52.1, D59.0, D59.2, D59.6, D69.5, D70.1-D70.2, D78-D78.8, E03.2, E06.4, E09E09.9, E16.0, E23.1, E24.2, E27.3, E36-E36.8, E66.1, E88.3, E89-E89.9, G21.0G21.1, G24.0, G25.1, G25.4, G25.6-G25.7, G72.0, G93.7, G97-G97.9, I95.2-I95.3,  I97-I97.9, I98.9, J70.0-J70.5, J95-J95.9, K43-K43.9, K52.0, K62.7, K91-K91.9, K94-  K95.8, M87.1, N14-N14.4, N30.4, N65-N65.1, N99-N99.9, P93-P93.8, P96.2, P96.5,  R50.2, Y40-Y84.9, Y88-Y88.3 |
| Venomous animal contact | X20-X29.9 |
| Non-venomous animal contact | W52.0-W62.9, W64-W64.9 |
| Pulmonary aspiration and foreign body in airway | W75-W75.9, W78-W80.9, W83-W84.9 |
| Foreign body in other body part | W44-W45, W45.3-W45.9 |
| Electrocution | W85-W87.9 |
| Environmental heat and cold exposure | L55-L55.9, L56.3, L56.8-L56.9, L58-L58.9, W88-W94.9, W97.9, W99-W99.9, X30-  X32.9, X39-X39.9 |
| Exposure to forces of nature | X33-X38.9 |
| Other unintentional injuries (internal) | W39-W39.9, W77-W77.9, W81-W81.9, X50-X54.9, X57-X58.9 |
| Self-harm by firearm | X72-X74.9 |
| Self-harm by other specified means | X60-X64.9, X66-X71.9, X75-X83.9, Y87.0 |
| Physical violence by firearm | X93-X95.9 |
| Physical violence by sharp object | X99-X99.9 |
| Physical violence by other means | X85-X92.9, X96-X98.9, Y00-Y04.9, Y06-Y08.9, Y87.1 |
| Conflict and terrorism | U00-U03, Y36-Y38.9, Y89.1 |
| Police conflict and executions | Y35-Y35.9, Y89.0 |

Table S3: ICD-10 codes by detailed garbage package

| **Table S3: ICD-10 codes by detailed garbage package** | |  |
| --- | --- | --- |
| **Detailed Garbage Package** | **ICD-10 Codes** | **Redistribution Package** |
| Abdomen and Pelvis Cancer | C762, C763, C772, C775, C784, C785, C786, C787, C788, C790, C791 | Negative correlation |
| Acute Respiratory Failure | J80, J800, J809, J810, J96, J960, J969, J981, J982, J983 | MCoD |
| Acute kidney failure | N17, N170, N171, N172, N178, N179, N19, N190, N199 | MCoD |
| Adrenal Site Cancer unspecified part of adrenal gland | C749 | Proportional |
| Adrenal Unspecified Site Cancer in medulla or cortex | C741 | Proportional |
| Adrenal Unspecified Site Cancer-parent cause | C74, C740 | Proportional |
| Alcoholic hepatic failure | K704, K709 | MCoD |
| All, Ill Defined code for causes of death | A59, A590, A598, A599, A71, A710, A711, A719, A740, B07, B070, B078, B079, B30, B300, B301, B302, B303, B308, B309, B35, B350, B351, B352, B353, B354, B355, B356, B358, B359, B36, B360, B361, B362, B363, B368, B369, B85, B850, B851, B852, B853, B854, B87, B870, B871, B872, B873, B874, B878, B879, B88, B880, B881, B882, B883, B888, B889, B940, D68, E15, E150, E16, E50, E500, E501, E502, E503, E504, E505, E506, E507, E508, E509, E641, G32, G320, G328, G43, G430, G431, G432, G433, G434, G435, G436, G437, G438, G439, G44, G440, G441, G442, G444, G445, G448, G47, G470, G471, G472, G474, G475, G476, G478, G479, G50, G500, G501, G508, G509, G51, G510, G511, G512, G513, G514, G518, G519, G52, G520, G521, G522, G523, G527, G528, G529, G53, G530, G531, G532, G533, G538, G54, G540, G541, G542, G543, G544, G545, G546, G547, G548, G549, G55, G550, G551, G552, G553, G558, G56, G560, G561, G562, G563, G564, G568, G569, G57, G570, G571, G572, G573, G574, G575, G576, G577, G578, G579, G58, G580, G587, G588, G589, G59, G590, G598, G60, G600, G601, G602, G603, G608, G609, G62, G620, G622, G628, G629, G63, G630, G631, G632, G633, G634, G635, G636, G638, G64, G640, G65, G650, G651, G652, G89, G890, G891, G892, G893, G894, G99, G990, G991, G992, G998, H00, H000, H001, H01, H010, H011, H018, H019, H02, H020, H021, H022, H023, H024, H025, H026, H027, H028, H029, H03, H030, H031, H038, H04, H040, H041, H042, H043, H044, H045, H046, H048, H049, H05, H052, H053, H054, H055, H058, H059, H06, H060, H061, H062, H063, H07, H08, H09, H10, H100, H101, H102, H103, H104, H105, H108, H109, H11, H110, H111, H112, H113, H114, H118, H119, H12, H13, H130, H131, H132, H133, H138, H14, H15, H150, H151, H158, H159, H16, H160, H161, H162, H163, H164, H168, H169, H17, H170, H171, H178, H179, H18, H180, H181, H182, H183, H184, H185, H186, H187, H188, H189, H19, H190, H191, H192, H193, H198, H20, H200, H201, H202, H208, H209, H21, H210, H211, H212, H213, H214, H215, H218, H219, H22, H220, H221, H228, H23, H24, H25, H250, H251, H252, H258, H259, H26, H260, H261, H262, H263, H264, H268, H269, H27, H270, H271, H278, H279, H28, H280, H281, H282, H288, H29, H30, H300, H301, H302, H308, H309, H31, H310, H311, H312, H313, H314, H318, H319, H32, H320, H328, H33, H330, H331, H332, H333, H334, H335, H338, H34, H340, H341, H342, H348, H349, H35, H350, H351, H352, H353, H354, H355, H356, H357, H358, H359, H36, H360, H368, H37, H38, H39, H40, H400, H401, H402, H403, H404, H405, H406, H408, H409, H41, H42, H420, H428, H43, H430, H431, H432, H433, H438, H439, H44, H440, H441, H442, H443, H444, H445, H446, H447, H448, H449, H45, H450, H451, H458, H46, H460, H461, H462, H463, H468, H469, H47, H470, H471, H472, H473, H474, H475, H476, H477, H479, H48, H480, H481, H488, H49, H490, H491, H492, H493, H494, H498, H499, H50, H500, H501, H502, H503, H504, H505, H506, H508, H509, H51, H510, H511, H512, H518, H519, H52, H520, H521, H522, H523, H524, H525, H526, H527, H53, H530, H531, H532, H533, H534, H535, H536, H537, H538, H539, H54, H540, H541, H542, H543, H544, H545, H546, H547, H548, H549, H55, H550, H558, H56, H57, H570, H571, H578, H579, H58, H580, H588, H589, H59, H590, H591, H592, H593, H594, H598, H60, H600, H601, H602, H603, H604, H605, H606, H608, H609, H61, H610, H611, H612, H613, H618, H619, H62, H620, H621, H622, H623, H624, H628, H65, H650, H651, H652, H653, H654, H659, H66, H660, H661, H662, H663, H664, H669, H67, H670, H671, H672, H673, H678, H679, H68, H680, H681, H69, H690, H698, H699, H71, H710, H711, H712, H713, H719, H72, H720, H721, H722, H728, H729, H73, H730, H731, H732, H738, H739, H74, H740, H741, H742, H743, H744, H748, H749, H75, H750, H758, H76, H77, H78, H79, H80, H800, H801, H802, H808, H809, H81, H810, H811, H812, H813, H814, H818, H819, H82, H821, H822, H823, H829, H83, H830, H831, H832, H833, H838, H839, H84, H85, H86, H87, H876, H88, H89, H90, H900, H901, H902, H903, H904, H905, H906, H907, H908, H91, H910, H911, H912, H913, H918, H919, H92, H920, H921, H922, H93, H930, H931, H932, H933, H938, H939, H94, H940, H948, H95, H950, H951, H952, H953, H954, H958, H959, H96, H97, H98, H99, K00, K000, K001, K002, K003, K004, K005, K006, K007, K008, K009, K01, K010, K011, K02, K020, K021, K022, K023, K024, K025, K026, K027, K028, K029, K03, K030, K031, K032, K033, K034, K035, K036, K037, K038, K039, K04, K040, K041, K042, K043, K044, K045, K046, K047, K048, K049, K05, K050, K051, K052, K053, K054, K055, K056, K06, K060, K061, K062, K068, K069, K07, K070, K071, K072, K073, K074, K075, K076, K078, K079, K08, K080, K081, K082, K083, K084, K085, K088, K089, K09, K090, K091, K092, K098, K099, K10, K100, K101, K102, K103, K108, K109, K11, K110, K111, K112, K113, K114, K115, K116, K117, K118, K119, K12, K120, K121, K122, K123, K13, K130, K131, K132, K133, K134, K135, K136, K137, K14, K140, K141, K142, K143, K144, K145, K146, K148, K149, K159, K16, K17, K18, K19, K30, L20, L200, L208, L209, L21, L210, L211, L218, L219, L22, L23, L230, L231, L232, L233, L234, L235, L236, L237, L238, L239, L24, L240, L241, L242, L243, L244, L245, L246, L247, L248, L249, L25, L250, L251, L252, L253, L254, L255, L258, L259, L26, L269, L27, L270, L271, L272, L278, L279, L28, L280, L281, L282, L29, L290, L291, L292, L293, L298, L299, L30, L300, L301, L302, L303, L304, L305, L308, L309, L40, L400, L401, L402, L403, L404, L405, L408, L409, L41, L410, L411, L412, L413, L414, L415, L418, L419, L42, L43, L430, L431, L432, L433, L438, L439, L44, L440, L441, L442, L443, L444, L448, L449, L45, L46, L47, L48, L49, L490, L491, L492, L493, L494, L495, L496, L497, L498, L499, L50, L500, L501, L502, L503, L504, L505, L506, L508, L509, L52, L53, L530, L531, L532, L533, L538, L539, L54, L540, L56, L560, L561, L562, L564, L565, L57, L570, L571, L572, L573, L574, L575, L578, L579, L59, L590, L598, L599, L60, L600, L601, L602, L603, L604, L605, L608, L609, L61, L62, L620, L628, L63, L630, L631, L632, L638, L639, L64, L640, L648, L649, L65, L650, L651, L652, L658, L659, L66, L660, L661, L662, L663, L664, L668, L669, L67, L670, L671, L678, L679, L68, L680, L681, L682, L683, L688, L689, L70, L700, L701, L702, L703, L704, L705, L708, L709, L71, L710, L711, L718, L719, L72, L720, L721, L722, L723, L728, L729, L73, L730, L731, L732, L738, L739, L74, L740, L741, L742, L743, L744, L745, L748, L749, L75, L750, L751, L752, L758, L759, L76, L760, L761, L762, L768, L80, L81, L810, L811, L812, L813, L814, L815, L816, L817, L818, L819, L82, L820, L821, L83, L84, L85, L850, L851, L852, L853, L858, L859, L86, L87, L870, L871, L872, L878, L879, L90, L900, L901, L902, L903, L904, L905, L906, L908, L909, L91, L910, L918, L919, L92, L920, L921, L922, L923, L928, L929, L94, L940, L941, L942, L943, L944, L945, L946, L948, L949, L95, L950, L951, L958, L959, L96, L985, L986, L988, L989, L99, L990, L998, M04, M10, M100, M101, M102, M103, M104, M109, M11, M110, M111, M112, M118, M119, M12, M120, M122, M123, M124, M125, M128, M129, M13, M130, M131, M138, M139, M14, M140, M141, M142, M143, M144, M145, M146, M148, M15, M150, M151, M152, M153, M154, M158, M159, M16, M160, M161, M162, M163, M164, M165, M166, M167, M169, M17, M170, M171, M172, M173, M174, M175, M179, M18, M180, M181, M182, M183, M184, M185, M189, M19, M190, M191, M192, M198, M199, M20, M200, M201, M202, M203, M204, M205, M206, M21, M210, M211, M212, M213, M214, M215, M216, M217, M218, M219, M22, M220, M221, M222, M223, M224, M228, M229, M23, M230, M231, M232, M233, M234, M235, M236, M238, M239, M24, M240, M241, M242, M243, M244, M245, M246, M247, M248, M249, M25, M250, M251, M252, M253, M254, M255, M256, M257, M258, M259, M26, M260, M261, M262, M263, M264, M265, M266, M267, M268, M269, M27, M270, M271, M272, M273, M274, M275, M276, M278, M279, M28, M29, M37, M38, M39, M432, M433, M434, M435, M436, M438, M439, M44, M45, M450, M451, M452, M453, M454, M455, M456, M457, M458, M459, M46, M460, M461, M462, M463, M464, M465, M468, M469, M47, M470, M471, M472, M478, M479, M48, M480, M481, M482, M483, M484, M485, M488, M489, M49, M492, M493, M494, M495, M498, M50, M500, M501, M502, M503, M508, M509, M51, M510, M511, M512, M513, M514, M518, M519, M52, M53, M530, M531, M532, M533, M538, M539, M54, M540, M541, M542, M543, M544, M545, M546, M548, M549, M55, M56, M57, M58, M59, M60, M600, M601, M602, M608, M609, M61, M610, M611, M612, M613, M614, M615, M619, M62, M620, M621, M622, M623, M624, M625, M626, M628, M629, M63, M630, M631, M632, M633, M638, M64, M651, M652, M653, M654, M658, M659, M66, M660, M661, M662, M663, M664, M665, M668, M669, M67, M670, M671, M672, M673, M674, M675, M678, M679, M68, M680, M688, M69, M70, M700, M701, M702, M703, M704, M705, M706, M707, M708, M709, M71, M712, M713, M714, M715, M718, M719, M72, M720, M721, M722, M723, M724, M728, M729, M73, M738, M74, M75, M750, M751, M752, M753, M754, M755, M758, M759, M76, M760, M761, M762, M763, M764, M765, M766, M767, M768, M769, M77, M770, M771, M772, M773, M774, M775, M778, M779, M78, M79, M790, M791, M792, M793, M794, M795, M796, M797, M798, M799, M83, M830, M831, M832, M833, M834, M835, M838, M839, M84, M840, M841, M842, M843, M844, M845, M846, M848, M849, M85, M850, M851, M852, M853, M854, M855, M856, M858, M859, M872, M873, M878, M879, M891, M892, M893, M894, M90, M900, M901, M902, M903, M904, M905, M906, M907, M908, M91, M910, M911, M912, M913, M914, M918, M919, M92, M920, M921, M922, M923, M924, M925, M926, M927, M928, M929, M93, M930, M931, M932, M938, M939, M94, M940, M941, M942, M943, M948, M949, M95, M950, M951, M952, M953, M954, M955, M958, M959, M96, M960, M961, M962, M963, M964, M965, M966, M968, M969, M97, M98, M99, M990, M991, M992, M993, M994, M995, M996, M997, M998, M999, N328, N329, N33, N330, N338, N35, N350, N351, N358, N359, N37, N370, N378, N393, N394, N398, N42, N420, N421, N422, N423, N428, N429, N43, N430, N431, N432, N433, N434, N441, N442, N448, N46, N460, N461, N468, N469, N47, N470, N471, N472, N473, N474, N475, N476, N477, N478, N48, N480, N481, N482, N483, N484, N485, N486, N488, N489, N50, N500, N501, N503, N508, N509, N51, N510, N511, N512, N518, N52, N520, N521, N522, N523, N528, N529, N53, N531, N538, N539, N61, N610, N619, N62, N63, N630, N64, N640, N641, N642, N643, N644, N645, N648, N649, N91, N910, N911, N912, N913, N914, N915, N95, N951, N952, N953, N958, N959, N97, N970, N971, N972, N973, N974, N978, N979, R070, R08, R09, R093, R12, R120, R14, R140, R141, R142, R143, R15, R150, R151, R152, R159, R19, R190, R191, R192, R193, R194, R195, R196, R198, R20, R200, R201, R202, R203, R208, R209, R21, R210, R22, R220, R221, R222, R223, R224, R227, R229, R23, R231, R232, R233, R234, R238, R239, R240, R25, R250, R251, R252, R253, R254, R258, R259, R26, R260, R261, R262, R263, R268, R269, R27, R270, R278, R279, R28, R29, R290, R291, R292, R293, R294, R295, R296, R298, R299, R30, R300, R301, R309, R32, R33, R330, R338, R339, R34, R340, R349, R35, R350, R351, R358, R36, R360, R361, R369, R37, R38, R39, R390, R391, R392, R398, R399, R41, R410, R411, R412, R413, R414, R418, R419, R42, R420, R43, R430, R431, R432, R438, R439, R44, R440, R441, R442, R443, R448, R449, R45, R450, R451, R452, R453, R454, R455, R456, R457, R458, R46, R460, R461, R462, R463, R464, R465, R466, R467, R468, R47, R470, R471, R478, R479, R48, R480, R481, R482, R483, R488, R489, R49, R490, R491, R492, R498, R499, R51, R510, R52, R520, R521, R522, R529, R53, R530, R531, R532, R538, R550, R580, R588, R589, R59, R590, R591, R599, R60, R600, R601, R609, R61, R610, R611, R619, R62, R620, R625, R627, R628, R629, R63, R630, R631, R632, R633, R635, R638, R640, R649, R65, R651, R66, R67, R68, R680, R681, R682, R683, R688, R69, R690, R699, R70, R700, R701, R71, R710, R718, R72, R720, R729, R74, R740, R746, R748, R749, R75, R750, R759, R76, R760, R761, R762, R768, R769, R77, R770, R771, R772, R778, R779, R78, R786, R787, R788, R789, R79, R790, R791, R798, R799, R80, R800, R801, R802, R803, R808, R809, R81, R810, R82, R820, R821, R822, R823, R824, R825, R826, R827, R828, R829, R83, R830, R831, R832, R833, R834, R835, R836, R837, R838, R839, R84, R840, R841, R842, R843, R844, R845, R846, R847, R848, R849, R85, R850, R851, R852, R853, R854, R855, R856, R857, R858, R859, R86, R860, R861, R862, R863, R864, R865, R866, R867, R868, R869, R87, R870, R871, R872, R873, R874, R875, R876, R877, R878, R879, R88, R880, R888, R89, R890, R891, R892, R893, R894, R895, R896, R897, R898, R899, R90, R900, R908, R91, R910, R911, R918, R92, R920, R921, R922, R928, R93, R930, R931, R932, R933, R934, R935, R936, R937, R938, R939, R94, R940, R941, R942, R943, R944, R945, R946, R947, R948, R960, R961, R97, R970, R971, R972, R978, R99, U08, U09, U10, U11, U12, U13, U14, U15, U16, U17, U18, U19, U20, U21, U22, U23, U24, U25, U26, U27, U28, U29, U30, U31, U32, U33, U34, U35, U36, U37, U38, U39, U40, U41, U42, U43, U44, U45, U46, U47, U48, U49, U51, U52, U53, U54, U55, U56, U57, U58, U59, U60, U61, U62, U63, U64, U65, U66, U67, U68, U69, U70, U71, U72, U73, U74, U75, U76, U77, U78, U79, U80, U81, U90, U91, U92, U93, U94, U95, U96, U97, U98, U99, Z00, Z000, Z001, Z002, Z003, Z004, Z005, Z006, Z007, Z008, Z01, Z010, Z011, Z012, Z013, Z014, Z015, Z016, Z017, Z018, Z019, Z02, Z020, Z021, Z022, Z023, Z024, Z025, Z026, Z027, Z028, Z029, Z03, Z030, Z031, Z032, Z033, Z034, Z035, Z036, Z037, Z038, Z039, Z04, Z040, Z041, Z042, Z043, Z044, Z045, Z046, Z047, Z048, Z049, Z08, Z080, Z081, Z082, Z087, Z088, Z089, Z09, Z090, Z091, Z092, Z093, Z094, Z097, Z098, Z099, Z10, Z100, Z101, Z102, Z103, Z108, Z11, Z110, Z111, Z112, Z113, Z114, Z115, Z116, Z118, Z119, Z12, Z120, Z121, Z122, Z123, Z124, Z125, Z126, Z127, Z128, Z129, Z13, Z130, Z131, Z132, Z133, Z134, Z135, Z136, Z137, Z138, Z139, Z14, Z140, Z141, Z148, Z15, Z150, Z158, Z17, Z170, Z171, Z18, Z180, Z181, Z182, Z183, Z188, Z189, Z19, Z20, Z200, Z201, Z202, Z203, Z204, Z205, Z206, Z207, Z208, Z209, Z21, Z210, Z22, Z220, Z221, Z222, Z223, Z224, Z225, Z226, Z228, Z229, Z23, Z230, Z231, Z232, Z233, Z234, Z235, Z236, Z237, Z238, Z24, Z240, Z241, Z242, Z243, Z244, Z245, Z246, Z25, Z250, Z251, Z258, Z26, Z260, Z268, Z269, Z27, Z270, Z271, Z272, Z273, Z274, Z278, Z279, Z28, Z280, Z281, Z282, Z283, Z288, Z289, Z29, Z290, Z291, Z292, Z298, Z299, Z30, Z300, Z301, Z302, Z303, Z304, Z305, Z308, Z309, Z31, Z310, Z311, Z312, Z313, Z314, Z315, Z316, Z318, Z319, Z32, Z320, Z321, Z322, Z323, Z33, Z331, Z332, Z34, Z340, Z348, Z349, Z35, Z350, Z351, Z352, Z353, Z354, Z355, Z356, Z357, Z358, Z359, Z36, Z360, Z361, Z362, Z363, Z364, Z365, Z368, Z369, Z37, Z370, Z371, Z372, Z373, Z374, Z375, Z376, Z377, Z379, Z38, Z380, Z381, Z382, Z383, Z384, Z385, Z386, Z387, Z388, Z39, Z390, Z391, Z392, Z40, Z400, Z408, Z409, Z41, Z410, Z411, Z412, Z413, Z418, Z419, Z42, Z420, Z421, Z422, Z423, Z424, Z428, Z429, Z43, Z430, Z431, Z432, Z433, Z434, Z435, Z436, Z437, Z438, Z439, Z44, Z440, Z441, Z442, Z443, Z448, Z449, Z45, Z450, Z451, Z452, Z453, Z454, Z458, Z459, Z46, Z460, Z461, Z462, Z463, Z464, Z465, Z466, Z467, Z468, Z469, Z47, Z470, Z471, Z472, Z473, Z478, Z479, Z48, Z480, Z481, Z482, Z483, Z488, Z489, Z49, Z490, Z491, Z492, Z493, Z50, Z500, Z501, Z502, Z503, Z504, Z505, Z506, Z507, Z508, Z509, Z51, Z510, Z511, Z512, Z513, Z514, Z515, Z516, Z518, Z519, Z52, Z520, Z521, Z522, Z523, Z524, Z525, Z526, Z527, Z528, Z529, Z53, Z530, Z531, Z532, Z538, Z539, Z54, Z540, Z541, Z542, Z543, Z544, Z547, Z548, Z549, Z55, Z550, Z551, Z552, Z553, Z554, Z558, Z559, Z56, Z560, Z561, Z562, Z563, Z564, Z565, Z566, Z567, Z568, Z569, Z57, Z570, Z571, Z572, Z573, Z574, Z575, Z576, Z577, Z578, Z579, Z58, Z580, Z581, Z582, Z583, Z584, Z585, Z586, Z587, Z588, Z589, Z59, Z590, Z591, Z592, Z593, Z594, Z595, Z596, Z597, Z598, Z599, Z60, Z600, Z601, Z602, Z603, Z604, Z605, Z608, Z609, Z61, Z610, Z611, Z612, Z613, Z614, Z615, Z616, Z617, Z618, Z619, Z62, Z620, Z621, Z622, Z623, Z624, Z625, Z626, Z628, Z629, Z63, Z630, Z631, Z632, Z633, Z634, Z635, Z636, Z637, Z638, Z639, Z64, Z640, Z641, Z642, Z643, Z644, Z65, Z650, Z651, Z652, Z653, Z654, Z655, Z658, Z659, Z66, Z67, Z671, Z672, Z673, Z674, Z679, Z68, Z681, Z682, Z683, Z684, Z685, Z69, Z690, Z691, Z698, Z70, Z700, Z701, Z702, Z703, Z708, Z709, Z71, Z710, Z711, Z712, Z713, Z714, Z715, Z716, Z717, Z718, Z719, Z72, Z720, Z721, Z722, Z723, Z724, Z725, Z726, Z728, Z729, Z73, Z730, Z731, Z732, Z733, Z734, Z735, Z736, Z738, Z739, Z74, Z740, Z741, Z742, Z743, Z748, Z749, Z75, Z750, Z751, Z752, Z753, Z754, Z755, Z758, Z759, Z76, Z760, Z761, Z762, Z763, Z764, Z765, Z768, Z769, Z77, Z770, Z771, Z772, Z779, Z78, Z780, Z781, Z789, Z79, Z790, Z791, Z792, Z793, Z794, Z795, Z798, Z80, Z800, Z801, Z802, Z803, Z804, Z805, Z806, Z807, Z808, Z809, Z81, Z810, Z811, Z812, Z813, Z814, Z818, Z82, Z820, Z821, Z822, Z823, Z824, Z825, Z826, Z827, Z828, Z83, Z830, Z831, Z832, Z833, Z834, Z835, Z836, Z837, Z84, Z840, Z841, Z842, Z843, Z848, Z85, Z850, Z851, Z852, Z853, Z854, Z855, Z856, Z857, Z858, Z859, Z86, Z860, Z861, Z862, Z863, Z864, Z865, Z866, Z867, Z87, Z870, Z871, Z872, Z873, Z874, Z875, Z876, Z877, Z878, Z88, Z880, Z881, Z882, Z883, Z884, Z885, Z886, Z887, Z888, Z889, Z89, Z890, Z891, Z892, Z893, Z894, Z895, Z896, Z897, Z898, Z899, Z90, Z900, Z901, Z902, Z903, Z904, Z905, Z906, Z907, Z908, Z91, Z910, Z911, Z912, Z913, Z914, Z915, Z916, Z918, Z92, Z920, Z921, Z922, Z923, Z924, Z925, Z926, Z928, Z929, Z93, Z930, Z931, Z932, Z933, Z934, Z935, Z936, Z938, Z939, Z94, Z940, Z941, Z942, Z943, Z944, Z945, Z946, Z947, Z948, Z949, Z95, Z950, Z951, Z952, Z953, Z954, Z955, Z958, Z959, Z96, Z960, Z961, Z962, Z963, Z964, Z965, Z966, Z967, Z968, Z969, Z97, Z970, Z971, Z972, Z973, Z974, Z975, Z978, Z98, Z980, Z981, Z982, Z983, Z984, Z985, Z986, Z988, Z99, Z990, Z991, Z992, Z993, Z998, Z999, R24, U800, H581, L548, R650, H64, I91, I911, R9 | Proportional |
| Amyloidosis | E853, E854, E855, E856, E857, E858, E859 | MCoD |
| Anemia Unspecified | D50, D500, D509, D62, D620, D629, D63, D638, D64, D641, D642, D643, D644, D648, D649, D699 | Impairments |
| Anemia in neoplastic Diseases | D630 | Proportional |
| Arterial Embolism | I74, I740, I741, I742, I743, I744, I745, I748, I749, I75, I750, I758 | MCoD |
| Assault by unspecified means | Y09, Y090, Y091, Y092, Y093, Y094, Y095, Y096, Y097, Y098, Y099 | Proportional |
| Assigned death to tobacco | F17, F170, F171, F172, F173, F174, F175, F176, F177, F178, F179 | Proportional |
| Atherosclerosis | I70, I700, I701, I708, I709 | MCoD |
| CKD due to diabetes Unspecified type | E122, E132, E142 | Negative correlation |
| CNS Abscess | G06, G060, G061, G062, G07, G070, G08, G080 | Proportional |
| CNS Fluid Diseases | E877 | Proportional |
| Cachexia | R634, R636, R64 | MCoD |
| Cardiac rhythm disorders | I44, I440, I441, I442, I443, I444, I445, I446, I447, I448, I449, I45, I450, I451, I452, I453, I454, I455, I456, I458, I459, I49, I490, I491, I492, I493, I494, I495, I498, I499 | MCoD |
| Cerebral Cysts | G930 | Proportional |
| Cerebral Palsy | G80, G800, G801, G802, G803, G804, G808, G809, G821, G824, G830, G838 | MCoD |
| Chronic lymphocytic leukemia by age | C911 | Proportional |
| Chronic respiratory failure | J961, J962, J964, J965, J968 | MCoD |
| Diabetes unspecified type | E12, E120, E121, E123, E124, E125, E126, E127, E128, E129, E13, E130, E131, E133, E134, E135, E136, E137, E138, E139, E14, E140, E141, E143, E144, E145, E146, E147, E148, E149, R73, R730, R739 | Negative correlation |
| Exposure to unspecified factor X59 | W48, W63, W71, W72, W82, W96, W97, W98, X07, X56, X59, X590, X591, X592, X593, X594, X595, X596, X597, X598, X599, W95, X55 | MCoD |
| External Causes UDI, type unspecified | F072, G443, G913, R58, S00, S000, S001, S002, S003, S004, S005, S007, S008, S009, S01, S010, S011, S012, S013, S014, S015, S017, S018, S019, S02, S020, S021, S022, S023, S024, S025, S026, S027, S028, S029, S03, S030, S031, S032, S033, S034, S035, S038, S039, S04, S040, S041, S042, S043, S044, S045, S046, S047, S048, S049, S05, S050, S051, S052, S053, S054, S055, S056, S057, S058, S059, S06, S060, S061, S062, S063, S064, S065, S066, S067, S068, S069, S07, S070, S071, S078, S079, S08, S080, S081, S088, S089, S09, S090, S091, S092, S093, S097, S098, S099, S10, S100, S101, S107, S108, S109, S11, S110, S111, S112, S117, S118, S119, S12, S120, S121, S122, S123, S124, S125, S126, S127, S128, S129, S13, S130, S131, S132, S133, S134, S135, S136, S138, S139, S14, S140, S141, S142, S143, S144, S145, S146, S148, S149, S15, S150, S151, S152, S153, S157, S158, S159, S16, S161, S162, S168, S169, S17, S170, S178, S179, S18, S19, S197, S198, S199, S20, S200, S201, S202, S203, S204, S207, S208, S209, S21, S210, S211, S212, S213, S214, S217, S218, S219, S22, S220, S221, S222, S223, S224, S225, S228, S229, S23, S230, S231, S232, S233, S234, S235, S238, S239, S24, S240, S241, S242, S243, S244, S245, S246, S248, S249, S25, S250, S251, S252, S253, S254, S255, S257, S258, S259, S26, S260, S261, S268, S269, S27, S270, S271, S272, S273, S274, S275, S276, S277, S278, S279, S28, S280, S281, S282, S29, S290, S297, S298, S299, S30, S300, S301, S302, S303, S307, S308, S309, S31, S310, S311, S312, S313, S314, S315, S317, S318, S32, S320, S321, S322, S323, S324, S325, S327, S328, S329, S33, S330, S331, S332, S333, S334, S335, S336, S337, S338, S339, S34, S340, S341, S342, S343, S344, S345, S346, S348, S349, S35, S350, S351, S352, S353, S354, S355, S357, S358, S359, S36, S360, S361, S362, S363, S364, S365, S366, S367, S368, S369, S37, S370, S371, S372, S373, S374, S375, S376, S377, S378, S379, S38, S380, S381, S382, S383, S39, S390, S396, S397, S398, S399, S40, S400, S402, S407, S408, S409, S41, S410, S411, S417, S418, S42, S420, S421, S422, S423, S424, S427, S428, S429, S43, S430, S431, S432, S433, S434, S435, S436, S437, S438, S439, S44, S440, S441, S442, S443, S444, S445, S447, S448, S449, S45, S450, S451, S452, S453, S457, S458, S459, S46, S460, S461, S462, S463, S467, S468, S469, S47, S471, S472, S479, S48, S480, S481, S489, S49, S490, S491, S497, S498, S499, S50, S500, S501, S503, S507, S508, S509, S51, S510, S517, S518, S519, S52, S520, S521, S522, S523, S524, S525, S526, S527, S528, S529, S53, S530, S531, S532, S533, S534, S537, S54, S540, S541, S542, S543, S547, S548, S549, S55, S550, S551, S552, S557, S558, S559, S56, S560, S561, S562, S563, S564, S565, S567, S568, S569, S57, S570, S578, S579, S58, S580, S581, S589, S59, S590, S591, S592, S597, S598, S599, S60, S600, S601, S602, S603, S604, S605, S607, S608, S609, S61, S610, S611, S612, S613, S614, S615, S617, S618, S619, S62, S620, S621, S622, S623, S624, S625, S626, S627, S628, S629, S63, S630, S631, S632, S633, S634, S635, S636, S637, S639, S64, S640, S641, S642, S643, S644, S647, S648, S649, S65, S650, S651, S652, S653, S654, S655, S657, S658, S659, S66, S660, S661, S662, S663, S664, S665, S666, S667, S668, S669, S67, S670, S671, S672, S673, S674, S678, S679, S68, S680, S681, S682, S683, S684, S685, S686, S687, S688, S689, S69, S697, S698, S699, S70, S700, S701, S702, S703, S707, S708, S709, S71, S710, S711, S717, S718, S72, S720, S721, S722, S723, S724, S727, S728, S729, S73, S730, S731, S74, S740, S741, S742, S747, S748, S749, S75, S750, S751, S752, S757, S758, S759, S76, S760, S761, S762, S763, S764, S767, S768, S769, S77, S770, S771, S772, S78, S780, S781, S789, S79, S790, S791, S797, S798, S799, S80, S800, S801, S802, S807, S808, S809, S81, S810, S817, S818, S819, S82, S820, S821, S822, S823, S824, S825, S826, S827, S828, S829, S83, S830, S831, S832, S833, S834, S835, S836, S837, S838, S839, S84, S840, S841, S842, S847, S848, S849, S85, S850, S851, S852, S853, S854, S855, S857, S858, S859, S86, S860, S861, S862, S863, S867, S868, S869, S87, S870, S878, S88, S880, S881, S889, S89, S890, S891, S892, S893, S897, S898, S899, S90, S900, S901, S902, S903, S904, S905, S907, S908, S909, S91, S910, S911, S912, S913, S917, S92, S920, S921, S922, S923, S924, S925, S927, S929, S93, S930, S931, S932, S933, S934, S935, S936, S94, S940, S941, S942, S943, S947, S948, S949, S95, S950, S951, S952, S957, S958, S959, S96, S960, S961, S962, S967, S968, S969, S97, S970, S971, S978, S98, S980, S981, S982, S983, S984, S989, S99, S997, S998, S999, SO69, T00, T000, T001, T002, T003, T006, T008, T009, T01, T010, T011, T012, T013, T016, T018, T019, T02, T020, T021, T022, T023, T024, T025, T026, T027, T028, T029, T03, T030, T031, T032, T033, T034, T038, T039, T04, T040, T041, T042, T043, T044, T047, T048, T049, T05, T050, T051, T052, T053, T054, T055, T056, T058, T059, T06, T060, T061, T062, T063, T064, T065, T068, T07, T070, T08, T080, T09, T090, T091, T092, T093, T094, T095, T096, T098, T099, T10, T100, T11, T110, T111, T112, T113, T114, T115, T116, T118, T119, T12, T120, T121, T13, T130, T131, T132, T133, T134, T135, T136, T138, T139, T14, T140, T141, T142, T143, T144, T145, T146, T147, T148, T149, T15, T150, T151, T158, T159, T16, T161, T162, T169, T17, T170, T171, T172, T173, T174, T175, T178, T179, T18, T180, T181, T182, T183, T184, T185, T188, T189, T19, T190, T191, T192, T193, T194, T198, T199, T20, T200, T201, T202, T203, T204, T205, T206, T207, T21, T210, T211, T212, T213, T214, T215, T216, T217, T219, T22, T220, T221, T222, T223, T224, T225, T226, T227, T23, T230, T231, T232, T233, T234, T235, T236, T237, T24, T240, T241, T242, T243, T244, T245, T246, T247, T25, T250, T251, T252, T253, T254, T255, T256, T257, T26, T260, T261, T262, T263, T264, T265, T266, T267, T268, T269, T27, T270, T271, T272, T273, T274, T275, T276, T277, T28, T280, T281, T282, T283, T284, T285, T286, T287, T288, T289, T29, T290, T291, T292, T293, T294, T295, T296, T297, T30, T300, T301, T302, T303, T304, T305, T306, T307, T31, T310, T311, T312, T313, T314, T315, T316, T317, T318, T319, T32, T320, T321, T322, T323, T324, T325, T326, T327, T328, T329, T33, T330, T331, T332, T333, T334, T335, T336, T337, T338, T339, T34, T340, T341, T342, T343, T344, T345, T346, T347, T348, T349, T35, T350, T351, T352, T353, T354, T355, T356, T357, T36, T360, T361, T362, T363, T364, T365, T366, T367, T368, T369, T37, T370, T371, T372, T373, T374, T375, T378, T379, T38, T380, T381, T382, T383, T384, T385, T386, T387, T388, T389, T39, T390, T391, T392, T393, T394, T398, T399, T40, T400, T401, T402, T403, T404, T405, T406, T407, T408, T409, T41, T410, T411, T412, T413, T414, T415, T42, T420, T421, T422, T423, T424, T425, T426, T427, T428, T43, T430, T431, T432, T433, T434, T435, T436, T438, T439, T44, T440, T441, T442, T443, T444, T445, T446, T447, T448, T449, T45, T450, T451, T452, T453, T454, T455, T456, T457, T458, T459, T46, T460, T461, T462, T463, T464, T465, T466, T467, T468, T469, T47, T470, T471, T472, T473, T474, T475, T476, T477, T478, T479, T48, T480, T481, T482, T483, T484, T485, T486, T487, T489, T49, T490, T491, T492, T493, T494, T495, T496, T497, T498, T499, T50, T500, T501, T502, T503, T504, T505, T506, T507, T508, T509, T51, T510, T511, T512, T513, T518, T519, T52, T520, T521, T522, T523, T524, T528, T529, T53, T530, T531, T532, T533, T534, T535, T536, T537, T539, T54, T540, T541, T542, T543, T549, T55, T550, T551, T56, T560, T561, T562, T563, T564, T565, T566, T567, T568, T569, T57, T570, T571, T572, T573, T578, T579, T58, T580, T581, T582, T588, T589, T59, T590, T591, T592, T593, T594, T595, T596, T597, T598, T599, T60, T600, T601, T602, T603, T604, T608, T609, T61, T610, T611, T612, T617, T618, T619, T62, T620, T621, T622, T628, T629, T63, T630, T631, T632, T633, T634, T635, T636, T637, T638, T639, T64, T640, T648, T65, T650, T651, T652, T653, T654, T655, T656, T658, T659, T66, T67, T670, T671, T672, T673, T674, T675, T676, T677, T678, T679, T68, T680, T687, T69, T690, T691, T698, T699, T70, T700, T701, T702, T703, T704, T708, T709, T71, T710, T711, T712, T719, T73, T730, T731, T732, T733, T738, T739, T74, T740, T741, T742, T743, T744, T748, T749, T75, T750, T751, T752, T753, T754, T758, T76, T760, T761, T762, T763, T769, T78, T780, T781, T782, T783, T784, T788, T789, T79, T790, T791, T792, T793, T794, T795, T796, T797, T798, T799, T80, T800, T801, T802, T803, T804, T805, T806, T808, T809, T81, T810, T811, T812, T813, T814, T815, T816, T817, T818, T819, T82, T820, T821, T822, T823, T824, T825, T826, T827, T828, T829, T83, T830, T831, T832, T833, T834, T835, T836, T837, T838, T839, T84, T840, T841, T842, T843, T844, T845, T846, T847, T848, T849, T85, T850, T851, T852, T853, T854, T855, T856, T857, T858, T859, T86, T860, T861, T862, T863, T864, T865, T868, T869, T87, T870, T871, T872, T873, T874, T875, T876, T878, T879, T88, T880, T881, T882, T883, T884, T885, T886, T887, T888, T889, T90, T900, T901, T902, T903, T904, T905, T908, T909, T91, T910, T911, T912, T913, T914, T915, T918, T919, T92, T920, T921, T922, T923, T924, T925, T926, T928, T929, T93, T930, T931, T932, T933, T934, T935, T936, T938, T939, T94, T940, T941, T95, T950, T951, T952, T953, T954, T958, T959, T96, T960, T97, T970, T98, T980, T981, T982, T983, TO7, U50, Y245, Y246, Y247, Y252, Y263, Y274, Y275, Y283, Y285, Y293, Y33, Y330, Y331, Y332, Y333, Y334, Y335, Y336, Y337, Y338, Y339, Y34, Y340, Y341, Y342, Y343, Y344, Y345, Y346, Y347, Y348, Y349, Y86, Y860, Y862, Y868, Y87, Y872, Y89, Y899, Y92, Y920, Y921, Y922, Y923, Y924, Y925, Y926, Y927, Y928, Y929, Y93, Y930, Y931, Y932, Y933, Y934, Y935, Y936, Y937, Y938, Y939, Y94, Y95, Y96, Y97, Y98, Y980, Y99, Y990, Y991, Y992, Y998, Y999, W47, T720, T721, T722, T728, T729, T779, T89, T892, T897, T99, T994, T999 | MCoD |
| Eye Unspecified Site Cancer | C69, C699 | Proportional |
| Female pelvic inflammatory diseases | N70, N700, N701, N709, N71, N710, N711, N719, N73, N730, N731, N732, N733, N734, N735, N736, N738, N739, N74, N742, N743, N744, N748 | Impairments |
| Fistula | N321, N322, N82, N820, N821, N822, N823, N824, N825, N828, N829 | Proportional |
| Fluid, Electrolyte, Acid Base Disorders | E86, E860, E861, E862, E863, E864, E865, E866, E867, E868, E869, E87, E870, E871, E872, E873, E874, E875, E876, E878, E879 | MCoD |
| Gastrointestinal Bleeding | K920, K921, K922 | MCoD |
| HIV correction for Actinomycosis | A42, A420, A421, A422, A427, A428, A429 | HIV Correction |
| HIV correction for Aspergillosis | B44, B440, B441, B442, B447, B448, B449 | HIV Correction |
| HIV correction for Bartonellosis | A44, A440, A441, A448, A449 | HIV Correction |
| HIV correction for Blastomycosis | B40, B400, B401, B402, B403, B407, B408, B409 | HIV Correction |
| HIV correction for Candidiasis | B37, B370, B371, B372, B375, B376, B377, B378, B379 | HIV Correction |
| HIV correction for Coccidioidomycosis | B38, B380, B381, B382, B383, B384, B387, B388, B389 | HIV Correction |
| HIV correction for Cryptococcosis | B45, B450, B451, B452, B453, B457, B458, B459 | HIV Correction |
| HIV correction for Cutaneous leishmaniasis | B55, B551, B552, B559 | HIV Correction |
| HIV correction for Histoplasmosis | B39, B390, B391, B392, B393, B394, B395, B399 | HIV Correction |
| HIV correction for Immunodeficiency antibody | D80, D800, D801, D802, D803, D804, D805, D806, D807, D808, D809 | HIV Correction |
| HIV correction for Immunodeficiency cell | D81, D810, D811, D812, D813, D814, D815, D816, D817, D818, D819, D82, D820, D821, D822, D823, D824, D828, D829 | HIV Correction |
| HIV correction for Immunodeficiency other | D83, D830, D831, D832, D838, D839, D84, D840, D841, D848, D849, D898, D899 | HIV Correction |
| HIV correction for Kaposi's sarcoma | C46, C460, C461, C462, C463, C464, C465, C466, C467, C468, C469 | Proportional |
| HIV correction for Mycobacterial skin infection | A311, A312 | HIV Correction |
| HIV correction for Nocardiosis | A43, A430, A431, A438, A439 | HIV Correction |
| HIV correction for Other Mycobacterial infection | A31, A310, A318, A319 | HIV Correction |
| HIV correction for Paracoccidioidomycosis | B41, B410, B417, B418, B419 | HIV Correction |
| HIV correction for Pneumocystosis | B59, B590, B599 | HIV Correction |
| HIV correction for Sporotrichosis and Chromomycosis | B42, B420, B421, B427, B428, B429, B43, B430, B431, B432, B438, B439 | HIV Correction |
| HIV correction for Toxoplasmosis | B58, B580, B581, B582, B583, B588, B589 | HIV Correction |
| HIV correction for Unspecified mycosis | B49, B495, B499 | HIV Correction |
| HIV correction for Urogenital Candidiasis | B373, B374 | HIV Correction |
| HIV correction for Zygomycosis | B46, B460, B461, B462, B463, B464, B465, B468, B469 | HIV Correction |
| Haemophilus influenza infection, unspecified site | A492 | Proportional |
| Head and Neck Cancer | C760, C761, C770, C771, C780, C781, C782, C783, D17, D170, D171, D172, D173, D174, D175, D176, D177, D179, D18, D180, D181, D19, D190, D191, D197, D199, D20, D200, D201, D209, D21, D210, D211, D212, D213, D214, D215, D216, D219 | Negative correlation |
| Heart failure unspecified right or left | I50, I508, I509, J81, J811 | MCoD |
| Hepatic Failure | K71, K710, K711, K712, K713, K714, K715, K716, K718, K719, K72, K720, K721, K729, R16, R160, R161, R162, R17, R170, R179, R18, R180, R188, R189 | MCoD |
| Hepatitis Unspecified | B17, B171, B178, B179, B19, B190, B192, B199, B942 | Proportional |
| Hypertension | I10, I100, I109, I15, I150, I151, I152, I158, I159, I674, R03, R030, R040 | MCoD |
| Intermediate cause for CNS | G91, G910, G911, G912, G914, G918, G919, G92, G925, G926, G929, G93, G931, G932, G934, G935, G936, G940, G941, G942, G948 | MCoD |
| Left heart failure | I500, I501, I502, I503, I504 | MCoD |
| Liver Abscess | K750 | Proportional |
| Lymphoid leukemia unspecified by age | C914, C915, C917, C918, C919 | Proportional |
| MDS not classified | C946 | Proportional |
| Maternal Complication | O08, O080, O081, O082, O083, O084, O085, O086, O087, O088, O089 | Proportional |
| Mental Disorders | F04, F040, F05, F050, F051, F058, F059, F06, F060, F061, F062, F063, F064, F065, F066, F067, F068, F069, F07, F070, F078, F079, F08, F09, F090, F099, F20, F200, F201, F202, F203, F204, F205, F206, F208, F209, F21, F22, F220, F228, F229, F23, F230, F231, F232, F233, F238, F239, F24, F25, F250, F251, F252, F258, F259, F26, F27, F28, F280, F29, F290, F299, F30, F300, F301, F302, F303, F304, F308, F309, F31, F310, F311, F312, F313, F314, F315, F316, F317, F318, F319, F32, F320, F321, F322, F323, F324, F325, F328, F329, F33, F330, F331, F332, F333, F334, F338, F339, F34, F340, F341, F348, F349, F35, F36, F37, F38, F380, F381, F388, F39, F40, F400, F401, F402, F408, F409, F41, F410, F411, F412, F413, F418, F419, F42, F420, F421, F422, F428, F429, F43, F430, F431, F432, F438, F439, F44, F440, F441, F442, F443, F444, F445, F446, F447, F448, F449, F45, F450, F451, F452, F453, F454, F458, F459, F46, F47, F48, F480, F481, F482, F488, F489, F49, F51, F510, F511, F512, F513, F514, F515, F518, F519, F52, F520, F521, F522, F523, F524, F525, F526, F527, F528, F529, F53, F530, F531, F538, F539, F54, F55, F550, F551, F552, F553, F554, F558, F559, F56, F57, F58, F59, F60, F600, F601, F602, F603, F604, F605, F606, F607, F608, F609, F61, F62, F620, F621, F628, F629, F63, F630, F631, F632, F633, F638, F639, F64, F640, F641, F642, F648, F649, F65, F650, F651, F652, F653, F654, F655, F656, F658, F659, F66, F660, F661, F662, F668, F669, F67, F68, F680, F681, F688, F69, F690, F70, F700, F701, F708, F709, F71, F710, F711, F718, F719, F72, F720, F721, F728, F729, F73, F730, F731, F738, F739, F74, F75, F76, F77, F78, F780, F781, F788, F789, F79, F790, F791, F798, F799, F80, F800, F801, F802, F803, F804, F808, F809, F81, F810, F811, F812, F813, F818, F819, F82, F820, F83, F84, F840, F841, F842, F843, F844, F845, F848, F849, F85, F86, F87, F88, F89, F890, F90, F900, F901, F902, F908, F909, F91, F910, F911, F912, F913, F918, F919, F92, F920, F928, F929, F93, F930, F931, F932, F933, F938, F939, F94, F940, F941, F942, F948, F949, F95, F950, F951, F952, F958, F959, F96, F97, F98, F980, F981, F982, F983, F984, F985, F986, F988, F989, F99, F990 | Linkage |
| Myocardial Degeneration | I515 | Proportional |
| Nausea and Vomiting | R11, R110, R111, R112, R119 | Proportional |
| Non-follicular lymphoma, unspecified | C83, C839, C851, C859 | Proportional |
| Osteomyelitis | M86, M860, M861, M862, M865, M866, M867, M868, M869 | MCoD |
| Peritonitis & Acute Abdomen | K65, K650, K651, K652, K653, K654, K658, K659, K66, K660, K661, K669, K681, K689, R10, R100, R101, R102, R103, R104, R108, R109 | MCoD |
| Plegia | G81, G810, G811, G819, G82, G820, G822, G823, G825, G829, G83, G831, G832, G833, G834, G835, G839 | MCoD |
| Pleurisy, Pyothorax | J86, J860, J869, J90, J900, J94, J940, J941, J948, J949, R091 | MCoD |
| Pneumoconiosis associated with tuberculosis | J65, J650 | Proportional |
| Pneumonitis | J69, J690, J691, J698, J699, J85, J850, J851, J852, J853 | MCoD |
| Pneumothorax | I312, I313, I314, J93, J930, J931, J938, J939, J942, R04, R041, R042, R048, R049 | MCoD |
| Primary or secondary Liver Cancer Unspecified | C229 | Proportional |
| Pulmonary Embolism | I26, I260, I269 | MCoD |
| Self-harm by unspecified means | X84, X840, X841, X842, X843, X844, X845, X846, X847, X848, X849 | Proportional |
| Self-poisoning unspecified | X64, X640, X641, X642, X643, X644, X645, X646, X647, X648, X649, X69, X690, X691, X692, X693, X694, X695, X696, X697, X698, X699 | Proportional |
| Senility | R54, R540, R549 | Proportional |
| Sepsis (Non- maternal and neonatal sepsis) | A40, A400, A401, A402, A403, A408, A409, A41, A410, A411, A412, A413, A414, A415, A416, A418, A419, A480, A483, A490, A491, D65, D650, D659, I76, R02, R020, R029, R50, R500, R501, R508, R509, R560, R652 | MCoD |
| Shock, Cardiac Arrest, Coma | I46, I460, I461, I462, I466, I468, I469, I95, I950, I951, I958, I959, R031, R090, R092, R098, R40, R400, R401, R402, R403, R404, R55, R56, R561, R566, R568, R569, R57, R570, R571, R574, R576, R578, R579 | MCoD |
| Typhoid or paratyphoid fevers | A01 | Proportional |
| Undetermined intent Drowning | Y21, Y210, Y211, Y212, Y213, Y214, Y215, Y216, Y217, Y218, Y219 | Negative correlation |
| Undetermined intent shooting by Handgun Firearm | Y22, Y220, Y221, Y222, Y223, Y224, Y225, Y226, Y227, Y228, Y229 | Negative correlation |
| Undetermined intent shooting by rifle and larger firearm | Y23, Y230, Y231, Y232, Y234, Y235, Y236, Y237 | Negative correlation |
| Undetermined intent shooting by unspecified firearm | Y233, Y238, Y239, Y24, Y240, Y241, Y242, Y243, Y244, Y248, Y249 | Negative correlation |
| Undetermined intent Poisoning by antiepileptic and psychotropic drugs | Y11, Y110, Y111, Y112, Y113, Y114, Y115, Y116, Y117, Y118, Y119 | Negative correlation |
| Undetermined intent Poisoning by autonomic nervous system drugs | Y13, Y130, Y131, Y132, Y133, Y134, Y135, Y136, Y137, Y138, Y139 | Negative correlation |
| Undetermined intent Poisoning by multiple or unspecified drug | F19, F190, F191, F192, F193, F194, F195, F196, F197, F198, F199, X40, X400, X401, X402, X404, X405, X406, X407, X408, X409, X41, X410, X411, X412, X413, X414, X415, X416, X417, X418, X419, X42, X420, X421, X422, X423, X424, X425, X426, X427, X428, X429, X43, X430, X431, X432, X433, X434, X435, X436, X437, X438, X439, X44, X440, X441, X442, X443, X444, X445, X446, X447, X448, X449, X46, X460, X461, X462, X463, X464, X465, X466, X467, X468, X469, X49, X490, X491, X492, X493, X494, X495, X496, X497, X498, X499 | MCoD |
| Undetermined intent Poisoning by narcotics and psychodysleptics drugs | Y12, Y120, Y121, Y122, Y123, Y124, Y125, Y126, Y127, Y128, Y129 | Negative correlation |
| Undetermined intent Poisoning by no opioid analgesics | Y10, Y100, Y101, Y102, Y103, Y104, Y105, Y106, Y107, Y108, Y109 | Negative correlation |
| Undetermined intent Poisoning by other gases and vapors | Y17, Y170, Y171, Y172, Y173, Y174, Y175, Y176, Y177, Y178, Y179 | Negative correlation |
| Undetermined intent Poisoning by pesticides | Y18, Y180, Y181, Y182, Y183, Y184, Y185, Y186, Y187, Y188, Y189 | Negative correlation |
| Undetermined intent Poisoning by solvents and halogenated hydrocarbons | Y16, Y160, Y161, Y162, Y163, Y164, Y165, Y166, Y167, Y168, Y169 | Negative correlation |
| Undetermined intent Poisoning by unspecified chemicals and noxious substances | Y19, Y190, Y191, Y192, Y193, Y194, Y195, Y196, Y197, Y198, Y199 | Negative correlation |
| Undetermined intent Poisoning by unspecified drugs and biological drugs | Y14, Y140, Y141, Y142, Y143, Y144, Y145, Y146, Y147, Y148, Y149 | Negative correlation |
| Undetermined intent Strangulation | Y20, Y200, Y201, Y202, Y203, Y204, Y205, Y206, Y207, Y208, Y209 | Negative correlation |
| Undetermined intent of Blunt Objects | Y29, Y290 | Negative correlation |
| Undetermined intent of Crashing | Y32, Y320, Y321, Y322, Y323, Y324, Y325, Y326, Y327, Y328, Y329 | Negative correlation |
| Undetermined intent of Explosion | Y25, Y250, Y251, Y254, Y255, Y256, Y257, Y258, Y259 | Negative correlation |
| Undetermined intent of Hot Objects | Y27, Y270, Y271, Y272, Y273, Y276, Y277, Y278, Y279 | Negative correlation |
| Undetermined intent of Moving Objects | Y31, Y310, Y311, Y312, Y313, Y314, Y315, Y316, Y317, Y318, Y319 | Negative correlation |
| Undetermined intent of Sharp Objects | Y28, Y280, Y281, Y282, Y284, Y286, Y287, Y288, Y289 | Negative correlation |
| Undetermined intent of fall | Y291, Y292, Y294, Y295, Y296, Y297, Y298, Y299, Y30, Y300, Y301, Y302, Y303, Y304, Y305, Y306, Y307, Y308, Y309 | Negative correlation |
| Undetermined intent of fire and flames | Y26, Y260, Y261, Y262, Y264, Y265, Y266, Y267, Y268, Y269 | Negative correlation |
| Unspecified Intestine Diseases | K21, K210, K219, K227, K63, K630, K631, K632, K633, K634, K638, K639 | Proportional |
| Unspecified Bacterial Diseases | A48, A488, A49, A493, A498, A499 | Proportional |
| Unspecified Blood Diseases | D759, D79, D85, D87, D88, D90, D91, D92, D93, D94, D95, D96, D97, D98, D99 | Proportional |
| Unspecified Brain Diseases | G938, G939, G94, G96, G960, G961, G968, G969, G98, G980, G988, G989 | Proportional |
| Unspecified Bronchitis and Bronchiectasis | J40, J400, J409, J47, J470, J471, J479 | Proportional |
| Unspecified CNS Diseases | G15, G16, G17, G18, G19, G21, G212, G214, G218, G219, G22, G220, G27, G28, G29, G33, G34, G38, G42, G48, G49, G66, G67, G68, G69, G74, G75, G76, G77, G78, G79, G84, G85, G86, G87, G88, G39 | Proportional |
| Unspecified CNS Infection | G09, G090, G099 | Proportional |
| Unspecified Cardiomyopathy | I42, I420, I429 | Proportional |
| Unspecified Chromosomal Diseases | Q999 | Proportional |
| Unspecified Congenital Diseases | Q08, Q09, Q10, Q100, Q101, Q102, Q103, Q19, Q46, Q47, Q48, Q49, Q88, Q899, Q94, Q29 | Proportional |
| Unspecified Diabetes Related cause | E08, E080, E081, E082, E083, E084, E085, E086, E088, E089 | Proportional |
| Unspecified Digestive Diseases | K319, K32, K33, K34, K39, K48, K49, K53, K54, K69, K78, K79, K84, K88, K89, K92, K929, K93, K96, K97, K98, K99, K47 | Proportional |
| Unspecified Eating Disorders | F50, F508, F509 | Proportional |
| Unspecified Endo/Metabolic Diseases | E17, E18, E19, E35, E37, E38, E39, E47, E48, E62, E69, E90, E900, E901, E902, E903, E904, E905, E906, E907, E908, E909, E91, E910, E911, E912, E913, E914, E915, E916, E917, E918, E919, E92, E920, E921, E922, E923, E924, E925, E926, E927, E928, E929, E93, E930, E931, E932, E933, E934, E935, E936, E937, E938, E939, E94, E940, E941, E942, E943, E944, E945, E946, E947, E948, E949, E95, E950, E951, E952, E953, E954, E955, E956, E957, E958, E959, E96, E960, E961, E962, E963, E965, E966, E967, E968, E969, E97, E970, E971, E973, E974, E975, E976, E977, E978, E979, E98, E980, E981, E982, E983, E984, E985, E986, E987, E988, E989, E99, E999, E990, E991, E993, E995, E996, E997, E998, E49 | Proportional |
| Unspecified Endocrine Cancer | C759, D44, D449, D497, E340 | Negative correlation |
| Unspecified Endocrine Diseases | E349, E350, E351, E358 | Proportional |
| Unspecified Female Genital Cancer | C579, C59, C6, D073, D28, D289, D39, D390, D397, D399, N842, N843, N848 | Negative correlation |
| Unspecified GI Cancer | C26, C260, C261, C262, C268, C269, C27, C28, C29, C35, C36, D0, D00, D01, D014, D015, D017, D019, D13, D139, D37, D376, D377, D378, D379, D490 | Negative correlation |
| Unspecified Gynecologic Diseases | N92, N920, N921, N922, N923, N924, N925, N926, N93, N930, N938, N939, N94, N940, N941, N942, N943, N944, N945, N946, N948, N949, N950 | Proportional |
| Unspecified Heart Diseases | I51, I517, I518, I519 | Proportional |
| Unspecified Hemorrhagic Fever | A99, A990 | Proportional |
| Unspecified Infectious Diseases | A149, A29, A30, A300, A301, A302, A303, A304, A305, A308, A309, A45, A459, A47, A61, A62, A72, A73, A76, A97, B11, B12, B13, B14, B28, B29, B31, B319, B32, B323, B324, B61, B62, B68, B680, B681, B689, B73, B730, B731, B74, B740, B741, B742, B76, B760, B761, B768, B769, B79, B80, B81, B810, B811, B812, B813, B814, B818, B84, B92, B93, B94, B948, B949, B956, B957, B958, B96, B960, B961, B962, B963, B964, B965, B966, B967, B968, B97, B970, B971, B973, B977, B978, B98, B99, B990, B998, B999, B980, B981 | Proportional |
| Unspecified Intestinal Parasite | B82, B820, B829, B839 | Proportional |
| Unspecified Malaria | B54, B540 | Proportional |
| Unspecified Male Genital Cancer | C639, D076, D29, D299, D4, D40, D409 | Negative correlation |
| Unspecified Maternal Diseases | O17, O18, O180, O19, O27, O37, O38, O384, O39, O49, O50, O51, O52, O53, O54, O55, O56, O57, O58, O59, O78, O79, O93, O94, O95, O959 | Proportional |
| Unspecified Meningitis | G00, G009, G01, G010, G02, G020, G021, G039 | Proportional |
| Unspecified Neonatal Diseases | P06, P09, P16, P18, P19, P190, P191, P192, P199, P30, P31, P32, P33, P34, P342, P40, P41, P42, P43, P44, P45, P46, P47, P48, P49, P62, P63, P64, P65, P66, P67, P68, P69, P73, P74, P740, P741, P742, P743, P744, P745, P746, P748, P749, P75, P750, P79, P80, P800, P808, P809, P81, P810, P818, P819, P82, P85, P86, P87, P88, P89, P92, P920, P921, P922, P923, P924, P925, P926, P928, P929, P969, P97, P98, P99, P999, P17 | Proportional |
| Unspecified Oropharynx Cancer | C14, C140, C141, C142, C143, C148, C149, D000, D10, D109, D370 | Negative correlation |
| Unspecified Parasitic Diseases | B89 | Proportional |
| Unspecified Pneumoconiosis | J64, J640, J649 | Proportional |
| Unspecified Protozoal Diseases | B64 | Proportional |
| Unspecified Respiratory Cancer | C39, C390, C398, C399, D02, D024, D029, D14, D144, D38, D386, D491 | Negative correlation |
| Unspecified Road Injuries | V89, V890, V891, V892, V893, V894, V899 | Negative correlation |
| Unspecified STD | A64, A640 | Proportional |
| Unspecified Site Cancer | C42, C76, C767, C768, C769, C77, C773, C774, C778, C779, C78, C79, C792, C793, C794, C795, C796, C797, C798, C799, C8, C80, C800, C801, C802, C809, C87, C97, C970, C979, C98, C99, D08, D09, D097, D099, D360, D369, D48, D487, D489, D49, D498, D499, D54 | Negative correlation |
| Unspecified Skin Diseases | L06, L07, L09, L15, L16, L17, L18, L19, L31, L32, L33, L34, L35, L36, L37, L38, L39, L69, L77, L78, L79 | Proportional |
| Unspecified Thyroid Diseases | E078, E079 | Proportional |
| Unspecified Traffic Injuries | V87, V870, V871, V874, V875, V876, V877, V878, V879, V88, V880, V881, V884, V885, V886, V887, V888, V889 | Proportional |
| Unspecified Transport Injuries | V99, V990, Y85, Y850, Y859 | Negative correlation |
| Unspecified Urinary Cancer | C68, C689, D091, D30, D309, D41, D419 | Negative correlation |
| Unspecified Urinary Diseases | N09, N24, N288, N289, N38, N399, N40, N400, N401, N402, N403, N409, N54, N55, N56, N57, N58, N59, N66, N67, N68, N69, N78, N79, N84, N849, N85, N850, N851, N852, N853, N854, N855, N856, N857, N858, N859, N86, N88, N880, N881, N882, N883, N884, N888, N889, N89, N890, N891, N892, N893, N894, N895, N896, N897, N898, N899, N90, N900, N901, N902, N903, N904, N905, N906, N907, N908, N909 | Proportional |
| Unspecified Uterus Cancer | C55, C550, C551, C559 | Negative correlation |
| Unspecified Viral Diseases | B08, B080, B081, B082, B083, B084, B085, B086, B087, B088, B09, B34, B340, B341, B343, B344, B348, B349, G933 | Proportional |
| Unspecified acute respiratory infectious | J07, J08, J23, J24, J25, J26, J27, J28, J29 | Proportional |
| Unspecified cardiovascular diseases | I000, I16, I169, I17, I19, I29, I299, I516, I52, I520, I521, I528, I53, I54, I55, I56, I57, I58, I59, I90, I919, I92, I93, I939, I94, I96, I960, I969, I984, I988, I99, I990, I998, I999, ID59, I03, I04, I14, I18 | Proportional |
| Unspecified chronic respiratory diseases | J48, J49, J499, J50, J51, J52, J53, J54, J55, J56, J57, J58, J59, J71, J712, J72, J73, J74, J75, J76, J77, J78, J79, J819, J83, J859, J87, J88, J89, J909, J936, J97, J98, J980, J984, J985, J986, J988, J989, J99, J990, J991, J998 | Proportional |
| Unspecified genital Cancer | D07, D495 | Negative correlation |
| Unspecified lower respiratory infectious | J159, J17, J170, J171, J172, J173, J178, J18, J180, J181, J182, J187, J188, J189, J19, J196, J22, J220, J229, P23, P235, P236, P238, P239 | Proportional |
| Unspecified sign and symptom for GI diseases | R13, R130, R131, R139 | Proportional |
| Unspecified sign and symptom for Heart diseases | R00, R000, R001, R002, R008, R009, R01, R010, R011, R012, R07, R071, R072, R073, R074, R078, R079 | Proportional |
| Unspecified sign and symptom for Respiratory diseases | R05, R050, R06, R060, R061, R062, R063, R064, R065, R066, R067, R068, R069, R230 | Proportional |
| Unspecified type of Stroke | I64, I640, I641, I649, I67, I678, I679, I68, I688, I69, I694, I698, I699 | Negative correlation |
| Unspecified upper respiratory infectious | J029, J039, J043, J06, J069 | Proportional |
| Urinary Obstruction Diseases | N13, N130, N131, N132, N133, N134, N135, N137, N138, N139, R31, R310, R311, R312, R319 | Proportional |
| Valve Disorder and Endocarditis | I379 | Proportional |
| hepatitis B unspecified | B169 | Proportional |
| myeloid leukemia by age | C927, C928, C929, C932, C935, C937, C939 | Proportional |
| right heart failure and pulmonary heart disease | I27, I278, I279 | MCoD |
| upper and lower limb cancer | C764, C765 | Proportional |

Table S4: Garbage code hierarchy by Disease

| Table 4: Garbage code hierarchy by Disease | | | |
| --- | --- | --- | --- |
| **Name** | **Short name** | **Level** | **Parent** |
| Other ill-defined and non-specific diseases and conditions | Other | 1 | all |
| Blood and endocrine diseases | Blood, Endo | 1 | all |
| Cardiovascular diseases | CVD | 1 | all |
| Digestive diseases | Digestive | 1 | all |
| Infectious diseases | Infectious | 1 | all |
| Injuries | Inj | 1 | all |
| Maternal, neonatal, congenital diseases | MNC | 1 | all |
| Mental and neurological diseases | Mental/Neuro | 1 | all |
| Cancers | Cancer | 1 | all |
| Respiratory diseases | Respiratory | 1 | all |
| Skin diseases | Skin | 1 | all |
| Genitourinary diseases | Genitourinary | 1 | all |
| Special signs and symptoms conditions | All special signs | 1 | all |
| All, Ill Defined code for causes of death | Ill Defined Overall | 2 | Other |
| Senility | Ill Defined Overall | 2 | Other |
| Unspecified Endo/Metabolic Diseases | Ill Defined Blood, Endo | 2 | Blood, Endo |
| Anemia in neoplastic Diseases | Ill Defined Blood, Endo | 2 | Blood, Endo |
| Acquired hemolytic anemia | Ill Defined Blood, Endo | 2 | Blood, Endo |
| Unspecified Blood Diseases | Ill Defined Blood, Endo | 2 | Blood, Endo |
| Unspecified Thyroid Diseases | Ill Defined Blood, Endo | 2 | Blood, Endo |
| Unspecified Diabetes Related cause | Ill Defined Blood, Endo | 2 | Blood, Endo |
| Unspecified Endocrine Diseases | Ill Defined Blood, Endo | 2 | Blood, Endo |
| Anemia Unspecified | Ill Defined Blood, Endo | 2 | Blood, Endo |
| Anemia Others | Ill Defined Blood, Endo | 2 | Blood, Endo |
| Diabetes unspecified type | Ill Defined Blood, Endo | 2 | Blood, Endo |
| CKD due to diabetes Unspecified type | Ill Defined Blood, Endo | 2 | Blood, Endo |
| Myocardial Degeneration | Ill Defined CVD | 2 | CVD |
| Valve Disorder and Endocarditis | Ill Defined CVD | 2 | CVD |
| Unspecified cardiovascular diseases | Ill Defined CVD | 2 | CVD |
| Unspecified Heart Diseases | Ill Defined CVD | 2 | CVD |
| Alcoholic hepatic failure | Ill Defined Digestive | 2 | Digestive |
| Unspecified Digestive Diseases | Ill Defined Digestive | 2 | Digestive |
| Unspecified Intestine Diseases | Ill Defined Digestive | 2 | Digestive |
| Other Non-collision motor vehicle traffic accident | Ill Defined Injuries | 2 | Inj |
| HIV correction for Kaposi's sarcoma | Ill Defined Infectious | 2 | Infectious |
| HIV correction for infectious diseases (AD-Tab10) | Ill Defined Infectious | 2 | Infectious |
| HIV correction for endocrean diseases (AD-Tab10) | Ill Defined Infectious | 2 | Infectious |
| Unspecified Intestinal Parasite | Ill Defined Infectious | 2 | Infectious |
| Unspecified Parasitic Diseases | Ill Defined Infectious | 2 | Infectious |
| Typhoid or paratyphoid fevers | Ill Defined Infectious | 2 | Infectious |
| Haemophilus influenza infection, unspecified site | Ill Defined Infectious | 2 | Infectious |
| Unspecified Malaria | Ill Defined Infectious | 2 | Infectious |
| Hepatitis Unspecified | Ill Defined Infectious | 2 | Infectious |
| hepatitis B unspecified | Ill Defined Infectious | 2 | Infectious |
| Unspecified Infectious Diseases | Ill Defined Infectious | 2 | Infectious |
| Unspecified Bacterial Diseases | Ill Defined Infectious | 2 | Infectious |
| Unspecified STD | Ill Defined Infectious | 2 | Infectious |
| Unspecified Hemorrhagic Fever | Ill Defined Infectious | 2 | Infectious |
| Unspecified Viral Diseases | Ill Defined Infectious | 2 | Infectious |
| Unspecified Malaria | Ill Defined Infectious | 2 | Infectious |
| Unspecified Protozoal Diseases | Ill Defined Infectious | 2 | Infectious |
| Female pelvic inflammatory diseases | Ill Defined Infectious | 2 | Infectious |
| Self-harm by unspecified means | Ill Defined Injuries | 2 | Inj |
| Exposure to unspecified factor X59 | Ill Defined Injuries | 2 | Inj |
| Undetermined intent Poisoning by multiple or unspecified drug | Ill Defined Injuries | 2 | Inj |
| Unspecified Traffic Injuries | Ill Defined Injuries | 2 | Inj |
| Assault by unspecified means | Ill Defined Injuries | 2 | Inj |
| Unspecified Transport Injuries | Ill Defined Injuries | 2 | Inj |
| Unspecified Road Injuries | Ill Defined Injuries | 2 | Inj |
| Undetermined intent Poisoning by antiepileptic and psychotropic drugs | Ill Defined Injuries | 2 | Inj |
| Undetermined intent Poisoning by narcotics and psychodysleptics drugs | Ill Defined Injuries | 2 | Inj |
| Undetermined intent Poisoning by unspecified drugs and biological drugs | Ill Defined Injuries | 2 | Inj |
| Undetermined intent Poisoning by no opioid analgesics | Ill Defined Injuries | 2 | Inj |
| Undetermined intent Poisoning by autonomic nervous system drugs | Ill Defined Injuries | 2 | Inj |
| Undetermined intent Poisoning by solvents and halogenated hydrocarbons | Ill Defined Injuries | 2 | Inj |
| Undetermined intent Poisoning by other gases and vapors | Ill Defined Injuries | 2 | Inj |
| Undetermined intent Poisoning by pesticides | Ill Defined Injuries | 2 | Inj |
| Undetermined intent Poisoning by unspecified chemicals and noxious substances | Ill Defined Injuries | 2 | Inj |
| Undetermined intent Strangulation | Ill Defined Injuries | 2 | Inj |
| Undetermined intent Drowning | Ill Defined Injuries | 2 | Inj |
| Undetermined intent shooting by Handgun Firearm | Ill Defined Injuries | 2 | Inj |
| Undetermined intent shooting by rifle and larger firearm | Ill Defined Injuries | 2 | Inj |
| Undetermined intent shooting by unspecified firearm | Ill Defined Injuries | 2 | Inj |
| Undetermined intent of Explosion | Ill Defined Injuries | 2 | Inj |
| Undetermined intent of fire and flames | Ill Defined Injuries | 2 | Inj |
| Undetermined intent of Hot Objects | Ill Defined Injuries | 2 | Inj |
| Undetermined intent of Sharp Objects | Ill Defined Injuries | 2 | Inj |
| Undetermined intent of Blunt Objects | Ill Defined Injuries | 2 | Inj |
| Undetermined intent of fall | Ill Defined Injuries | 2 | Inj |
| Undetermined intent of Moving Objects | Ill Defined Injuries | 2 | Inj |
| Undetermined intent of Crashing | Ill Defined Injuries | 2 | Inj |
| self-poisoning unspecified | Ill Defined Injuries | 2 | Inj |
| Maternal Complication | Ill Defined MNC | 2 | MNC |
| Unspecified Maternal Diseases | Ill Defined MNC | 2 | MNC |
| Mental disorders | Ill Defined Mental & Neuro | 2 | Mental/Neuro |
| External Causes UDI, type unspecified | Ill Defined Mental & Neuro | 2 | Mental/Neuro |
| Tobacco death | Ill Defined Mental & Neuro | 2 | Mental/Neuro |
| Unspecified Mental/Brain Disorders | Ill Defined Mental & Neuro | 2 | Mental/Neuro |
| Unspecified Eating Disorders | Ill Defined Mental & Neuro | 2 | Mental/Neuro |
| Unspecified Meningitis | Ill Defined Mental & Neuro | 2 | Mental/Neuro |
| Unspecified CNS Infection | Ill Defined Mental & Neuro | 2 | Mental/Neuro |
| Unspecified CNS Diseases | Ill Defined Mental & Neuro | 2 | Mental/Neuro |
| Unspecified Brain Diseases | Ill Defined Mental & Neuro | 2 | Mental/Neuro |
| Unspecified Neonatal Diseases | Ill Defined MNC | 2 | MNC |
| Unspecified Congenital Diseases | Ill Defined MNC | 2 | MNC |
| Unspecified Chromosomal Diseases | Ill Defined MNC | 2 | MNC |
| Primary or secondary Liver Cancer Unspecified | Ill Defined Cancers | 2 | Cancer |
| Eye Unspecified Site Cancer | Ill Defined Cancers | 2 | Cancer |
| Adrenal Unspecified Site Cancer-parent cause | Ill Defined Cancers | 2 | Cancer |
| Chronic lymphocytic leukemia by age | Ill Defined Cancers | 2 | Cancer |
| Lymphoid leukemia unspecified by age | Ill Defined Cancers | 2 | Cancer |
| myeloid leukemia by age | Ill Defined Cancers | 2 | Cancer |
| Adrenal Unspecified Site Cancer in medulla or cortex | Ill Defined Cancers | 2 | Cancer |
| Adrenal Site Cancer unspecified part of adrenal gland | Ill Defined Cancers | 2 | Cancer |
| MDS not classified | Ill Defined Cancers | 2 | Cancer |
| upper and lower limb cancer | Ill Defined Cancers | 2 | Cancer |
| Non-follicular lymphoma, unspecified | Ill Defined Cancers | 2 | Cancer |
| Unspecified Oropharynx Cancer | Ill Defined Cancers | 2 | Cancer |
| Unspecified GI Cancer | Ill Defined Cancers | 2 | Cancer |
| Unspecified Respiratory Cancer | Ill Defined Cancers | 2 | Cancer |
| Unspecified Uterus Cancer | Ill Defined Cancers | 2 | Cancer |
| Unspecified Female Genital Cancer | Ill Defined Cancers | 2 | Cancer |
| Unspecified Male Genital Cancer | Ill Defined Cancers | 2 | Cancer |
| Unspecified Urinary Cancer | Ill Defined Cancers | 2 | Cancer |
| Unspecified Endocrine Cancer | Ill Defined Cancers | 2 | Cancer |
| Unspecified Site Cancer | Ill Defined Cancers | 2 | Cancer |
| Head and Neck Cancer | Ill Defined Cancers | 2 | Cancer |
| Abdomen and Pelvis Cancer | Ill Defined Cancers | 2 | Cancer |
| Unspecified genital Cancer | Ill Defined Cancers | 2 | Cancer |
| Unspecified upper respiratory infectious | Ill Defined Respiratory | 2 | Respiratory |
| Unspecified Bronchitis and Bronchiectasis | Ill Defined Respiratory | 2 | Respiratory |
| Pneumoconiosis with tb | Ill Defined Respiratory | 2 | Respiratory |
| Unspecified acute respiratory infectious | Ill Defined Respiratory | 2 | Respiratory |
| Unspecified lower respiratory infectious | Ill Defined Respiratory | 2 | Respiratory |
| Unspecified chronic respiratory diseases | Ill Defined Respiratory | 2 | Respiratory |
| Unspecified Pneumoconiosis | Ill Defined Respiratory | 2 | Respiratory |
| Unspecified Skin Diseases | Ill Defined Skin | 2 | Skin |
| Unspecified Urinary Diseases | Ill Defined Genitourinary | 2 | Genitourinary |
| Hypertension | Intermediate CVD | 2 | CVD |
| Atherosclerosis | Intermediate CVD | 2 | CVD |
| Chronic lymphocytic leukemia by age | Intermediate CVD | 2 | CVD |
| right heart failure and pulmonary heart disease | Intermediate CVD | 2 | CVD |
| Left heart failure | Intermediate CVD | 2 | CVD |
| Heart failure unspecified right or left | Intermediate CVD | 2 | CVD |
| Pulmonary Embolism | Intermediate CVD | 2 | CVD |
| Unspecified type of Stroke | Intermediate CVD | 2 | CVD |
| Hepatic Failure | Intermediate Digestive | 2 | Digestive |
| Peritonitis and Acute Abdomen | Intermediate Digestive | 2 | Digestive |
| Liver Abscess | Intermediate Digestive | 2 | Digestive |
| AKI and Renal Failure | Intermediate Genitourinary | 2 | Genitourinary |
| Urinary Obstruction Diseases | Intermediate Genitourinary | 2 | Genitourinary |
| Fistula | Intermediate Genitourinary | 2 | Genitourinary |
| Unspecified CNS sign and symptom | Intermediate Neuro | 2 | Mental/Neuro |
| CNS Abscess | Intermediate Neuro | 2 | Mental/Neuro |
| Cerebral Cysts | Intermediate Neuro | 2 | Mental/Neuro |
| Arterial Embolism | Intermediate Neuro | 2 | Mental/Neuro |
| Arrhythmia,Flutter and Fibrillation | Intermediate Neuro | 2 | Mental/Neuro |
| Unspecified Cardiomyopathy | Intermediate Neuro | 2 | Mental/Neuro |
| Pneumonitis | Intermediate Respiratory | 2 | Respiratory |
| Acute Respiratory Failure | Intermediate Respiratory | 2 | Respiratory |
| Chronic respiratory failure | Intermediate Respiratory | 2 | Respiratory |
| Pyothorax and Pleurisy | Intermediate Respiratory | 2 | Respiratory |
| Pneumothorax | Intermediate Respiratory | 2 | Respiratory |
| Shock/coma/asphyxia/convolsion | Intermediate Overall | 2 | Other |
| Acid-Base Balance Disorders | Intermediate Overall | 2 | Other |
| CNS Fluid Diseases | Intermediate Neuro | 2 | Mental/Neuro |
| Osteomyelitis | Intermediate Overall | 2 | Other |
| Amyloidosis | Intermediate Overall | 2 | Other |
| Sepsis and FUO(Non- maternal and neonatal sepsis) | Intermediate Overall | 2 | Other |
| Cerebral palsy | Intermediate Neuro | 2 | Mental/Neuro |
| Intermediate cause for CNS | Intermediate Neuro | 2 | Mental/Neuro |
| Gastrointestinal Bleeding | Special Signs | 2 | All special signs |
| Respiratory Bleeding and Hemothorax | Special Signs | 2 | All special signs |
| Hemi/Mono/Paraplegia | Special Signs | 2 | All special signs |
| Monoplegia | Special Signs | 2 | All special signs |
| Paraplegia | Special Signs | 2 | All special signs |
| Cachexia | Special Signs | 2 | All special signs |
| Unspecified Gynecologic Diseases | Intermediate Genitourinary | 2 | Genitoruinary |
| Unspecified sign and symptom for Heart diseases | Special Signs | 2 | All special signs |
| Unspecified sign and symptom for Respiratory diseases | Special Signs | 2 | All special signs |
| Unspecified sign and symptom for GI diseases | Special Signs | 2 | All special signs |
| Nausea and Vomiting | Special Signs | 2 | All special signs |
| Dementia | Ill Defined Neuro | 2 | Mental/Neuro |
| HIV correction for gynecological candidiasis | Ill Defined Infectious | 2 | Infectious |
| HIV correction for leishmaniasis | Ill Defined Infectious | 2 | Infectious |
| HIV correction for other infectious diseases | Ill Defined Infectious | 2 | Infectious |
| HIV correction for immunological and endocrine diseases | Ill Defined Infectious | 2 | Infectious |
| HIV correction for other HIV complications | Ill Defined Infectious | 2 | Infectious |
| HIV correction for immunological and anti-endocrine diseases | Ill Defined Infectious | 2 | Infectious |
| HIV correction for immunological and cellular endocrine diseases | Ill Defined Infectious | 2 | Infectious |
| Mental disorders | Ill Defined Mental & Neuro | 2 | Mental/Neuro |
| Unspecified birth injury | Ill Defined MNC | 2 | MNC |
| Senility | Ill Defined Overall | 2 | Other |
| HIV correction for Candidiasis | Ill Defined Infectious | 2 | Infectious |
| HIV correction for Aspergillosis | Ill Defined Infectious | 2 | Infectious |
| HIV correction for Other Mycobacterial infection | Ill Defined Infectious | 2 | Infectious |
| HIV correction for Actinomycosis | Ill Defined Infectious | 2 | Infectious |

Table S5: Relative distribution of garbage among all garbage codes by education, 1997, 2023 and all years

| **Table S5. Relative distribution of garbage among all garbage codes by education, 1997, 2023, and all years** | | | | | | | | | |
| --- | --- | --- | --- | --- | --- | --- | --- | --- | --- |
| **Garbage category** | **Low educated** | | | **Intermediate educated** | | | **High educated** | | |
|  | **1997** | **2023** | **All years** | **1997** | **2023** | **All years** | **1997** | **2023** | **All years** |
| Blood, Endo | 6.9 (6.4 - 7.3) | 5.3 (4.8 - 5.7) | 6.4 (6.3 - 6.5) | 5.7 (5.0 - 6.5) | 4.7 (4.3 - 5.1) | 5.9 (5.8 - 6.0) | 4.3 (2.6 - 6.0) | 3.3 (2.7 - 4.0) | 4.5 (4.3 - 4.7) |
| CVD | 43.3 (42.4 - 44.3) | 35.8 (34.9 - 36.8) | 42.0 (41.8 - 42.2) | 36.7 (35.2 - 38.2) | 31.9 (31.0 - 32.8) | 35.6 (35.4 - 35.8) | 30.5 (26.7 - 34.3) | 32.3 (30.5 - 34.1) | 33.6 (33.1 - 34.1) |
| Cancer | 15.9 (15.2 - 16.6) | 9.2 (8.6 - 9.7) | 11.6 (11.5 - 11.7) | 19.5 (18.3 - 20.7) | 12.3 (11.7 - 12.9) | 14.1 (13.9 - 14.2) | 25.3 (21.7 - 28.9) | 11.9 (10.7 - 13.1) | 15.4 (15.1 - 15.8) |
| Digestive | 1.1 (0.9 - 1.3) | 1.1 (0.9 - 1.3) | 1.1 (1.1 - 1.1) | 1.2 (0.8 - 1.5) | 2.1 (1.8 - 2.3) | 1.7 (1.6 - 1.7) | 1.1 (0.2 - 1.9) | 1.6 (1.1 - 2.1) | 1.5 (1.4 - 1.6) |
| Genitourinary | 1.9 (1.7 - 2.2) | 1.6 (1.4 - 1.9) | 1.8 (1.7 - 1.8) | 1.3 (1.0 - 1.7) | 1.6 (1.3 - 1.8) | 1.5 (1.5 - 1.6) | 1.4 (0.4 - 2.4) | 1.3 (0.9 - 1.7) | 1.4 (1.3 - 1.5) |
| Infectious | 1.0 (0.8 - 1.2) | 5.0 (4.6 - 5.5) | 2.5 (2.4 - 2.5) | 0.8 (0.5 - 1.1) | 4.3 (4.0 - 4.7) | 2.6 (2.5 - 2.6) | 1.4 (0.4 - 2.4) | 4.0 (3.3 - 4.8) | 2.7 (2.5 - 2.9) |
| Inj | 5.0 (4.6 - 5.5) | 7.3 (6.7 - 7.8) | 6.0 (5.9 - 6.1) | 9.0 (8.1 - 9.9) | 8.8 (8.3 - 9.3) | 8.8 (8.6 - 8.9) | 7.5 (5.3 - 9.7) | 8.5 (7.4 - 9.6) | 7.5 (7.3 - 7.8) |
| MNC | 0.0 (-0.0 - 0.0) | 0.0 (-0.0 - 0.0) | 0.0 (0.0 - 0.0) | 0.1 (0.0 - 0.2) | 0.0 (-0.0 - 0.0) | 0.0 (0.0 - 0.0) |  | 0.0 (-0.0 - 0.1) | 0.1 (0.1 - 0.1) |
| Mental/Neuro | 4.0 (3.7 - 4.4) | 4.2 (3.8 - 4.6) | 3.8 (3.8 - 3.9) | 5.4 (4.7 - 6.1) | 4.8 (4.4 - 5.2) | 4.5 (4.4 - 4.5) | 6.6 (4.5 - 8.6) | 5.0 (4.2 - 5.8) | 4.8 (4.6 - 5.1) |
| Other | 7.3 (6.8 - 7.8) | 20.4 (19.6 - 21.2) | 14.7 (14.6 - 14.8) | 9.5 (8.6 - 10.5) | 21.2 (20.4 - 22.0) | 16.8 (16.6 - 16.9) | 10.0 (7.5 - 12.5) | 23.1 (21.5 - 24.7) | 19.5 (19.1 - 19.9) |
| Respiratory | 13.4 (12.8 - 14.1) | 10.0 (9.4 - 10.6) | 10.1 (10.0 - 10.2) | 10.7 (9.7 - 11.6) | 8.4 (7.9 - 8.9) | 8.7 (8.6 - 8.8) | 11.9 (9.3 - 14.6) | 8.9 (7.8 - 10.0) | 9.0 (8.7 - 9.3) |

Table S6: Relative distribution of garbage among all garbage codes by education, 1997 to 2023

| **Table S6. Proportions of garbage code by cause of death category, region, garbage code type and year** | | | | | | | |  |  |  |  |  |
| --- | --- | --- | --- | --- | --- | --- | --- | --- | --- | --- | --- | --- |
| **Garbage category** | **Skåne** | | | **Stockholm** | | | **Västra Götaland** | | | **Other** | | |
|  | **1997** | **2023** | **All years** | **1997** | **2023** | **All years** | **1997** | **2023** | **All years** | **1997** | **2023** | **All years** |
| Blood & Endo | 5.0 (4.3 - 5.8) | 5.2 (4.4 - 5.9) | 5.7 (5.6 - 5.9) | 3.7 (3.1 - 4.2) | 4.1 (3.5 - 4.6) | 4.8 (4.7 - 5.0) | 5.1 (4.2 - 5.9) | 4.8 (4.2 - 5.5) | 6.0 (5.8 - 6.1) | 5.5 (5.2 - 5.9) | 4.9 (4.6 - 5.3) | 6.1 (6.0 - 6.1) |
| CVD | 45.5 (43.8 - 47.1) | 29.2 (27.7 - 30.7) | 37.8 (37.5 - 38.1) | 43.6 (42.2 - 45.1) | 32.4 (31.0 - 33.8) | 38.1 (37.8 - 38.4) | 42.1 (40.1 - 44.1) | 31.6 (30.2 - 33.0) | 38.9 (38.6 - 39.1) | 46.7 (45.9 - 47.5) | 35.3 (34.5 - 36.2) | 41.5 (41.4 - 41.7) |
| Cancer | 12.0 (10.9 - 13.1) | 10.9 (9.8 - 11.9) | 11.6 (11.4 - 11.8) | 12.3 (11.4 - 13.3) | 9.4 (8.6 - 10.3) | 11.0 (10.8 - 11.1) | 12.5 (11.2 - 13.8) | 10.8 (9.9 - 11.8) | 11.9 (11.7 - 12.1) | 11.9 (11.4 - 12.4) | 11.4 (10.8 - 11.9) | 11.9 (11.8 - 12.0) |
| Digestive | 0.6 (0.4 - 0.9) | 1.5 (1.1 - 1.9) | 1.2 (1.2 - 1.3) | 1.0 (0.7 - 1.3) | 1.8 (1.4 - 2.2) | 1.2 (1.2 - 1.3) | 0.8 (0.4 - 1.2) | 1.2 (0.9 - 1.5) | 1.2 (1.1 - 1.2) | 0.8 (0.7 - 1.0) | 1.7 (1.5 - 1.9) | 1.2 (1.2 - 1.3) |
| Genitourinary | 1.3 (0.9 - 1.7) | 1.7 (1.2 - 2.1) | 1.7 (1.6 - 1.8) | 1.8 (1.4 - 2.2) | 1.3 (1.0 - 1.7) | 1.4 (1.3 - 1.5) | 1.4 (0.9 - 1.9) | 1.6 (1.2 - 2.0) | 1.7 (1.6 - 1.8) | 1.7 (1.5 - 1.9) | 1.5 (1.3 - 1.8) | 1.6 (1.6 - 1.7) |
| Infectious | 0.9 (0.6 - 1.3) | 5.7 (4.9 - 6.5) | 2.5 (2.4 - 2.6) | 1.3 (0.9 - 1.6) | 2.9 (2.4 - 3.4) | 1.9 (1.8 - 2.0) | 1.3 (0.8 - 1.8) | 4.2 (3.6 - 4.8) | 2.3 (2.2 - 2.4) | 1.1 (0.9 - 1.3) | 4.9 (4.5 - 5.3) | 2.6 (2.5 - 2.6) |
| Inj | 5.0 (4.3 - 5.8) | 6.8 (5.9 - 7.6) | 6.5 (6.3 - 6.6) | 6.3 (5.6 - 7.0) | 8.3 (7.4 - 9.1) | 7.9 (7.8 - 8.1) | 5.4 (4.5 - 6.4) | 8.6 (7.7 - 9.5) | 6.7 (6.6 - 6.9) | 5.1 (4.7 - 5.4) | 8.2 (7.7 - 8.7) | 6.4 (6.3 - 6.5) |
| MNC |  | 0.0 (-0.0 - 0.1) | 0.0 (0.0 - 0.0) | 0.0 (-0.0 - 0.1) | 0.0 (-0.0 - 0.1) | 0.0 (0.0 - 0.0) | 0.0 (-0.0 - 0.1) | 0.0 (-0.0 - 0.1) | 0.0 (0.0 - 0.0) | 0.0 (0.0 - 0.1) | 0.0 (-0.0 - 0.0) | 0.0 (0.0 - 0.0) |
| Mental/Neuro | 3.4 (2.8 - 4.1) | 4.6 (3.9 - 5.3) | 3.7 (3.6 - 3.8) | 3.8 (3.2 - 4.3) | 3.7 (3.1 - 4.3) | 4.0 (3.9 - 4.1) | 3.8 (3.0 - 4.5) | 4.8 (4.1 - 5.4) | 4.4 (4.2 - 4.5) | 3.4 (3.1 - 3.7) | 5.0 (4.6 - 5.4) | 4.0 (3.9 - 4.1) |
| Overall | 11.4 (10.4 - 12.5) | 24.1 (22.7 - 25.5) | 18.9 (18.6 - 19.1) | 12.0 (11.0 - 12.9) | 27.5 (26.1 - 28.8) | 20.0 (19.7 - 20.2) | 12.3 (11.0 - 13.6) | 22.6 (21.3 - 23.9) | 16.4 (16.2 - 16.7) | 9.3 (8.8 - 9.8) | 18.4 (17.7 - 19.1) | 14.7 (14.6 - 14.8) |
| Respiratory | 14.7 (13.5 - 15.9) | 10.4 (9.4 - 11.4) | 10.3 (10.2 - 10.5) | 14.2 (13.2 - 15.2) | 8.6 (7.8 - 9.5) | 9.6 (9.4 - 9.8) | 15.2 (13.8 - 16.6) | 9.7 (8.8 - 10.6) | 10.5 (10.3 - 10.7) | 14.5 (13.9 - 15.1) | 8.7 (8.2 - 9.2) | 9.9 (9.8 - 10.0) |

Table S7: Garbage level by age, education, and disease category, 1997 and 2023

| Table S7: Garbage level by age, education, and disease category, 1997 and 2023 | | | | | |
| --- | --- | --- | --- | --- | --- |
| **Year** | **GC** | **Education level** | **Age** | **Garbage Level (percent)** | **Rate per 100k** |
| 1997 | Abdomen and Pelvis Cancer | high | 40-69 | 0.6 (-0.6 - 1.8) | 0.2 (-0.2 - 0.6) |
| 1997 | Abdomen and Pelvis Cancer | high | 70-79 | 1.6 (-0.2 - 3.5) | 8.5 (-1.1 - 18.1) |
| 1997 | Abdomen and Pelvis Cancer | high | 80-89 | 1.6 (-0.2 - 3.3) | 32.5 (-4.3 - 69.3) |
| 1997 | Abdomen and Pelvis Cancer | intermediate | under 40 | 0.5 (-0.5 - 1.5) | 0.0 (-0.0 - 0.1) |
| 1997 | Abdomen and Pelvis Cancer | intermediate | 40-69 | 1.6 (0.8 - 2.4) | 1.1 (0.6 - 1.6) |
| 1997 | Abdomen and Pelvis Cancer | intermediate | 70-79 | 1.6 (0.9 - 2.2) | 9.3 (5.2 - 13.4) |
| 1997 | Abdomen and Pelvis Cancer | intermediate | 80-89 | 0.6 (0.2 - 1.0) | 13.4 (4.1 - 22.7) |
| 1997 | Abdomen and Pelvis Cancer | low | 40-69 | 1.1 (0.5 - 1.6) | 1.5 (0.7 - 2.2) |
| 1997 | Abdomen and Pelvis Cancer | low | 70-79 | 1.1 (0.8 - 1.5) | 9.0 (6.2 - 11.8) |
| 1997 | Abdomen and Pelvis Cancer | low | 80-89 | 0.9 (0.6 - 1.1) | 24.3 (17.4 - 31.2) |
| 1997 | Acute Respiratory Failure | intermediate | 40-69 | 0.2 (-0.1 - 0.4) | 0.1 (-0.0 - 0.3) |
| 1997 | Acute Respiratory Failure | intermediate | 70-79 | 0.2 (-0.1 - 0.4) | 0.9 (-0.4 - 2.2) |
| 1997 | Acute Respiratory Failure | intermediate | 80-89 | 0.1 (-0.1 - 0.2) | 1.7 (-1.6 - 5.0) |
| 1997 | Acute Respiratory Failure | low | 40-69 | 0.1 (-0.1 - 0.3) | 0.2 (-0.1 - 0.5) |
| 1997 | Acute Respiratory Failure | low | 70-79 | 0.1 (-0.0 - 0.1) | 0.5 (-0.2 - 1.1) |
| 1997 | Acute Respiratory Failure | low | 80-89 | 0.1 (0.0 - 0.2) | 2.5 (0.3 - 4.8) |
| 1997 | Acute kidney failure | high | 40-69 | 0.6 (-0.6 - 1.8) | 0.2 (-0.2 - 0.6) |
| 1997 | Acute kidney failure | high | 70-79 | 1.6 (-0.2 - 3.5) | 8.5 (-1.1 - 18.1) |
| 1997 | Acute kidney failure | high | 80-89 | 1.6 (-0.2 - 3.3) | 32.5 (-4.3 - 69.3) |
| 1997 | Acute kidney failure | intermediate | under 40 | 0.5 (-0.5 - 1.5) | 0.0 (-0.0 - 0.1) |
| 1997 | Acute kidney failure | intermediate | 40-69 | 0.8 (0.3 - 1.4) | 0.6 (0.2 - 0.9) |
| 1997 | Acute kidney failure | intermediate | 70-79 | 0.9 (0.4 - 1.4) | 5.1 (2.1 - 8.2) |
| 1997 | Acute kidney failure | intermediate | 80-89 | 1.5 (0.8 - 2.1) | 33.6 (18.9 - 48.3) |
| 1997 | Acute kidney failure | low | under 40 | 0.9 (-0.8 - 2.6) | 0.3 (-0.2 - 0.8) |
| 1997 | Acute kidney failure | low | 40-69 | 0.9 (0.4 - 1.4) | 1.3 (0.6 - 1.9) |
| 1997 | Acute kidney failure | low | 70-79 | 1.9 (1.4 - 2.3) | 14.6 (11.1 - 18.2) |
| 1997 | Acute kidney failure | low | 80-89 | 1.6 (1.2 - 1.9) | 44.1 (34.8 - 53.3) |
| 1997 | Adrenal Site Cancer unspecified part of adrenal gland | intermediate | under 40 | 0.5 (-0.5 - 1.5) | 0.0 (-0.0 - 0.1) |
| 1997 | Adrenal Site Cancer unspecified part of adrenal gland | intermediate | 40-69 | 0.4 (0.0 - 0.7) | 0.2 (0.0 - 0.5) |
| 1997 | Adrenal Site Cancer unspecified part of adrenal gland | intermediate | 70-79 | 0.1 (-0.1 - 0.2) | 0.5 (-0.4 - 1.4) |
| 1997 | Adrenal Site Cancer unspecified part of adrenal gland | low | 40-69 | 0.1 (-0.1 - 0.2) | 0.1 (-0.1 - 0.3) |
| 1997 | Adrenal Site Cancer unspecified part of adrenal gland | low | 80-89 | 0.0 (-0.0 - 0.1) | 0.5 (-0.5 - 1.5) |
| 1997 | Adrenal Unspecified Site Cancer in medulla or cortex | intermediate | 40-69 | 0.3 (-0.0 - 0.6) | 0.2 (-0.0 - 0.4) |
| 1997 | Adrenal Unspecified Site Cancer in medulla or cortex | low | 70-79 | 0.0 (-0.0 - 0.1) | 0.2 (-0.2 - 0.7) |
| 1997 | Adrenal Unspecified Site Cancer-parent cause | intermediate | 40-69 | 0.1 (-0.1 - 0.3) | 0.1 (-0.1 - 0.2) |
| 1997 | Adrenal Unspecified Site Cancer-parent cause | intermediate | 70-79 | 0.1 (-0.1 - 0.2) | 0.5 (-0.4 - 1.4) |
| 1997 | Adrenal Unspecified Site Cancer-parent cause | low | 40-69 | 0.2 (-0.0 - 0.5) | 0.3 (-0.0 - 0.6) |
| 1997 | Alcoholic hepatic failure | intermediate | under 40 | 1.0 (-0.4 - 2.3) | 0.1 (-0.0 - 0.2) |
| 1997 | Alcoholic hepatic failure | intermediate | 40-69 | 0.9 (0.4 - 1.5) | 0.6 (0.2 - 1.0) |
| 1997 | Alcoholic hepatic failure | intermediate | 70-79 | 0.1 (-0.1 - 0.2) | 0.5 (-0.4 - 1.4) |
| 1997 | Alcoholic hepatic failure | low | 40-69 | 0.9 (0.4 - 1.4) | 1.3 (0.6 - 1.9) |
| 1997 | Alcoholic hepatic failure | low | 70-79 | 0.2 (0.1 - 0.3) | 1.6 (0.4 - 2.7) |
| 1997 | Alcoholic hepatic failure | low | 80-89 | 0.0 (-0.0 - 0.1) | 0.5 (-0.5 - 1.5) |
| 1997 | All, Ill Defined code for causes of death | high | under 40 | 22.7 (5.2 - 40.2) | 0.4 (0.0 - 0.7) |
| 1997 | All, Ill Defined code for causes of death | high | 40-69 | 12.2 (7.2 - 17.2) | 4.4 (2.5 - 6.3) |
| 1997 | All, Ill Defined code for causes of death | high | 70-79 | 3.8 (1.0 - 6.6) | 19.8 (5.1 - 34.5) |
| 1997 | All, Ill Defined code for causes of death | high | 80-89 | 3.6 (1.0 - 6.3) | 75.9 (19.7 - 132.1) |
| 1997 | All, Ill Defined code for causes of death | intermediate | under 40 | 11.3 (7.0 - 15.7) | 0.8 (0.5 - 1.2) |
| 1997 | All, Ill Defined code for causes of death | intermediate | 40-69 | 10.0 (8.2 - 11.8) | 6.6 (5.3 - 7.8) |
| 1997 | All, Ill Defined code for causes of death | intermediate | 70-79 | 4.6 (3.5 - 5.8) | 27.5 (20.5 - 34.5) |
| 1997 | All, Ill Defined code for causes of death | intermediate | 80-89 | 2.4 (1.6 - 3.2) | 53.7 (35.1 - 72.3) |
| 1997 | All, Ill Defined code for causes of death | low | under 40 | 7.8 (2.9 - 12.7) | 2.3 (0.8 - 3.8) |
| 1997 | All, Ill Defined code for causes of death | low | 40-69 | 6.7 (5.4 - 8.0) | 9.0 (7.2 - 10.9) |
| 1997 | All, Ill Defined code for causes of death | low | 70-79 | 3.1 (2.5 - 3.7) | 24.3 (19.7 - 28.9) |
| 1997 | All, Ill Defined code for causes of death | low | 80-89 | 2.3 (1.9 - 2.7) | 63.8 (52.7 - 75.0) |
| 1997 | Amyloidosis | intermediate | 40-69 | 0.5 (0.1 - 0.9) | 0.3 (0.0 - 0.6) |
| 1997 | Amyloidosis | intermediate | 70-79 | 0.3 (0.0 - 0.6) | 1.9 (0.0 - 3.7) |
| 1997 | Amyloidosis | low | under 40 | 0.9 (-0.8 - 2.6) | 0.3 (-0.2 - 0.8) |
| 1997 | Amyloidosis | low | 40-69 | 0.6 (0.2 - 1.1) | 0.9 (0.3 - 1.4) |
| 1997 | Amyloidosis | low | 70-79 | 0.5 (0.3 - 0.7) | 3.8 (2.0 - 5.7) |
| 1997 | Amyloidosis | low | 80-89 | 0.1 (0.0 - 0.2) | 3.0 (0.6 - 5.5) |
| 1997 | Anemia Unspecified | intermediate | under 40 | 0.5 (-0.5 - 1.5) | 0.0 (-0.0 - 0.1) |
| 1997 | Anemia Unspecified | intermediate | 70-79 | 0.2 (-0.1 - 0.4) | 0.9 (-0.4 - 2.2) |
| 1997 | Anemia Unspecified | intermediate | 80-89 | 0.3 (0.0 - 0.6) | 6.7 (0.1 - 13.3) |
| 1997 | Anemia Unspecified | low | 70-79 | 0.1 (0.0 - 0.2) | 0.9 (0.0 - 1.8) |
| 1997 | Anemia Unspecified | low | 80-89 | 0.3 (0.2 - 0.5) | 8.6 (4.5 - 12.7) |
| 1997 | Arterial Embolism | high | 40-69 | 0.6 (-0.6 - 1.8) | 0.2 (-0.2 - 0.6) |
| 1997 | Arterial Embolism | high | 70-79 | 1.1 (-0.4 - 2.6) | 5.7 (-2.2 - 13.5) |
| 1997 | Arterial Embolism | high | 80-89 | 0.5 (-0.5 - 1.5) | 10.8 (-10.4 - 32.1) |
| 1997 | Arterial Embolism | intermediate | under 40 | 0.5 (-0.5 - 1.5) | 0.0 (-0.0 - 0.1) |
| 1997 | Arterial Embolism | intermediate | 40-69 | 0.3 (-0.0 - 0.6) | 0.2 (-0.0 - 0.4) |
| 1997 | Arterial Embolism | intermediate | 70-79 | 0.5 (0.1 - 0.8) | 2.8 (0.6 - 5.0) |
| 1997 | Arterial Embolism | intermediate | 80-89 | 0.4 (0.0 - 0.7) | 8.4 (1.0 - 15.7) |
| 1997 | Arterial Embolism | low | 40-69 | 0.4 (0.0 - 0.7) | 0.5 (0.1 - 0.9) |
| 1997 | Arterial Embolism | low | 70-79 | 0.6 (0.4 - 0.9) | 5.0 (2.9 - 7.0) |
| 1997 | Arterial Embolism | low | 80-89 | 0.6 (0.4 - 0.8) | 17.2 (11.4 - 23.0) |
| 1997 | Assault by unspecified means | intermediate | under 40 | 0.5 (-0.5 - 1.5) | 0.0 (-0.0 - 0.1) |
| 1997 | Assault by unspecified means | low | 70-79 | 0.1 (-0.0 - 0.1) | 0.5 (-0.2 - 1.1) |
| 1997 | Assigned death to tobacco | high | 70-79 | 0.5 (-0.5 - 1.6) | 2.8 (-2.7 - 8.4) |
| 1997 | Assigned death to tobacco | intermediate | 40-69 | 0.1 (-0.1 - 0.3) | 0.1 (-0.1 - 0.2) |
| 1997 | Assigned death to tobacco | low | 40-69 | 0.2 (-0.0 - 0.5) | 0.3 (-0.0 - 0.6) |
| 1997 | Assigned death to tobacco | low | 70-79 | 0.1 (-0.0 - 0.1) | 0.5 (-0.2 - 1.1) |
| 1997 | Assigned death to tobacco | low | 80-89 | 0.1 (-0.0 - 0.1) | 1.5 (-0.2 - 3.2) |
| 1997 | Atherosclerosis | high | 40-69 | 2.4 (0.1 - 4.8) | 0.9 (0.0 - 1.7) |
| 1997 | Atherosclerosis | high | 70-79 | 4.4 (1.4 - 7.3) | 22.7 (7.0 - 38.4) |
| 1997 | Atherosclerosis | high | 80-89 | 7.8 (4.0 - 11.6) | 162.6 (80.4 - 244.8) |
| 1997 | Atherosclerosis | intermediate | under 40 | 0.5 (-0.5 - 1.5) | 0.0 (-0.0 - 0.1) |
| 1997 | Atherosclerosis | intermediate | 40-69 | 3.1 (2.1 - 4.1) | 2.0 (1.3 - 2.7) |
| 1997 | Atherosclerosis | intermediate | 70-79 | 6.3 (4.9 - 7.6) | 37.3 (29.1 - 45.5) |
| 1997 | Atherosclerosis | intermediate | 80-89 | 9.6 (8.0 - 11.1) | 216.5 (179.2 - 253.8) |
| 1997 | Atherosclerosis | low | 40-69 | 3.7 (2.7 - 4.7) | 5.0 (3.7 - 6.4) |
| 1997 | Atherosclerosis | low | 70-79 | 6.8 (6.0 - 7.7) | 53.9 (47.0 - 60.7) |
| 1997 | Atherosclerosis | low | 80-89 | 7.8 (7.1 - 8.5) | 217.8 (197.3 - 238.4) |
| 1997 | CKD due to diabetes Unspecified type | high | 40-69 | 1.2 (-0.5 - 2.9) | 0.4 (-0.2 - 1.0) |
| 1997 | CKD due to diabetes Unspecified type | high | 80-89 | 1.0 (-0.4 - 2.5) | 21.7 (-8.4 - 51.7) |
| 1997 | CKD due to diabetes Unspecified type | intermediate | under 40 | 1.0 (-0.4 - 2.3) | 0.1 (-0.0 - 0.2) |
| 1997 | CKD due to diabetes Unspecified type | intermediate | 40-69 | 1.0 (0.4 - 1.6) | 0.7 (0.3 - 1.1) |
| 1997 | CKD due to diabetes Unspecified type | intermediate | 70-79 | 1.0 (0.5 - 1.6) | 6.1 (2.8 - 9.4) |
| 1997 | CKD due to diabetes Unspecified type | intermediate | 80-89 | 0.1 (-0.1 - 0.4) | 3.4 (-1.3 - 8.0) |
| 1997 | CKD due to diabetes Unspecified type | low | under 40 | 1.7 (-0.7 - 4.1) | 0.5 (-0.2 - 1.2) |
| 1997 | CKD due to diabetes Unspecified type | low | 40-69 | 1.7 (1.0 - 2.3) | 2.2 (1.3 - 3.1) |
| 1997 | CKD due to diabetes Unspecified type | low | 70-79 | 1.0 (0.7 - 1.4) | 8.1 (5.5 - 10.8) |
| 1997 | CKD due to diabetes Unspecified type | low | 80-89 | 0.4 (0.2 - 0.5) | 10.1 (5.7 - 14.6) |
| 1997 | CNS Abscess | intermediate | 40-69 | 0.2 (-0.1 - 0.4) | 0.1 (-0.0 - 0.3) |
| 1997 | CNS Abscess | intermediate | 70-79 | 0.1 (-0.1 - 0.2) | 0.5 (-0.4 - 1.4) |
| 1997 | CNS Abscess | low | 40-69 | 0.1 (-0.1 - 0.2) | 0.1 (-0.1 - 0.3) |
| 1997 | CNS Abscess | low | 70-79 | 0.0 (-0.0 - 0.1) | 0.2 (-0.2 - 0.7) |
| 1997 | CNS Abscess | low | 80-89 | 0.0 (-0.0 - 0.1) | 0.5 (-0.5 - 1.5) |
| 1997 | Cardiac rhythm disorders | high | 70-79 | 1.6 (-0.2 - 3.5) | 8.5 (-1.1 - 18.1) |
| 1997 | Cardiac rhythm disorders | high | 80-89 | 0.5 (-0.5 - 1.5) | 10.8 (-10.4 - 32.1) |
| 1997 | Cardiac rhythm disorders | intermediate | under 40 | 0.5 (-0.5 - 1.5) | 0.0 (-0.0 - 0.1) |
| 1997 | Cardiac rhythm disorders | intermediate | 40-69 | 0.9 (0.4 - 1.5) | 0.6 (0.2 - 1.0) |
| 1997 | Cardiac rhythm disorders | intermediate | 70-79 | 0.5 (0.1 - 0.8) | 2.8 (0.6 - 5.0) |
| 1997 | Cardiac rhythm disorders | intermediate | 80-89 | 0.7 (0.2 - 1.1) | 15.1 (5.2 - 25.0) |
| 1997 | Cardiac rhythm disorders | low | under 40 | 1.7 (-0.7 - 4.1) | 0.5 (-0.2 - 1.2) |
| 1997 | Cardiac rhythm disorders | low | 40-69 | 0.7 (0.3 - 1.2) | 1.0 (0.4 - 1.6) |
| 1997 | Cardiac rhythm disorders | low | 70-79 | 0.4 (0.2 - 0.6) | 3.4 (1.7 - 5.1) |
| 1997 | Cardiac rhythm disorders | low | 80-89 | 0.5 (0.3 - 0.7) | 14.2 (8.9 - 19.4) |
| 1997 | Cerebral Palsy | high | 40-69 | 0.6 (-0.6 - 1.8) | 0.2 (-0.2 - 0.6) |
| 1997 | Cerebral Palsy | low | under 40 | 2.6 (-0.3 - 5.5) | 0.8 (-0.1 - 1.6) |
| 1997 | Cerebral Palsy | low | 40-69 | 0.3 (0.0 - 0.6) | 0.4 (0.0 - 0.8) |
| 1997 | Cerebral Palsy | low | 70-79 | 0.0 (-0.0 - 0.1) | 0.2 (-0.2 - 0.7) |
| 1997 | Chronic lymphocytic leukemia by age | high | 40-69 | 1.2 (-0.5 - 2.9) | 0.4 (-0.2 - 1.0) |
| 1997 | Chronic lymphocytic leukemia by age | high | 70-79 | 1.1 (-0.4 - 2.6) | 5.7 (-2.2 - 13.5) |
| 1997 | Chronic lymphocytic leukemia by age | high | 80-89 | 2.1 (0.1 - 4.1) | 43.4 (0.9 - 85.8) |
| 1997 | Chronic lymphocytic leukemia by age | intermediate | 40-69 | 0.7 (0.2 - 1.1) | 0.4 (0.1 - 0.8) |
| 1997 | Chronic lymphocytic leukemia by age | intermediate | 70-79 | 0.8 (0.3 - 1.3) | 4.7 (1.8 - 7.6) |
| 1997 | Chronic lymphocytic leukemia by age | intermediate | 80-89 | 1.0 (0.4 - 1.5) | 21.8 (10.0 - 33.7) |
| 1997 | Chronic lymphocytic leukemia by age | low | 40-69 | 1.4 (0.8 - 2.0) | 1.8 (1.0 - 2.7) |
| 1997 | Chronic lymphocytic leukemia by age | low | 70-79 | 1.4 (1.0 - 1.8) | 11.3 (8.1 - 14.4) |
| 1997 | Chronic lymphocytic leukemia by age | low | 80-89 | 0.7 (0.4 - 0.9) | 18.2 (12.3 - 24.2) |
| 1997 | Chronic respiratory failure | low | 80-89 | 0.0 (-0.0 - 0.1) | 0.5 (-0.5 - 1.5) |
| 1997 | Diabetes unspecified type | high | 40-69 | 6.7 (2.9 - 10.5) | 2.4 (1.0 - 3.8) |
| 1997 | Diabetes unspecified type | high | 70-79 | 4.4 (1.4 - 7.3) | 22.7 (7.0 - 38.4) |
| 1997 | Diabetes unspecified type | high | 80-89 | 0.5 (-0.5 - 1.5) | 10.8 (-10.4 - 32.1) |
| 1997 | Diabetes unspecified type | intermediate | under 40 | 3.0 (0.6 - 5.3) | 0.2 (0.0 - 0.4) |
| 1997 | Diabetes unspecified type | intermediate | 40-69 | 6.6 (5.1 - 8.1) | 4.3 (3.3 - 5.4) |
| 1997 | Diabetes unspecified type | intermediate | 70-79 | 5.6 (4.3 - 6.8) | 33.1 (25.4 - 40.8) |
| 1997 | Diabetes unspecified type | intermediate | 80-89 | 3.0 (2.1 - 4.0) | 68.8 (47.8 - 89.9) |
| 1997 | Diabetes unspecified type | low | under 40 | 6.1 (1.7 - 10.5) | 1.8 (0.5 - 3.1) |
| 1997 | Diabetes unspecified type | low | 40-69 | 10.7 (9.1 - 12.3) | 14.4 (12.1 - 16.8) |
| 1997 | Diabetes unspecified type | low | 70-79 | 6.9 (6.1 - 7.8) | 54.5 (47.7 - 61.4) |
| 1997 | Diabetes unspecified type | low | 80-89 | 4.0 (3.5 - 4.6) | 113.0 (98.1 - 127.8) |
| 1997 | Exposure to unspecified factor X59 | high | 40-69 | 1.2 (-0.5 - 2.9) | 0.4 (-0.2 - 1.0) |
| 1997 | Exposure to unspecified factor X59 | high | 70-79 | 2.2 (0.1 - 4.3) | 11.3 (0.2 - 22.4) |
| 1997 | Exposure to unspecified factor X59 | high | 80-89 | 1.0 (-0.4 - 2.5) | 21.7 (-8.4 - 51.7) |
| 1997 | Exposure to unspecified factor X59 | intermediate | under 40 | 1.5 (-0.2 - 3.1) | 0.1 (-0.0 - 0.2) |
| 1997 | Exposure to unspecified factor X59 | intermediate | 40-69 | 0.5 (0.1 - 0.9) | 0.3 (0.0 - 0.6) |
| 1997 | Exposure to unspecified factor X59 | intermediate | 70-79 | 2.9 (2.0 - 3.8) | 17.3 (11.7 - 22.8) |
| 1997 | Exposure to unspecified factor X59 | intermediate | 80-89 | 3.1 (2.2 - 4.0) | 70.5 (49.2 - 91.8) |
| 1997 | Exposure to unspecified factor X59 | low | under 40 | 1.7 (-0.7 - 4.1) | 0.5 (-0.2 - 1.2) |
| 1997 | Exposure to unspecified factor X59 | low | 40-69 | 0.9 (0.4 - 1.3) | 1.2 (0.5 - 1.8) |
| 1997 | Exposure to unspecified factor X59 | low | 70-79 | 2.7 (2.1 - 3.2) | 21.0 (16.7 - 25.2) |
| 1997 | Exposure to unspecified factor X59 | low | 80-89 | 3.5 (3.0 - 4.0) | 98.8 (84.9 - 112.6) |
| 1997 | External Causes UDI, type unspecified | high | 70-79 | 1.1 (-0.4 - 2.6) | 5.7 (-2.2 - 13.5) |
| 1997 | External Causes UDI, type unspecified | high | 80-89 | 1.6 (-0.2 - 3.3) | 32.5 (-4.3 - 69.3) |
| 1997 | External Causes UDI, type unspecified | intermediate | under 40 | 0.5 (-0.5 - 1.5) | 0.0 (-0.0 - 0.1) |
| 1997 | External Causes UDI, type unspecified | intermediate | 40-69 | 0.8 (0.2 - 1.3) | 0.5 (0.2 - 0.8) |
| 1997 | External Causes UDI, type unspecified | intermediate | 70-79 | 0.5 (0.1 - 0.8) | 2.8 (0.6 - 5.0) |
| 1997 | External Causes UDI, type unspecified | intermediate | 80-89 | 0.3 (0.0 - 0.6) | 6.7 (0.1 - 13.3) |
| 1997 | External Causes UDI, type unspecified | low | under 40 | 1.7 (-0.7 - 4.1) | 0.5 (-0.2 - 1.2) |
| 1997 | External Causes UDI, type unspecified | low | 40-69 | 0.4 (0.1 - 0.8) | 0.6 (0.1 - 1.0) |
| 1997 | External Causes UDI, type unspecified | low | 70-79 | 0.3 (0.1 - 0.5) | 2.5 (1.0 - 3.9) |
| 1997 | External Causes UDI, type unspecified | low | 80-89 | 0.3 (0.1 - 0.4) | 7.1 (3.4 - 10.8) |
| 1997 | Eye Unspecified Site Cancer | intermediate | 40-69 | 0.1 (-0.1 - 0.3) | 0.1 (-0.1 - 0.2) |
| 1997 | Eye Unspecified Site Cancer | low | 40-69 | 0.2 (-0.0 - 0.5) | 0.3 (-0.0 - 0.6) |
| 1997 | Eye Unspecified Site Cancer | low | 70-79 | 0.0 (-0.0 - 0.1) | 0.2 (-0.2 - 0.7) |
| 1997 | Female pelvic inflammatory diseases | high | 40-69 | 0.6 (-0.6 - 1.8) | 0.2 (-0.2 - 0.6) |
| 1997 | Female pelvic inflammatory diseases | low | 40-69 | 0.1 (-0.1 - 0.2) | 0.1 (-0.1 - 0.3) |
| 1997 | Female pelvic inflammatory diseases | low | 70-79 | 0.1 (-0.0 - 0.1) | 0.5 (-0.2 - 1.1) |
| 1997 | Female pelvic inflammatory diseases | low | 80-89 | 0.0 (-0.0 - 0.1) | 0.5 (-0.5 - 1.5) |
| 1997 | Fistula | low | 80-89 | 0.0 (-0.0 - 0.1) | 1.0 (-0.4 - 2.4) |
| 1997 | Fluid, Electrolyte, Acid Base Disorders | high | 80-89 | 1.0 (-0.4 - 2.5) | 21.7 (-8.4 - 51.7) |
| 1997 | Fluid, Electrolyte, Acid Base Disorders | intermediate | 40-69 | 0.1 (-0.1 - 0.3) | 0.1 (-0.1 - 0.2) |
| 1997 | Fluid, Electrolyte, Acid Base Disorders | intermediate | 70-79 | 0.3 (0.0 - 0.6) | 1.9 (0.0 - 3.7) |
| 1997 | Fluid, Electrolyte, Acid Base Disorders | intermediate | 80-89 | 0.1 (-0.1 - 0.4) | 3.4 (-1.3 - 8.0) |
| 1997 | Fluid, Electrolyte, Acid Base Disorders | low | 40-69 | 0.1 (-0.1 - 0.3) | 0.2 (-0.1 - 0.5) |
| 1997 | Fluid, Electrolyte, Acid Base Disorders | low | 70-79 | 0.1 (-0.0 - 0.1) | 0.5 (-0.2 - 1.1) |
| 1997 | Fluid, Electrolyte, Acid Base Disorders | low | 80-89 | 0.1 (0.0 - 0.2) | 3.0 (0.6 - 5.5) |
| 1997 | HIV correction for Aspergillosis | intermediate | 80-89 | 0.1 (-0.1 - 0.2) | 1.7 (-1.6 - 5.0) |
| 1997 | HIV correction for Aspergillosis | low | 70-79 | 0.1 (-0.0 - 0.1) | 0.5 (-0.2 - 1.1) |
| 1997 | HIV correction for Aspergillosis | low | 80-89 | 0.0 (-0.0 - 0.1) | 0.5 (-0.5 - 1.5) |
| 1997 | HIV correction for Candidiasis | high | 80-89 | 0.5 (-0.5 - 1.5) | 10.8 (-10.4 - 32.1) |
| 1997 | HIV correction for Candidiasis | intermediate | 40-69 | 0.1 (-0.1 - 0.3) | 0.1 (-0.1 - 0.2) |
| 1997 | HIV correction for Candidiasis | low | 70-79 | 0.0 (-0.0 - 0.1) | 0.2 (-0.2 - 0.7) |
| 1997 | HIV correction for Candidiasis | low | 80-89 | 0.0 (-0.0 - 0.1) | 0.5 (-0.5 - 1.5) |
| 1997 | HIV correction for Immunodeficiency antibody | low | under 40 | 0.9 (-0.8 - 2.6) | 0.3 (-0.2 - 0.8) |
| 1997 | HIV correction for Immunodeficiency antibody | low | 70-79 | 0.1 (-0.0 - 0.1) | 0.5 (-0.2 - 1.1) |
| 1997 | HIV correction for Immunodeficiency cell | high | under 40 | 4.5 (-4.2 - 13.2) | 0.1 (-0.1 - 0.2) |
| 1997 | HIV correction for Immunodeficiency other | high | 70-79 | 0.5 (-0.5 - 1.6) | 2.8 (-2.7 - 8.4) |
| 1997 | HIV correction for Immunodeficiency other | intermediate | under 40 | 0.5 (-0.5 - 1.5) | 0.0 (-0.0 - 0.1) |
| 1997 | HIV correction for Immunodeficiency other | intermediate | 80-89 | 0.1 (-0.1 - 0.2) | 1.7 (-1.6 - 5.0) |
| 1997 | HIV correction for Immunodeficiency other | low | 40-69 | 0.1 (-0.1 - 0.2) | 0.1 (-0.1 - 0.3) |
| 1997 | HIV correction for Immunodeficiency other | low | 80-89 | 0.0 (-0.0 - 0.1) | 1.0 (-0.4 - 2.4) |
| 1997 | HIV correction for Kaposi's sarcoma | low | 70-79 | 0.0 (-0.0 - 0.1) | 0.2 (-0.2 - 0.7) |
| 1997 | HIV correction for Kaposi's sarcoma | low | 80-89 | 0.0 (-0.0 - 0.1) | 0.5 (-0.5 - 1.5) |
| 1997 | HIV correction for Other Mycobacterial infection | intermediate | 70-79 | 0.1 (-0.1 - 0.2) | 0.5 (-0.4 - 1.4) |
| 1997 | HIV correction for Other Mycobacterial infection | low | 70-79 | 0.1 (0.0 - 0.3) | 1.1 (0.1 - 2.1) |
| 1997 | HIV correction for Pneumocystosis | intermediate | 40-69 | 0.1 (-0.1 - 0.3) | 0.1 (-0.1 - 0.2) |
| 1997 | HIV correction for Unspecified mycosis | intermediate | 70-79 | 0.1 (-0.1 - 0.2) | 0.5 (-0.4 - 1.4) |
| 1997 | HIV correction for Unspecified mycosis | low | under 40 | 0.9 (-0.8 - 2.6) | 0.3 (-0.2 - 0.8) |
| 1997 | HIV correction for Unspecified mycosis | low | 80-89 | 0.0 (-0.0 - 0.1) | 1.0 (-0.4 - 2.4) |
| 1997 | Head and Neck Cancer | intermediate | under 40 | 0.5 (-0.5 - 1.5) | 0.0 (-0.0 - 0.1) |
| 1997 | Head and Neck Cancer | intermediate | 40-69 | 0.3 (-0.0 - 0.6) | 0.2 (-0.0 - 0.4) |
| 1997 | Head and Neck Cancer | intermediate | 70-79 | 0.1 (-0.1 - 0.2) | 0.5 (-0.4 - 1.4) |
| 1997 | Head and Neck Cancer | low | 40-69 | 0.1 (-0.1 - 0.3) | 0.2 (-0.1 - 0.5) |
| 1997 | Head and Neck Cancer | low | 70-79 | 0.1 (0.0 - 0.3) | 1.1 (0.1 - 2.1) |
| 1997 | Head and Neck Cancer | low | 80-89 | 0.1 (0.0 - 0.1) | 2.0 (0.0 - 4.0) |
| 1997 | Heart failure unspecified right or left | high | 40-69 | 0.6 (-0.6 - 1.8) | 0.2 (-0.2 - 0.6) |
| 1997 | Heart failure unspecified right or left | high | 70-79 | 4.9 (1.8 - 8.1) | 25.5 (8.8 - 42.2) |
| 1997 | Heart failure unspecified right or left | high | 80-89 | 7.8 (4.0 - 11.6) | 162.6 (80.4 - 244.8) |
| 1997 | Heart failure unspecified right or left | intermediate | under 40 | 0.5 (-0.5 - 1.5) | 0.0 (-0.0 - 0.1) |
| 1997 | Heart failure unspecified right or left | intermediate | 40-69 | 0.8 (0.2 - 1.3) | 0.5 (0.2 - 0.8) |
| 1997 | Heart failure unspecified right or left | intermediate | 70-79 | 6.4 (5.1 - 7.8) | 38.2 (30.0 - 46.5) |
| 1997 | Heart failure unspecified right or left | intermediate | 80-89 | 9.7 (8.1 - 11.3) | 219.9 (182.3 - 257.5) |
| 1997 | Heart failure unspecified right or left | low | under 40 | 3.5 (0.1 - 6.8) | 1.0 (0.0 - 2.0) |
| 1997 | Heart failure unspecified right or left | low | 40-69 | 1.7 (1.0 - 2.4) | 2.3 (1.4 - 3.3) |
| 1997 | Heart failure unspecified right or left | low | 70-79 | 6.6 (5.8 - 7.4) | 52.1 (45.3 - 58.8) |
| 1997 | Heart failure unspecified right or left | low | 80-89 | 10.6 (9.8 - 11.4) | 296.9 (272.9 - 320.9) |
| 1997 | Hepatic Failure | high | 40-69 | 1.8 (-0.2 - 3.9) | 0.7 (-0.1 - 1.4) |
| 1997 | Hepatic Failure | high | 70-79 | 0.5 (-0.5 - 1.6) | 2.8 (-2.7 - 8.4) |
| 1997 | Hepatic Failure | intermediate | 40-69 | 0.8 (0.3 - 1.4) | 0.6 (0.2 - 0.9) |
| 1997 | Hepatic Failure | intermediate | 70-79 | 0.2 (-0.0 - 0.5) | 1.4 (-0.2 - 3.0) |
| 1997 | Hepatic Failure | intermediate | 80-89 | 0.1 (-0.1 - 0.4) | 3.4 (-1.3 - 8.0) |
| 1997 | Hepatic Failure | low | 40-69 | 1.1 (0.5 - 1.6) | 1.5 (0.7 - 2.2) |
| 1997 | Hepatic Failure | low | 70-79 | 0.6 (0.3 - 0.8) | 4.5 (2.5 - 6.5) |
| 1997 | Hepatic Failure | low | 80-89 | 0.1 (0.0 - 0.2) | 4.1 (1.2 - 6.9) |
| 1997 | Hepatitis Unspecified | high | 40-69 | 0.6 (-0.6 - 1.8) | 0.2 (-0.2 - 0.6) |
| 1997 | Hepatitis Unspecified | intermediate | 40-69 | 0.7 (0.2 - 1.1) | 0.4 (0.1 - 0.8) |
| 1997 | Hepatitis Unspecified | low | under 40 | 0.9 (-0.8 - 2.6) | 0.3 (-0.2 - 0.8) |
| 1997 | Hepatitis Unspecified | low | 40-69 | 0.5 (0.1 - 0.9) | 0.7 (0.2 - 1.2) |
| 1997 | Hypertension | high | 40-69 | 0.6 (-0.6 - 1.8) | 0.2 (-0.2 - 0.6) |
| 1997 | Hypertension | high | 80-89 | 0.5 (-0.5 - 1.5) | 10.8 (-10.4 - 32.1) |
| 1997 | Hypertension | intermediate | 40-69 | 0.2 (-0.1 - 0.4) | 0.1 (-0.0 - 0.3) |
| 1997 | Hypertension | intermediate | 70-79 | 1.3 (0.6 - 1.9) | 7.5 (3.8 - 11.1) |
| 1997 | Hypertension | intermediate | 80-89 | 0.7 (0.2 - 1.1) | 15.1 (5.2 - 25.0) |
| 1997 | Hypertension | low | 40-69 | 1.1 (0.5 - 1.6) | 1.5 (0.7 - 2.2) |
| 1997 | Hypertension | low | 70-79 | 0.9 (0.6 - 1.2) | 6.8 (4.3 - 9.2) |
| 1997 | Hypertension | low | 80-89 | 0.9 (0.6 - 1.1) | 23.8 (17.0 - 30.6) |
| 1997 | Intermediate cause for CNS | high | 40-69 | 1.2 (-0.5 - 2.9) | 0.4 (-0.2 - 1.0) |
| 1997 | Intermediate cause for CNS | high | 70-79 | 0.5 (-0.5 - 1.6) | 2.8 (-2.7 - 8.4) |
| 1997 | Intermediate cause for CNS | intermediate | 40-69 | 0.4 (0.0 - 0.7) | 0.2 (0.0 - 0.5) |
| 1997 | Intermediate cause for CNS | intermediate | 70-79 | 0.4 (0.0 - 0.7) | 2.3 (0.3 - 4.4) |
| 1997 | Intermediate cause for CNS | intermediate | 80-89 | 0.4 (0.0 - 0.7) | 8.4 (1.0 - 15.7) |
| 1997 | Intermediate cause for CNS | low | under 40 | 0.9 (-0.8 - 2.6) | 0.3 (-0.2 - 0.8) |
| 1997 | Intermediate cause for CNS | low | 40-69 | 0.7 (0.3 - 1.2) | 1.0 (0.4 - 1.6) |
| 1997 | Intermediate cause for CNS | low | 70-79 | 0.3 (0.1 - 0.5) | 2.7 (1.2 - 4.2) |
| 1997 | Intermediate cause for CNS | low | 80-89 | 0.1 (0.0 - 0.1) | 2.0 (0.0 - 4.0) |
| 1997 | Left heart failure | high | 40-69 | 0.6 (-0.6 - 1.8) | 0.2 (-0.2 - 0.6) |
| 1997 | Left heart failure | high | 70-79 | 1.1 (-0.4 - 2.6) | 5.7 (-2.2 - 13.5) |
| 1997 | Left heart failure | intermediate | 40-69 | 0.2 (-0.1 - 0.4) | 0.1 (-0.0 - 0.3) |
| 1997 | Left heart failure | intermediate | 70-79 | 1.1 (0.5 - 1.7) | 6.5 (3.1 - 9.9) |
| 1997 | Left heart failure | intermediate | 80-89 | 2.7 (1.8 - 3.5) | 60.4 (40.7 - 80.2) |
| 1997 | Left heart failure | low | 40-69 | 0.3 (0.0 - 0.6) | 0.4 (0.0 - 0.8) |
| 1997 | Left heart failure | low | 70-79 | 1.1 (0.7 - 1.4) | 8.6 (5.8 - 11.3) |
| 1997 | Left heart failure | low | 80-89 | 1.5 (1.2 - 1.8) | 41.5 (32.5 - 50.5) |
| 1997 | Liver Abscess | low | 70-79 | 0.0 (-0.0 - 0.1) | 0.2 (-0.2 - 0.7) |
| 1997 | Lymphoid leukemia unspecified by age | high | under 40 | 4.5 (-4.2 - 13.2) | 0.1 (-0.1 - 0.2) |
| 1997 | Lymphoid leukemia unspecified by age | intermediate | 40-69 | 0.1 (-0.1 - 0.3) | 0.1 (-0.1 - 0.2) |
| 1997 | Lymphoid leukemia unspecified by age | intermediate | 70-79 | 0.2 (-0.1 - 0.4) | 0.9 (-0.4 - 2.2) |
| 1997 | Lymphoid leukemia unspecified by age | low | 40-69 | 0.1 (-0.1 - 0.3) | 0.2 (-0.1 - 0.5) |
| 1997 | Lymphoid leukemia unspecified by age | low | 70-79 | 0.2 (0.0 - 0.3) | 1.4 (0.3 - 2.4) |
| 1997 | Lymphoid leukemia unspecified by age | low | 80-89 | 0.1 (-0.0 - 0.1) | 1.5 (-0.2 - 3.2) |
| 1997 | Mental Disorders | high | under 40 | 9.1 (-2.9 - 21.1) | 0.1 (-0.1 - 0.4) |
| 1997 | Mental Disorders | high | 70-79 | 0.5 (-0.5 - 1.6) | 2.8 (-2.7 - 8.4) |
| 1997 | Mental Disorders | intermediate | 40-69 | 0.6 (0.1 - 1.0) | 0.4 (0.1 - 0.7) |
| 1997 | Mental Disorders | intermediate | 70-79 | 0.5 (0.1 - 0.8) | 2.8 (0.6 - 5.0) |
| 1997 | Mental Disorders | intermediate | 80-89 | 0.4 (0.1 - 0.8) | 10.1 (2.0 - 18.1) |
| 1997 | Mental Disorders | low | under 40 | 0.9 (-0.8 - 2.6) | 0.3 (-0.2 - 0.8) |
| 1997 | Mental Disorders | low | 40-69 | 0.6 (0.2 - 1.0) | 0.8 (0.2 - 1.3) |
| 1997 | Mental Disorders | low | 70-79 | 0.6 (0.3 - 0.8) | 4.5 (2.5 - 6.5) |
| 1997 | Mental Disorders | low | 80-89 | 0.5 (0.3 - 0.7) | 15.2 (9.8 - 20.6) |
| 1997 | Myocardial Degeneration | intermediate | 70-79 | 0.2 (-0.1 - 0.4) | 0.9 (-0.4 - 2.2) |
| 1997 | Myocardial Degeneration | intermediate | 80-89 | 0.1 (-0.1 - 0.2) | 1.7 (-1.6 - 5.0) |
| 1997 | Myocardial Degeneration | low | 40-69 | 0.1 (-0.1 - 0.3) | 0.2 (-0.1 - 0.5) |
| 1997 | Myocardial Degeneration | low | 70-79 | 0.2 (0.1 - 0.4) | 1.8 (0.6 - 3.1) |
| 1997 | Myocardial Degeneration | low | 80-89 | 0.1 (0.0 - 0.2) | 2.5 (0.3 - 4.8) |
| 1997 | Non-follicular lymphoma, unspecified | high | under 40 | 9.1 (-2.9 - 21.1) | 0.1 (-0.1 - 0.4) |
| 1997 | Non-follicular lymphoma, unspecified | high | 40-69 | 11.6 (6.7 - 16.5) | 4.2 (2.3 - 6.0) |
| 1997 | Non-follicular lymphoma, unspecified | high | 70-79 | 7.7 (3.8 - 11.5) | 39.7 (18.9 - 60.4) |
| 1997 | Non-follicular lymphoma, unspecified | high | 80-89 | 3.6 (1.0 - 6.3) | 75.9 (19.7 - 132.1) |
| 1997 | Non-follicular lymphoma, unspecified | intermediate | under 40 | 2.5 (0.3 - 4.6) | 0.2 (0.0 - 0.3) |
| 1997 | Non-follicular lymphoma, unspecified | intermediate | 40-69 | 8.7 (7.0 - 10.3) | 5.7 (4.5 - 6.9) |
| 1997 | Non-follicular lymphoma, unspecified | intermediate | 70-79 | 5.0 (3.8 - 6.2) | 29.8 (22.5 - 37.2) |
| 1997 | Non-follicular lymphoma, unspecified | intermediate | 80-89 | 2.1 (1.4 - 2.9) | 48.7 (31.0 - 66.4) |
| 1997 | Non-follicular lymphoma, unspecified | low | under 40 | 0.9 (-0.8 - 2.6) | 0.3 (-0.2 - 0.8) |
| 1997 | Non-follicular lymphoma, unspecified | low | 40-69 | 6.9 (5.6 - 8.2) | 9.3 (7.4 - 11.2) |
| 1997 | Non-follicular lymphoma, unspecified | low | 70-79 | 4.1 (3.5 - 4.8) | 32.4 (27.1 - 37.7) |
| 1997 | Non-follicular lymphoma, unspecified | low | 80-89 | 1.8 (1.5 - 2.2) | 50.7 (40.7 - 60.6) |
| 1997 | Osteomyelitis | intermediate | 70-79 | 0.1 (-0.1 - 0.2) | 0.5 (-0.4 - 1.4) |
| 1997 | Osteomyelitis | low | 70-79 | 0.1 (-0.0 - 0.1) | 0.5 (-0.2 - 1.1) |
| 1997 | Osteomyelitis | low | 80-89 | 0.0 (-0.0 - 0.1) | 1.0 (-0.4 - 2.4) |
| 1997 | Peritonitis & Acute Abdomen | high | 80-89 | 0.5 (-0.5 - 1.5) | 10.8 (-10.4 - 32.1) |
| 1997 | Peritonitis & Acute Abdomen | intermediate | 40-69 | 0.3 (-0.0 - 0.6) | 0.2 (-0.0 - 0.4) |
| 1997 | Peritonitis & Acute Abdomen | intermediate | 70-79 | 0.3 (0.0 - 0.6) | 1.9 (0.0 - 3.7) |
| 1997 | Peritonitis & Acute Abdomen | intermediate | 80-89 | 0.1 (-0.1 - 0.4) | 3.4 (-1.3 - 8.0) |
| 1997 | Peritonitis & Acute Abdomen | low | 40-69 | 0.1 (-0.1 - 0.3) | 0.2 (-0.1 - 0.5) |
| 1997 | Peritonitis & Acute Abdomen | low | 70-79 | 0.2 (0.0 - 0.3) | 1.4 (0.3 - 2.4) |
| 1997 | Peritonitis & Acute Abdomen | low | 80-89 | 0.2 (0.1 - 0.3) | 6.1 (2.6 - 9.5) |
| 1997 | Pleurisy, Pyothorax | high | 80-89 | 0.5 (-0.5 - 1.5) | 10.8 (-10.4 - 32.1) |
| 1997 | Pleurisy, Pyothorax | intermediate | 40-69 | 0.1 (-0.1 - 0.3) | 0.1 (-0.1 - 0.2) |
| 1997 | Pleurisy, Pyothorax | intermediate | 70-79 | 0.2 (-0.1 - 0.4) | 0.9 (-0.4 - 2.2) |
| 1997 | Pleurisy, Pyothorax | intermediate | 80-89 | 0.1 (-0.1 - 0.2) | 1.7 (-1.6 - 5.0) |
| 1997 | Pleurisy, Pyothorax | low | 40-69 | 0.1 (-0.1 - 0.2) | 0.1 (-0.1 - 0.3) |
| 1997 | Pleurisy, Pyothorax | low | 70-79 | 0.1 (0.0 - 0.2) | 0.9 (0.0 - 1.8) |
| 1997 | Pleurisy, Pyothorax | low | 80-89 | 0.1 (0.0 - 0.2) | 3.5 (0.9 - 6.2) |
| 1997 | Pneumoconiosis associated with tuberculosis | low | 80-89 | 0.0 (-0.0 - 0.1) | 1.0 (-0.4 - 2.4) |
| 1997 | Pneumonitis | high | 70-79 | 0.5 (-0.5 - 1.6) | 2.8 (-2.7 - 8.4) |
| 1997 | Pneumonitis | high | 80-89 | 0.5 (-0.5 - 1.5) | 10.8 (-10.4 - 32.1) |
| 1997 | Pneumonitis | intermediate | 40-69 | 0.6 (0.1 - 1.0) | 0.4 (0.1 - 0.7) |
| 1997 | Pneumonitis | intermediate | 70-79 | 0.5 (0.1 - 0.8) | 2.8 (0.6 - 5.0) |
| 1997 | Pneumonitis | intermediate | 80-89 | 0.4 (0.1 - 0.8) | 10.1 (2.0 - 18.1) |
| 1997 | Pneumonitis | low | under 40 | 0.9 (-0.8 - 2.6) | 0.3 (-0.2 - 0.8) |
| 1997 | Pneumonitis | low | 40-69 | 0.5 (0.1 - 0.9) | 0.7 (0.2 - 1.2) |
| 1997 | Pneumonitis | low | 70-79 | 0.4 (0.2 - 0.6) | 2.9 (1.3 - 4.5) |
| 1997 | Pneumonitis | low | 80-89 | 0.4 (0.2 - 0.6) | 11.1 (6.5 - 15.8) |
| 1997 | Primary or secondary Liver Cancer Unspecified | high | 40-69 | 8.5 (4.3 - 12.8) | 3.1 (1.5 - 4.7) |
| 1997 | Primary or secondary Liver Cancer Unspecified | high | 70-79 | 2.7 (0.4 - 5.1) | 14.2 (1.7 - 26.6) |
| 1997 | Primary or secondary Liver Cancer Unspecified | high | 80-89 | 2.6 (0.4 - 4.9) | 54.2 (6.7 - 101.7) |
| 1997 | Primary or secondary Liver Cancer Unspecified | intermediate | under 40 | 1.5 (-0.2 - 3.1) | 0.1 (-0.0 - 0.2) |
| 1997 | Primary or secondary Liver Cancer Unspecified | intermediate | 40-69 | 5.4 (4.0 - 6.7) | 3.5 (2.6 - 4.4) |
| 1997 | Primary or secondary Liver Cancer Unspecified | intermediate | 70-79 | 3.1 (2.2 - 4.1) | 18.7 (12.9 - 24.4) |
| 1997 | Primary or secondary Liver Cancer Unspecified | intermediate | 80-89 | 1.6 (1.0 - 2.3) | 36.9 (21.5 - 52.4) |
| 1997 | Primary or secondary Liver Cancer Unspecified | low | 40-69 | 4.2 (3.1 - 5.2) | 5.6 (4.2 - 7.1) |
| 1997 | Primary or secondary Liver Cancer Unspecified | low | 70-79 | 3.9 (3.3 - 4.6) | 31.1 (25.9 - 36.3) |
| 1997 | Primary or secondary Liver Cancer Unspecified | low | 80-89 | 1.6 (1.3 - 2.0) | 45.6 (36.2 - 55.0) |
| 1997 | Pulmonary Embolism | high | 40-69 | 1.8 (-0.2 - 3.9) | 0.7 (-0.1 - 1.4) |
| 1997 | Pulmonary Embolism | high | 70-79 | 4.4 (1.4 - 7.3) | 22.7 (7.0 - 38.4) |
| 1997 | Pulmonary Embolism | high | 80-89 | 1.6 (-0.2 - 3.3) | 32.5 (-4.3 - 69.3) |
| 1997 | Pulmonary Embolism | intermediate | under 40 | 2.0 (0.1 - 3.9) | 0.1 (0.0 - 0.3) |
| 1997 | Pulmonary Embolism | intermediate | 40-69 | 4.6 (3.3 - 5.9) | 3.0 (2.2 - 3.9) |
| 1997 | Pulmonary Embolism | intermediate | 70-79 | 5.4 (4.2 - 6.6) | 32.2 (24.6 - 39.8) |
| 1997 | Pulmonary Embolism | intermediate | 80-89 | 3.3 (2.3 - 4.2) | 73.8 (52.0 - 95.7) |
| 1997 | Pulmonary Embolism | low | under 40 | 1.7 (-0.7 - 4.1) | 0.5 (-0.2 - 1.2) |
| 1997 | Pulmonary Embolism | low | 40-69 | 5.1 (3.9 - 6.3) | 6.9 (5.3 - 8.5) |
| 1997 | Pulmonary Embolism | low | 70-79 | 4.3 (3.7 - 5.0) | 34.3 (28.8 - 39.7) |
| 1997 | Pulmonary Embolism | low | 80-89 | 3.3 (2.8 - 3.8) | 92.2 (78.8 - 105.6) |
| 1997 | Self-harm by unspecified means | low | 70-79 | 0.0 (-0.0 - 0.1) | 0.2 (-0.2 - 0.7) |
| 1997 | Self-poisoning unspecified | high | under 40 | 13.6 (-0.7 - 28.0) | 0.2 (-0.0 - 0.5) |
| 1997 | Self-poisoning unspecified | high | 40-69 | 5.5 (2.0 - 9.0) | 2.0 (0.7 - 3.3) |
| 1997 | Self-poisoning unspecified | intermediate | under 40 | 14.3 (9.5 - 19.1) | 1.1 (0.7 - 1.4) |
| 1997 | Self-poisoning unspecified | intermediate | 40-69 | 4.0 (2.8 - 5.1) | 2.6 (1.8 - 3.4) |
| 1997 | Self-poisoning unspecified | intermediate | 70-79 | 0.3 (0.0 - 0.6) | 1.9 (0.0 - 3.7) |
| 1997 | Self-poisoning unspecified | intermediate | 80-89 | 0.1 (-0.1 - 0.4) | 3.4 (-1.3 - 8.0) |
| 1997 | Self-poisoning unspecified | low | under 40 | 11.3 (5.5 - 17.1) | 3.3 (1.5 - 5.1) |
| 1997 | Self-poisoning unspecified | low | 40-69 | 2.7 (1.8 - 3.5) | 3.6 (2.4 - 4.7) |
| 1997 | Self-poisoning unspecified | low | 70-79 | 0.3 (0.1 - 0.5) | 2.5 (1.0 - 3.9) |
| 1997 | Self-poisoning unspecified | low | 80-89 | 0.1 (0.0 - 0.2) | 3.0 (0.6 - 5.5) |
| 1997 | Senility | high | 80-89 | 2.6 (0.4 - 4.9) | 54.2 (6.7 - 101.7) |
| 1997 | Senility | intermediate | 70-79 | 0.3 (0.0 - 0.6) | 1.9 (0.0 - 3.7) |
| 1997 | Senility | intermediate | 80-89 | 2.1 (1.4 - 2.9) | 48.7 (31.0 - 66.4) |
| 1997 | Senility | low | 70-79 | 0.4 (0.2 - 0.6) | 3.2 (1.5 - 4.8) |
| 1997 | Senility | low | 80-89 | 2.0 (1.7 - 2.4) | 57.2 (46.7 - 67.8) |
| 1997 | Sepsis (Non- maternal and neonatal sepsis) | high | 40-69 | 1.2 (-0.5 - 2.9) | 0.4 (-0.2 - 1.0) |
| 1997 | Sepsis (Non- maternal and neonatal sepsis) | high | 70-79 | 0.5 (-0.5 - 1.6) | 2.8 (-2.7 - 8.4) |
| 1997 | Sepsis (Non- maternal and neonatal sepsis) | high | 80-89 | 1.0 (-0.4 - 2.5) | 21.7 (-8.4 - 51.7) |
| 1997 | Sepsis (Non- maternal and neonatal sepsis) | intermediate | under 40 | 2.0 (0.1 - 3.9) | 0.1 (0.0 - 0.3) |
| 1997 | Sepsis (Non- maternal and neonatal sepsis) | intermediate | 40-69 | 1.7 (0.9 - 2.5) | 1.1 (0.6 - 1.6) |
| 1997 | Sepsis (Non- maternal and neonatal sepsis) | intermediate | 70-79 | 1.6 (0.9 - 2.2) | 9.3 (5.2 - 13.4) |
| 1997 | Sepsis (Non- maternal and neonatal sepsis) | intermediate | 80-89 | 1.4 (0.8 - 2.0) | 31.9 (17.6 - 46.2) |
| 1997 | Sepsis (Non- maternal and neonatal sepsis) | low | under 40 | 4.3 (0.6 - 8.1) | 1.3 (0.2 - 2.4) |
| 1997 | Sepsis (Non- maternal and neonatal sepsis) | low | 40-69 | 1.7 (1.0 - 2.3) | 2.2 (1.3 - 3.1) |
| 1997 | Sepsis (Non- maternal and neonatal sepsis) | low | 70-79 | 1.7 (1.2 - 2.1) | 13.1 (9.7 - 16.4) |
| 1997 | Sepsis (Non- maternal and neonatal sepsis) | low | 80-89 | 1.2 (0.9 - 1.5) | 33.9 (25.8 - 42.1) |
| 1997 | Shock, Cardiac Arrest, Coma | high | under 40 | 4.5 (-4.2 - 13.2) | 0.1 (-0.1 - 0.2) |
| 1997 | Shock, Cardiac Arrest, Coma | high | 40-69 | 0.6 (-0.6 - 1.8) | 0.2 (-0.2 - 0.6) |
| 1997 | Shock, Cardiac Arrest, Coma | high | 70-79 | 1.6 (-0.2 - 3.5) | 8.5 (-1.1 - 18.1) |
| 1997 | Shock, Cardiac Arrest, Coma | intermediate | under 40 | 1.0 (-0.4 - 2.3) | 0.1 (-0.0 - 0.2) |
| 1997 | Shock, Cardiac Arrest, Coma | intermediate | 40-69 | 0.6 (0.1 - 1.0) | 0.4 (0.1 - 0.7) |
| 1997 | Shock, Cardiac Arrest, Coma | intermediate | 70-79 | 1.4 (0.8 - 2.1) | 8.4 (4.5 - 12.3) |
| 1997 | Shock, Cardiac Arrest, Coma | intermediate | 80-89 | 1.0 (0.5 - 1.6) | 23.5 (11.2 - 35.8) |
| 1997 | Shock, Cardiac Arrest, Coma | low | under 40 | 0.9 (-0.8 - 2.6) | 0.3 (-0.2 - 0.8) |
| 1997 | Shock, Cardiac Arrest, Coma | low | 40-69 | 0.6 (0.2 - 1.0) | 0.8 (0.2 - 1.3) |
| 1997 | Shock, Cardiac Arrest, Coma | low | 70-79 | 0.8 (0.5 - 1.1) | 6.5 (4.2 - 8.9) |
| 1997 | Shock, Cardiac Arrest, Coma | low | 80-89 | 1.3 (1.0 - 1.6) | 36.5 (28.0 - 44.9) |
| 1997 | Undetermined intent Drowning | high | under 40 | 4.5 (-4.2 - 13.2) | 0.1 (-0.1 - 0.2) |
| 1997 | Undetermined intent Drowning | high | 40-69 | 0.6 (-0.6 - 1.8) | 0.2 (-0.2 - 0.6) |
| 1997 | Undetermined intent Drowning | high | 70-79 | 0.5 (-0.5 - 1.6) | 2.8 (-2.7 - 8.4) |
| 1997 | Undetermined intent Drowning | intermediate | under 40 | 3.4 (0.9 - 6.0) | 0.3 (0.1 - 0.4) |
| 1997 | Undetermined intent Drowning | intermediate | 40-69 | 1.3 (0.6 - 2.0) | 0.9 (0.4 - 1.3) |
| 1997 | Undetermined intent Drowning | low | under 40 | 3.5 (0.1 - 6.8) | 1.0 (0.0 - 2.0) |
| 1997 | Undetermined intent Drowning | low | 40-69 | 0.4 (0.1 - 0.8) | 0.6 (0.1 - 1.0) |
| 1997 | Undetermined intent Drowning | low | 70-79 | 0.2 (0.0 - 0.3) | 1.4 (0.3 - 2.4) |
| 1997 | Undetermined intent Drowning | low | 80-89 | 0.0 (-0.0 - 0.1) | 1.0 (-0.4 - 2.4) |
| 1997 | Undetermined intent shooting by Handgun Firearm | intermediate | under 40 | 0.5 (-0.5 - 1.5) | 0.0 (-0.0 - 0.1) |
| 1997 | Undetermined intent shooting by rifle and larger firearm | intermediate | 40-69 | 0.1 (-0.1 - 0.3) | 0.1 (-0.1 - 0.2) |
| 1997 | Undetermined intent shooting by unspecified firearm | low | under 40 | 0.9 (-0.8 - 2.6) | 0.3 (-0.2 - 0.8) |
| 1997 | Undetermined intent shooting by unspecified firearm | low | 40-69 | 0.1 (-0.1 - 0.2) | 0.1 (-0.1 - 0.3) |
| 1997 | Undetermined intent shooting by unspecified firearm | low | 70-79 | 0.0 (-0.0 - 0.1) | 0.2 (-0.2 - 0.7) |
| 1997 | Undetermined intent Poisoning by antiepileptic and psychotropic drugs | high | under 40 | 4.5 (-4.2 - 13.2) | 0.1 (-0.1 - 0.2) |
| 1997 | Undetermined intent Poisoning by antiepileptic and psychotropic drugs | high | 40-69 | 1.8 (-0.2 - 3.9) | 0.7 (-0.1 - 1.4) |
| 1997 | Undetermined intent Poisoning by antiepileptic and psychotropic drugs | intermediate | under 40 | 4.4 (1.6 - 7.3) | 0.3 (0.1 - 0.5) |
| 1997 | Undetermined intent Poisoning by antiepileptic and psychotropic drugs | intermediate | 40-69 | 1.9 (1.1 - 2.7) | 1.2 (0.7 - 1.8) |
| 1997 | Undetermined intent Poisoning by antiepileptic and psychotropic drugs | low | under 40 | 2.6 (-0.3 - 5.5) | 0.8 (-0.1 - 1.6) |
| 1997 | Undetermined intent Poisoning by antiepileptic and psychotropic drugs | low | 40-69 | 0.6 (0.2 - 1.1) | 0.9 (0.3 - 1.4) |
| 1997 | Undetermined intent Poisoning by antiepileptic and psychotropic drugs | low | 70-79 | 0.0 (-0.0 - 0.1) | 0.2 (-0.2 - 0.7) |
| 1997 | Undetermined intent Poisoning by multiple or unspecified drug | intermediate | under 40 | 6.9 (3.4 - 10.4) | 0.5 (0.2 - 0.8) |
| 1997 | Undetermined intent Poisoning by multiple or unspecified drug | intermediate | 40-69 | 1.1 (0.5 - 1.8) | 0.7 (0.3 - 1.2) |
| 1997 | Undetermined intent Poisoning by multiple or unspecified drug | intermediate | 70-79 | 0.1 (-0.1 - 0.2) | 0.5 (-0.4 - 1.4) |
| 1997 | Undetermined intent Poisoning by multiple or unspecified drug | intermediate | 80-89 | 0.1 (-0.1 - 0.2) | 1.7 (-1.6 - 5.0) |
| 1997 | Undetermined intent Poisoning by multiple or unspecified drug | low | under 40 | 16.5 (9.7 - 23.3) | 4.8 (2.7 - 7.0) |
| 1997 | Undetermined intent Poisoning by multiple or unspecified drug | low | 40-69 | 1.5 (0.9 - 2.1) | 2.0 (1.2 - 2.9) |
| 1997 | Undetermined intent Poisoning by multiple or unspecified drug | low | 70-79 | 0.0 (-0.0 - 0.1) | 0.2 (-0.2 - 0.7) |
| 1997 | Undetermined intent Poisoning by multiple or unspecified drug | low | 80-89 | 0.1 (0.0 - 0.2) | 2.5 (0.3 - 4.8) |
| 1997 | Undetermined intent Poisoning by narcotics and psychodysleptics drugs | high | under 40 | 4.5 (-4.2 - 13.2) | 0.1 (-0.1 - 0.2) |
| 1997 | Undetermined intent Poisoning by narcotics and psychodysleptics drugs | intermediate | under 40 | 7.9 (4.2 - 11.6) | 0.6 (0.3 - 0.9) |
| 1997 | Undetermined intent Poisoning by narcotics and psychodysleptics drugs | intermediate | 40-69 | 0.8 (0.2 - 1.3) | 0.5 (0.2 - 0.8) |
| 1997 | Undetermined intent Poisoning by narcotics and psychodysleptics drugs | low | under 40 | 3.5 (0.1 - 6.8) | 1.0 (0.0 - 2.0) |
| 1997 | Undetermined intent Poisoning by narcotics and psychodysleptics drugs | low | 40-69 | 0.6 (0.2 - 1.1) | 0.9 (0.3 - 1.4) |
| 1997 | Undetermined intent Poisoning by narcotics and psychodysleptics drugs | low | 70-79 | 0.1 (-0.0 - 0.1) | 0.5 (-0.2 - 1.1) |
| 1997 | Undetermined intent Poisoning by other gases and vapors | intermediate | under 40 | 0.5 (-0.5 - 1.5) | 0.0 (-0.0 - 0.1) |
| 1997 | Undetermined intent Poisoning by other gases and vapors | low | 40-69 | 0.1 (-0.1 - 0.2) | 0.1 (-0.1 - 0.3) |
| 1997 | Undetermined intent Poisoning by other gases and vapors | low | 80-89 | 0.0 (-0.0 - 0.1) | 1.0 (-0.4 - 2.4) |
| 1997 | Undetermined intent Poisoning by pesticides | low | 40-69 | 0.1 (-0.1 - 0.2) | 0.1 (-0.1 - 0.3) |
| 1997 | Undetermined intent Poisoning by solvents and halogenated hydrocarbons | low | 40-69 | 0.1 (-0.1 - 0.2) | 0.1 (-0.1 - 0.3) |
| 1997 | Undetermined intent Poisoning by unspecified chemicals and noxious substances | low | 40-69 | 0.1 (-0.1 - 0.2) | 0.1 (-0.1 - 0.3) |
| 1997 | Undetermined intent Poisoning by unspecified chemicals and noxious substances | low | 80-89 | 0.0 (-0.0 - 0.1) | 0.5 (-0.5 - 1.5) |
| 1997 | Undetermined intent Poisoning by unspecified drugs and biological drugs | high | under 40 | 4.5 (-4.2 - 13.2) | 0.1 (-0.1 - 0.2) |
| 1997 | Undetermined intent Poisoning by unspecified drugs and biological drugs | high | 40-69 | 1.8 (-0.2 - 3.9) | 0.7 (-0.1 - 1.4) |
| 1997 | Undetermined intent Poisoning by unspecified drugs and biological drugs | high | 70-79 | 0.5 (-0.5 - 1.6) | 2.8 (-2.7 - 8.4) |
| 1997 | Undetermined intent Poisoning by unspecified drugs and biological drugs | intermediate | under 40 | 7.4 (3.8 - 11.0) | 0.5 (0.3 - 0.8) |
| 1997 | Undetermined intent Poisoning by unspecified drugs and biological drugs | intermediate | 40-69 | 3.1 (2.1 - 4.1) | 2.0 (1.3 - 2.7) |
| 1997 | Undetermined intent Poisoning by unspecified drugs and biological drugs | low | under 40 | 6.1 (1.7 - 10.5) | 1.8 (0.5 - 3.1) |
| 1997 | Undetermined intent Poisoning by unspecified drugs and biological drugs | low | 40-69 | 1.4 (0.8 - 2.0) | 1.8 (1.0 - 2.7) |
| 1997 | Undetermined intent Poisoning by unspecified drugs and biological drugs | low | 70-79 | 0.0 (-0.0 - 0.1) | 0.2 (-0.2 - 0.7) |
| 1997 | Undetermined intent Poisoning by unspecified drugs and biological drugs | low | 80-89 | 0.1 (-0.0 - 0.1) | 1.5 (-0.2 - 3.2) |
| 1997 | Undetermined intent Strangulation | high | 40-69 | 0.6 (-0.6 - 1.8) | 0.2 (-0.2 - 0.6) |
| 1997 | Undetermined intent Strangulation | intermediate | under 40 | 1.5 (-0.2 - 3.1) | 0.1 (-0.0 - 0.2) |
| 1997 | Undetermined intent Strangulation | low | under 40 | 0.9 (-0.8 - 2.6) | 0.3 (-0.2 - 0.8) |
| 1997 | Undetermined intent Strangulation | low | 70-79 | 0.0 (-0.0 - 0.1) | 0.2 (-0.2 - 0.7) |
| 1997 | Undetermined intent of Blunt Objects | high | 40-69 | 0.6 (-0.6 - 1.8) | 0.2 (-0.2 - 0.6) |
| 1997 | Undetermined intent of Blunt Objects | intermediate | 70-79 | 0.1 (-0.1 - 0.2) | 0.5 (-0.4 - 1.4) |
| 1997 | Undetermined intent of Crashing | intermediate | under 40 | 1.5 (-0.2 - 3.1) | 0.1 (-0.0 - 0.2) |
| 1997 | Undetermined intent of Hot Objects | low | 40-69 | 0.1 (-0.1 - 0.2) | 0.1 (-0.1 - 0.3) |
| 1997 | Undetermined intent of Moving Objects | intermediate | under 40 | 1.5 (-0.2 - 3.1) | 0.1 (-0.0 - 0.2) |
| 1997 | Undetermined intent of Moving Objects | intermediate | 40-69 | 0.4 (0.0 - 0.7) | 0.2 (0.0 - 0.5) |
| 1997 | Undetermined intent of Moving Objects | low | under 40 | 0.9 (-0.8 - 2.6) | 0.3 (-0.2 - 0.8) |
| 1997 | Undetermined intent of Moving Objects | low | 40-69 | 0.1 (-0.1 - 0.3) | 0.2 (-0.1 - 0.5) |
| 1997 | Undetermined intent of Moving Objects | low | 80-89 | 0.0 (-0.0 - 0.1) | 0.5 (-0.5 - 1.5) |
| 1997 | Undetermined intent of fall | high | under 40 | 4.5 (-4.2 - 13.2) | 0.1 (-0.1 - 0.2) |
| 1997 | Undetermined intent of fall | high | 40-69 | 1.8 (-0.2 - 3.9) | 0.7 (-0.1 - 1.4) |
| 1997 | Undetermined intent of fall | intermediate | under 40 | 1.0 (-0.4 - 2.3) | 0.1 (-0.0 - 0.2) |
| 1997 | Undetermined intent of fall | intermediate | 40-69 | 0.2 (-0.1 - 0.4) | 0.1 (-0.0 - 0.3) |
| 1997 | Undetermined intent of fall | intermediate | 70-79 | 0.1 (-0.1 - 0.2) | 0.5 (-0.4 - 1.4) |
| 1997 | Undetermined intent of fall | low | under 40 | 0.9 (-0.8 - 2.6) | 0.3 (-0.2 - 0.8) |
| 1997 | Undetermined intent of fall | low | 40-69 | 0.1 (-0.1 - 0.3) | 0.2 (-0.1 - 0.5) |
| 1997 | Undetermined intent of fall | low | 80-89 | 0.0 (-0.0 - 0.1) | 0.5 (-0.5 - 1.5) |
| 1997 | Undetermined intent of fire and flames | intermediate | 40-69 | 0.3 (-0.0 - 0.6) | 0.2 (-0.0 - 0.4) |
| 1997 | Undetermined intent of fire and flames | low | under 40 | 0.9 (-0.8 - 2.6) | 0.3 (-0.2 - 0.8) |
| 1997 | Undetermined intent of fire and flames | low | 40-69 | 0.2 (-0.0 - 0.5) | 0.3 (-0.0 - 0.6) |
| 1997 | Unspecified Intestine Diseases | high | 80-89 | 0.5 (-0.5 - 1.5) | 10.8 (-10.4 - 32.1) |
| 1997 | Unspecified Intestine Diseases | intermediate | 40-69 | 0.3 (-0.0 - 0.6) | 0.2 (-0.0 - 0.4) |
| 1997 | Unspecified Intestine Diseases | intermediate | 70-79 | 0.2 (-0.0 - 0.5) | 1.4 (-0.2 - 3.0) |
| 1997 | Unspecified Intestine Diseases | intermediate | 80-89 | 0.3 (0.0 - 0.6) | 6.7 (0.1 - 13.3) |
| 1997 | Unspecified Intestine Diseases | low | 40-69 | 0.5 (0.1 - 0.9) | 0.7 (0.2 - 1.2) |
| 1997 | Unspecified Intestine Diseases | low | 70-79 | 0.5 (0.3 - 0.7) | 3.8 (2.0 - 5.7) |
| 1997 | Unspecified Intestine Diseases | low | 80-89 | 0.1 (0.0 - 0.2) | 3.0 (0.6 - 5.5) |
| 1997 | Unspecified Bacterial Diseases | high | 40-69 | 0.6 (-0.6 - 1.8) | 0.2 (-0.2 - 0.6) |
| 1997 | Unspecified Bacterial Diseases | intermediate | 80-89 | 0.1 (-0.1 - 0.2) | 1.7 (-1.6 - 5.0) |
| 1997 | Unspecified Bacterial Diseases | low | 70-79 | 0.0 (-0.0 - 0.1) | 0.2 (-0.2 - 0.7) |
| 1997 | Unspecified Bacterial Diseases | low | 80-89 | 0.1 (0.0 - 0.2) | 3.0 (0.6 - 5.5) |
| 1997 | Unspecified Brain Diseases | high | 40-69 | 1.2 (-0.5 - 2.9) | 0.4 (-0.2 - 1.0) |
| 1997 | Unspecified Brain Diseases | high | 80-89 | 1.0 (-0.4 - 2.5) | 21.7 (-8.4 - 51.7) |
| 1997 | Unspecified Brain Diseases | intermediate | 40-69 | 0.4 (0.0 - 0.7) | 0.2 (0.0 - 0.5) |
| 1997 | Unspecified Brain Diseases | intermediate | 70-79 | 0.3 (0.0 - 0.6) | 1.9 (0.0 - 3.7) |
| 1997 | Unspecified Brain Diseases | intermediate | 80-89 | 0.4 (0.0 - 0.7) | 8.4 (1.0 - 15.7) |
| 1997 | Unspecified Brain Diseases | low | 40-69 | 0.2 (-0.0 - 0.5) | 0.3 (-0.0 - 0.6) |
| 1997 | Unspecified Brain Diseases | low | 70-79 | 0.3 (0.1 - 0.5) | 2.7 (1.2 - 4.2) |
| 1997 | Unspecified Brain Diseases | low | 80-89 | 0.1 (0.0 - 0.2) | 2.5 (0.3 - 4.8) |
| 1997 | Unspecified Bronchitis and Bronchiectasis | high | 70-79 | 0.5 (-0.5 - 1.6) | 2.8 (-2.7 - 8.4) |
| 1997 | Unspecified Bronchitis and Bronchiectasis | intermediate | 40-69 | 0.3 (-0.0 - 0.6) | 0.2 (-0.0 - 0.4) |
| 1997 | Unspecified Bronchitis and Bronchiectasis | intermediate | 70-79 | 0.2 (-0.0 - 0.5) | 1.4 (-0.2 - 3.0) |
| 1997 | Unspecified Bronchitis and Bronchiectasis | intermediate | 80-89 | 0.4 (0.1 - 0.8) | 10.1 (2.0 - 18.1) |
| 1997 | Unspecified Bronchitis and Bronchiectasis | low | 40-69 | 0.5 (0.1 - 0.9) | 0.7 (0.2 - 1.2) |
| 1997 | Unspecified Bronchitis and Bronchiectasis | low | 70-79 | 0.3 (0.1 - 0.4) | 2.0 (0.7 - 3.4) |
| 1997 | Unspecified Bronchitis and Bronchiectasis | low | 80-89 | 0.2 (0.1 - 0.3) | 5.6 (2.3 - 8.9) |
| 1997 | Unspecified CNS Infection | low | under 40 | 0.9 (-0.8 - 2.6) | 0.3 (-0.2 - 0.8) |
| 1997 | Unspecified Cardiomyopathy | high | 40-69 | 4.3 (1.2 - 7.4) | 1.5 (0.4 - 2.7) |
| 1997 | Unspecified Cardiomyopathy | high | 70-79 | 1.1 (-0.4 - 2.6) | 5.7 (-2.2 - 13.5) |
| 1997 | Unspecified Cardiomyopathy | high | 80-89 | 1.0 (-0.4 - 2.5) | 21.7 (-8.4 - 51.7) |
| 1997 | Unspecified Cardiomyopathy | intermediate | under 40 | 1.5 (-0.2 - 3.1) | 0.1 (-0.0 - 0.2) |
| 1997 | Unspecified Cardiomyopathy | intermediate | 40-69 | 5.8 (4.4 - 7.2) | 3.8 (2.9 - 4.8) |
| 1997 | Unspecified Cardiomyopathy | intermediate | 70-79 | 2.0 (1.3 - 2.8) | 12.1 (7.5 - 16.8) |
| 1997 | Unspecified Cardiomyopathy | intermediate | 80-89 | 0.2 (-0.0 - 0.5) | 5.0 (-0.7 - 10.7) |
| 1997 | Unspecified Cardiomyopathy | low | under 40 | 1.7 (-0.7 - 4.1) | 0.5 (-0.2 - 1.2) |
| 1997 | Unspecified Cardiomyopathy | low | 40-69 | 4.3 (3.2 - 5.4) | 5.8 (4.3 - 7.3) |
| 1997 | Unspecified Cardiomyopathy | low | 70-79 | 1.3 (1.0 - 1.7) | 10.6 (7.6 - 13.6) |
| 1997 | Unspecified Cardiomyopathy | low | 80-89 | 0.5 (0.3 - 0.6) | 12.7 (7.7 - 17.6) |
| 1997 | Unspecified Chromosomal Diseases | intermediate | under 40 | 1.5 (-0.2 - 3.1) | 0.1 (-0.0 - 0.2) |
| 1997 | Unspecified Congenital Diseases | intermediate | under 40 | 1.0 (-0.4 - 2.3) | 0.1 (-0.0 - 0.2) |
| 1997 | Unspecified Congenital Diseases | low | 80-89 | 0.0 (-0.0 - 0.1) | 0.5 (-0.5 - 1.5) |
| 1997 | Unspecified Digestive Diseases | low | 70-79 | 0.0 (-0.0 - 0.1) | 0.2 (-0.2 - 0.7) |
| 1997 | Unspecified Digestive Diseases | low | 80-89 | 0.0 (-0.0 - 0.1) | 1.0 (-0.4 - 2.4) |
| 1997 | Unspecified Eating Disorders | intermediate | 70-79 | 0.1 (-0.1 - 0.2) | 0.5 (-0.4 - 1.4) |
| 1997 | Unspecified Eating Disorders | low | 40-69 | 0.1 (-0.1 - 0.3) | 0.2 (-0.1 - 0.5) |
| 1997 | Unspecified Eating Disorders | low | 80-89 | 0.1 (-0.0 - 0.1) | 1.5 (-0.2 - 3.2) |
| 1997 | Unspecified Endocrine Cancer | intermediate | 70-79 | 0.1 (-0.1 - 0.2) | 0.5 (-0.4 - 1.4) |
| 1997 | Unspecified Endocrine Cancer | low | 70-79 | 0.0 (-0.0 - 0.1) | 0.2 (-0.2 - 0.7) |
| 1997 | Unspecified Female Genital Cancer | high | 80-89 | 1.0 (-0.4 - 2.5) | 21.7 (-8.4 - 51.7) |
| 1997 | Unspecified Female Genital Cancer | intermediate | 40-69 | 0.2 (-0.1 - 0.4) | 0.1 (-0.0 - 0.3) |
| 1997 | Unspecified Female Genital Cancer | intermediate | 70-79 | 0.4 (0.0 - 0.7) | 2.3 (0.3 - 4.4) |
| 1997 | Unspecified Female Genital Cancer | intermediate | 80-89 | 0.1 (-0.1 - 0.4) | 3.4 (-1.3 - 8.0) |
| 1997 | Unspecified Female Genital Cancer | low | 40-69 | 0.2 (-0.0 - 0.5) | 0.3 (-0.0 - 0.6) |
| 1997 | Unspecified Female Genital Cancer | low | 70-79 | 0.3 (0.1 - 0.5) | 2.3 (0.9 - 3.7) |
| 1997 | Unspecified Female Genital Cancer | low | 80-89 | 0.2 (0.1 - 0.4) | 6.6 (3.0 - 10.2) |
| 1997 | Unspecified GI Cancer | high | 40-69 | 2.4 (0.1 - 4.8) | 0.9 (0.0 - 1.7) |
| 1997 | Unspecified GI Cancer | high | 70-79 | 1.1 (-0.4 - 2.6) | 5.7 (-2.2 - 13.5) |
| 1997 | Unspecified GI Cancer | intermediate | under 40 | 0.5 (-0.5 - 1.5) | 0.0 (-0.0 - 0.1) |
| 1997 | Unspecified GI Cancer | intermediate | 40-69 | 1.4 (0.7 - 2.1) | 0.9 (0.5 - 1.4) |
| 1997 | Unspecified GI Cancer | intermediate | 70-79 | 1.4 (0.8 - 2.1) | 8.4 (4.5 - 12.3) |
| 1997 | Unspecified GI Cancer | intermediate | 80-89 | 1.0 (0.5 - 1.6) | 23.5 (11.2 - 35.8) |
| 1997 | Unspecified GI Cancer | low | 40-69 | 1.7 (1.0 - 2.3) | 2.2 (1.3 - 3.1) |
| 1997 | Unspecified GI Cancer | low | 70-79 | 1.3 (0.9 - 1.6) | 9.9 (7.0 - 12.8) |
| 1997 | Unspecified GI Cancer | low | 80-89 | 0.9 (0.7 - 1.2) | 25.3 (18.3 - 32.3) |
| 1997 | Unspecified Heart Diseases | high | 40-69 | 3.0 (0.4 - 5.7) | 1.1 (0.1 - 2.1) |
| 1997 | Unspecified Heart Diseases | high | 70-79 | 4.4 (1.4 - 7.3) | 22.7 (7.0 - 38.4) |
| 1997 | Unspecified Heart Diseases | high | 80-89 | 2.1 (0.1 - 4.1) | 43.4 (0.9 - 85.8) |
| 1997 | Unspecified Heart Diseases | intermediate | under 40 | 2.5 (0.3 - 4.6) | 0.2 (0.0 - 0.3) |
| 1997 | Unspecified Heart Diseases | intermediate | 40-69 | 1.8 (1.0 - 2.6) | 1.2 (0.6 - 1.7) |
| 1997 | Unspecified Heart Diseases | intermediate | 70-79 | 3.1 (2.1 - 4.0) | 18.2 (12.5 - 23.9) |
| 1997 | Unspecified Heart Diseases | intermediate | 80-89 | 2.3 (1.5 - 3.1) | 52.0 (33.7 - 70.3) |
| 1997 | Unspecified Heart Diseases | low | under 40 | 3.5 (0.1 - 6.8) | 1.0 (0.0 - 2.0) |
| 1997 | Unspecified Heart Diseases | low | 40-69 | 2.1 (1.3 - 2.8) | 2.8 (1.8 - 3.8) |
| 1997 | Unspecified Heart Diseases | low | 70-79 | 2.1 (1.7 - 2.6) | 16.9 (13.1 - 20.7) |
| 1997 | Unspecified Heart Diseases | low | 80-89 | 2.9 (2.4 - 3.3) | 80.0 (67.6 - 92.5) |
| 1997 | Unspecified Infectious Diseases | high | 70-79 | 1.1 (-0.4 - 2.6) | 5.7 (-2.2 - 13.5) |
| 1997 | Unspecified Infectious Diseases | intermediate | 40-69 | 0.1 (-0.1 - 0.3) | 0.1 (-0.1 - 0.2) |
| 1997 | Unspecified Infectious Diseases | intermediate | 70-79 | 0.2 (-0.0 - 0.5) | 1.4 (-0.2 - 3.0) |
| 1997 | Unspecified Infectious Diseases | intermediate | 80-89 | 0.5 (0.1 - 0.9) | 11.7 (3.0 - 20.5) |
| 1997 | Unspecified Infectious Diseases | low | 70-79 | 0.4 (0.2 - 0.6) | 2.9 (1.3 - 4.5) |
| 1997 | Unspecified Infectious Diseases | low | 80-89 | 0.5 (0.3 - 0.7) | 14.7 (9.3 - 20.0) |
| 1997 | Unspecified Male Genital Cancer | intermediate | 70-79 | 0.1 (-0.1 - 0.2) | 0.5 (-0.4 - 1.4) |
| 1997 | Unspecified Male Genital Cancer | low | 40-69 | 0.1 (-0.1 - 0.2) | 0.1 (-0.1 - 0.3) |
| 1997 | Unspecified Male Genital Cancer | low | 70-79 | 0.0 (-0.0 - 0.1) | 0.2 (-0.2 - 0.7) |
| 1997 | Unspecified Meningitis | high | 70-79 | 0.5 (-0.5 - 1.6) | 2.8 (-2.7 - 8.4) |
| 1997 | Unspecified Meningitis | intermediate | 40-69 | 0.3 (-0.0 - 0.6) | 0.2 (-0.0 - 0.4) |
| 1997 | Unspecified Meningitis | intermediate | 70-79 | 0.2 (-0.1 - 0.4) | 0.9 (-0.4 - 2.2) |
| 1997 | Unspecified Meningitis | low | 40-69 | 0.2 (-0.0 - 0.5) | 0.3 (-0.0 - 0.6) |
| 1997 | Unspecified Meningitis | low | 70-79 | 0.1 (0.0 - 0.2) | 0.9 (0.0 - 1.8) |
| 1997 | Unspecified Meningitis | low | 80-89 | 0.1 (0.0 - 0.1) | 2.0 (0.0 - 4.0) |
| 1997 | Unspecified Oropharynx Cancer | intermediate | 40-69 | 0.1 (-0.1 - 0.3) | 0.1 (-0.1 - 0.2) |
| 1997 | Unspecified Oropharynx Cancer | low | 40-69 | 0.1 (-0.1 - 0.3) | 0.2 (-0.1 - 0.5) |
| 1997 | Unspecified Oropharynx Cancer | low | 70-79 | 0.0 (-0.0 - 0.1) | 0.2 (-0.2 - 0.7) |
| 1997 | Unspecified Oropharynx Cancer | low | 80-89 | 0.0 (-0.0 - 0.1) | 1.0 (-0.4 - 2.4) |
| 1997 | Unspecified Pneumoconiosis | low | 80-89 | 0.0 (-0.0 - 0.1) | 0.5 (-0.5 - 1.5) |
| 1997 | Unspecified Respiratory Cancer | low | 40-69 | 0.1 (-0.1 - 0.2) | 0.1 (-0.1 - 0.3) |
| 1997 | Unspecified Respiratory Cancer | low | 70-79 | 0.1 (-0.0 - 0.1) | 0.5 (-0.2 - 1.1) |
| 1997 | Unspecified Respiratory Cancer | low | 80-89 | 0.0 (-0.0 - 0.1) | 1.0 (-0.4 - 2.4) |
| 1997 | Unspecified Road Injuries | high | 40-69 | 1.2 (-0.5 - 2.9) | 0.4 (-0.2 - 1.0) |
| 1997 | Unspecified Road Injuries | intermediate | under 40 | 2.0 (0.1 - 3.9) | 0.1 (0.0 - 0.3) |
| 1997 | Unspecified Road Injuries | intermediate | 40-69 | 0.4 (0.0 - 0.7) | 0.2 (0.0 - 0.5) |
| 1997 | Unspecified Road Injuries | intermediate | 70-79 | 0.1 (-0.1 - 0.2) | 0.5 (-0.4 - 1.4) |
| 1997 | Unspecified Road Injuries | low | under 40 | 2.6 (-0.3 - 5.5) | 0.8 (-0.1 - 1.6) |
| 1997 | Unspecified Road Injuries | low | 40-69 | 0.1 (-0.1 - 0.2) | 0.1 (-0.1 - 0.3) |
| 1997 | Unspecified Road Injuries | low | 80-89 | 0.0 (-0.0 - 0.1) | 0.5 (-0.5 - 1.5) |
| 1997 | Unspecified Site Cancer | high | 40-69 | 10.4 (5.7 - 15.0) | 3.7 (2.0 - 5.5) |
| 1997 | Unspecified Site Cancer | high | 70-79 | 9.8 (5.5 - 14.2) | 51.0 (27.4 - 74.5) |
| 1997 | Unspecified Site Cancer | high | 80-89 | 4.7 (1.7 - 7.7) | 97.6 (33.8 - 161.3) |
| 1997 | Unspecified Site Cancer | intermediate | under 40 | 1.0 (-0.4 - 2.3) | 0.1 (-0.0 - 0.2) |
| 1997 | Unspecified Site Cancer | intermediate | 40-69 | 8.7 (7.0 - 10.3) | 5.7 (4.5 - 6.9) |
| 1997 | Unspecified Site Cancer | intermediate | 70-79 | 7.0 (5.6 - 8.4) | 41.5 (32.9 - 50.1) |
| 1997 | Unspecified Site Cancer | intermediate | 80-89 | 4.7 (3.6 - 5.9) | 107.4 (81.1 - 133.7) |
| 1997 | Unspecified Site Cancer | low | 40-69 | 8.5 (7.0 - 9.9) | 11.4 (9.4 - 13.5) |
| 1997 | Unspecified Site Cancer | low | 70-79 | 6.5 (5.7 - 7.4) | 51.6 (44.9 - 58.3) |
| 1997 | Unspecified Site Cancer | low | 80-89 | 3.8 (3.3 - 4.3) | 105.4 (91.1 - 119.7) |
| 1997 | Unspecified Traffic Injuries | intermediate | under 40 | 1.0 (-0.4 - 2.3) | 0.1 (-0.0 - 0.2) |
| 1997 | Unspecified Transport Injuries | high | 70-79 | 0.5 (-0.5 - 1.6) | 2.8 (-2.7 - 8.4) |
| 1997 | Unspecified Transport Injuries | intermediate | 70-79 | 0.1 (-0.1 - 0.2) | 0.5 (-0.4 - 1.4) |
| 1997 | Unspecified Transport Injuries | low | 40-69 | 0.2 (-0.0 - 0.5) | 0.3 (-0.0 - 0.6) |
| 1997 | Unspecified Transport Injuries | low | 70-79 | 0.1 (-0.0 - 0.1) | 0.5 (-0.2 - 1.1) |
| 1997 | Unspecified Transport Injuries | low | 80-89 | 0.0 (-0.0 - 0.1) | 1.0 (-0.4 - 2.4) |
| 1997 | Unspecified Urinary Cancer | high | 80-89 | 0.5 (-0.5 - 1.5) | 10.8 (-10.4 - 32.1) |
| 1997 | Unspecified Urinary Cancer | intermediate | 40-69 | 0.2 (-0.1 - 0.4) | 0.1 (-0.0 - 0.3) |
| 1997 | Unspecified Urinary Cancer | intermediate | 70-79 | 0.1 (-0.1 - 0.2) | 0.5 (-0.4 - 1.4) |
| 1997 | Unspecified Urinary Cancer | intermediate | 80-89 | 0.1 (-0.1 - 0.4) | 3.4 (-1.3 - 8.0) |
| 1997 | Unspecified Urinary Cancer | low | 40-69 | 0.3 (0.0 - 0.6) | 0.4 (0.0 - 0.8) |
| 1997 | Unspecified Urinary Cancer | low | 70-79 | 0.2 (0.1 - 0.3) | 1.6 (0.4 - 2.7) |
| 1997 | Unspecified Urinary Cancer | low | 80-89 | 0.0 (-0.0 - 0.1) | 0.5 (-0.5 - 1.5) |
| 1997 | Unspecified Urinary Diseases | high | 70-79 | 0.5 (-0.5 - 1.6) | 2.8 (-2.7 - 8.4) |
| 1997 | Unspecified Urinary Diseases | intermediate | 70-79 | 0.2 (-0.1 - 0.4) | 0.9 (-0.4 - 2.2) |
| 1997 | Unspecified Urinary Diseases | intermediate | 80-89 | 0.4 (0.0 - 0.7) | 8.4 (1.0 - 15.7) |
| 1997 | Unspecified Urinary Diseases | low | 40-69 | 0.1 (-0.1 - 0.2) | 0.1 (-0.1 - 0.3) |
| 1997 | Unspecified Urinary Diseases | low | 70-79 | 0.4 (0.2 - 0.6) | 2.9 (1.3 - 4.5) |
| 1997 | Unspecified Urinary Diseases | low | 80-89 | 0.3 (0.2 - 0.5) | 9.1 (4.9 - 13.3) |
| 1997 | Unspecified Uterus Cancer | high | 40-69 | 1.2 (-0.5 - 2.9) | 0.4 (-0.2 - 1.0) |
| 1997 | Unspecified Uterus Cancer | high | 70-79 | 1.1 (-0.4 - 2.6) | 5.7 (-2.2 - 13.5) |
| 1997 | Unspecified Uterus Cancer | intermediate | under 40 | 0.5 (-0.5 - 1.5) | 0.0 (-0.0 - 0.1) |
| 1997 | Unspecified Uterus Cancer | intermediate | 40-69 | 1.0 (0.4 - 1.6) | 0.7 (0.3 - 1.1) |
| 1997 | Unspecified Uterus Cancer | intermediate | 70-79 | 1.3 (0.7 - 2.0) | 7.9 (4.2 - 11.7) |
| 1997 | Unspecified Uterus Cancer | intermediate | 80-89 | 0.5 (0.1 - 0.9) | 11.7 (3.0 - 20.5) |
| 1997 | Unspecified Uterus Cancer | low | under 40 | 0.9 (-0.8 - 2.6) | 0.3 (-0.2 - 0.8) |
| 1997 | Unspecified Uterus Cancer | low | 40-69 | 1.4 (0.8 - 2.0) | 1.8 (1.0 - 2.7) |
| 1997 | Unspecified Uterus Cancer | low | 70-79 | 1.1 (0.7 - 1.4) | 8.3 (5.7 - 11.0) |
| 1997 | Unspecified Uterus Cancer | low | 80-89 | 0.3 (0.2 - 0.5) | 8.6 (4.5 - 12.7) |
| 1997 | Unspecified Viral Diseases | intermediate | 70-79 | 0.2 (-0.0 - 0.5) | 1.4 (-0.2 - 3.0) |
| 1997 | Unspecified Viral Diseases | intermediate | 80-89 | 0.1 (-0.1 - 0.4) | 3.4 (-1.3 - 8.0) |
| 1997 | Unspecified Viral Diseases | low | 40-69 | 0.1 (-0.1 - 0.3) | 0.2 (-0.1 - 0.5) |
| 1997 | Unspecified Viral Diseases | low | 70-79 | 0.1 (0.0 - 0.2) | 0.9 (0.0 - 1.8) |
| 1997 | Unspecified Viral Diseases | low | 80-89 | 0.3 (0.2 - 0.5) | 9.6 (5.3 - 14.0) |
| 1997 | Unspecified cardiovascular diseases | high | 80-89 | 1.0 (-0.4 - 2.5) | 21.7 (-8.4 - 51.7) |
| 1997 | Unspecified cardiovascular diseases | intermediate | 40-69 | 0.3 (-0.0 - 0.6) | 0.2 (-0.0 - 0.4) |
| 1997 | Unspecified cardiovascular diseases | intermediate | 70-79 | 0.2 (-0.1 - 0.4) | 0.9 (-0.4 - 2.2) |
| 1997 | Unspecified cardiovascular diseases | intermediate | 80-89 | 1.0 (0.5 - 1.6) | 23.5 (11.2 - 35.8) |
| 1997 | Unspecified cardiovascular diseases | low | 40-69 | 0.1 (-0.1 - 0.2) | 0.1 (-0.1 - 0.3) |
| 1997 | Unspecified cardiovascular diseases | low | 70-79 | 0.5 (0.2 - 0.7) | 3.6 (1.8 - 5.4) |
| 1997 | Unspecified cardiovascular diseases | low | 80-89 | 1.0 (0.7 - 1.2) | 26.8 (19.6 - 34.1) |
| 1997 | Unspecified chronic respiratory diseases | intermediate | 40-69 | 0.2 (-0.1 - 0.4) | 0.1 (-0.0 - 0.3) |
| 1997 | Unspecified chronic respiratory diseases | intermediate | 70-79 | 0.3 (0.0 - 0.6) | 1.9 (0.0 - 3.7) |
| 1997 | Unspecified chronic respiratory diseases | intermediate | 80-89 | 0.2 (-0.0 - 0.5) | 5.0 (-0.7 - 10.7) |
| 1997 | Unspecified chronic respiratory diseases | low | 40-69 | 0.3 (0.0 - 0.6) | 0.4 (0.0 - 0.8) |
| 1997 | Unspecified chronic respiratory diseases | low | 70-79 | 0.4 (0.2 - 0.6) | 3.4 (1.7 - 5.1) |
| 1997 | Unspecified chronic respiratory diseases | low | 80-89 | 0.5 (0.3 - 0.7) | 13.2 (8.1 - 18.2) |
| 1997 | Unspecified lower respiratory infectious | high | under 40 | 9.1 (-2.9 - 21.1) | 0.1 (-0.1 - 0.4) |
| 1997 | Unspecified lower respiratory infectious | high | 40-69 | 2.4 (0.1 - 4.8) | 0.9 (0.0 - 1.7) |
| 1997 | Unspecified lower respiratory infectious | high | 70-79 | 10.9 (6.4 - 15.4) | 56.7 (31.8 - 81.5) |
| 1997 | Unspecified lower respiratory infectious | high | 80-89 | 19.3 (13.7 - 24.9) | 401.0 (272.1 - 530.0) |
| 1997 | Unspecified lower respiratory infectious | intermediate | under 40 | 3.9 (1.3 - 6.6) | 0.3 (0.1 - 0.5) |
| 1997 | Unspecified lower respiratory infectious | intermediate | 40-69 | 5.7 (4.3 - 7.1) | 3.8 (2.8 - 4.7) |
| 1997 | Unspecified lower respiratory infectious | intermediate | 70-79 | 8.4 (6.9 - 9.9) | 49.9 (40.4 - 59.3) |
| 1997 | Unspecified lower respiratory infectious | intermediate | 80-89 | 13.9 (12.0 - 15.7) | 313.9 (268.9 - 358.8) |
| 1997 | Unspecified lower respiratory infectious | low | under 40 | 0.9 (-0.8 - 2.6) | 0.3 (-0.2 - 0.8) |
| 1997 | Unspecified lower respiratory infectious | low | 40-69 | 6.2 (5.0 - 7.5) | 8.4 (6.7 - 10.2) |
| 1997 | Unspecified lower respiratory infectious | low | 70-79 | 10.3 (9.3 - 11.3) | 81.6 (73.2 - 90.0) |
| 1997 | Unspecified lower respiratory infectious | low | 80-89 | 14.8 (13.8 - 15.7) | 413.4 (385.1 - 441.7) |
| 1997 | Unspecified type of Stroke | high | 40-69 | 2.4 (0.1 - 4.8) | 0.9 (0.0 - 1.7) |
| 1997 | Unspecified type of Stroke | high | 70-79 | 18.0 (12.5 - 23.6) | 93.5 (61.6 - 125.4) |
| 1997 | Unspecified type of Stroke | high | 80-89 | 22.9 (17.0 - 28.9) | 476.9 (336.3 - 617.5) |
| 1997 | Unspecified type of Stroke | intermediate | 40-69 | 4.4 (3.2 - 5.7) | 2.9 (2.1 - 3.7) |
| 1997 | Unspecified type of Stroke | intermediate | 70-79 | 18.5 (16.4 - 20.6) | 110.0 (96.0 - 124.1) |
| 1997 | Unspecified type of Stroke | intermediate | 80-89 | 23.6 (21.3 - 25.8) | 533.7 (475.2 - 592.2) |
| 1997 | Unspecified type of Stroke | low | 40-69 | 6.9 (5.6 - 8.2) | 9.3 (7.4 - 11.2) |
| 1997 | Unspecified type of Stroke | low | 70-79 | 18.3 (17.0 - 19.6) | 144.4 (133.3 - 155.6) |
| 1997 | Unspecified type of Stroke | low | 80-89 | 23.2 (22.1 - 24.3) | 649.9 (614.5 - 685.4) |
| 1997 | Unspecified upper respiratory infectious | intermediate | under 40 | 1.0 (-0.4 - 2.3) | 0.1 (-0.0 - 0.2) |
| 1997 | Unspecified upper respiratory infectious | intermediate | 80-89 | 0.1 (-0.1 - 0.4) | 3.4 (-1.3 - 8.0) |
| 1997 | Unspecified upper respiratory infectious | low | 70-79 | 0.0 (-0.0 - 0.1) | 0.2 (-0.2 - 0.7) |
| 1997 | Unspecified upper respiratory infectious | low | 80-89 | 0.1 (0.0 - 0.2) | 4.1 (1.2 - 6.9) |
| 1997 | Urinary Obstruction Diseases | intermediate | 40-69 | 0.2 (-0.1 - 0.4) | 0.1 (-0.0 - 0.3) |
| 1997 | Urinary Obstruction Diseases | intermediate | 70-79 | 0.1 (-0.1 - 0.2) | 0.5 (-0.4 - 1.4) |
| 1997 | Urinary Obstruction Diseases | low | 40-69 | 0.1 (-0.1 - 0.2) | 0.1 (-0.1 - 0.3) |
| 1997 | Urinary Obstruction Diseases | low | 70-79 | 0.0 (-0.0 - 0.1) | 0.2 (-0.2 - 0.7) |
| 1997 | Urinary Obstruction Diseases | low | 80-89 | 0.0 (-0.0 - 0.1) | 0.5 (-0.5 - 1.5) |
| 1997 | hepatitis B unspecified | intermediate | 40-69 | 0.1 (-0.1 - 0.3) | 0.1 (-0.1 - 0.2) |
| 1997 | hepatitis B unspecified | low | 40-69 | 0.1 (-0.1 - 0.2) | 0.1 (-0.1 - 0.3) |
| 1997 | myeloid leukemia by age | high | 40-69 | 0.6 (-0.6 - 1.8) | 0.2 (-0.2 - 0.6) |
| 1997 | myeloid leukemia by age | high | 80-89 | 1.0 (-0.4 - 2.5) | 21.7 (-8.4 - 51.7) |
| 1997 | myeloid leukemia by age | intermediate | 70-79 | 0.2 (-0.0 - 0.5) | 1.4 (-0.2 - 3.0) |
| 1997 | myeloid leukemia by age | low | 40-69 | 0.2 (-0.0 - 0.5) | 0.3 (-0.0 - 0.6) |
| 1997 | myeloid leukemia by age | low | 70-79 | 0.1 (-0.0 - 0.1) | 0.5 (-0.2 - 1.1) |
| 1997 | myeloid leukemia by age | low | 80-89 | 0.1 (0.0 - 0.1) | 2.0 (0.0 - 4.0) |
| 1997 | right heart failure and pulmonary heart disease | intermediate | under 40 | 0.5 (-0.5 - 1.5) | 0.0 (-0.0 - 0.1) |
| 1997 | right heart failure and pulmonary heart disease | intermediate | 40-69 | 0.1 (-0.1 - 0.3) | 0.1 (-0.1 - 0.2) |
| 1997 | right heart failure and pulmonary heart disease | low | 40-69 | 0.1 (-0.1 - 0.3) | 0.2 (-0.1 - 0.5) |
| 1997 | right heart failure and pulmonary heart disease | low | 70-79 | 0.1 (-0.0 - 0.1) | 0.5 (-0.2 - 1.1) |
| 1997 | right heart failure and pulmonary heart disease | low | 80-89 | 0.0 (-0.0 - 0.1) | 1.0 (-0.4 - 2.4) |
| 1997 | upper and lower limb cancer | low | 70-79 | 0.0 (-0.0 - 0.1) | 0.2 (-0.2 - 0.7) |
| 2023 | Abdomen and Pelvis Cancer | high | 40-69 | 2.0 (0.4 - 3.5) | 0.6 (0.1 - 1.0) |
| 2023 | Abdomen and Pelvis Cancer | high | 70-79 | 0.7 (0.0 - 1.5) | 2.1 (0.0 - 4.2) |
| 2023 | Abdomen and Pelvis Cancer | high | 80-89 | 0.2 (-0.1 - 0.5) | 2.8 (-1.1 - 6.6) |
| 2023 | Abdomen and Pelvis Cancer | high | 90+ | 0.3 (-0.1 - 0.6) | 19.0 (-7.3 - 45.3) |
| 2023 | Abdomen and Pelvis Cancer | intermediate | 40-69 | 0.7 (0.3 - 1.1) | 0.6 (0.3 - 0.9) |
| 2023 | Abdomen and Pelvis Cancer | intermediate | 70-79 | 0.9 (0.5 - 1.3) | 4.0 (2.3 - 5.6) |
| 2023 | Abdomen and Pelvis Cancer | intermediate | 80-89 | 0.8 (0.5 - 1.1) | 13.3 (8.6 - 18.0) |
| 2023 | Abdomen and Pelvis Cancer | intermediate | 90+ | 0.4 (0.2 - 0.7) | 32.7 (14.2 - 51.2) |
| 2023 | Abdomen and Pelvis Cancer | low | 40-69 | 0.3 (-0.1 - 0.7) | 0.5 (-0.2 - 1.1) |
| 2023 | Abdomen and Pelvis Cancer | low | 70-79 | 0.8 (0.3 - 1.2) | 5.2 (2.3 - 8.2) |
| 2023 | Abdomen and Pelvis Cancer | low | 80-89 | 0.4 (0.2 - 0.6) | 8.9 (4.4 - 13.4) |
| 2023 | Abdomen and Pelvis Cancer | low | 90+ | 0.2 (0.1 - 0.4) | 20.5 (7.1 - 33.8) |
| 2023 | Acute Respiratory Failure | high | 40-69 | 0.7 (-0.3 - 1.6) | 0.2 (-0.1 - 0.5) |
| 2023 | Acute Respiratory Failure | high | 70-79 | 0.2 (-0.2 - 0.6) | 0.5 (-0.5 - 1.6) |
| 2023 | Acute Respiratory Failure | high | 80-89 | 0.6 (0.1 - 1.0) | 6.9 (0.9 - 13.0) |
| 2023 | Acute Respiratory Failure | high | 90+ | 0.3 (-0.1 - 0.6) | 19.0 (-7.3 - 45.3) |
| 2023 | Acute Respiratory Failure | intermediate | 40-69 | 0.2 (0.0 - 0.4) | 0.2 (0.0 - 0.4) |
| 2023 | Acute Respiratory Failure | intermediate | 70-79 | 0.5 (0.2 - 0.7) | 2.1 (0.9 - 3.2) |
| 2023 | Acute Respiratory Failure | intermediate | 80-89 | 0.4 (0.2 - 0.6) | 6.0 (2.9 - 9.2) |
| 2023 | Acute Respiratory Failure | intermediate | 90+ | 0.1 (0.0 - 0.3) | 10.9 (0.2 - 21.6) |
| 2023 | Acute Respiratory Failure | low | 70-79 | 0.2 (-0.0 - 0.4) | 1.3 (-0.2 - 2.8) |
| 2023 | Acute Respiratory Failure | low | 80-89 | 0.4 (0.2 - 0.6) | 8.9 (4.4 - 13.4) |
| 2023 | Acute Respiratory Failure | low | 90+ | 0.4 (0.2 - 0.6) | 36.4 (18.6 - 54.2) |
| 2023 | Acute kidney failure | high | 70-79 | 0.2 (-0.2 - 0.6) | 0.5 (-0.5 - 1.6) |
| 2023 | Acute kidney failure | high | 80-89 | 1.2 (0.5 - 1.9) | 15.2 (6.2 - 24.2) |
| 2023 | Acute kidney failure | high | 90+ | 0.9 (0.2 - 1.6) | 66.5 (17.3 - 115.8) |
| 2023 | Acute kidney failure | intermediate | 40-69 | 0.3 (0.1 - 0.6) | 0.3 (0.1 - 0.5) |
| 2023 | Acute kidney failure | intermediate | 70-79 | 1.0 (0.6 - 1.4) | 4.3 (2.6 - 6.0) |
| 2023 | Acute kidney failure | intermediate | 80-89 | 1.5 (1.1 - 1.8) | 23.6 (17.4 - 29.9) |
| 2023 | Acute kidney failure | intermediate | 90+ | 1.4 (1.0 - 1.9) | 111.8 (77.6 - 146.0) |
| 2023 | Acute kidney failure | low | 40-69 | 0.4 (-0.1 - 0.9) | 0.7 (-0.1 - 1.5) |
| 2023 | Acute kidney failure | low | 70-79 | 1.4 (0.8 - 2.0) | 9.1 (5.2 - 13.0) |
| 2023 | Acute kidney failure | low | 80-89 | 1.2 (0.8 - 1.5) | 24.2 (16.8 - 31.7) |
| 2023 | Acute kidney failure | low | 90+ | 1.5 (1.1 - 1.9) | 127.3 (94.0 - 160.6) |
| 2023 | Adrenal Site Cancer unspecified part of adrenal gland | intermediate | under 40 | 1.2 (0.0 - 2.3) | 0.2 (0.0 - 0.3) |
| 2023 | Adrenal Site Cancer unspecified part of adrenal gland | intermediate | 40-69 | 0.1 (-0.1 - 0.2) | 0.0 (-0.0 - 0.1) |
| 2023 | Adrenal Site Cancer unspecified part of adrenal gland | intermediate | 80-89 | 0.0 (-0.0 - 0.1) | 0.4 (-0.4 - 1.3) |
| 2023 | Adrenal Site Cancer unspecified part of adrenal gland | low | 40-69 | 0.4 (-0.1 - 0.9) | 0.7 (-0.1 - 1.5) |
| 2023 | Adrenal Site Cancer unspecified part of adrenal gland | low | 70-79 | 0.1 (-0.1 - 0.2) | 0.4 (-0.4 - 1.3) |
| 2023 | Adrenal Site Cancer unspecified part of adrenal gland | low | 80-89 | 0.0 (-0.0 - 0.1) | 0.6 (-0.6 - 1.8) |
| 2023 | Adrenal Unspecified Site Cancer in medulla or cortex | high | 40-69 | 0.7 (-0.3 - 1.6) | 0.2 (-0.1 - 0.5) |
| 2023 | Adrenal Unspecified Site Cancer in medulla or cortex | low | 40-69 | 0.1 (-0.1 - 0.4) | 0.2 (-0.2 - 0.7) |
| 2023 | Adrenal Unspecified Site Cancer-parent cause | intermediate | under 40 | 0.3 (-0.3 - 0.9) | 0.0 (-0.0 - 0.1) |
| 2023 | Adrenal Unspecified Site Cancer-parent cause | intermediate | 40-69 | 0.1 (-0.0 - 0.3) | 0.1 (-0.0 - 0.2) |
| 2023 | Adrenal Unspecified Site Cancer-parent cause | intermediate | 70-79 | 0.0 (-0.0 - 0.1) | 0.2 (-0.2 - 0.5) |
| 2023 | Adrenal Unspecified Site Cancer-parent cause | intermediate | 80-89 | 0.0 (-0.0 - 0.1) | 0.4 (-0.4 - 1.3) |
| 2023 | Adrenal Unspecified Site Cancer-parent cause | low | 70-79 | 0.2 (-0.0 - 0.4) | 1.3 (-0.2 - 2.8) |
| 2023 | Alcoholic hepatic failure | high | under 40 | 1.0 (-1.0 - 3.0) | 0.0 (-0.0 - 0.1) |
| 2023 | Alcoholic hepatic failure | high | 40-69 | 1.3 (0.0 - 2.6) | 0.4 (0.0 - 0.8) |
| 2023 | Alcoholic hepatic failure | high | 70-79 | 0.4 (-0.1 - 0.9) | 1.0 (-0.4 - 2.5) |
| 2023 | Alcoholic hepatic failure | high | 80-89 | 0.1 (-0.1 - 0.3) | 1.4 (-1.3 - 4.1) |
| 2023 | Alcoholic hepatic failure | intermediate | under 40 | 0.3 (-0.3 - 0.9) | 0.0 (-0.0 - 0.1) |
| 2023 | Alcoholic hepatic failure | intermediate | 40-69 | 2.9 (2.1 - 3.7) | 2.3 (1.7 - 2.9) |
| 2023 | Alcoholic hepatic failure | intermediate | 70-79 | 0.7 (0.4 - 1.0) | 2.9 (1.5 - 4.3) |
| 2023 | Alcoholic hepatic failure | intermediate | 80-89 | 0.1 (0.0 - 0.2) | 2.1 (0.3 - 4.0) |
| 2023 | Alcoholic hepatic failure | low | 40-69 | 1.4 (0.5 - 2.2) | 2.3 (0.9 - 3.8) |
| 2023 | Alcoholic hepatic failure | low | 70-79 | 0.7 (0.3 - 1.1) | 4.3 (1.7 - 7.0) |
| 2023 | Alcoholic hepatic failure | low | 80-89 | 0.1 (-0.0 - 0.1) | 1.2 (-0.5 - 2.8) |
| 2023 | All, Ill Defined code for causes of death | high | under 40 | 38.4 (28.8 - 48.0) | 1.8 (1.2 - 2.4) |
| 2023 | All, Ill Defined code for causes of death | high | 40-69 | 34.8 (29.4 - 40.1) | 10.1 (8.2 - 12.1) |
| 2023 | All, Ill Defined code for causes of death | high | 70-79 | 16.4 (13.3 - 19.6) | 46.1 (36.5 - 55.8) |
| 2023 | All, Ill Defined code for causes of death | high | 80-89 | 10.0 (8.0 - 11.9) | 124.3 (98.6 - 150.0) |
| 2023 | All, Ill Defined code for causes of death | high | 90+ | 7.3 (5.5 - 9.2) | 532.1 (393.1 - 671.1) |
| 2023 | All, Ill Defined code for causes of death | intermediate | under 40 | 27.9 (23.1 - 32.7) | 3.7 (2.9 - 4.4) |
| 2023 | All, Ill Defined code for causes of death | intermediate | 40-69 | 23.4 (21.4 - 25.4) | 18.7 (16.9 - 20.6) |
| 2023 | All, Ill Defined code for causes of death | intermediate | 70-79 | 15.0 (13.6 - 16.3) | 65.3 (58.7 - 71.8) |
| 2023 | All, Ill Defined code for causes of death | intermediate | 80-89 | 8.5 (7.6 - 9.4) | 138.4 (123.3 - 153.5) |
| 2023 | All, Ill Defined code for causes of death | intermediate | 90+ | 5.9 (5.0 - 6.7) | 455.5 (386.5 - 524.4) |
| 2023 | All, Ill Defined code for causes of death | low | under 40 | 18.5 (11.5 - 25.5) | 7.2 (4.2 - 10.1) |
| 2023 | All, Ill Defined code for causes of death | low | 40-69 | 21.5 (18.5 - 24.5) | 36.9 (31.1 - 42.6) |
| 2023 | All, Ill Defined code for causes of death | low | 70-79 | 13.4 (11.7 - 15.1) | 88.6 (76.4 - 100.7) |
| 2023 | All, Ill Defined code for causes of death | low | 80-89 | 8.5 (7.6 - 9.4) | 176.8 (156.8 - 196.8) |
| 2023 | All, Ill Defined code for causes of death | low | 90+ | 6.2 (5.4 - 7.0) | 532.0 (464.0 - 600.0) |
| 2023 | Amyloidosis | high | 40-69 | 0.7 (-0.3 - 1.6) | 0.2 (-0.1 - 0.5) |
| 2023 | Amyloidosis | high | 70-79 | 1.1 (0.2 - 2.0) | 3.1 (0.6 - 5.7) |
| 2023 | Amyloidosis | high | 80-89 | 0.9 (0.3 - 1.5) | 11.0 (3.4 - 18.7) |
| 2023 | Amyloidosis | high | 90+ | 0.1 (-0.1 - 0.4) | 9.5 (-9.1 - 28.1) |
| 2023 | Amyloidosis | intermediate | 40-69 | 0.6 (0.3 - 1.0) | 0.5 (0.2 - 0.8) |
| 2023 | Amyloidosis | intermediate | 70-79 | 0.8 (0.4 - 1.1) | 3.3 (1.8 - 4.8) |
| 2023 | Amyloidosis | intermediate | 80-89 | 0.8 (0.5 - 1.1) | 13.3 (8.6 - 18.0) |
| 2023 | Amyloidosis | intermediate | 90+ | 0.1 (-0.0 - 0.2) | 8.2 (-1.1 - 17.4) |
| 2023 | Amyloidosis | low | 40-69 | 0.7 (0.1 - 1.3) | 1.2 (0.1 - 2.2) |
| 2023 | Amyloidosis | low | 70-79 | 1.1 (0.5 - 1.6) | 6.9 (3.5 - 10.4) |
| 2023 | Amyloidosis | low | 80-89 | 0.6 (0.4 - 0.9) | 13.0 (7.6 - 18.4) |
| 2023 | Amyloidosis | low | 90+ | 0.2 (0.0 - 0.3) | 13.6 (2.7 - 24.6) |
| 2023 | Anemia Unspecified | high | 70-79 | 0.6 (-0.1 - 1.2) | 1.6 (-0.2 - 3.4) |
| 2023 | Anemia Unspecified | high | 80-89 | 0.4 (0.0 - 0.9) | 5.5 (0.1 - 10.9) |
| 2023 | Anemia Unspecified | high | 90+ | 0.9 (0.2 - 1.6) | 66.5 (17.3 - 115.8) |
| 2023 | Anemia Unspecified | intermediate | 40-69 | 0.1 (-0.1 - 0.2) | 0.0 (-0.0 - 0.1) |
| 2023 | Anemia Unspecified | intermediate | 70-79 | 0.3 (0.1 - 0.5) | 1.4 (0.4 - 2.3) |
| 2023 | Anemia Unspecified | intermediate | 80-89 | 0.5 (0.3 - 0.8) | 8.6 (4.8 - 12.4) |
| 2023 | Anemia Unspecified | intermediate | 90+ | 1.5 (1.0 - 1.9) | 114.5 (79.9 - 149.2) |
| 2023 | Anemia Unspecified | low | 40-69 | 0.1 (-0.1 - 0.4) | 0.2 (-0.2 - 0.7) |
| 2023 | Anemia Unspecified | low | 70-79 | 0.3 (0.0 - 0.6) | 2.2 (0.3 - 4.1) |
| 2023 | Anemia Unspecified | low | 80-89 | 0.8 (0.5 - 1.1) | 16.0 (9.9 - 22.0) |
| 2023 | Anemia Unspecified | low | 90+ | 1.7 (1.3 - 2.1) | 143.2 (107.9 - 178.6) |
| 2023 | Arterial Embolism | high | 40-69 | 0.3 (-0.3 - 1.0) | 0.1 (-0.1 - 0.3) |
| 2023 | Arterial Embolism | high | 70-79 | 0.2 (-0.2 - 0.6) | 0.5 (-0.5 - 1.6) |
| 2023 | Arterial Embolism | high | 80-89 | 0.1 (-0.1 - 0.3) | 1.4 (-1.3 - 4.1) |
| 2023 | Arterial Embolism | intermediate | 40-69 | 0.3 (0.0 - 0.5) | 0.2 (0.0 - 0.4) |
| 2023 | Arterial Embolism | intermediate | 70-79 | 0.2 (0.0 - 0.4) | 1.0 (0.2 - 1.9) |
| 2023 | Arterial Embolism | intermediate | 80-89 | 0.2 (0.1 - 0.4) | 3.4 (1.1 - 5.8) |
| 2023 | Arterial Embolism | intermediate | 90+ | 0.1 (0.0 - 0.3) | 10.9 (0.2 - 21.6) |
| 2023 | Arterial Embolism | low | 40-69 | 0.7 (0.1 - 1.3) | 1.2 (0.1 - 2.2) |
| 2023 | Arterial Embolism | low | 70-79 | 0.3 (0.0 - 0.5) | 1.7 (0.0 - 3.4) |
| 2023 | Arterial Embolism | low | 80-89 | 0.1 (0.0 - 0.3) | 3.0 (0.4 - 5.5) |
| 2023 | Arterial Embolism | low | 90+ | 0.1 (0.0 - 0.2) | 9.1 (0.2 - 18.0) |
| 2023 | Assault by unspecified means | intermediate | under 40 | 0.3 (-0.3 - 0.9) | 0.0 (-0.0 - 0.1) |
| 2023 | Assault by unspecified means | intermediate | 40-69 | 0.1 (-0.1 - 0.2) | 0.0 (-0.0 - 0.1) |
| 2023 | Assault by unspecified means | low | 40-69 | 0.1 (-0.1 - 0.4) | 0.2 (-0.2 - 0.7) |
| 2023 | Assigned death to tobacco | intermediate | 40-69 | 0.2 (0.0 - 0.4) | 0.2 (0.0 - 0.4) |
| 2023 | Assigned death to tobacco | intermediate | 70-79 | 0.2 (0.0 - 0.4) | 1.0 (0.2 - 1.9) |
| 2023 | Assigned death to tobacco | intermediate | 80-89 | 0.1 (0.0 - 0.2) | 1.7 (0.0 - 3.4) |
| 2023 | Assigned death to tobacco | low | 70-79 | 0.2 (-0.0 - 0.4) | 1.3 (-0.2 - 2.8) |
| 2023 | Assigned death to tobacco | low | 80-89 | 0.1 (-0.0 - 0.2) | 1.8 (-0.2 - 3.8) |
| 2023 | Atherosclerosis | high | 40-69 | 0.3 (-0.3 - 1.0) | 0.1 (-0.1 - 0.3) |
| 2023 | Atherosclerosis | high | 70-79 | 1.3 (0.3 - 2.3) | 3.7 (1.0 - 6.4) |
| 2023 | Atherosclerosis | high | 80-89 | 1.6 (0.7 - 2.4) | 19.3 (9.2 - 29.5) |
| 2023 | Atherosclerosis | high | 90+ | 2.0 (1.0 - 2.9) | 142.5 (70.4 - 214.6) |
| 2023 | Atherosclerosis | intermediate | 40-69 | 1.2 (0.7 - 1.7) | 0.9 (0.5 - 1.4) |
| 2023 | Atherosclerosis | intermediate | 70-79 | 1.5 (1.0 - 2.0) | 6.6 (4.5 - 8.6) |
| 2023 | Atherosclerosis | intermediate | 80-89 | 1.4 (1.0 - 1.8) | 23.2 (17.0 - 29.4) |
| 2023 | Atherosclerosis | intermediate | 90+ | 1.5 (1.1 - 2.0) | 117.3 (82.2 - 152.3) |
| 2023 | Atherosclerosis | low | 40-69 | 0.8 (0.2 - 1.5) | 1.4 (0.3 - 2.5) |
| 2023 | Atherosclerosis | low | 70-79 | 1.0 (0.5 - 1.5) | 6.5 (3.2 - 9.8) |
| 2023 | Atherosclerosis | low | 80-89 | 1.3 (0.9 - 1.6) | 26.6 (18.8 - 34.4) |
| 2023 | Atherosclerosis | low | 90+ | 1.3 (1.0 - 1.7) | 113.7 (82.2 - 145.2) |
| 2023 | CKD due to diabetes Unspecified type | high | 70-79 | 0.4 (-0.1 - 0.9) | 1.0 (-0.4 - 2.5) |
| 2023 | CKD due to diabetes Unspecified type | high | 80-89 | 0.2 (-0.1 - 0.5) | 2.8 (-1.1 - 6.6) |
| 2023 | CKD due to diabetes Unspecified type | high | 90+ | 0.4 (-0.1 - 0.8) | 28.5 (-3.7 - 60.8) |
| 2023 | CKD due to diabetes Unspecified type | intermediate | 40-69 | 0.6 (0.3 - 1.0) | 0.5 (0.2 - 0.8) |
| 2023 | CKD due to diabetes Unspecified type | intermediate | 70-79 | 0.6 (0.3 - 0.9) | 2.6 (1.3 - 3.9) |
| 2023 | CKD due to diabetes Unspecified type | intermediate | 80-89 | 0.9 (0.6 - 1.2) | 15.0 (10.1 - 20.0) |
| 2023 | CKD due to diabetes Unspecified type | intermediate | 90+ | 0.5 (0.2 - 0.7) | 38.2 (18.2 - 58.2) |
| 2023 | CKD due to diabetes Unspecified type | low | 40-69 | 1.0 (0.3 - 1.7) | 1.6 (0.4 - 2.9) |
| 2023 | CKD due to diabetes Unspecified type | low | 70-79 | 1.0 (0.5 - 1.5) | 6.5 (3.2 - 9.8) |
| 2023 | CKD due to diabetes Unspecified type | low | 80-89 | 0.5 (0.3 - 0.7) | 10.1 (5.3 - 14.8) |
| 2023 | CKD due to diabetes Unspecified type | low | 90+ | 0.4 (0.2 - 0.6) | 34.1 (16.8 - 51.4) |
| 2023 | CNS Abscess | intermediate | 40-69 | 0.2 (-0.0 - 0.4) | 0.1 (-0.0 - 0.3) |
| 2023 | CNS Abscess | intermediate | 70-79 | 0.1 (-0.0 - 0.2) | 0.3 (-0.1 - 0.8) |
| 2023 | CNS Abscess | intermediate | 80-89 | 0.0 (-0.0 - 0.1) | 0.4 (-0.4 - 1.3) |
| 2023 | CNS Abscess | intermediate | 90+ | 0.0 (-0.0 - 0.1) | 2.7 (-2.6 - 8.1) |
| 2023 | CNS Abscess | low | 70-79 | 0.1 (-0.1 - 0.2) | 0.4 (-0.4 - 1.3) |
| 2023 | CNS Abscess | low | 80-89 | 0.0 (-0.0 - 0.1) | 0.6 (-0.6 - 1.8) |
| 2023 | CNS Fluid Diseases | intermediate | 80-89 | 0.1 (-0.0 - 0.1) | 0.9 (-0.3 - 2.1) |
| 2023 | CNS Fluid Diseases | low | 70-79 | 0.1 (-0.1 - 0.2) | 0.4 (-0.4 - 1.3) |
| 2023 | Cardiac rhythm disorders | high | under 40 | 1.0 (-1.0 - 3.0) | 0.0 (-0.0 - 0.1) |
| 2023 | Cardiac rhythm disorders | high | 40-69 | 1.3 (0.0 - 2.6) | 0.4 (0.0 - 0.8) |
| 2023 | Cardiac rhythm disorders | high | 70-79 | 1.9 (0.7 - 3.0) | 5.2 (2.0 - 8.5) |
| 2023 | Cardiac rhythm disorders | high | 80-89 | 1.1 (0.4 - 1.8) | 13.8 (5.3 - 22.4) |
| 2023 | Cardiac rhythm disorders | high | 90+ | 1.7 (0.8 - 2.6) | 123.5 (56.4 - 190.6) |
| 2023 | Cardiac rhythm disorders | intermediate | under 40 | 1.8 (0.4 - 3.2) | 0.2 (0.0 - 0.4) |
| 2023 | Cardiac rhythm disorders | intermediate | 40-69 | 0.8 (0.4 - 1.3) | 0.7 (0.3 - 1.0) |
| 2023 | Cardiac rhythm disorders | intermediate | 70-79 | 1.2 (0.8 - 1.6) | 5.2 (3.3 - 7.0) |
| 2023 | Cardiac rhythm disorders | intermediate | 80-89 | 1.8 (1.4 - 2.2) | 29.2 (22.3 - 36.2) |
| 2023 | Cardiac rhythm disorders | intermediate | 90+ | 1.8 (1.3 - 2.3) | 139.1 (100.9 - 177.2) |
| 2023 | Cardiac rhythm disorders | low | 40-69 | 1.5 (0.6 - 2.4) | 2.6 (1.1 - 4.1) |
| 2023 | Cardiac rhythm disorders | low | 70-79 | 1.1 (0.6 - 1.6) | 7.4 (3.9 - 10.9) |
| 2023 | Cardiac rhythm disorders | low | 80-89 | 1.5 (1.1 - 1.9) | 30.7 (22.4 - 39.1) |
| 2023 | Cardiac rhythm disorders | low | 90+ | 1.4 (1.0 - 1.8) | 120.5 (88.1 - 152.9) |
| 2023 | Cerebral Cysts | low | 80-89 | 0.0 (-0.0 - 0.1) | 0.6 (-0.6 - 1.8) |
| 2023 | Cerebral Palsy | high | under 40 | 2.0 (-0.8 - 4.8) | 0.1 (-0.0 - 0.2) |
| 2023 | Cerebral Palsy | intermediate | under 40 | 2.4 (0.7 - 4.0) | 0.3 (0.1 - 0.5) |
| 2023 | Cerebral Palsy | intermediate | 40-69 | 0.2 (-0.0 - 0.4) | 0.1 (-0.0 - 0.3) |
| 2023 | Cerebral Palsy | intermediate | 70-79 | 0.1 (-0.0 - 0.3) | 0.5 (-0.1 - 1.1) |
| 2023 | Cerebral Palsy | intermediate | 80-89 | 0.0 (-0.0 - 0.1) | 0.4 (-0.4 - 1.3) |
| 2023 | Cerebral Palsy | low | under 40 | 1.7 (-0.6 - 4.0) | 0.7 (-0.3 - 1.6) |
| 2023 | Cerebral Palsy | low | 40-69 | 0.8 (0.2 - 1.5) | 1.4 (0.3 - 2.5) |
| 2023 | Cerebral Palsy | low | 70-79 | 0.1 (-0.1 - 0.2) | 0.4 (-0.4 - 1.3) |
| 2023 | Cerebral Palsy | low | 80-89 | 0.1 (0.0 - 0.2) | 2.4 (0.0 - 4.7) |
| 2023 | Chronic lymphocytic leukemia by age | high | 70-79 | 0.4 (-0.1 - 0.9) | 1.0 (-0.4 - 2.5) |
| 2023 | Chronic lymphocytic leukemia by age | high | 80-89 | 1.2 (0.5 - 1.9) | 15.2 (6.2 - 24.2) |
| 2023 | Chronic lymphocytic leukemia by age | high | 90+ | 0.3 (-0.1 - 0.6) | 19.0 (-7.3 - 45.3) |
| 2023 | Chronic lymphocytic leukemia by age | intermediate | 40-69 | 0.2 (0.0 - 0.4) | 0.2 (0.0 - 0.4) |
| 2023 | Chronic lymphocytic leukemia by age | intermediate | 70-79 | 1.0 (0.6 - 1.4) | 4.5 (2.8 - 6.2) |
| 2023 | Chronic lymphocytic leukemia by age | intermediate | 80-89 | 0.9 (0.6 - 1.2) | 14.2 (9.3 - 19.0) |
| 2023 | Chronic lymphocytic leukemia by age | intermediate | 90+ | 0.4 (0.2 - 0.7) | 32.7 (14.2 - 51.2) |
| 2023 | Chronic lymphocytic leukemia by age | low | 70-79 | 0.9 (0.4 - 1.3) | 5.6 (2.6 - 8.7) |
| 2023 | Chronic lymphocytic leukemia by age | low | 80-89 | 0.7 (0.5 - 1.0) | 15.4 (9.5 - 21.3) |
| 2023 | Chronic lymphocytic leukemia by age | low | 90+ | 0.2 (0.1 - 0.4) | 18.2 (5.6 - 30.8) |
| 2023 | Chronic respiratory failure | high | 70-79 | 0.6 (-0.1 - 1.2) | 1.6 (-0.2 - 3.4) |
| 2023 | Chronic respiratory failure | high | 80-89 | 0.1 (-0.1 - 0.3) | 1.4 (-1.3 - 4.1) |
| 2023 | Chronic respiratory failure | high | 90+ | 0.4 (-0.1 - 0.8) | 28.5 (-3.7 - 60.8) |
| 2023 | Chronic respiratory failure | intermediate | 40-69 | 0.2 (-0.0 - 0.4) | 0.1 (-0.0 - 0.3) |
| 2023 | Chronic respiratory failure | intermediate | 70-79 | 0.2 (0.0 - 0.4) | 1.0 (0.2 - 1.9) |
| 2023 | Chronic respiratory failure | intermediate | 80-89 | 0.3 (0.2 - 0.5) | 5.6 (2.5 - 8.6) |
| 2023 | Chronic respiratory failure | intermediate | 90+ | 0.2 (0.0 - 0.4) | 16.4 (3.3 - 29.5) |
| 2023 | Chronic respiratory failure | low | 40-69 | 0.1 (-0.1 - 0.4) | 0.2 (-0.2 - 0.7) |
| 2023 | Chronic respiratory failure | low | 70-79 | 0.2 (-0.0 - 0.4) | 1.3 (-0.2 - 2.8) |
| 2023 | Chronic respiratory failure | low | 80-89 | 0.2 (0.1 - 0.4) | 4.7 (1.5 - 8.0) |
| 2023 | Chronic respiratory failure | low | 90+ | 0.2 (0.0 - 0.3) | 13.6 (2.7 - 24.6) |
| 2023 | Diabetes unspecified type | high | under 40 | 1.0 (-1.0 - 3.0) | 0.0 (-0.0 - 0.1) |
| 2023 | Diabetes unspecified type | high | 40-69 | 2.3 (0.6 - 4.0) | 0.7 (0.2 - 1.2) |
| 2023 | Diabetes unspecified type | high | 70-79 | 3.9 (2.3 - 5.6) | 11.0 (6.3 - 15.7) |
| 2023 | Diabetes unspecified type | high | 80-89 | 2.9 (1.8 - 4.0) | 35.9 (22.1 - 49.7) |
| 2023 | Diabetes unspecified type | high | 90+ | 1.3 (0.5 - 2.1) | 95.0 (36.2 - 153.9) |
| 2023 | Diabetes unspecified type | intermediate | under 40 | 0.9 (-0.1 - 1.9) | 0.1 (-0.0 - 0.2) |
| 2023 | Diabetes unspecified type | intermediate | 40-69 | 4.4 (3.4 - 5.4) | 3.5 (2.7 - 4.3) |
| 2023 | Diabetes unspecified type | intermediate | 70-79 | 4.6 (3.8 - 5.4) | 20.2 (16.5 - 23.9) |
| 2023 | Diabetes unspecified type | intermediate | 80-89 | 3.2 (2.6 - 3.8) | 52.0 (42.7 - 61.3) |
| 2023 | Diabetes unspecified type | intermediate | 90+ | 2.4 (1.8 - 2.9) | 185.5 (141.4 - 229.5) |
| 2023 | Diabetes unspecified type | low | under 40 | 0.8 (-0.8 - 2.5) | 0.3 (-0.3 - 1.0) |
| 2023 | Diabetes unspecified type | low | 40-69 | 5.3 (3.7 - 7.0) | 9.2 (6.3 - 12.0) |
| 2023 | Diabetes unspecified type | low | 70-79 | 4.7 (3.6 - 5.7) | 30.8 (23.7 - 38.0) |
| 2023 | Diabetes unspecified type | low | 80-89 | 4.0 (3.4 - 4.7) | 84.0 (70.2 - 97.8) |
| 2023 | Diabetes unspecified type | low | 90+ | 2.8 (2.3 - 3.4) | 243.3 (197.2 - 289.3) |
| 2023 | Exposure to unspecified factor X59 | high | 40-69 | 1.3 (0.0 - 2.6) | 0.4 (0.0 - 0.8) |
| 2023 | Exposure to unspecified factor X59 | high | 70-79 | 3.9 (2.3 - 5.6) | 11.0 (6.3 - 15.7) |
| 2023 | Exposure to unspecified factor X59 | high | 80-89 | 5.5 (4.1 - 7.0) | 69.1 (49.9 - 88.2) |
| 2023 | Exposure to unspecified factor X59 | high | 90+ | 7.2 (5.4 - 9.0) | 522.6 (384.8 - 660.3) |
| 2023 | Exposure to unspecified factor X59 | intermediate | under 40 | 0.3 (-0.3 - 0.9) | 0.0 (-0.0 - 0.1) |
| 2023 | Exposure to unspecified factor X59 | intermediate | 40-69 | 1.6 (1.0 - 2.2) | 1.3 (0.8 - 1.7) |
| 2023 | Exposure to unspecified factor X59 | intermediate | 70-79 | 3.3 (2.6 - 4.0) | 14.5 (11.4 - 17.6) |
| 2023 | Exposure to unspecified factor X59 | intermediate | 80-89 | 6.2 (5.5 - 7.0) | 101.4 (88.5 - 114.3) |
| 2023 | Exposure to unspecified factor X59 | intermediate | 90+ | 5.3 (4.5 - 6.2) | 414.6 (348.8 - 480.3) |
| 2023 | Exposure to unspecified factor X59 | low | 40-69 | 1.5 (0.6 - 2.4) | 2.6 (1.1 - 4.1) |
| 2023 | Exposure to unspecified factor X59 | low | 70-79 | 4.3 (3.3 - 5.4) | 28.7 (21.7 - 35.6) |
| 2023 | Exposure to unspecified factor X59 | low | 80-89 | 5.6 (4.8 - 6.4) | 116.5 (100.2 - 132.7) |
| 2023 | Exposure to unspecified factor X59 | low | 90+ | 5.9 (5.2 - 6.7) | 509.3 (442.8 - 575.8) |
| 2023 | External Causes UDI, type unspecified | high | 40-69 | 0.7 (-0.3 - 1.6) | 0.2 (-0.1 - 0.5) |
| 2023 | External Causes UDI, type unspecified | high | 70-79 | 0.9 (0.1 - 1.7) | 2.6 (0.3 - 4.9) |
| 2023 | External Causes UDI, type unspecified | high | 80-89 | 0.1 (-0.1 - 0.3) | 1.4 (-1.3 - 4.1) |
| 2023 | External Causes UDI, type unspecified | high | 90+ | 0.4 (-0.1 - 0.8) | 28.5 (-3.7 - 60.8) |
| 2023 | External Causes UDI, type unspecified | intermediate | under 40 | 1.8 (0.4 - 3.2) | 0.2 (0.0 - 0.4) |
| 2023 | External Causes UDI, type unspecified | intermediate | 40-69 | 0.8 (0.4 - 1.3) | 0.7 (0.3 - 1.0) |
| 2023 | External Causes UDI, type unspecified | intermediate | 70-79 | 0.5 (0.2 - 0.7) | 2.1 (0.9 - 3.2) |
| 2023 | External Causes UDI, type unspecified | intermediate | 80-89 | 0.1 (0.0 - 0.2) | 2.1 (0.3 - 4.0) |
| 2023 | External Causes UDI, type unspecified | intermediate | 90+ | 0.1 (-0.0 - 0.2) | 8.2 (-1.1 - 17.4) |
| 2023 | External Causes UDI, type unspecified | low | under 40 | 0.8 (-0.8 - 2.5) | 0.3 (-0.3 - 1.0) |
| 2023 | External Causes UDI, type unspecified | low | 40-69 | 1.4 (0.5 - 2.2) | 2.3 (0.9 - 3.8) |
| 2023 | External Causes UDI, type unspecified | low | 70-79 | 0.3 (0.0 - 0.5) | 1.7 (0.0 - 3.4) |
| 2023 | External Causes UDI, type unspecified | low | 80-89 | 0.1 (-0.0 - 0.2) | 1.8 (-0.2 - 3.8) |
| 2023 | External Causes UDI, type unspecified | low | 90+ | 0.2 (0.0 - 0.3) | 15.9 (4.1 - 27.7) |
| 2023 | Eye Unspecified Site Cancer | high | 40-69 | 0.7 (-0.3 - 1.6) | 0.2 (-0.1 - 0.5) |
| 2023 | Eye Unspecified Site Cancer | high | 70-79 | 0.2 (-0.2 - 0.6) | 0.5 (-0.5 - 1.6) |
| 2023 | Eye Unspecified Site Cancer | intermediate | 40-69 | 0.2 (0.0 - 0.4) | 0.2 (0.0 - 0.4) |
| 2023 | Eye Unspecified Site Cancer | intermediate | 70-79 | 0.1 (-0.0 - 0.2) | 0.3 (-0.1 - 0.8) |
| 2023 | Eye Unspecified Site Cancer | low | 40-69 | 0.5 (0.0 - 1.1) | 0.9 (0.0 - 1.9) |
| 2023 | Female pelvic inflammatory diseases | intermediate | 40-69 | 0.1 (-0.1 - 0.2) | 0.0 (-0.0 - 0.1) |
| 2023 | Female pelvic inflammatory diseases | intermediate | 80-89 | 0.0 (-0.0 - 0.1) | 0.4 (-0.4 - 1.3) |
| 2023 | Female pelvic inflammatory diseases | low | 90+ | 0.0 (-0.0 - 0.1) | 2.3 (-2.2 - 6.7) |
| 2023 | Fistula | high | 70-79 | 0.4 (-0.1 - 0.9) | 1.0 (-0.4 - 2.5) |
| 2023 | Fistula | intermediate | 40-69 | 0.1 (-0.1 - 0.2) | 0.0 (-0.0 - 0.1) |
| 2023 | Fistula | intermediate | 80-89 | 0.0 (-0.0 - 0.1) | 0.4 (-0.4 - 1.3) |
| 2023 | Fistula | low | 40-69 | 0.1 (-0.1 - 0.4) | 0.2 (-0.2 - 0.7) |
| 2023 | Fistula | low | 70-79 | 0.1 (-0.1 - 0.3) | 0.9 (-0.3 - 2.1) |
| 2023 | Fistula | low | 80-89 | 0.1 (-0.0 - 0.2) | 1.8 (-0.2 - 3.8) |
| 2023 | Fluid, Electrolyte, Acid Base Disorders | high | 70-79 | 0.6 (-0.1 - 1.2) | 1.6 (-0.2 - 3.4) |
| 2023 | Fluid, Electrolyte, Acid Base Disorders | high | 80-89 | 0.8 (0.2 - 1.4) | 9.7 (2.5 - 16.8) |
| 2023 | Fluid, Electrolyte, Acid Base Disorders | high | 90+ | 0.7 (0.1 - 1.2) | 47.5 (5.9 - 89.1) |
| 2023 | Fluid, Electrolyte, Acid Base Disorders | intermediate | under 40 | 0.6 (-0.2 - 1.4) | 0.1 (-0.0 - 0.2) |
| 2023 | Fluid, Electrolyte, Acid Base Disorders | intermediate | 40-69 | 0.8 (0.4 - 1.3) | 0.7 (0.3 - 1.0) |
| 2023 | Fluid, Electrolyte, Acid Base Disorders | intermediate | 70-79 | 0.8 (0.4 - 1.1) | 3.3 (1.8 - 4.8) |
| 2023 | Fluid, Electrolyte, Acid Base Disorders | intermediate | 80-89 | 0.6 (0.3 - 0.8) | 9.5 (5.5 - 13.4) |
| 2023 | Fluid, Electrolyte, Acid Base Disorders | intermediate | 90+ | 0.9 (0.5 - 1.2) | 68.2 (41.5 - 94.9) |
| 2023 | Fluid, Electrolyte, Acid Base Disorders | low | 40-69 | 0.7 (0.1 - 1.3) | 1.2 (0.1 - 2.2) |
| 2023 | Fluid, Electrolyte, Acid Base Disorders | low | 70-79 | 0.7 (0.3 - 1.1) | 4.3 (1.7 - 7.0) |
| 2023 | Fluid, Electrolyte, Acid Base Disorders | low | 80-89 | 0.9 (0.5 - 1.2) | 17.7 (11.4 - 24.1) |
| 2023 | Fluid, Electrolyte, Acid Base Disorders | low | 90+ | 0.4 (0.2 - 0.7) | 38.7 (20.3 - 57.0) |
| 2023 | HIV correction for Aspergillosis | intermediate | 70-79 | 0.0 (-0.0 - 0.1) | 0.2 (-0.2 - 0.5) |
| 2023 | HIV correction for Aspergillosis | low | 80-89 | 0.0 (-0.0 - 0.1) | 0.6 (-0.6 - 1.8) |
| 2023 | HIV correction for Candidiasis | high | 70-79 | 0.2 (-0.2 - 0.6) | 0.5 (-0.5 - 1.6) |
| 2023 | HIV correction for Candidiasis | intermediate | 40-69 | 0.1 (-0.1 - 0.2) | 0.0 (-0.0 - 0.1) |
| 2023 | HIV correction for Candidiasis | intermediate | 70-79 | 0.0 (-0.0 - 0.1) | 0.2 (-0.2 - 0.5) |
| 2023 | HIV correction for Candidiasis | intermediate | 80-89 | 0.1 (0.0 - 0.2) | 2.1 (0.3 - 4.0) |
| 2023 | HIV correction for Candidiasis | low | 70-79 | 0.1 (-0.1 - 0.2) | 0.4 (-0.4 - 1.3) |
| 2023 | HIV correction for Candidiasis | low | 80-89 | 0.1 (-0.0 - 0.1) | 1.2 (-0.5 - 2.8) |
| 2023 | HIV correction for Immunodeficiency antibody | high | 80-89 | 0.1 (-0.1 - 0.3) | 1.4 (-1.3 - 4.1) |
| 2023 | HIV correction for Immunodeficiency antibody | intermediate | 70-79 | 0.1 (-0.0 - 0.2) | 0.3 (-0.1 - 0.8) |
| 2023 | HIV correction for Immunodeficiency antibody | low | 70-79 | 0.1 (-0.1 - 0.2) | 0.4 (-0.4 - 1.3) |
| 2023 | HIV correction for Immunodeficiency cell | high | under 40 | 1.0 (-1.0 - 3.0) | 0.0 (-0.0 - 0.1) |
| 2023 | HIV correction for Immunodeficiency other | high | under 40 | 1.0 (-1.0 - 3.0) | 0.0 (-0.0 - 0.1) |
| 2023 | HIV correction for Immunodeficiency other | high | 80-89 | 0.1 (-0.1 - 0.3) | 1.4 (-1.3 - 4.1) |
| 2023 | HIV correction for Immunodeficiency other | intermediate | 40-69 | 0.1 (-0.0 - 0.3) | 0.1 (-0.0 - 0.2) |
| 2023 | HIV correction for Immunodeficiency other | intermediate | 70-79 | 0.2 (0.0 - 0.3) | 0.7 (0.0 - 1.4) |
| 2023 | HIV correction for Immunodeficiency other | intermediate | 80-89 | 0.1 (-0.0 - 0.1) | 0.9 (-0.3 - 2.1) |
| 2023 | HIV correction for Immunodeficiency other | intermediate | 90+ | 0.1 (-0.0 - 0.2) | 5.5 (-2.1 - 13.0) |
| 2023 | HIV correction for Immunodeficiency other | low | 40-69 | 0.1 (-0.1 - 0.4) | 0.2 (-0.2 - 0.7) |
| 2023 | HIV correction for Immunodeficiency other | low | 80-89 | 0.0 (-0.0 - 0.1) | 0.6 (-0.6 - 1.8) |
| 2023 | HIV correction for Immunodeficiency other | low | 90+ | 0.1 (-0.0 - 0.1) | 4.5 (-1.8 - 10.8) |
| 2023 | HIV correction for Other Mycobacterial infection | intermediate | 70-79 | 0.0 (-0.0 - 0.1) | 0.2 (-0.2 - 0.5) |
| 2023 | HIV correction for Other Mycobacterial infection | low | 40-69 | 0.1 (-0.1 - 0.4) | 0.2 (-0.2 - 0.7) |
| 2023 | HIV correction for Unspecified mycosis | high | 80-89 | 0.1 (-0.1 - 0.3) | 1.4 (-1.3 - 4.1) |
| 2023 | HIV correction for Unspecified mycosis | intermediate | 40-69 | 0.1 (-0.1 - 0.2) | 0.0 (-0.0 - 0.1) |
| 2023 | HIV correction for Unspecified mycosis | intermediate | 70-79 | 0.0 (-0.0 - 0.1) | 0.2 (-0.2 - 0.5) |
| 2023 | HIV correction for Unspecified mycosis | intermediate | 80-89 | 0.0 (-0.0 - 0.1) | 0.4 (-0.4 - 1.3) |
| 2023 | HIV correction for Unspecified mycosis | low | 90+ | 0.0 (-0.0 - 0.1) | 2.3 (-2.2 - 6.7) |
| 2023 | HIV correction for Zygomycosis | intermediate | 40-69 | 0.1 (-0.1 - 0.2) | 0.0 (-0.0 - 0.1) |
| 2023 | Haemophilus influenza infection, unspecified site | intermediate | under 40 | 0.3 (-0.3 - 0.9) | 0.0 (-0.0 - 0.1) |
| 2023 | Head and Neck Cancer | high | 80-89 | 0.2 (-0.1 - 0.5) | 2.8 (-1.1 - 6.6) |
| 2023 | Head and Neck Cancer | intermediate | 40-69 | 0.1 (-0.1 - 0.2) | 0.0 (-0.0 - 0.1) |
| 2023 | Head and Neck Cancer | intermediate | 70-79 | 0.1 (-0.0 - 0.3) | 0.5 (-0.1 - 1.1) |
| 2023 | Head and Neck Cancer | intermediate | 80-89 | 0.1 (-0.0 - 0.1) | 0.9 (-0.3 - 2.1) |
| 2023 | Head and Neck Cancer | low | 80-89 | 0.1 (0.0 - 0.2) | 2.4 (0.0 - 4.7) |
| 2023 | Head and Neck Cancer | low | 90+ | 0.1 (-0.0 - 0.2) | 6.8 (-0.9 - 14.5) |
| 2023 | Heart failure unspecified right or left | high | 40-69 | 1.3 (0.0 - 2.6) | 0.4 (0.0 - 0.8) |
| 2023 | Heart failure unspecified right or left | high | 70-79 | 8.4 (6.1 - 10.8) | 23.6 (16.7 - 30.5) |
| 2023 | Heart failure unspecified right or left | high | 80-89 | 14.4 (12.1 - 16.7) | 179.6 (148.7 - 210.4) |
| 2023 | Heart failure unspecified right or left | high | 90+ | 18.8 (16.1 - 21.6) | 1368.2 (1146.2 - 1590.1) |
| 2023 | Heart failure unspecified right or left | intermediate | 40-69 | 1.9 (1.2 - 2.5) | 1.5 (1.0 - 2.0) |
| 2023 | Heart failure unspecified right or left | intermediate | 70-79 | 8.7 (7.6 - 9.8) | 38.2 (33.1 - 43.2) |
| 2023 | Heart failure unspecified right or left | intermediate | 80-89 | 13.9 (12.8 - 15.0) | 226.0 (206.7 - 245.3) |
| 2023 | Heart failure unspecified right or left | intermediate | 90+ | 20.0 (18.6 - 21.5) | 1557.3 (1430.6 - 1684.0) |
| 2023 | Heart failure unspecified right or left | low | 40-69 | 1.8 (0.8 - 2.7) | 3.1 (1.4 - 4.7) |
| 2023 | Heart failure unspecified right or left | low | 70-79 | 8.3 (6.9 - 9.7) | 54.7 (45.2 - 64.3) |
| 2023 | Heart failure unspecified right or left | low | 80-89 | 15.8 (14.6 - 17.0) | 329.3 (302.0 - 356.7) |
| 2023 | Heart failure unspecified right or left | low | 90+ | 20.5 (19.2 - 21.8) | 1762.0 (1639.0 - 1885.0) |
| 2023 | Hepatic Failure | high | 40-69 | 1.3 (0.0 - 2.6) | 0.4 (0.0 - 0.8) |
| 2023 | Hepatic Failure | high | 70-79 | 0.7 (0.0 - 1.5) | 2.1 (0.0 - 4.2) |
| 2023 | Hepatic Failure | high | 80-89 | 0.6 (0.1 - 1.0) | 6.9 (0.9 - 13.0) |
| 2023 | Hepatic Failure | high | 90+ | 0.3 (-0.1 - 0.6) | 19.0 (-7.3 - 45.3) |
| 2023 | Hepatic Failure | intermediate | 40-69 | 1.1 (0.6 - 1.6) | 0.9 (0.5 - 1.3) |
| 2023 | Hepatic Failure | intermediate | 70-79 | 0.7 (0.4 - 1.0) | 3.1 (1.7 - 4.5) |
| 2023 | Hepatic Failure | intermediate | 80-89 | 0.4 (0.2 - 0.7) | 7.3 (3.8 - 10.8) |
| 2023 | Hepatic Failure | intermediate | 90+ | 0.2 (0.1 - 0.4) | 19.1 (4.9 - 33.2) |
| 2023 | Hepatic Failure | low | 40-69 | 0.5 (0.0 - 1.1) | 0.9 (0.0 - 1.9) |
| 2023 | Hepatic Failure | low | 70-79 | 0.3 (0.0 - 0.6) | 2.2 (0.3 - 4.1) |
| 2023 | Hepatic Failure | low | 80-89 | 0.4 (0.2 - 0.6) | 8.3 (3.9 - 12.6) |
| 2023 | Hepatic Failure | low | 90+ | 0.1 (-0.0 - 0.2) | 6.8 (-0.9 - 14.5) |
| 2023 | Hepatitis Unspecified | intermediate | 40-69 | 0.1 (-0.1 - 0.2) | 0.0 (-0.0 - 0.1) |
| 2023 | Hypertension | high | 40-69 | 1.0 (-0.1 - 2.1) | 0.3 (-0.0 - 0.6) |
| 2023 | Hypertension | high | 70-79 | 2.6 (1.3 - 4.0) | 7.3 (3.5 - 11.2) |
| 2023 | Hypertension | high | 80-89 | 4.0 (2.7 - 5.3) | 49.7 (33.5 - 66.0) |
| 2023 | Hypertension | high | 90+ | 5.0 (3.4 - 6.5) | 361.0 (246.5 - 475.6) |
| 2023 | Hypertension | intermediate | 40-69 | 1.8 (1.2 - 2.4) | 1.4 (0.9 - 1.9) |
| 2023 | Hypertension | intermediate | 70-79 | 2.9 (2.3 - 3.6) | 12.8 (9.9 - 15.7) |
| 2023 | Hypertension | intermediate | 80-89 | 3.5 (2.9 - 4.1) | 57.1 (47.4 - 66.9) |
| 2023 | Hypertension | intermediate | 90+ | 5.5 (4.7 - 6.3) | 428.2 (361.4 - 495.0) |
| 2023 | Hypertension | low | 40-69 | 1.5 (0.6 - 2.4) | 2.6 (1.1 - 4.1) |
| 2023 | Hypertension | low | 70-79 | 2.6 (1.8 - 3.4) | 17.4 (12.0 - 22.7) |
| 2023 | Hypertension | low | 80-89 | 4.1 (3.5 - 4.8) | 85.7 (71.8 - 99.7) |
| 2023 | Hypertension | low | 90+ | 5.7 (4.9 - 6.4) | 486.5 (421.5 - 551.6) |
| 2023 | Intermediate cause for CNS | high | under 40 | 2.0 (-0.8 - 4.8) | 0.1 (-0.0 - 0.2) |
| 2023 | Intermediate cause for CNS | high | 40-69 | 0.7 (-0.3 - 1.6) | 0.2 (-0.1 - 0.5) |
| 2023 | Intermediate cause for CNS | high | 70-79 | 0.6 (-0.1 - 1.2) | 1.6 (-0.2 - 3.4) |
| 2023 | Intermediate cause for CNS | high | 80-89 | 0.6 (0.1 - 1.0) | 6.9 (0.9 - 13.0) |
| 2023 | Intermediate cause for CNS | high | 90+ | 0.3 (-0.1 - 0.6) | 19.0 (-7.3 - 45.3) |
| 2023 | Intermediate cause for CNS | intermediate | under 40 | 0.3 (-0.3 - 0.9) | 0.0 (-0.0 - 0.1) |
| 2023 | Intermediate cause for CNS | intermediate | 40-69 | 0.5 (0.2 - 0.8) | 0.4 (0.1 - 0.7) |
| 2023 | Intermediate cause for CNS | intermediate | 70-79 | 0.6 (0.3 - 0.8) | 2.4 (1.2 - 3.7) |
| 2023 | Intermediate cause for CNS | intermediate | 80-89 | 0.5 (0.3 - 0.7) | 8.2 (4.5 - 11.8) |
| 2023 | Intermediate cause for CNS | intermediate | 90+ | 0.1 (-0.0 - 0.2) | 5.5 (-2.1 - 13.0) |
| 2023 | Intermediate cause for CNS | low | under 40 | 1.7 (-0.6 - 4.0) | 0.7 (-0.3 - 1.6) |
| 2023 | Intermediate cause for CNS | low | 40-69 | 0.7 (0.1 - 1.3) | 1.2 (0.1 - 2.2) |
| 2023 | Intermediate cause for CNS | low | 70-79 | 0.5 (0.1 - 0.8) | 3.0 (0.8 - 5.3) |
| 2023 | Intermediate cause for CNS | low | 80-89 | 0.1 (0.0 - 0.2) | 2.4 (0.0 - 4.7) |
| 2023 | Intermediate cause for CNS | low | 90+ | 0.0 (-0.0 - 0.1) | 2.3 (-2.2 - 6.7) |
| 2023 | Left heart failure | high | 70-79 | 0.2 (-0.2 - 0.6) | 0.5 (-0.5 - 1.6) |
| 2023 | Left heart failure | high | 80-89 | 1.4 (0.7 - 2.2) | 18.0 (8.2 - 27.7) |
| 2023 | Left heart failure | high | 90+ | 1.0 (0.3 - 1.8) | 76.0 (23.4 - 128.7) |
| 2023 | Left heart failure | intermediate | 40-69 | 0.3 (0.1 - 0.6) | 0.3 (0.1 - 0.5) |
| 2023 | Left heart failure | intermediate | 70-79 | 0.8 (0.5 - 1.2) | 3.6 (2.1 - 5.2) |
| 2023 | Left heart failure | intermediate | 80-89 | 0.8 (0.5 - 1.0) | 12.5 (7.9 - 17.0) |
| 2023 | Left heart failure | intermediate | 90+ | 1.0 (0.6 - 1.3) | 76.4 (48.1 - 104.6) |
| 2023 | Left heart failure | low | 40-69 | 0.4 (-0.1 - 0.9) | 0.7 (-0.1 - 1.5) |
| 2023 | Left heart failure | low | 70-79 | 0.7 (0.3 - 1.1) | 4.8 (2.0 - 7.6) |
| 2023 | Left heart failure | low | 80-89 | 1.0 (0.7 - 1.3) | 20.7 (13.8 - 27.6) |
| 2023 | Left heart failure | low | 90+ | 0.8 (0.5 - 1.1) | 68.2 (43.8 - 92.6) |
| 2023 | Liver Abscess | high | 80-89 | 0.1 (-0.1 - 0.3) | 1.4 (-1.3 - 4.1) |
| 2023 | Liver Abscess | intermediate | 70-79 | 0.1 (-0.0 - 0.2) | 0.3 (-0.1 - 0.8) |
| 2023 | Liver Abscess | intermediate | 80-89 | 0.1 (0.0 - 0.2) | 1.7 (0.0 - 3.4) |
| 2023 | Liver Abscess | intermediate | 90+ | 0.0 (-0.0 - 0.1) | 2.7 (-2.6 - 8.1) |
| 2023 | Liver Abscess | low | 80-89 | 0.0 (-0.0 - 0.1) | 0.6 (-0.6 - 1.8) |
| 2023 | Lymphoid leukemia unspecified by age | intermediate | 80-89 | 0.1 (-0.0 - 0.1) | 0.9 (-0.3 - 2.1) |
| 2023 | Lymphoid leukemia unspecified by age | low | 80-89 | 0.1 (-0.0 - 0.1) | 1.2 (-0.5 - 2.8) |
| 2023 | MDS not classified | high | 70-79 | 0.2 (-0.2 - 0.6) | 0.5 (-0.5 - 1.6) |
| 2023 | MDS not classified | intermediate | 40-69 | 0.1 (-0.0 - 0.3) | 0.1 (-0.0 - 0.2) |
| 2023 | MDS not classified | intermediate | 70-79 | 0.0 (-0.0 - 0.1) | 0.2 (-0.2 - 0.5) |
| 2023 | MDS not classified | intermediate | 80-89 | 0.0 (-0.0 - 0.1) | 0.4 (-0.4 - 1.3) |
| 2023 | MDS not classified | low | 80-89 | 0.1 (-0.0 - 0.1) | 1.2 (-0.5 - 2.8) |
| 2023 | Mental Disorders | high | 40-69 | 0.3 (-0.3 - 1.0) | 0.1 (-0.1 - 0.3) |
| 2023 | Mental Disorders | high | 70-79 | 1.3 (0.3 - 2.3) | 3.7 (1.0 - 6.4) |
| 2023 | Mental Disorders | high | 80-89 | 0.8 (0.2 - 1.4) | 9.7 (2.5 - 16.8) |
| 2023 | Mental Disorders | high | 90+ | 0.5 (0.0 - 1.0) | 38.0 (0.8 - 75.2) |
| 2023 | Mental Disorders | intermediate | under 40 | 0.6 (-0.2 - 1.4) | 0.1 (-0.0 - 0.2) |
| 2023 | Mental Disorders | intermediate | 40-69 | 0.6 (0.3 - 1.0) | 0.5 (0.2 - 0.8) |
| 2023 | Mental Disorders | intermediate | 70-79 | 0.8 (0.4 - 1.1) | 3.3 (1.8 - 4.8) |
| 2023 | Mental Disorders | intermediate | 80-89 | 0.6 (0.4 - 0.9) | 10.3 (6.2 - 14.4) |
| 2023 | Mental Disorders | intermediate | 90+ | 0.7 (0.4 - 1.0) | 51.8 (28.5 - 75.1) |
| 2023 | Mental Disorders | low | 40-69 | 2.2 (1.1 - 3.2) | 3.8 (1.9 - 5.6) |
| 2023 | Mental Disorders | low | 70-79 | 2.0 (1.3 - 2.7) | 13.0 (8.4 - 17.7) |
| 2023 | Mental Disorders | low | 80-89 | 0.9 (0.6 - 1.2) | 18.3 (11.9 - 24.8) |
| 2023 | Mental Disorders | low | 90+ | 0.4 (0.2 - 0.6) | 34.1 (16.8 - 51.4) |
| 2023 | Myocardial Degeneration | intermediate | 70-79 | 0.1 (-0.0 - 0.2) | 0.3 (-0.1 - 0.8) |
| 2023 | Myocardial Degeneration | intermediate | 90+ | 0.0 (-0.0 - 0.1) | 2.7 (-2.6 - 8.1) |
| 2023 | Non-follicular lymphoma, unspecified | high | 40-69 | 3.3 (1.3 - 5.3) | 1.0 (0.4 - 1.5) |
| 2023 | Non-follicular lymphoma, unspecified | high | 70-79 | 3.4 (1.8 - 4.9) | 9.4 (5.1 - 13.8) |
| 2023 | Non-follicular lymphoma, unspecified | high | 80-89 | 1.8 (0.9 - 2.6) | 22.1 (11.3 - 32.9) |
| 2023 | Non-follicular lymphoma, unspecified | high | 90+ | 0.5 (0.0 - 1.0) | 38.0 (0.8 - 75.2) |
| 2023 | Non-follicular lymphoma, unspecified | intermediate | under 40 | 0.3 (-0.3 - 0.9) | 0.0 (-0.0 - 0.1) |
| 2023 | Non-follicular lymphoma, unspecified | intermediate | 40-69 | 1.2 (0.7 - 1.7) | 0.9 (0.5 - 1.4) |
| 2023 | Non-follicular lymphoma, unspecified | intermediate | 70-79 | 1.7 (1.2 - 2.2) | 7.4 (5.2 - 9.6) |
| 2023 | Non-follicular lymphoma, unspecified | intermediate | 80-89 | 1.6 (1.2 - 2.0) | 26.2 (19.6 - 32.8) |
| 2023 | Non-follicular lymphoma, unspecified | intermediate | 90+ | 0.6 (0.3 - 0.9) | 49.1 (26.4 - 71.8) |
| 2023 | Non-follicular lymphoma, unspecified | low | 40-69 | 1.8 (0.8 - 2.7) | 3.1 (1.4 - 4.7) |
| 2023 | Non-follicular lymphoma, unspecified | low | 70-79 | 1.8 (1.2 - 2.5) | 12.2 (7.7 - 16.7) |
| 2023 | Non-follicular lymphoma, unspecified | low | 80-89 | 1.1 (0.8 - 1.5) | 23.1 (15.8 - 30.3) |
| 2023 | Non-follicular lymphoma, unspecified | low | 90+ | 0.2 (0.1 - 0.4) | 20.5 (7.1 - 33.8) |
| 2023 | Osteomyelitis | high | 40-69 | 0.3 (-0.3 - 1.0) | 0.1 (-0.1 - 0.3) |
| 2023 | Osteomyelitis | high | 80-89 | 0.1 (-0.1 - 0.3) | 1.4 (-1.3 - 4.1) |
| 2023 | Osteomyelitis | high | 90+ | 0.1 (-0.1 - 0.4) | 9.5 (-9.1 - 28.1) |
| 2023 | Osteomyelitis | intermediate | 40-69 | 0.1 (-0.0 - 0.3) | 0.1 (-0.0 - 0.2) |
| 2023 | Osteomyelitis | intermediate | 70-79 | 0.1 (-0.0 - 0.2) | 0.3 (-0.1 - 0.8) |
| 2023 | Osteomyelitis | intermediate | 80-89 | 0.2 (0.0 - 0.3) | 3.0 (0.8 - 5.2) |
| 2023 | Osteomyelitis | intermediate | 90+ | 0.1 (-0.0 - 0.2) | 8.2 (-1.1 - 17.4) |
| 2023 | Osteomyelitis | low | 40-69 | 0.1 (-0.1 - 0.4) | 0.2 (-0.2 - 0.7) |
| 2023 | Osteomyelitis | low | 70-79 | 0.2 (-0.0 - 0.4) | 1.3 (-0.2 - 2.8) |
| 2023 | Osteomyelitis | low | 80-89 | 0.2 (0.0 - 0.3) | 3.5 (0.7 - 6.4) |
| 2023 | Osteomyelitis | low | 90+ | 0.2 (0.0 - 0.3) | 13.6 (2.7 - 24.6) |
| 2023 | Peritonitis & Acute Abdomen | high | 40-69 | 0.3 (-0.3 - 1.0) | 0.1 (-0.1 - 0.3) |
| 2023 | Peritonitis & Acute Abdomen | high | 70-79 | 0.4 (-0.1 - 0.9) | 1.0 (-0.4 - 2.5) |
| 2023 | Peritonitis & Acute Abdomen | high | 80-89 | 0.1 (-0.1 - 0.3) | 1.4 (-1.3 - 4.1) |
| 2023 | Peritonitis & Acute Abdomen | intermediate | under 40 | 0.3 (-0.3 - 0.9) | 0.0 (-0.0 - 0.1) |
| 2023 | Peritonitis & Acute Abdomen | intermediate | 40-69 | 0.3 (0.0 - 0.5) | 0.2 (0.0 - 0.4) |
| 2023 | Peritonitis & Acute Abdomen | intermediate | 70-79 | 0.3 (0.1 - 0.5) | 1.2 (0.3 - 2.1) |
| 2023 | Peritonitis & Acute Abdomen | intermediate | 80-89 | 0.3 (0.1 - 0.5) | 5.2 (2.2 - 8.1) |
| 2023 | Peritonitis & Acute Abdomen | intermediate | 90+ | 0.3 (0.1 - 0.5) | 21.8 (6.7 - 36.9) |
| 2023 | Peritonitis & Acute Abdomen | low | 40-69 | 0.5 (0.0 - 1.1) | 0.9 (0.0 - 1.9) |
| 2023 | Peritonitis & Acute Abdomen | low | 70-79 | 0.3 (0.0 - 0.5) | 1.7 (0.0 - 3.4) |
| 2023 | Peritonitis & Acute Abdomen | low | 80-89 | 0.3 (0.1 - 0.5) | 5.9 (2.2 - 9.6) |
| 2023 | Peritonitis & Acute Abdomen | low | 90+ | 0.2 (0.0 - 0.3) | 15.9 (4.1 - 27.7) |
| 2023 | Pleurisy, Pyothorax | high | under 40 | 1.0 (-1.0 - 3.0) | 0.0 (-0.0 - 0.1) |
| 2023 | Pleurisy, Pyothorax | high | 40-69 | 0.3 (-0.3 - 1.0) | 0.1 (-0.1 - 0.3) |
| 2023 | Pleurisy, Pyothorax | high | 70-79 | 0.6 (-0.1 - 1.2) | 1.6 (-0.2 - 3.4) |
| 2023 | Pleurisy, Pyothorax | high | 80-89 | 0.9 (0.3 - 1.5) | 11.0 (3.4 - 18.7) |
| 2023 | Pleurisy, Pyothorax | high | 90+ | 0.4 (-0.1 - 0.8) | 28.5 (-3.7 - 60.8) |
| 2023 | Pleurisy, Pyothorax | intermediate | 40-69 | 0.4 (0.1 - 0.7) | 0.3 (0.1 - 0.6) |
| 2023 | Pleurisy, Pyothorax | intermediate | 70-79 | 0.5 (0.2 - 0.7) | 2.1 (0.9 - 3.2) |
| 2023 | Pleurisy, Pyothorax | intermediate | 80-89 | 0.6 (0.3 - 0.8) | 9.0 (5.2 - 12.9) |
| 2023 | Pleurisy, Pyothorax | intermediate | 90+ | 0.3 (0.1 - 0.5) | 21.8 (6.7 - 36.9) |
| 2023 | Pleurisy, Pyothorax | low | 40-69 | 0.5 (0.0 - 1.1) | 0.9 (0.0 - 1.9) |
| 2023 | Pleurisy, Pyothorax | low | 70-79 | 0.7 (0.3 - 1.1) | 4.8 (2.0 - 7.6) |
| 2023 | Pleurisy, Pyothorax | low | 80-89 | 0.4 (0.2 - 0.6) | 8.3 (3.9 - 12.6) |
| 2023 | Pleurisy, Pyothorax | low | 90+ | 0.1 (0.0 - 0.2) | 9.1 (0.2 - 18.0) |
| 2023 | Pneumonitis | high | 70-79 | 0.9 (0.1 - 1.7) | 2.6 (0.3 - 4.9) |
| 2023 | Pneumonitis | high | 80-89 | 1.7 (0.8 - 2.5) | 20.7 (10.2 - 31.2) |
| 2023 | Pneumonitis | high | 90+ | 1.8 (0.9 - 2.8) | 133.0 (63.4 - 202.6) |
| 2023 | Pneumonitis | intermediate | 40-69 | 0.5 (0.2 - 0.8) | 0.4 (0.1 - 0.7) |
| 2023 | Pneumonitis | intermediate | 70-79 | 0.6 (0.3 - 0.8) | 2.4 (1.2 - 3.7) |
| 2023 | Pneumonitis | intermediate | 80-89 | 0.9 (0.6 - 1.2) | 14.2 (9.3 - 19.0) |
| 2023 | Pneumonitis | intermediate | 90+ | 0.9 (0.6 - 1.3) | 70.9 (43.7 - 98.2) |
| 2023 | Pneumonitis | low | 40-69 | 1.0 (0.3 - 1.7) | 1.6 (0.4 - 2.9) |
| 2023 | Pneumonitis | low | 70-79 | 1.5 (0.9 - 2.1) | 10.0 (5.9 - 14.1) |
| 2023 | Pneumonitis | low | 80-89 | 1.2 (0.9 - 1.6) | 25.4 (17.8 - 33.0) |
| 2023 | Pneumonitis | low | 90+ | 0.8 (0.5 - 1.0) | 65.9 (41.9 - 89.9) |
| 2023 | Primary or secondary Liver Cancer Unspecified | high | 40-69 | 1.3 (0.0 - 2.6) | 0.4 (0.0 - 0.8) |
| 2023 | Primary or secondary Liver Cancer Unspecified | high | 70-79 | 0.9 (0.1 - 1.7) | 2.6 (0.3 - 4.9) |
| 2023 | Primary or secondary Liver Cancer Unspecified | high | 80-89 | 1.2 (0.5 - 1.9) | 15.2 (6.2 - 24.2) |
| 2023 | Primary or secondary Liver Cancer Unspecified | high | 90+ | 0.4 (-0.1 - 0.8) | 28.5 (-3.7 - 60.8) |
| 2023 | Primary or secondary Liver Cancer Unspecified | intermediate | 40-69 | 2.0 (1.3 - 2.6) | 1.6 (1.1 - 2.1) |
| 2023 | Primary or secondary Liver Cancer Unspecified | intermediate | 70-79 | 1.9 (1.4 - 2.4) | 8.3 (5.9 - 10.6) |
| 2023 | Primary or secondary Liver Cancer Unspecified | intermediate | 80-89 | 1.0 (0.7 - 1.4) | 16.8 (11.5 - 22.0) |
| 2023 | Primary or secondary Liver Cancer Unspecified | intermediate | 90+ | 0.5 (0.2 - 0.7) | 35.5 (16.2 - 54.7) |
| 2023 | Primary or secondary Liver Cancer Unspecified | low | 40-69 | 2.2 (1.1 - 3.2) | 3.8 (1.9 - 5.6) |
| 2023 | Primary or secondary Liver Cancer Unspecified | low | 70-79 | 1.8 (1.1 - 2.4) | 11.7 (7.3 - 16.1) |
| 2023 | Primary or secondary Liver Cancer Unspecified | low | 80-89 | 0.9 (0.6 - 1.2) | 18.3 (11.9 - 24.8) |
| 2023 | Primary or secondary Liver Cancer Unspecified | low | 90+ | 0.1 (0.0 - 0.2) | 11.4 (1.4 - 21.3) |
| 2023 | Pulmonary Embolism | high | 40-69 | 2.6 (0.8 - 4.4) | 0.8 (0.2 - 1.3) |
| 2023 | Pulmonary Embolism | high | 70-79 | 4.5 (2.7 - 6.2) | 12.6 (7.5 - 17.6) |
| 2023 | Pulmonary Embolism | high | 80-89 | 1.2 (0.5 - 1.9) | 15.2 (6.2 - 24.2) |
| 2023 | Pulmonary Embolism | high | 90+ | 1.0 (0.3 - 1.8) | 76.0 (23.4 - 128.7) |
| 2023 | Pulmonary Embolism | intermediate | under 40 | 2.4 (0.7 - 4.0) | 0.3 (0.1 - 0.5) |
| 2023 | Pulmonary Embolism | intermediate | 40-69 | 3.8 (2.9 - 4.7) | 3.1 (2.3 - 3.8) |
| 2023 | Pulmonary Embolism | intermediate | 70-79 | 2.6 (2.0 - 3.2) | 11.2 (8.5 - 13.9) |
| 2023 | Pulmonary Embolism | intermediate | 80-89 | 1.3 (0.9 - 1.6) | 20.6 (14.8 - 26.5) |
| 2023 | Pulmonary Embolism | intermediate | 90+ | 0.8 (0.5 - 1.1) | 62.7 (37.1 - 88.4) |
| 2023 | Pulmonary Embolism | low | under 40 | 0.8 (-0.8 - 2.5) | 0.3 (-0.3 - 1.0) |
| 2023 | Pulmonary Embolism | low | 40-69 | 2.7 (1.6 - 3.9) | 4.7 (2.6 - 6.8) |
| 2023 | Pulmonary Embolism | low | 70-79 | 2.1 (1.4 - 2.8) | 13.9 (9.1 - 18.7) |
| 2023 | Pulmonary Embolism | low | 80-89 | 0.9 (0.5 - 1.2) | 17.7 (11.4 - 24.1) |
| 2023 | Pulmonary Embolism | low | 90+ | 0.9 (0.6 - 1.2) | 79.6 (53.2 - 105.9) |
| 2023 | Self-harm by unspecified means | high | 40-69 | 0.3 (-0.3 - 1.0) | 0.1 (-0.1 - 0.3) |
| 2023 | Self-harm by unspecified means | intermediate | 90+ | 0.0 (-0.0 - 0.1) | 2.7 (-2.6 - 8.1) |
| 2023 | Self-poisoning unspecified | high | under 40 | 4.0 (0.2 - 7.9) | 0.2 (0.0 - 0.4) |
| 2023 | Self-poisoning unspecified | high | 40-69 | 3.0 (1.1 - 4.9) | 0.9 (0.3 - 1.4) |
| 2023 | Self-poisoning unspecified | high | 70-79 | 0.9 (0.1 - 1.7) | 2.6 (0.3 - 4.9) |
| 2023 | Self-poisoning unspecified | high | 80-89 | 0.1 (-0.1 - 0.3) | 1.4 (-1.3 - 4.1) |
| 2023 | Self-poisoning unspecified | intermediate | under 40 | 5.6 (3.2 - 8.1) | 0.7 (0.4 - 1.1) |
| 2023 | Self-poisoning unspecified | intermediate | 40-69 | 2.8 (2.0 - 3.5) | 2.2 (1.6 - 2.8) |
| 2023 | Self-poisoning unspecified | intermediate | 70-79 | 0.6 (0.3 - 0.8) | 2.4 (1.2 - 3.7) |
| 2023 | Self-poisoning unspecified | intermediate | 80-89 | 0.2 (0.0 - 0.3) | 2.6 (0.5 - 4.6) |
| 2023 | Self-poisoning unspecified | low | under 40 | 4.2 (0.6 - 7.8) | 1.6 (0.2 - 3.1) |
| 2023 | Self-poisoning unspecified | low | 40-69 | 2.5 (1.3 - 3.6) | 4.2 (2.3 - 6.2) |
| 2023 | Self-poisoning unspecified | low | 70-79 | 0.5 (0.2 - 0.9) | 3.5 (1.1 - 5.9) |
| 2023 | Self-poisoning unspecified | low | 80-89 | 0.0 (-0.0 - 0.1) | 0.6 (-0.6 - 1.8) |
| 2023 | Self-poisoning unspecified | low | 90+ | 0.1 (-0.0 - 0.1) | 4.5 (-1.8 - 10.8) |
| 2023 | Senility | high | 80-89 | 1.4 (0.7 - 2.2) | 18.0 (8.2 - 27.7) |
| 2023 | Senility | high | 90+ | 9.3 (7.2 - 11.4) | 674.6 (518.2 - 831.0) |
| 2023 | Senility | intermediate | 70-79 | 0.4 (0.1 - 0.6) | 1.6 (0.5 - 2.6) |
| 2023 | Senility | intermediate | 80-89 | 1.7 (1.3 - 2.1) | 27.5 (20.8 - 34.2) |
| 2023 | Senility | intermediate | 90+ | 9.9 (8.8 - 11.0) | 766.4 (677.1 - 855.6) |
| 2023 | Senility | low | 70-79 | 0.4 (0.1 - 0.7) | 2.6 (0.5 - 4.7) |
| 2023 | Senility | low | 80-89 | 2.2 (1.7 - 2.7) | 45.5 (35.4 - 55.7) |
| 2023 | Senility | low | 90+ | 10.5 (9.5 - 11.4) | 900.3 (812.1 - 988.6) |
| 2023 | Sepsis (Non- maternal and neonatal sepsis) | high | under 40 | 6.1 (1.4 - 10.8) | 0.3 (0.1 - 0.5) |
| 2023 | Sepsis (Non- maternal and neonatal sepsis) | high | 40-69 | 1.3 (0.0 - 2.6) | 0.4 (0.0 - 0.8) |
| 2023 | Sepsis (Non- maternal and neonatal sepsis) | high | 70-79 | 2.8 (1.4 - 4.2) | 7.9 (3.9 - 11.8) |
| 2023 | Sepsis (Non- maternal and neonatal sepsis) | high | 80-89 | 5.5 (4.1 - 7.0) | 69.1 (49.9 - 88.2) |
| 2023 | Sepsis (Non- maternal and neonatal sepsis) | high | 90+ | 2.7 (1.6 - 3.9) | 199.5 (114.3 - 284.8) |
| 2023 | Sepsis (Non- maternal and neonatal sepsis) | intermediate | under 40 | 0.6 (-0.2 - 1.4) | 0.1 (-0.0 - 0.2) |
| 2023 | Sepsis (Non- maternal and neonatal sepsis) | intermediate | 40-69 | 2.7 (2.0 - 3.5) | 2.2 (1.6 - 2.8) |
| 2023 | Sepsis (Non- maternal and neonatal sepsis) | intermediate | 70-79 | 4.4 (3.6 - 5.2) | 19.2 (15.6 - 22.7) |
| 2023 | Sepsis (Non- maternal and neonatal sepsis) | intermediate | 80-89 | 5.0 (4.3 - 5.7) | 80.8 (69.2 - 92.3) |
| 2023 | Sepsis (Non- maternal and neonatal sepsis) | intermediate | 90+ | 3.9 (3.2 - 4.6) | 300.0 (244.0 - 356.0) |
| 2023 | Sepsis (Non- maternal and neonatal sepsis) | low | under 40 | 2.5 (-0.3 - 5.3) | 1.0 (-0.1 - 2.1) |
| 2023 | Sepsis (Non- maternal and neonatal sepsis) | low | 40-69 | 3.0 (1.8 - 4.2) | 5.2 (3.0 - 7.3) |
| 2023 | Sepsis (Non- maternal and neonatal sepsis) | low | 70-79 | 5.0 (3.9 - 6.1) | 33.0 (25.6 - 40.4) |
| 2023 | Sepsis (Non- maternal and neonatal sepsis) | low | 80-89 | 5.2 (4.5 - 6.0) | 108.8 (93.1 - 124.5) |
| 2023 | Sepsis (Non- maternal and neonatal sepsis) | low | 90+ | 3.7 (3.1 - 4.3) | 318.3 (265.7 - 370.9) |
| 2023 | Shock, Cardiac Arrest, Coma | high | 40-69 | 1.0 (-0.1 - 2.1) | 0.3 (-0.0 - 0.6) |
| 2023 | Shock, Cardiac Arrest, Coma | high | 70-79 | 0.2 (-0.2 - 0.6) | 0.5 (-0.5 - 1.6) |
| 2023 | Shock, Cardiac Arrest, Coma | high | 80-89 | 0.1 (-0.1 - 0.3) | 1.4 (-1.3 - 4.1) |
| 2023 | Shock, Cardiac Arrest, Coma | high | 90+ | 0.4 (-0.1 - 0.8) | 28.5 (-3.7 - 60.8) |
| 2023 | Shock, Cardiac Arrest, Coma | intermediate | under 40 | 0.3 (-0.3 - 0.9) | 0.0 (-0.0 - 0.1) |
| 2023 | Shock, Cardiac Arrest, Coma | intermediate | 40-69 | 0.3 (0.0 - 0.5) | 0.2 (0.0 - 0.4) |
| 2023 | Shock, Cardiac Arrest, Coma | intermediate | 70-79 | 0.4 (0.1 - 0.6) | 1.6 (0.5 - 2.6) |
| 2023 | Shock, Cardiac Arrest, Coma | intermediate | 80-89 | 0.3 (0.1 - 0.5) | 4.7 (1.9 - 7.5) |
| 2023 | Shock, Cardiac Arrest, Coma | intermediate | 90+ | 0.5 (0.2 - 0.7) | 35.5 (16.2 - 54.7) |
| 2023 | Shock, Cardiac Arrest, Coma | low | under 40 | 0.8 (-0.8 - 2.5) | 0.3 (-0.3 - 1.0) |
| 2023 | Shock, Cardiac Arrest, Coma | low | 40-69 | 0.1 (-0.1 - 0.4) | 0.2 (-0.2 - 0.7) |
| 2023 | Shock, Cardiac Arrest, Coma | low | 70-79 | 0.3 (0.0 - 0.5) | 1.7 (0.0 - 3.4) |
| 2023 | Shock, Cardiac Arrest, Coma | low | 80-89 | 0.5 (0.2 - 0.7) | 9.5 (4.8 - 14.1) |
| 2023 | Shock, Cardiac Arrest, Coma | low | 90+ | 0.2 (0.1 - 0.4) | 20.5 (7.1 - 33.8) |
| 2023 | Undetermined intent Drowning | high | under 40 | 2.0 (-0.8 - 4.8) | 0.1 (-0.0 - 0.2) |
| 2023 | Undetermined intent Drowning | high | 40-69 | 1.3 (0.0 - 2.6) | 0.4 (0.0 - 0.8) |
| 2023 | Undetermined intent Drowning | high | 90+ | 0.1 (-0.1 - 0.4) | 9.5 (-9.1 - 28.1) |
| 2023 | Undetermined intent Drowning | intermediate | under 40 | 1.5 (0.2 - 2.8) | 0.2 (0.0 - 0.4) |
| 2023 | Undetermined intent Drowning | intermediate | 40-69 | 0.3 (0.0 - 0.5) | 0.2 (0.0 - 0.4) |
| 2023 | Undetermined intent Drowning | intermediate | 80-89 | 0.1 (-0.0 - 0.1) | 0.9 (-0.3 - 2.1) |
| 2023 | Undetermined intent Drowning | low | under 40 | 2.5 (-0.3 - 5.3) | 1.0 (-0.1 - 2.1) |
| 2023 | Undetermined intent Drowning | low | 40-69 | 0.3 (-0.1 - 0.7) | 0.5 (-0.2 - 1.1) |
| 2023 | Undetermined intent Drowning | low | 80-89 | 0.0 (-0.0 - 0.1) | 0.6 (-0.6 - 1.8) |
| 2023 | Undetermined intent shooting by unspecified firearm | low | under 40 | 0.8 (-0.8 - 2.5) | 0.3 (-0.3 - 1.0) |
| 2023 | Undetermined intent Poisoning by antiepileptic and psychotropic drugs | high | 40-69 | 1.6 (0.2 - 3.1) | 0.5 (0.1 - 0.9) |
| 2023 | Undetermined intent Poisoning by antiepileptic and psychotropic drugs | high | 70-79 | 0.2 (-0.2 - 0.6) | 0.5 (-0.5 - 1.6) |
| 2023 | Undetermined intent Poisoning by antiepileptic and psychotropic drugs | high | 80-89 | 0.1 (-0.1 - 0.3) | 1.4 (-1.3 - 4.1) |
| 2023 | Undetermined intent Poisoning by antiepileptic and psychotropic drugs | intermediate | under 40 | 1.8 (0.4 - 3.2) | 0.2 (0.0 - 0.4) |
| 2023 | Undetermined intent Poisoning by antiepileptic and psychotropic drugs | intermediate | 40-69 | 1.2 (0.7 - 1.7) | 0.9 (0.5 - 1.4) |
| 2023 | Undetermined intent Poisoning by antiepileptic and psychotropic drugs | intermediate | 70-79 | 0.3 (0.1 - 0.5) | 1.2 (0.3 - 2.1) |
| 2023 | Undetermined intent Poisoning by antiepileptic and psychotropic drugs | low | under 40 | 0.8 (-0.8 - 2.5) | 0.3 (-0.3 - 1.0) |
| 2023 | Undetermined intent Poisoning by antiepileptic and psychotropic drugs | low | 40-69 | 1.6 (0.7 - 2.6) | 2.8 (1.2 - 4.4) |
| 2023 | Undetermined intent Poisoning by autonomic nervous system drugs | intermediate | 40-69 | 0.1 (-0.1 - 0.2) | 0.0 (-0.0 - 0.1) |
| 2023 | Undetermined intent Poisoning by autonomic nervous system drugs | intermediate | 70-79 | 0.0 (-0.0 - 0.1) | 0.2 (-0.2 - 0.5) |
| 2023 | Undetermined intent Poisoning by autonomic nervous system drugs | intermediate | 80-89 | 0.0 (-0.0 - 0.1) | 0.4 (-0.4 - 1.3) |
| 2023 | Undetermined intent Poisoning by multiple or unspecified drug | high | under 40 | 17.2 (9.7 - 24.6) | 0.8 (0.4 - 1.2) |
| 2023 | Undetermined intent Poisoning by multiple or unspecified drug | high | 40-69 | 4.6 (2.2 - 6.9) | 1.3 (0.6 - 2.0) |
| 2023 | Undetermined intent Poisoning by multiple or unspecified drug | high | 80-89 | 0.1 (-0.1 - 0.3) | 1.4 (-1.3 - 4.1) |
| 2023 | Undetermined intent Poisoning by multiple or unspecified drug | intermediate | under 40 | 32.0 (27.1 - 37.0) | 4.2 (3.4 - 5.0) |
| 2023 | Undetermined intent Poisoning by multiple or unspecified drug | intermediate | 40-69 | 5.4 (4.4 - 6.5) | 4.3 (3.5 - 5.2) |
| 2023 | Undetermined intent Poisoning by multiple or unspecified drug | intermediate | 70-79 | 0.3 (0.1 - 0.5) | 1.2 (0.3 - 2.1) |
| 2023 | Undetermined intent Poisoning by multiple or unspecified drug | intermediate | 80-89 | 0.1 (0.0 - 0.2) | 2.1 (0.3 - 4.0) |
| 2023 | Undetermined intent Poisoning by multiple or unspecified drug | low | under 40 | 43.7 (34.8 - 52.6) | 16.9 (12.3 - 21.5) |
| 2023 | Undetermined intent Poisoning by multiple or unspecified drug | low | 40-69 | 5.6 (3.9 - 7.3) | 9.6 (6.7 - 12.6) |
| 2023 | Undetermined intent Poisoning by multiple or unspecified drug | low | 70-79 | 0.4 (0.1 - 0.7) | 2.6 (0.5 - 4.7) |
| 2023 | Undetermined intent Poisoning by multiple or unspecified drug | low | 80-89 | 0.1 (-0.0 - 0.2) | 1.8 (-0.2 - 3.8) |
| 2023 | Undetermined intent Poisoning by multiple or unspecified drug | low | 90+ | 0.0 (-0.0 - 0.1) | 2.3 (-2.2 - 6.7) |
| 2023 | Undetermined intent Poisoning by narcotics and psychodysleptics drugs | high | under 40 | 4.0 (0.2 - 7.9) | 0.2 (0.0 - 0.4) |
| 2023 | Undetermined intent Poisoning by narcotics and psychodysleptics drugs | high | 40-69 | 0.3 (-0.3 - 1.0) | 0.1 (-0.1 - 0.3) |
| 2023 | Undetermined intent Poisoning by narcotics and psychodysleptics drugs | high | 70-79 | 0.2 (-0.2 - 0.6) | 0.5 (-0.5 - 1.6) |
| 2023 | Undetermined intent Poisoning by narcotics and psychodysleptics drugs | intermediate | under 40 | 2.1 (0.6 - 3.6) | 0.3 (0.1 - 0.5) |
| 2023 | Undetermined intent Poisoning by narcotics and psychodysleptics drugs | intermediate | 40-69 | 0.8 (0.4 - 1.3) | 0.7 (0.3 - 1.0) |
| 2023 | Undetermined intent Poisoning by narcotics and psychodysleptics drugs | intermediate | 80-89 | 0.0 (-0.0 - 0.1) | 0.4 (-0.4 - 1.3) |
| 2023 | Undetermined intent Poisoning by narcotics and psychodysleptics drugs | low | under 40 | 3.4 (0.1 - 6.6) | 1.3 (0.0 - 2.6) |
| 2023 | Undetermined intent Poisoning by narcotics and psychodysleptics drugs | low | 40-69 | 0.8 (0.2 - 1.5) | 1.4 (0.3 - 2.5) |
| 2023 | Undetermined intent Poisoning by narcotics and psychodysleptics drugs | low | 70-79 | 0.1 (-0.1 - 0.2) | 0.4 (-0.4 - 1.3) |
| 2023 | Undetermined intent Poisoning by no opioid analgesics | intermediate | 40-69 | 0.2 (0.0 - 0.4) | 0.2 (0.0 - 0.4) |
| 2023 | Undetermined intent Poisoning by no opioid analgesics | low | 40-69 | 0.1 (-0.1 - 0.4) | 0.2 (-0.2 - 0.7) |
| 2023 | Undetermined intent Poisoning by no opioid analgesics | low | 70-79 | 0.1 (-0.1 - 0.2) | 0.4 (-0.4 - 1.3) |
| 2023 | Undetermined intent Poisoning by other gases and vapors | intermediate | under 40 | 0.3 (-0.3 - 0.9) | 0.0 (-0.0 - 0.1) |
| 2023 | Undetermined intent Poisoning by other gases and vapors | intermediate | 40-69 | 0.1 (-0.1 - 0.2) | 0.0 (-0.0 - 0.1) |
| 2023 | Undetermined intent Poisoning by other gases and vapors | intermediate | 80-89 | 0.0 (-0.0 - 0.1) | 0.4 (-0.4 - 1.3) |
| 2023 | Undetermined intent Poisoning by solvents and halogenated hydrocarbons | intermediate | 40-69 | 0.1 (-0.1 - 0.2) | 0.0 (-0.0 - 0.1) |
| 2023 | Undetermined intent Poisoning by unspecified chemicals and noxious substances | intermediate | under 40 | 0.3 (-0.3 - 0.9) | 0.0 (-0.0 - 0.1) |
| 2023 | Undetermined intent Poisoning by unspecified chemicals and noxious substances | intermediate | 70-79 | 0.0 (-0.0 - 0.1) | 0.2 (-0.2 - 0.5) |
| 2023 | Undetermined intent Poisoning by unspecified drugs and biological drugs | high | under 40 | 4.0 (0.2 - 7.9) | 0.2 (0.0 - 0.4) |
| 2023 | Undetermined intent Poisoning by unspecified drugs and biological drugs | high | 40-69 | 0.3 (-0.3 - 1.0) | 0.1 (-0.1 - 0.3) |
| 2023 | Undetermined intent Poisoning by unspecified drugs and biological drugs | intermediate | under 40 | 4.2 (2.0 - 6.3) | 0.5 (0.3 - 0.8) |
| 2023 | Undetermined intent Poisoning by unspecified drugs and biological drugs | intermediate | 40-69 | 2.0 (1.3 - 2.6) | 1.6 (1.1 - 2.1) |
| 2023 | Undetermined intent Poisoning by unspecified drugs and biological drugs | intermediate | 70-79 | 0.1 (-0.0 - 0.3) | 0.5 (-0.1 - 1.1) |
| 2023 | Undetermined intent Poisoning by unspecified drugs and biological drugs | intermediate | 80-89 | 0.0 (-0.0 - 0.1) | 0.4 (-0.4 - 1.3) |
| 2023 | Undetermined intent Poisoning by unspecified drugs and biological drugs | low | under 40 | 5.0 (1.1 - 9.0) | 2.0 (0.4 - 3.5) |
| 2023 | Undetermined intent Poisoning by unspecified drugs and biological drugs | low | 40-69 | 1.2 (0.4 - 2.0) | 2.1 (0.7 - 3.5) |
| 2023 | Undetermined intent Poisoning by unspecified drugs and biological drugs | low | 70-79 | 0.1 (-0.1 - 0.3) | 0.9 (-0.3 - 2.1) |
| 2023 | Undetermined intent Poisoning by unspecified drugs and biological drugs | low | 80-89 | 0.0 (-0.0 - 0.1) | 0.6 (-0.6 - 1.8) |
| 2023 | Undetermined intent Strangulation | high | under 40 | 2.0 (-0.8 - 4.8) | 0.1 (-0.0 - 0.2) |
| 2023 | Undetermined intent Strangulation | intermediate | under 40 | 0.6 (-0.2 - 1.4) | 0.1 (-0.0 - 0.2) |
| 2023 | Undetermined intent Strangulation | intermediate | 40-69 | 0.2 (-0.0 - 0.4) | 0.1 (-0.0 - 0.3) |
| 2023 | Undetermined intent of Crashing | high | under 40 | 2.0 (-0.8 - 4.8) | 0.1 (-0.0 - 0.2) |
| 2023 | Undetermined intent of Crashing | high | 70-79 | 0.2 (-0.2 - 0.6) | 0.5 (-0.5 - 1.6) |
| 2023 | Undetermined intent of Crashing | intermediate | under 40 | 0.6 (-0.2 - 1.4) | 0.1 (-0.0 - 0.2) |
| 2023 | Undetermined intent of Crashing | intermediate | 40-69 | 0.5 (0.1 - 0.8) | 0.4 (0.1 - 0.6) |
| 2023 | Undetermined intent of Crashing | intermediate | 80-89 | 0.1 (-0.0 - 0.1) | 0.9 (-0.3 - 2.1) |
| 2023 | Undetermined intent of Explosion | intermediate | 40-69 | 0.1 (-0.1 - 0.2) | 0.0 (-0.0 - 0.1) |
| 2023 | Undetermined intent of Moving Objects | intermediate | under 40 | 0.3 (-0.3 - 0.9) | 0.0 (-0.0 - 0.1) |
| 2023 | Undetermined intent of Moving Objects | intermediate | 40-69 | 0.1 (-0.0 - 0.3) | 0.1 (-0.0 - 0.2) |
| 2023 | Undetermined intent of Moving Objects | intermediate | 80-89 | 0.0 (-0.0 - 0.1) | 0.4 (-0.4 - 1.3) |
| 2023 | Undetermined intent of Moving Objects | low | 40-69 | 0.1 (-0.1 - 0.4) | 0.2 (-0.2 - 0.7) |
| 2023 | Undetermined intent of Moving Objects | low | 80-89 | 0.0 (-0.0 - 0.1) | 0.6 (-0.6 - 1.8) |
| 2023 | Undetermined intent of fall | high | under 40 | 1.0 (-1.0 - 3.0) | 0.0 (-0.0 - 0.1) |
| 2023 | Undetermined intent of fall | high | 40-69 | 1.0 (-0.1 - 2.1) | 0.3 (-0.0 - 0.6) |
| 2023 | Undetermined intent of fall | intermediate | under 40 | 0.9 (-0.1 - 1.9) | 0.1 (-0.0 - 0.2) |
| 2023 | Undetermined intent of fall | low | under 40 | 1.7 (-0.6 - 4.0) | 0.7 (-0.3 - 1.6) |
| 2023 | Undetermined intent of fall | low | 80-89 | 0.1 (-0.0 - 0.1) | 1.2 (-0.5 - 2.8) |
| 2023 | Undetermined intent of fire and flames | high | 90+ | 0.1 (-0.1 - 0.4) | 9.5 (-9.1 - 28.1) |
| 2023 | Undetermined intent of fire and flames | intermediate | 40-69 | 0.4 (0.1 - 0.7) | 0.3 (0.1 - 0.6) |
| 2023 | Undetermined intent of fire and flames | intermediate | 70-79 | 0.0 (-0.0 - 0.1) | 0.2 (-0.2 - 0.5) |
| 2023 | Undetermined intent of fire and flames | low | 40-69 | 0.3 (-0.1 - 0.7) | 0.5 (-0.2 - 1.1) |
| 2023 | Undetermined intent of fire and flames | low | 70-79 | 0.1 (-0.1 - 0.2) | 0.4 (-0.4 - 1.3) |
| 2023 | Unspecified Intestine Diseases | high | 40-69 | 0.7 (-0.3 - 1.6) | 0.2 (-0.1 - 0.5) |
| 2023 | Unspecified Intestine Diseases | high | 70-79 | 1.1 (0.2 - 2.0) | 3.1 (0.6 - 5.7) |
| 2023 | Unspecified Intestine Diseases | high | 80-89 | 0.3 (-0.0 - 0.7) | 4.1 (-0.5 - 8.8) |
| 2023 | Unspecified Intestine Diseases | high | 90+ | 0.4 (-0.1 - 0.8) | 28.5 (-3.7 - 60.8) |
| 2023 | Unspecified Intestine Diseases | intermediate | under 40 | 0.6 (-0.2 - 1.4) | 0.1 (-0.0 - 0.2) |
| 2023 | Unspecified Intestine Diseases | intermediate | 40-69 | 0.5 (0.1 - 0.8) | 0.4 (0.1 - 0.6) |
| 2023 | Unspecified Intestine Diseases | intermediate | 70-79 | 0.4 (0.2 - 0.7) | 1.9 (0.8 - 3.0) |
| 2023 | Unspecified Intestine Diseases | intermediate | 80-89 | 0.6 (0.4 - 0.9) | 9.9 (5.8 - 13.9) |
| 2023 | Unspecified Intestine Diseases | intermediate | 90+ | 0.3 (0.1 - 0.5) | 24.5 (8.5 - 40.6) |
| 2023 | Unspecified Intestine Diseases | low | 40-69 | 0.4 (-0.1 - 0.9) | 0.7 (-0.1 - 1.5) |
| 2023 | Unspecified Intestine Diseases | low | 70-79 | 0.6 (0.2 - 1.0) | 3.9 (1.4 - 6.5) |
| 2023 | Unspecified Intestine Diseases | low | 80-89 | 0.3 (0.1 - 0.5) | 6.5 (2.7 - 10.3) |
| 2023 | Unspecified Intestine Diseases | low | 90+ | 0.2 (0.1 - 0.4) | 20.5 (7.1 - 33.8) |
| 2023 | Unspecified Bacterial Diseases | high | 70-79 | 0.2 (-0.2 - 0.6) | 0.5 (-0.5 - 1.6) |
| 2023 | Unspecified Bacterial Diseases | high | 80-89 | 0.4 (0.0 - 0.9) | 5.5 (0.1 - 10.9) |
| 2023 | Unspecified Bacterial Diseases | high | 90+ | 1.4 (0.6 - 2.3) | 104.5 (42.8 - 166.2) |
| 2023 | Unspecified Bacterial Diseases | intermediate | 40-69 | 0.2 (-0.0 - 0.4) | 0.1 (-0.0 - 0.3) |
| 2023 | Unspecified Bacterial Diseases | intermediate | 70-79 | 0.2 (0.0 - 0.4) | 1.0 (0.2 - 1.9) |
| 2023 | Unspecified Bacterial Diseases | intermediate | 80-89 | 0.7 (0.4 - 1.0) | 11.6 (7.2 - 16.0) |
| 2023 | Unspecified Bacterial Diseases | intermediate | 90+ | 0.5 (0.3 - 0.8) | 40.9 (20.2 - 61.6) |
| 2023 | Unspecified Bacterial Diseases | low | 40-69 | 0.4 (-0.1 - 0.9) | 0.7 (-0.1 - 1.5) |
| 2023 | Unspecified Bacterial Diseases | low | 70-79 | 0.6 (0.2 - 1.0) | 3.9 (1.4 - 6.5) |
| 2023 | Unspecified Bacterial Diseases | low | 80-89 | 0.7 (0.5 - 1.0) | 15.4 (9.5 - 21.3) |
| 2023 | Unspecified Bacterial Diseases | low | 90+ | 0.7 (0.4 - 1.0) | 59.1 (36.4 - 81.8) |
| 2023 | Unspecified Blood Diseases | intermediate | 70-79 | 0.0 (-0.0 - 0.1) | 0.2 (-0.2 - 0.5) |
| 2023 | Unspecified Blood Diseases | low | 90+ | 0.0 (-0.0 - 0.1) | 2.3 (-2.2 - 6.7) |
| 2023 | Unspecified Brain Diseases | high | 70-79 | 0.7 (0.0 - 1.5) | 2.1 (0.0 - 4.2) |
| 2023 | Unspecified Brain Diseases | high | 80-89 | 0.6 (0.1 - 1.0) | 6.9 (0.9 - 13.0) |
| 2023 | Unspecified Brain Diseases | high | 90+ | 0.3 (-0.1 - 0.6) | 19.0 (-7.3 - 45.3) |
| 2023 | Unspecified Brain Diseases | intermediate | 70-79 | 0.1 (-0.0 - 0.3) | 0.5 (-0.1 - 1.1) |
| 2023 | Unspecified Brain Diseases | intermediate | 80-89 | 0.1 (0.0 - 0.2) | 2.1 (0.3 - 4.0) |
| 2023 | Unspecified Brain Diseases | intermediate | 90+ | 0.1 (0.0 - 0.3) | 10.9 (0.2 - 21.6) |
| 2023 | Unspecified Brain Diseases | low | 70-79 | 0.1 (-0.1 - 0.2) | 0.4 (-0.4 - 1.3) |
| 2023 | Unspecified Brain Diseases | low | 80-89 | 0.1 (-0.0 - 0.1) | 1.2 (-0.5 - 2.8) |
| 2023 | Unspecified Brain Diseases | low | 90+ | 0.1 (0.0 - 0.2) | 9.1 (0.2 - 18.0) |
| 2023 | Unspecified Bronchitis and Bronchiectasis | high | 70-79 | 0.2 (-0.2 - 0.6) | 0.5 (-0.5 - 1.6) |
| 2023 | Unspecified Bronchitis and Bronchiectasis | high | 80-89 | 0.2 (-0.1 - 0.5) | 2.8 (-1.1 - 6.6) |
| 2023 | Unspecified Bronchitis and Bronchiectasis | high | 90+ | 0.1 (-0.1 - 0.4) | 9.5 (-9.1 - 28.1) |
| 2023 | Unspecified Bronchitis and Bronchiectasis | intermediate | 40-69 | 0.1 (-0.1 - 0.2) | 0.0 (-0.0 - 0.1) |
| 2023 | Unspecified Bronchitis and Bronchiectasis | intermediate | 70-79 | 0.2 (0.0 - 0.3) | 0.7 (0.0 - 1.4) |
| 2023 | Unspecified Bronchitis and Bronchiectasis | intermediate | 80-89 | 0.1 (-0.0 - 0.2) | 1.3 (-0.2 - 2.7) |
| 2023 | Unspecified Bronchitis and Bronchiectasis | low | 70-79 | 0.1 (-0.1 - 0.2) | 0.4 (-0.4 - 1.3) |
| 2023 | Unspecified Bronchitis and Bronchiectasis | low | 80-89 | 0.1 (-0.0 - 0.2) | 1.8 (-0.2 - 3.8) |
| 2023 | Unspecified Bronchitis and Bronchiectasis | low | 90+ | 0.1 (-0.0 - 0.1) | 4.5 (-1.8 - 10.8) |
| 2023 | Unspecified CNS Diseases | high | 70-79 | 0.2 (-0.2 - 0.6) | 0.5 (-0.5 - 1.6) |
| 2023 | Unspecified CNS Diseases | high | 80-89 | 0.3 (-0.0 - 0.7) | 4.1 (-0.5 - 8.8) |
| 2023 | Unspecified CNS Diseases | high | 90+ | 0.1 (-0.1 - 0.4) | 9.5 (-9.1 - 28.1) |
| 2023 | Unspecified CNS Diseases | intermediate | 40-69 | 0.1 (-0.0 - 0.3) | 0.1 (-0.0 - 0.2) |
| 2023 | Unspecified CNS Diseases | intermediate | 70-79 | 0.2 (0.0 - 0.3) | 0.7 (0.0 - 1.4) |
| 2023 | Unspecified CNS Diseases | intermediate | 80-89 | 0.1 (0.0 - 0.2) | 1.7 (0.0 - 3.4) |
| 2023 | Unspecified CNS Diseases | intermediate | 90+ | 0.2 (0.0 - 0.3) | 13.6 (1.7 - 25.6) |
| 2023 | Unspecified CNS Diseases | low | 70-79 | 0.1 (-0.1 - 0.3) | 0.9 (-0.3 - 2.1) |
| 2023 | Unspecified CNS Diseases | low | 80-89 | 0.1 (-0.0 - 0.2) | 1.8 (-0.2 - 3.8) |
| 2023 | Unspecified CNS Diseases | low | 90+ | 0.1 (-0.0 - 0.1) | 4.5 (-1.8 - 10.8) |
| 2023 | Unspecified CNS Infection | intermediate | under 40 | 0.6 (-0.2 - 1.4) | 0.1 (-0.0 - 0.2) |
| 2023 | Unspecified CNS Infection | intermediate | 70-79 | 0.1 (-0.0 - 0.2) | 0.3 (-0.1 - 0.8) |
| 2023 | Unspecified CNS Infection | low | 70-79 | 0.1 (-0.1 - 0.2) | 0.4 (-0.4 - 1.3) |
| 2023 | Unspecified CNS Infection | low | 80-89 | 0.0 (-0.0 - 0.1) | 0.6 (-0.6 - 1.8) |
| 2023 | Unspecified Cardiomyopathy | high | under 40 | 1.0 (-1.0 - 3.0) | 0.0 (-0.0 - 0.1) |
| 2023 | Unspecified Cardiomyopathy | high | 40-69 | 2.0 (0.4 - 3.5) | 0.6 (0.1 - 1.0) |
| 2023 | Unspecified Cardiomyopathy | high | 70-79 | 1.7 (0.6 - 2.8) | 4.7 (1.6 - 7.8) |
| 2023 | Unspecified Cardiomyopathy | high | 80-89 | 1.0 (0.3 - 1.6) | 12.4 (4.3 - 20.6) |
| 2023 | Unspecified Cardiomyopathy | high | 90+ | 0.3 (-0.1 - 0.6) | 19.0 (-7.3 - 45.3) |
| 2023 | Unspecified Cardiomyopathy | intermediate | under 40 | 0.9 (-0.1 - 1.9) | 0.1 (-0.0 - 0.2) |
| 2023 | Unspecified Cardiomyopathy | intermediate | 40-69 | 2.1 (1.4 - 2.8) | 1.7 (1.1 - 2.2) |
| 2023 | Unspecified Cardiomyopathy | intermediate | 70-79 | 1.5 (1.0 - 2.0) | 6.6 (4.5 - 8.6) |
| 2023 | Unspecified Cardiomyopathy | intermediate | 80-89 | 0.6 (0.4 - 0.9) | 9.9 (5.8 - 13.9) |
| 2023 | Unspecified Cardiomyopathy | intermediate | 90+ | 0.2 (0.1 - 0.4) | 19.1 (4.9 - 33.2) |
| 2023 | Unspecified Cardiomyopathy | low | under 40 | 3.4 (0.1 - 6.6) | 1.3 (0.0 - 2.6) |
| 2023 | Unspecified Cardiomyopathy | low | 40-69 | 2.2 (1.1 - 3.2) | 3.8 (1.9 - 5.6) |
| 2023 | Unspecified Cardiomyopathy | low | 70-79 | 1.5 (0.9 - 2.1) | 10.0 (5.9 - 14.1) |
| 2023 | Unspecified Cardiomyopathy | low | 80-89 | 0.8 (0.5 - 1.1) | 16.6 (10.4 - 22.7) |
| 2023 | Unspecified Cardiomyopathy | low | 90+ | 0.2 (0.1 - 0.4) | 18.2 (5.6 - 30.8) |
| 2023 | Unspecified Chromosomal Diseases | high | under 40 | 1.0 (-1.0 - 3.0) | 0.0 (-0.0 - 0.1) |
| 2023 | Unspecified Chromosomal Diseases | low | 40-69 | 0.1 (-0.1 - 0.4) | 0.2 (-0.2 - 0.7) |
| 2023 | Unspecified Congenital Diseases | intermediate | under 40 | 0.3 (-0.3 - 0.9) | 0.0 (-0.0 - 0.1) |
| 2023 | Unspecified Digestive Diseases | intermediate | 40-69 | 0.1 (-0.1 - 0.2) | 0.0 (-0.0 - 0.1) |
| 2023 | Unspecified Digestive Diseases | intermediate | 80-89 | 0.0 (-0.0 - 0.1) | 0.4 (-0.4 - 1.3) |
| 2023 | Unspecified Digestive Diseases | intermediate | 90+ | 0.0 (-0.0 - 0.1) | 2.7 (-2.6 - 8.1) |
| 2023 | Unspecified Digestive Diseases | low | 70-79 | 0.1 (-0.1 - 0.2) | 0.4 (-0.4 - 1.3) |
| 2023 | Unspecified Digestive Diseases | low | 80-89 | 0.0 (-0.0 - 0.1) | 0.6 (-0.6 - 1.8) |
| 2023 | Unspecified Digestive Diseases | low | 90+ | 0.0 (-0.0 - 0.1) | 2.3 (-2.2 - 6.7) |
| 2023 | Unspecified Eating Disorders | intermediate | 40-69 | 0.1 (-0.1 - 0.2) | 0.0 (-0.0 - 0.1) |
| 2023 | Unspecified Female Genital Cancer | high | 40-69 | 1.0 (-0.1 - 2.1) | 0.3 (-0.0 - 0.6) |
| 2023 | Unspecified Female Genital Cancer | high | 70-79 | 0.6 (-0.1 - 1.2) | 1.6 (-0.2 - 3.4) |
| 2023 | Unspecified Female Genital Cancer | high | 80-89 | 0.7 (0.1 - 1.2) | 8.3 (1.7 - 14.9) |
| 2023 | Unspecified Female Genital Cancer | high | 90+ | 0.1 (-0.1 - 0.4) | 9.5 (-9.1 - 28.1) |
| 2023 | Unspecified Female Genital Cancer | intermediate | 40-69 | 0.6 (0.3 - 1.0) | 0.5 (0.2 - 0.8) |
| 2023 | Unspecified Female Genital Cancer | intermediate | 70-79 | 0.7 (0.4 - 1.0) | 2.9 (1.5 - 4.3) |
| 2023 | Unspecified Female Genital Cancer | intermediate | 80-89 | 0.3 (0.1 - 0.5) | 4.7 (1.9 - 7.5) |
| 2023 | Unspecified Female Genital Cancer | intermediate | 90+ | 0.1 (0.0 - 0.3) | 10.9 (0.2 - 21.6) |
| 2023 | Unspecified Female Genital Cancer | low | 40-69 | 0.4 (-0.1 - 0.9) | 0.7 (-0.1 - 1.5) |
| 2023 | Unspecified Female Genital Cancer | low | 70-79 | 0.3 (0.0 - 0.5) | 1.7 (0.0 - 3.4) |
| 2023 | Unspecified Female Genital Cancer | low | 80-89 | 0.3 (0.1 - 0.5) | 6.5 (2.7 - 10.3) |
| 2023 | Unspecified Female Genital Cancer | low | 90+ | 0.3 (0.1 - 0.5) | 25.0 (10.2 - 39.8) |
| 2023 | Unspecified GI Cancer | high | 40-69 | 2.3 (0.6 - 4.0) | 0.7 (0.2 - 1.2) |
| 2023 | Unspecified GI Cancer | high | 70-79 | 2.6 (1.3 - 4.0) | 7.3 (3.5 - 11.2) |
| 2023 | Unspecified GI Cancer | high | 80-89 | 1.0 (0.3 - 1.6) | 12.4 (4.3 - 20.6) |
| 2023 | Unspecified GI Cancer | high | 90+ | 0.4 (-0.1 - 0.8) | 28.5 (-3.7 - 60.8) |
| 2023 | Unspecified GI Cancer | intermediate | 40-69 | 1.5 (0.9 - 2.0) | 1.2 (0.7 - 1.6) |
| 2023 | Unspecified GI Cancer | intermediate | 70-79 | 1.7 (1.2 - 2.2) | 7.3 (5.1 - 9.4) |
| 2023 | Unspecified GI Cancer | intermediate | 80-89 | 1.2 (0.9 - 1.6) | 19.8 (14.1 - 25.5) |
| 2023 | Unspecified GI Cancer | intermediate | 90+ | 0.9 (0.6 - 1.3) | 73.6 (45.9 - 101.4) |
| 2023 | Unspecified GI Cancer | low | 40-69 | 1.0 (0.3 - 1.7) | 1.6 (0.4 - 2.9) |
| 2023 | Unspecified GI Cancer | low | 70-79 | 2.0 (1.3 - 2.7) | 13.5 (8.7 - 18.2) |
| 2023 | Unspecified GI Cancer | low | 80-89 | 1.0 (0.6 - 1.3) | 20.1 (13.3 - 26.9) |
| 2023 | Unspecified GI Cancer | low | 90+ | 0.8 (0.5 - 1.0) | 65.9 (41.9 - 89.9) |
| 2023 | Unspecified Gynecologic Diseases | intermediate | 80-89 | 0.0 (-0.0 - 0.1) | 0.4 (-0.4 - 1.3) |
| 2023 | Unspecified Gynecologic Diseases | low | 80-89 | 0.0 (-0.0 - 0.1) | 0.6 (-0.6 - 1.8) |
| 2023 | Unspecified Heart Diseases | high | 40-69 | 3.9 (1.8 - 6.1) | 1.1 (0.5 - 1.8) |
| 2023 | Unspecified Heart Diseases | high | 70-79 | 1.1 (0.2 - 2.0) | 3.1 (0.6 - 5.7) |
| 2023 | Unspecified Heart Diseases | high | 80-89 | 0.6 (0.1 - 1.0) | 6.9 (0.9 - 13.0) |
| 2023 | Unspecified Heart Diseases | high | 90+ | 0.4 (-0.1 - 0.8) | 28.5 (-3.7 - 60.8) |
| 2023 | Unspecified Heart Diseases | intermediate | under 40 | 0.3 (-0.3 - 0.9) | 0.0 (-0.0 - 0.1) |
| 2023 | Unspecified Heart Diseases | intermediate | 40-69 | 5.0 (4.0 - 6.0) | 4.0 (3.2 - 4.9) |
| 2023 | Unspecified Heart Diseases | intermediate | 70-79 | 1.8 (1.3 - 2.3) | 7.9 (5.6 - 10.2) |
| 2023 | Unspecified Heart Diseases | intermediate | 80-89 | 0.6 (0.3 - 0.8) | 9.0 (5.2 - 12.9) |
| 2023 | Unspecified Heart Diseases | intermediate | 90+ | 0.6 (0.3 - 0.9) | 49.1 (26.4 - 71.8) |
| 2023 | Unspecified Heart Diseases | low | under 40 | 1.7 (-0.6 - 4.0) | 0.7 (-0.3 - 1.6) |
| 2023 | Unspecified Heart Diseases | low | 40-69 | 5.1 (3.5 - 6.7) | 8.7 (5.9 - 11.5) |
| 2023 | Unspecified Heart Diseases | low | 70-79 | 1.2 (0.7 - 1.8) | 8.2 (4.5 - 12.0) |
| 2023 | Unspecified Heart Diseases | low | 80-89 | 0.7 (0.4 - 0.9) | 13.6 (8.0 - 19.2) |
| 2023 | Unspecified Heart Diseases | low | 90+ | 0.6 (0.4 - 0.9) | 52.3 (30.9 - 73.7) |
| 2023 | Unspecified Infectious Diseases | high | under 40 | 2.0 (-0.8 - 4.8) | 0.1 (-0.0 - 0.2) |
| 2023 | Unspecified Infectious Diseases | high | 40-69 | 0.7 (-0.3 - 1.6) | 0.2 (-0.1 - 0.5) |
| 2023 | Unspecified Infectious Diseases | high | 70-79 | 2.4 (1.1 - 3.7) | 6.8 (3.1 - 10.5) |
| 2023 | Unspecified Infectious Diseases | high | 80-89 | 3.8 (2.5 - 5.0) | 47.0 (31.2 - 62.7) |
| 2023 | Unspecified Infectious Diseases | high | 90+ | 3.9 (2.5 - 5.3) | 285.0 (183.2 - 386.9) |
| 2023 | Unspecified Infectious Diseases | intermediate | 40-69 | 0.6 (0.2 - 0.9) | 0.5 (0.2 - 0.7) |
| 2023 | Unspecified Infectious Diseases | intermediate | 70-79 | 4.0 (3.2 - 4.7) | 17.3 (13.9 - 20.6) |
| 2023 | Unspecified Infectious Diseases | intermediate | 80-89 | 4.5 (3.8 - 5.1) | 72.6 (61.7 - 83.6) |
| 2023 | Unspecified Infectious Diseases | intermediate | 90+ | 4.0 (3.3 - 4.8) | 313.6 (256.4 - 370.9) |
| 2023 | Unspecified Infectious Diseases | low | 40-69 | 1.6 (0.7 - 2.6) | 2.8 (1.2 - 4.4) |
| 2023 | Unspecified Infectious Diseases | low | 70-79 | 3.6 (2.7 - 4.5) | 23.9 (17.6 - 30.2) |
| 2023 | Unspecified Infectious Diseases | low | 80-89 | 4.5 (3.8 - 5.2) | 93.4 (78.9 - 108.0) |
| 2023 | Unspecified Infectious Diseases | low | 90+ | 4.4 (3.7 - 5.0) | 377.4 (320.1 - 434.7) |
| 2023 | Unspecified Male Genital Cancer | intermediate | 80-89 | 0.0 (-0.0 - 0.1) | 0.4 (-0.4 - 1.3) |
| 2023 | Unspecified Male Genital Cancer | low | 40-69 | 0.1 (-0.1 - 0.4) | 0.2 (-0.2 - 0.7) |
| 2023 | Unspecified Meningitis | intermediate | 40-69 | 0.1 (-0.1 - 0.2) | 0.0 (-0.0 - 0.1) |
| 2023 | Unspecified Meningitis | intermediate | 70-79 | 0.1 (-0.0 - 0.2) | 0.3 (-0.1 - 0.8) |
| 2023 | Unspecified Meningitis | intermediate | 80-89 | 0.0 (-0.0 - 0.1) | 0.4 (-0.4 - 1.3) |
| 2023 | Unspecified Meningitis | intermediate | 90+ | 0.0 (-0.0 - 0.1) | 2.7 (-2.6 - 8.1) |
| 2023 | Unspecified Meningitis | low | under 40 | 0.8 (-0.8 - 2.5) | 0.3 (-0.3 - 1.0) |
| 2023 | Unspecified Meningitis | low | 70-79 | 0.1 (-0.1 - 0.2) | 0.4 (-0.4 - 1.3) |
| 2023 | Unspecified Meningitis | low | 80-89 | 0.0 (-0.0 - 0.1) | 0.6 (-0.6 - 1.8) |
| 2023 | Unspecified Oropharynx Cancer | high | 40-69 | 0.3 (-0.3 - 1.0) | 0.1 (-0.1 - 0.3) |
| 2023 | Unspecified Oropharynx Cancer | high | 80-89 | 0.1 (-0.1 - 0.3) | 1.4 (-1.3 - 4.1) |
| 2023 | Unspecified Oropharynx Cancer | intermediate | 40-69 | 0.1 (-0.0 - 0.3) | 0.1 (-0.0 - 0.2) |
| 2023 | Unspecified Oropharynx Cancer | intermediate | 70-79 | 0.0 (-0.0 - 0.1) | 0.2 (-0.2 - 0.5) |
| 2023 | Unspecified Oropharynx Cancer | low | 80-89 | 0.1 (-0.0 - 0.1) | 1.2 (-0.5 - 2.8) |
| 2023 | Unspecified Pneumoconiosis | low | 90+ | 0.0 (-0.0 - 0.1) | 2.3 (-2.2 - 6.7) |
| 2023 | Unspecified Respiratory Cancer | intermediate | 70-79 | 0.0 (-0.0 - 0.1) | 0.2 (-0.2 - 0.5) |
| 2023 | Unspecified Road Injuries | high | 40-69 | 0.3 (-0.3 - 1.0) | 0.1 (-0.1 - 0.3) |
| 2023 | Unspecified Road Injuries | intermediate | under 40 | 0.3 (-0.3 - 0.9) | 0.0 (-0.0 - 0.1) |
| 2023 | Unspecified Road Injuries | intermediate | 40-69 | 0.1 (-0.1 - 0.2) | 0.0 (-0.0 - 0.1) |
| 2023 | Unspecified Road Injuries | intermediate | 70-79 | 0.0 (-0.0 - 0.1) | 0.2 (-0.2 - 0.5) |
| 2023 | Unspecified Road Injuries | intermediate | 90+ | 0.1 (-0.0 - 0.2) | 5.5 (-2.1 - 13.0) |
| 2023 | Unspecified Road Injuries | low | 80-89 | 0.0 (-0.0 - 0.1) | 0.6 (-0.6 - 1.8) |
| 2023 | Unspecified Site Cancer | high | 40-69 | 8.2 (5.1 - 11.3) | 2.4 (1.5 - 3.3) |
| 2023 | Unspecified Site Cancer | high | 70-79 | 7.1 (4.9 - 9.3) | 19.9 (13.6 - 26.2) |
| 2023 | Unspecified Site Cancer | high | 80-89 | 5.4 (4.0 - 6.9) | 67.7 (48.7 - 86.6) |
| 2023 | Unspecified Site Cancer | high | 90+ | 2.4 (1.3 - 3.4) | 171.0 (92.1 - 250.0) |
| 2023 | Unspecified Site Cancer | intermediate | under 40 | 1.2 (0.0 - 2.3) | 0.2 (0.0 - 0.3) |
| 2023 | Unspecified Site Cancer | intermediate | 40-69 | 5.5 (4.5 - 6.6) | 4.4 (3.6 - 5.3) |
| 2023 | Unspecified Site Cancer | intermediate | 70-79 | 8.5 (7.4 - 9.6) | 36.9 (32.0 - 41.9) |
| 2023 | Unspecified Site Cancer | intermediate | 80-89 | 5.9 (5.1 - 6.6) | 95.4 (82.8 - 107.9) |
| 2023 | Unspecified Site Cancer | intermediate | 90+ | 2.7 (2.1 - 3.3) | 207.3 (160.7 - 253.8) |
| 2023 | Unspecified Site Cancer | low | 40-69 | 3.8 (2.4 - 5.2) | 6.6 (4.1 - 9.0) |
| 2023 | Unspecified Site Cancer | low | 70-79 | 7.9 (6.5 - 9.2) | 52.1 (42.8 - 61.4) |
| 2023 | Unspecified Site Cancer | low | 80-89 | 5.2 (4.4 - 5.9) | 107.6 (92.0 - 123.2) |
| 2023 | Unspecified Site Cancer | low | 90+ | 2.4 (1.9 - 2.9) | 204.6 (162.4 - 246.9) |
| 2023 | Unspecified Thyroid Diseases | high | 80-89 | 0.1 (-0.1 - 0.3) | 1.4 (-1.3 - 4.1) |
| 2023 | Unspecified Thyroid Diseases | low | 90+ | 0.0 (-0.0 - 0.1) | 2.3 (-2.2 - 6.7) |
| 2023 | Unspecified Transport Injuries | high | 70-79 | 0.6 (-0.1 - 1.2) | 1.6 (-0.2 - 3.4) |
| 2023 | Unspecified Transport Injuries | intermediate | under 40 | 0.3 (-0.3 - 0.9) | 0.0 (-0.0 - 0.1) |
| 2023 | Unspecified Transport Injuries | intermediate | 40-69 | 0.3 (0.1 - 0.6) | 0.3 (0.1 - 0.5) |
| 2023 | Unspecified Transport Injuries | intermediate | 70-79 | 0.2 (0.0 - 0.3) | 0.7 (0.0 - 1.4) |
| 2023 | Unspecified Transport Injuries | low | under 40 | 0.8 (-0.8 - 2.5) | 0.3 (-0.3 - 1.0) |
| 2023 | Unspecified Transport Injuries | low | 40-69 | 0.5 (0.0 - 1.1) | 0.9 (0.0 - 1.9) |
| 2023 | Unspecified Urinary Cancer | high | 40-69 | 0.3 (-0.3 - 1.0) | 0.1 (-0.1 - 0.3) |
| 2023 | Unspecified Urinary Cancer | high | 70-79 | 0.2 (-0.2 - 0.6) | 0.5 (-0.5 - 1.6) |
| 2023 | Unspecified Urinary Cancer | high | 80-89 | 0.2 (-0.1 - 0.5) | 2.8 (-1.1 - 6.6) |
| 2023 | Unspecified Urinary Cancer | high | 90+ | 0.1 (-0.1 - 0.4) | 9.5 (-9.1 - 28.1) |
| 2023 | Unspecified Urinary Cancer | intermediate | 40-69 | 0.5 (0.1 - 0.8) | 0.4 (0.1 - 0.6) |
| 2023 | Unspecified Urinary Cancer | intermediate | 70-79 | 0.8 (0.4 - 1.1) | 3.3 (1.8 - 4.8) |
| 2023 | Unspecified Urinary Cancer | intermediate | 80-89 | 0.4 (0.2 - 0.7) | 7.3 (3.8 - 10.8) |
| 2023 | Unspecified Urinary Cancer | intermediate | 90+ | 0.1 (-0.0 - 0.2) | 8.2 (-1.1 - 17.4) |
| 2023 | Unspecified Urinary Cancer | low | 40-69 | 1.0 (0.3 - 1.7) | 1.6 (0.4 - 2.9) |
| 2023 | Unspecified Urinary Cancer | low | 70-79 | 0.4 (0.1 - 0.7) | 2.6 (0.5 - 4.7) |
| 2023 | Unspecified Urinary Cancer | low | 80-89 | 0.2 (0.1 - 0.3) | 4.1 (1.1 - 7.2) |
| 2023 | Unspecified Urinary Cancer | low | 90+ | 0.0 (-0.0 - 0.1) | 2.3 (-2.2 - 6.7) |
| 2023 | Unspecified Urinary Diseases | high | 70-79 | 0.2 (-0.2 - 0.6) | 0.5 (-0.5 - 1.6) |
| 2023 | Unspecified Urinary Diseases | high | 80-89 | 0.2 (-0.1 - 0.5) | 2.8 (-1.1 - 6.6) |
| 2023 | Unspecified Urinary Diseases | high | 90+ | 0.4 (-0.1 - 0.8) | 28.5 (-3.7 - 60.8) |
| 2023 | Unspecified Urinary Diseases | intermediate | 40-69 | 0.1 (-0.1 - 0.2) | 0.0 (-0.0 - 0.1) |
| 2023 | Unspecified Urinary Diseases | intermediate | 70-79 | 0.3 (0.1 - 0.5) | 1.4 (0.4 - 2.3) |
| 2023 | Unspecified Urinary Diseases | intermediate | 80-89 | 0.3 (0.1 - 0.5) | 5.2 (2.2 - 8.1) |
| 2023 | Unspecified Urinary Diseases | intermediate | 90+ | 0.3 (0.1 - 0.5) | 21.8 (6.7 - 36.9) |
| 2023 | Unspecified Urinary Diseases | low | 70-79 | 0.3 (0.0 - 0.6) | 2.2 (0.3 - 4.1) |
| 2023 | Unspecified Urinary Diseases | low | 80-89 | 0.3 (0.1 - 0.5) | 7.1 (3.1 - 11.1) |
| 2023 | Unspecified Urinary Diseases | low | 90+ | 0.1 (0.0 - 0.2) | 9.1 (0.2 - 18.0) |
| 2023 | Unspecified Uterus Cancer | high | 40-69 | 0.3 (-0.3 - 1.0) | 0.1 (-0.1 - 0.3) |
| 2023 | Unspecified Uterus Cancer | high | 70-79 | 1.3 (0.3 - 2.3) | 3.7 (1.0 - 6.4) |
| 2023 | Unspecified Uterus Cancer | high | 80-89 | 0.9 (0.3 - 1.5) | 11.0 (3.4 - 18.7) |
| 2023 | Unspecified Uterus Cancer | high | 90+ | 0.3 (-0.1 - 0.6) | 19.0 (-7.3 - 45.3) |
| 2023 | Unspecified Uterus Cancer | intermediate | 40-69 | 1.0 (0.5 - 1.4) | 0.8 (0.4 - 1.1) |
| 2023 | Unspecified Uterus Cancer | intermediate | 70-79 | 0.8 (0.5 - 1.2) | 3.6 (2.1 - 5.2) |
| 2023 | Unspecified Uterus Cancer | intermediate | 80-89 | 0.5 (0.3 - 0.8) | 8.6 (4.8 - 12.4) |
| 2023 | Unspecified Uterus Cancer | intermediate | 90+ | 0.3 (0.1 - 0.5) | 21.8 (6.7 - 36.9) |
| 2023 | Unspecified Uterus Cancer | low | 40-69 | 0.7 (0.1 - 1.3) | 1.2 (0.1 - 2.2) |
| 2023 | Unspecified Uterus Cancer | low | 70-79 | 0.5 (0.1 - 0.8) | 3.0 (0.8 - 5.3) |
| 2023 | Unspecified Uterus Cancer | low | 80-89 | 0.4 (0.2 - 0.6) | 8.3 (3.9 - 12.6) |
| 2023 | Unspecified Uterus Cancer | low | 90+ | 0.3 (0.1 - 0.4) | 22.7 (8.6 - 36.8) |
| 2023 | Unspecified Viral Diseases | high | 70-79 | 0.2 (-0.2 - 0.6) | 0.5 (-0.5 - 1.6) |
| 2023 | Unspecified Viral Diseases | high | 80-89 | 0.1 (-0.1 - 0.3) | 1.4 (-1.3 - 4.1) |
| 2023 | Unspecified Viral Diseases | intermediate | 40-69 | 0.1 (-0.1 - 0.2) | 0.0 (-0.0 - 0.1) |
| 2023 | Unspecified Viral Diseases | intermediate | 70-79 | 0.2 (0.0 - 0.3) | 0.7 (0.0 - 1.4) |
| 2023 | Unspecified Viral Diseases | intermediate | 80-89 | 0.1 (-0.0 - 0.1) | 0.9 (-0.3 - 2.1) |
| 2023 | Unspecified Viral Diseases | intermediate | 90+ | 0.2 (0.1 - 0.4) | 19.1 (4.9 - 33.2) |
| 2023 | Unspecified Viral Diseases | low | 70-79 | 0.1 (-0.1 - 0.2) | 0.4 (-0.4 - 1.3) |
| 2023 | Unspecified Viral Diseases | low | 80-89 | 0.3 (0.1 - 0.5) | 7.1 (3.1 - 11.1) |
| 2023 | Unspecified Viral Diseases | low | 90+ | 0.2 (0.0 - 0.3) | 15.9 (4.1 - 27.7) |
| 2023 | Unspecified cardiovascular diseases | high | 70-79 | 2.2 (1.0 - 3.5) | 6.3 (2.7 - 9.8) |
| 2023 | Unspecified cardiovascular diseases | high | 80-89 | 3.4 (2.3 - 4.6) | 42.8 (27.7 - 57.9) |
| 2023 | Unspecified cardiovascular diseases | high | 90+ | 2.6 (1.5 - 3.7) | 190.0 (106.8 - 273.2) |
| 2023 | Unspecified cardiovascular diseases | intermediate | 40-69 | 1.0 (0.5 - 1.4) | 0.8 (0.4 - 1.1) |
| 2023 | Unspecified cardiovascular diseases | intermediate | 70-79 | 2.3 (1.7 - 2.8) | 9.8 (7.3 - 12.4) |
| 2023 | Unspecified cardiovascular diseases | intermediate | 80-89 | 3.3 (2.7 - 3.8) | 52.8 (43.5 - 62.2) |
| 2023 | Unspecified cardiovascular diseases | intermediate | 90+ | 2.8 (2.2 - 3.5) | 220.9 (172.9 - 269.0) |
| 2023 | Unspecified cardiovascular diseases | low | 40-69 | 0.8 (0.2 - 1.5) | 1.4 (0.3 - 2.5) |
| 2023 | Unspecified cardiovascular diseases | low | 70-79 | 2.8 (1.9 - 3.6) | 18.2 (12.7 - 23.7) |
| 2023 | Unspecified cardiovascular diseases | low | 80-89 | 2.9 (2.4 - 3.5) | 60.9 (49.1 - 72.7) |
| 2023 | Unspecified cardiovascular diseases | low | 90+ | 2.6 (2.1 - 3.2) | 227.4 (182.8 - 271.9) |
| 2023 | Unspecified chronic respiratory diseases | high | 40-69 | 0.3 (-0.3 - 1.0) | 0.1 (-0.1 - 0.3) |
| 2023 | Unspecified chronic respiratory diseases | high | 70-79 | 0.2 (-0.2 - 0.6) | 0.5 (-0.5 - 1.6) |
| 2023 | Unspecified chronic respiratory diseases | high | 80-89 | 0.3 (-0.0 - 0.7) | 4.1 (-0.5 - 8.8) |
| 2023 | Unspecified chronic respiratory diseases | high | 90+ | 0.1 (-0.1 - 0.4) | 9.5 (-9.1 - 28.1) |
| 2023 | Unspecified chronic respiratory diseases | intermediate | 40-69 | 0.2 (0.0 - 0.4) | 0.2 (0.0 - 0.4) |
| 2023 | Unspecified chronic respiratory diseases | intermediate | 70-79 | 0.3 (0.1 - 0.5) | 1.4 (0.4 - 2.3) |
| 2023 | Unspecified chronic respiratory diseases | intermediate | 80-89 | 0.3 (0.1 - 0.5) | 5.2 (2.2 - 8.1) |
| 2023 | Unspecified chronic respiratory diseases | intermediate | 90+ | 0.3 (0.1 - 0.5) | 24.5 (8.5 - 40.6) |
| 2023 | Unspecified chronic respiratory diseases | low | 40-69 | 0.3 (-0.1 - 0.7) | 0.5 (-0.2 - 1.1) |
| 2023 | Unspecified chronic respiratory diseases | low | 70-79 | 0.5 (0.1 - 0.8) | 3.0 (0.8 - 5.3) |
| 2023 | Unspecified chronic respiratory diseases | low | 80-89 | 0.3 (0.1 - 0.5) | 6.5 (2.7 - 10.3) |
| 2023 | Unspecified chronic respiratory diseases | low | 90+ | 0.3 (0.1 - 0.4) | 22.7 (8.6 - 36.8) |
| 2023 | Unspecified lower respiratory infectious | high | under 40 | 4.0 (0.2 - 7.9) | 0.2 (0.0 - 0.4) |
| 2023 | Unspecified lower respiratory infectious | high | 40-69 | 3.9 (1.8 - 6.1) | 1.1 (0.5 - 1.8) |
| 2023 | Unspecified lower respiratory infectious | high | 70-79 | 5.4 (3.5 - 7.3) | 15.2 (9.7 - 20.7) |
| 2023 | Unspecified lower respiratory infectious | high | 80-89 | 5.5 (4.1 - 7.0) | 69.1 (49.9 - 88.2) |
| 2023 | Unspecified lower respiratory infectious | high | 90+ | 7.7 (5.8 - 9.6) | 560.6 (417.9 - 703.2) |
| 2023 | Unspecified lower respiratory infectious | intermediate | under 40 | 2.1 (0.6 - 3.6) | 0.3 (0.1 - 0.5) |
| 2023 | Unspecified lower respiratory infectious | intermediate | 40-69 | 5.2 (4.2 - 6.2) | 4.2 (3.3 - 5.0) |
| 2023 | Unspecified lower respiratory infectious | intermediate | 70-79 | 5.4 (4.5 - 6.3) | 23.7 (19.7 - 27.6) |
| 2023 | Unspecified lower respiratory infectious | intermediate | 80-89 | 6.6 (5.8 - 7.3) | 106.6 (93.3 - 119.8) |
| 2023 | Unspecified lower respiratory infectious | intermediate | 90+ | 7.6 (6.7 - 8.6) | 594.6 (515.9 - 673.2) |
| 2023 | Unspecified lower respiratory infectious | low | under 40 | 3.4 (0.1 - 6.6) | 1.3 (0.0 - 2.6) |
| 2023 | Unspecified lower respiratory infectious | low | 40-69 | 6.4 (4.7 - 8.2) | 11.0 (7.9 - 14.2) |
| 2023 | Unspecified lower respiratory infectious | low | 70-79 | 6.0 (4.8 - 7.2) | 39.5 (31.4 - 47.6) |
| 2023 | Unspecified lower respiratory infectious | low | 80-89 | 8.0 (7.1 - 8.8) | 165.6 (146.2 - 184.9) |
| 2023 | Unspecified lower respiratory infectious | low | 90+ | 8.3 (7.4 - 9.2) | 716.2 (637.4 - 795.0) |
| 2023 | Unspecified type of Stroke | high | under 40 | 1.0 (-1.0 - 3.0) | 0.0 (-0.0 - 0.1) |
| 2023 | Unspecified type of Stroke | high | 40-69 | 1.3 (0.0 - 2.6) | 0.4 (0.0 - 0.8) |
| 2023 | Unspecified type of Stroke | high | 70-79 | 8.0 (5.7 - 10.3) | 22.5 (15.8 - 29.3) |
| 2023 | Unspecified type of Stroke | high | 80-89 | 10.3 (8.3 - 12.3) | 128.5 (102.4 - 154.5) |
| 2023 | Unspecified type of Stroke | high | 90+ | 11.4 (9.1 - 13.6) | 826.6 (653.6 - 999.6) |
| 2023 | Unspecified type of Stroke | intermediate | 40-69 | 2.6 (1.9 - 3.4) | 2.1 (1.5 - 2.7) |
| 2023 | Unspecified type of Stroke | intermediate | 70-79 | 7.3 (6.3 - 8.3) | 31.8 (27.2 - 36.4) |
| 2023 | Unspecified type of Stroke | intermediate | 80-89 | 11.3 (10.3 - 12.3) | 183.0 (165.7 - 200.4) |
| 2023 | Unspecified type of Stroke | intermediate | 90+ | 9.9 (8.8 - 11.0) | 771.8 (682.3 - 861.4) |
| 2023 | Unspecified type of Stroke | low | 40-69 | 3.6 (2.2 - 4.9) | 6.1 (3.8 - 8.5) |
| 2023 | Unspecified type of Stroke | low | 70-79 | 8.4 (7.0 - 9.8) | 55.6 (45.9 - 65.2) |
| 2023 | Unspecified type of Stroke | low | 80-89 | 10.4 (9.4 - 11.4) | 217.0 (194.8 - 239.2) |
| 2023 | Unspecified type of Stroke | low | 90+ | 10.4 (9.4 - 11.4) | 893.5 (805.6 - 981.5) |
| 2023 | Unspecified upper respiratory infectious | high | 90+ | 0.1 (-0.1 - 0.4) | 9.5 (-9.1 - 28.1) |
| 2023 | Unspecified upper respiratory infectious | intermediate | 40-69 | 0.1 (-0.1 - 0.2) | 0.0 (-0.0 - 0.1) |
| 2023 | Unspecified upper respiratory infectious | intermediate | 80-89 | 0.1 (-0.0 - 0.2) | 1.3 (-0.2 - 2.7) |
| 2023 | Unspecified upper respiratory infectious | intermediate | 90+ | 0.1 (0.0 - 0.3) | 10.9 (0.2 - 21.6) |
| 2023 | Unspecified upper respiratory infectious | low | 70-79 | 0.1 (-0.1 - 0.2) | 0.4 (-0.4 - 1.3) |
| 2023 | Unspecified upper respiratory infectious | low | 80-89 | 0.2 (0.0 - 0.3) | 3.5 (0.7 - 6.4) |
| 2023 | Unspecified upper respiratory infectious | low | 90+ | 0.1 (-0.0 - 0.2) | 6.8 (-0.9 - 14.5) |
| 2023 | Urinary Obstruction Diseases | high | 70-79 | 0.2 (-0.2 - 0.6) | 0.5 (-0.5 - 1.6) |
| 2023 | Urinary Obstruction Diseases | high | 80-89 | 0.3 (-0.0 - 0.7) | 4.1 (-0.5 - 8.8) |
| 2023 | Urinary Obstruction Diseases | high | 90+ | 0.4 (-0.1 - 0.8) | 28.5 (-3.7 - 60.8) |
| 2023 | Urinary Obstruction Diseases | intermediate | under 40 | 0.6 (-0.2 - 1.4) | 0.1 (-0.0 - 0.2) |
| 2023 | Urinary Obstruction Diseases | intermediate | 40-69 | 0.2 (-0.0 - 0.4) | 0.1 (-0.0 - 0.3) |
| 2023 | Urinary Obstruction Diseases | intermediate | 70-79 | 0.1 (-0.0 - 0.2) | 0.3 (-0.1 - 0.8) |
| 2023 | Urinary Obstruction Diseases | intermediate | 80-89 | 0.1 (0.0 - 0.2) | 2.1 (0.3 - 4.0) |
| 2023 | Urinary Obstruction Diseases | intermediate | 90+ | 0.2 (0.0 - 0.3) | 13.6 (1.7 - 25.6) |
| 2023 | Urinary Obstruction Diseases | low | 70-79 | 0.2 (-0.0 - 0.4) | 1.3 (-0.2 - 2.8) |
| 2023 | Urinary Obstruction Diseases | low | 80-89 | 0.1 (0.0 - 0.2) | 2.4 (0.0 - 4.7) |
| 2023 | Urinary Obstruction Diseases | low | 90+ | 0.0 (-0.0 - 0.1) | 2.3 (-2.2 - 6.7) |
| 2023 | myeloid leukemia by age | high | 80-89 | 0.1 (-0.1 - 0.3) | 1.4 (-1.3 - 4.1) |
| 2023 | myeloid leukemia by age | intermediate | 40-69 | 0.1 (-0.1 - 0.2) | 0.0 (-0.0 - 0.1) |
| 2023 | myeloid leukemia by age | intermediate | 70-79 | 0.1 (-0.0 - 0.2) | 0.3 (-0.1 - 0.8) |
| 2023 | myeloid leukemia by age | intermediate | 80-89 | 0.0 (-0.0 - 0.1) | 0.4 (-0.4 - 1.3) |
| 2023 | right heart failure and pulmonary heart disease | intermediate | 70-79 | 0.0 (-0.0 - 0.1) | 0.2 (-0.2 - 0.5) |
| 2023 | right heart failure and pulmonary heart disease | low | 80-89 | 0.0 (-0.0 - 0.1) | 0.6 (-0.6 - 1.8) |
| 2023 | right heart failure and pulmonary heart disease | low | 90+ | 0.0 (-0.0 - 0.1) | 2.3 (-2.2 - 6.7) |

Table S8: Deaths before and after redistribution by year, cause, and education, 1997 and 2023

| **Table S8: Deaths before and after redistribution by year, cause, and education, 1997 and 2023** | | | | | | | |
| --- | --- | --- | --- | --- | --- | --- | --- |
| **Year** | **Cause** | **Cause level** | **Education level** | **Deaths pre-redistribution** | **Deaths post redistribution** | **Death difference** | **Percent Change** |
| 1997 | cardiovascular diseases | 3 | low | 15 500 | 20 500.0 | 4929.669 | 31.7 |
| 1997 | cardiovascular diseases | 3 | intermediate | 6 000 | 7 620.0 | 1619.428 | 27 |
| 1997 | cardiovascular diseases | 3 | high | 809 | 1 020.0 | 208.9997 | 25.8 |
| 1997 | chronic respiratory diseases | 3 | low | 1 450 | 1 610.0 | 166.9456 | 11.5 |
| 1997 | chronic respiratory diseases | 3 | high | 73 | 77.5 | 4.473729 | 6.1 |
| 1997 | chronic respiratory diseases | 3 | intermediate | 549 | 602 | 52.91113 | 9.6 |
| 1997 | diabetes and kidney diseases | 3 | low | 397 | 1 260.0 | 860.7395 | 216.8 |
| 1997 | diabetes and kidney diseases | 3 | high | 11 | 40.7 | 29.67284 | 269.8 |
| 1997 | diabetes and kidney diseases | 3 | intermediate | 159 | 423 | 264.3876 | 166.3 |
| 1997 | digestive diseases | 3 | high | 54 | 62.7 | 8.731405 | 16.2 |
| 1997 | digestive diseases | 3 | intermediate | 462 | 538 | 76.34836 | 16.5 |
| 1997 | digestive diseases | 3 | low | 1 060 | 1 240.0 | 187.0383 | 17.7 |
| 1997 | enteric infections | 3 | high | 3 | 3.34 | 0.344923 | 11.5 |
| 1997 | enteric infections | 3 | intermediate | 7 | 10.7 | 3.71489 | 53.1 |
| 1997 | enteric infections | 3 | low | 21 | 36.6 | 15.61332 | 74.3 |
| 1997 | hiv/aids and sexually transmitted infections | 3 | low | 19 | 26.1 | 7.086939 | 37.3 |
| 1997 | hiv/aids and sexually transmitted infections | 3 | intermediate | 17 | 20.5 | 3.458631 | 20.3 |
| 1997 | hiv/aids and sexually transmitted infections | 3 | high | 5 | 6.21 | 1.206745 | 24.1 |
| 1997 | maternal and neonatal disorders | 3 | intermediate | 75 | 78.3 | 3.259019 | 4.3 |
| 1997 | maternal and neonatal disorders | 3 | high | 39 | 40.9 | 1.928435 | 4.9 |
| 1997 | maternal and neonatal disorders | 3 | low | 8 | 8.16 | 0.155755 | 1.9 |
| 1997 | mental disorders | 3 | intermediate | 1 | 1 | 0 | 0 |
| 1997 | musculoskeletal disorders | 3 | high | 10 | 10.4 | 0.363779 | 3.6 |
| 1997 | musculoskeletal disorders | 3 | intermediate | 70 | 73.6 | 3.638453 | 5.2 |
| 1997 | musculoskeletal disorders | 3 | low | 182 | 192 | 9.960789 | 5.5 |
| 1997 | neglected tropical diseases and malaria | 3 | high | 1 | 1 | 0.000343 | 0 |
| 1997 | neglected tropical diseases and malaria | 3 | intermediate | 1 | 1.56 | 0.555908 | 55.6 |
| 1997 | neoplasms | 3 | low | 9 690 | 12 000.0 | 2290.961 | 23.6 |
| 1997 | neoplasms | 3 | intermediate | 5 400 | 6 440.0 | 1037.613 | 19.2 |
| 1997 | neoplasms | 3 | high | 931 | 1 110.0 | 177.8111 | 19.1 |
| 1997 | neurological disorders | 3 | high | 108 | 110 | 1.958135 | 1.8 |
| 1997 | neurological disorders | 3 | low | 1 480 | 1 500.0 | 22.04423 | 1.5 |
| 1997 | neurological disorders | 3 | intermediate | 617 | 628 | 10.50396 | 1.7 |
| 1997 | nutritional deficiencies | 3 | low | 25 | 32.6 | 7.58285 | 30.3 |
| 1997 | nutritional deficiencies | 3 | intermediate | 6 | 8.24 | 2.242737 | 37.4 |
| 1997 | other infectious diseases | 3 | high | 4 | 5.97 | 1.965339 | 49.1 |
| 1997 | other infectious diseases | 3 | intermediate | 11 | 22.3 | 11.30026 | 102.7 |
| 1997 | other infectious diseases | 3 | low | 17 | 47.6 | 30.59089 | 179.9 |
| 1997 | other non-communicable diseases | 3 | high | 53 | 57.8 | 4.75788 | 9 |
| 1997 | other non-communicable diseases | 3 | intermediate | 216 | 246 | 29.9196 | 13.9 |
| 1997 | other non-communicable diseases | 3 | low | 397 | 467 | 69.85301 | 17.6 |
| 1997 | respiratory infections and tuberculosis | 3 | low | 144 | 1 530.0 | 1383.577 | 960.8 |
| 1997 | respiratory infections and tuberculosis | 3 | high | 9 | 77.4 | 68.35396 | 759.5 |
| 1997 | respiratory infections and tuberculosis | 3 | intermediate | 69 | 467 | 397.5862 | 576.2 |
| 1997 | self-harm and interpersonal violence | 3 | intermediate | 519 | 717 | 197.5062 | 38.1 |
| 1997 | self-harm and interpersonal violence | 3 | high | 81 | 111 | 29.97014 | 37 |
| 1997 | self-harm and interpersonal violence | 3 | low | 453 | 613 | 159.7729 | 35.3 |
| 1997 | skin and subcutaneous diseases | 3 | high | 3 | 3.6 | 0.602166 | 20.1 |
| 1997 | skin and subcutaneous diseases | 3 | intermediate | 18 | 23.4 | 5.384848 | 29.9 |
| 1997 | skin and subcutaneous diseases | 3 | low | 70 | 89.3 | 19.30094 | 27.6 |
| 1997 | substance use disorders | 3 | high | 21 | 23.6 | 2.565799 | 12.2 |
| 1997 | substance use disorders | 3 | intermediate | 238 | 299 | 60.693 | 25.5 |
| 1997 | substance use disorders | 3 | low | 322 | 391 | 68.6675 | 21.3 |
| 1997 | transport injuries | 3 | low | 192 | 236 | 44.42496 | 23.1 |
| 1997 | transport injuries | 3 | high | 29 | 34.2 | 5.167304 | 17.8 |
| 1997 | transport injuries | 3 | intermediate | 291 | 328 | 36.78035 | 12.6 |
| 1997 | unintentional injuries | 3 | low | 494 | 873 | 378.6801 | 76.7 |
| 1997 | unintentional injuries | 3 | intermediate | 318 | 453 | 134.7677 | 42.4 |
| 1997 | unintentional injuries | 3 | high | 45 | 62 | 17.01383 | 37.8 |
| 2023 | cardiovascular diseases | 3 | intermediate | 8 490 | 11 800.0 | 3297.518 | 38.9 |
| 2023 | cardiovascular diseases | 3 | low | 7 680 | 10 800.0 | 3120.033 | 40.6 |
| 2023 | cardiovascular diseases | 3 | high | 1 760 | 2 550.0 | 783.9441 | 44.4 |
| 2023 | chronic respiratory diseases | 3 | intermediate | 1 990 | 2 230.0 | 237.4734 | 11.9 |
| 2023 | chronic respiratory diseases | 3 | high | 258 | 314 | 56.45855 | 21.9 |
| 2023 | chronic respiratory diseases | 3 | low | 1 710 | 1 920.0 | 214.0518 | 12.5 |
| 2023 | diabetes and kidney diseases | 3 | low | 1 360 | 1 920.0 | 560.4007 | 41.4 |
| 2023 | diabetes and kidney diseases | 3 | high | 227 | 348 | 121.3446 | 53.5 |
| 2023 | diabetes and kidney diseases | 3 | intermediate | 1 290 | 1 860.0 | 566.1586 | 43.9 |
| 2023 | digestive diseases | 3 | high | 242 | 318 | 76.3238 | 31.5 |
| 2023 | digestive diseases | 3 | intermediate | 1 380 | 1 750.0 | 371.5679 | 26.9 |
| 2023 | digestive diseases | 3 | low | 887 | 1 150.0 | 265.8303 | 30 |
| 2023 | enteric infections | 3 | low | 153 | 310 | 157.0459 | 102.6 |
| 2023 | enteric infections | 3 | intermediate | 138 | 282 | 144.0203 | 104.4 |
| 2023 | enteric infections | 3 | high | 23 | 58.3 | 35.31427 | 153.5 |
| 2023 | hiv/aids and sexually transmitted infections | 3 | intermediate | 8 | 12.3 | 4.336814 | 54.2 |
| 2023 | hiv/aids and sexually transmitted infections | 3 | low | 5 | 8.14 | 3.142449 | 62.8 |
| 2023 | maternal and neonatal disorders | 3 | high | 48 | 52.4 | 4.409523 | 9.2 |
| 2023 | maternal and neonatal disorders | 3 | intermediate | 64 | 68.6 | 4.611159 | 7.2 |
| 2023 | maternal and neonatal disorders | 3 | low | 9 | 9.14 | 0.142297 | 1.6 |
| 2023 | mental disorders | 3 | low | 2 | 2 | 0 | 0 |
| 2023 | mental disorders | 3 | intermediate | 2 | 2 | 0 | 0 |
| 2023 | mental disorders | 3 | high | 1 | 1 | 0 | 0 |
| 2023 | musculoskeletal disorders | 3 | high | 37 | 40.1 | 3.054123 | 8.3 |
| 2023 | musculoskeletal disorders | 3 | low | 123 | 133 | 10.38871 | 8.4 |
| 2023 | musculoskeletal disorders | 3 | intermediate | 175 | 187 | 12.22231 | 7 |
| 2023 | neoplasms | 3 | low | 6 530 | 7 930.0 | 1404.316 | 21.5 |
| 2023 | neoplasms | 3 | high | 3 050 | 3 550.0 | 498.219 | 16.3 |
| 2023 | neoplasms | 3 | intermediate | 11 200 | 13 300.0 | 2093.54 | 18.7 |
| 2023 | neurological disorders | 3 | high | 1 530 | 1 560.0 | 22.43304 | 1.5 |
| 2023 | neurological disorders | 3 | low | 4 670 | 4 720.0 | 51.11421 | 1.1 |
| 2023 | neurological disorders | 3 | intermediate | 5 690 | 5 760.0 | 71.50644 | 1.3 |
| 2023 | nutritional deficiencies | 3 | intermediate | 82 | 95.6 | 13.64748 | 16.6 |
| 2023 | nutritional deficiencies | 3 | low | 72 | 87.6 | 15.58609 | 21.6 |
| 2023 | nutritional deficiencies | 3 | high | 16 | 19.3 | 3.298525 | 20.6 |
| 2023 | other infectious diseases | 3 | intermediate | 64 | 107 | 42.98891 | 67.2 |
| 2023 | other infectious diseases | 3 | low | 47 | 86.9 | 39.88755 | 84.9 |
| 2023 | other infectious diseases | 3 | high | 17 | 23.7 | 6.738173 | 39.6 |
| 2023 | other non-communicable diseases | 3 | low | 581 | 711 | 130.2004 | 22.4 |
| 2023 | other non-communicable diseases | 3 | high | 127 | 168 | 41.37556 | 32.6 |
| 2023 | other non-communicable diseases | 3 | intermediate | 631 | 784 | 153.1273 | 24.3 |
| 2023 | respiratory infections and tuberculosis | 3 | intermediate | 1 130 | 2 340.0 | 1211.703 | 107.6 |
| 2023 | respiratory infections and tuberculosis | 3 | high | 265 | 534 | 269.0646 | 101.5 |
| 2023 | respiratory infections and tuberculosis | 3 | low | 1 090 | 2 330.0 | 1246.102 | 114.7 |
| 2023 | self-harm and interpersonal violence | 3 | intermediate | 786 | 1 090.0 | 304.4351 | 38.7 |
| 2023 | self-harm and interpersonal violence | 3 | high | 237 | 308 | 70.98447 | 30 |
| 2023 | self-harm and interpersonal violence | 3 | low | 248 | 361 | 112.5558 | 45.4 |
| 2023 | skin and subcutaneous diseases | 3 | high | 37 | 55.6 | 18.57172 | 50.2 |
| 2023 | skin and subcutaneous diseases | 3 | low | 153 | 241 | 87.83977 | 57.4 |
| 2023 | skin and subcutaneous diseases | 3 | intermediate | 172 | 258 | 86.25616 | 50.1 |
| 2023 | substance use disorders | 3 | intermediate | 290 | 338 | 47.73806 | 16.5 |
| 2023 | substance use disorders | 3 | high | 42 | 52.7 | 10.71899 | 25.5 |
| 2023 | substance use disorders | 3 | low | 111 | 134 | 22.93081 | 20.7 |
| 2023 | transport injuries | 3 | intermediate | 132 | 195 | 62.74351 | 47.5 |
| 2023 | transport injuries | 3 | high | 39 | 54 | 15.01557 | 38.5 |
| 2023 | transport injuries | 3 | low | 60 | 87.8 | 27.84444 | 46.4 |
| 2023 | unintentional injuries | 3 | intermediate | 826 | 1 470.0 | 643.8987 | 78 |
| 2023 | unintentional injuries | 3 | low | 579 | 1 180.0 | 601.5105 | 103.9 |

Table S9: Detailed Garbage codes accounting for greater than 1% of all death assignment by education, 1997 and 2023

| Table S9: Detailed Garbage codes accounting for greater than 1% of all death assignment by education, 1997 and 2023 | | | | | | |
| --- | --- | --- | --- | --- | --- | --- |
| **Education level** | **Year** | **Detailed garbage code** | **Deaths** | **Fraction of all deaths** | **Fraction of all deaths (CI 95%: lower value)** | **Fraction of all deaths (CI 95%: upper value)** |
| Low | 1997 | Atherosclerosis | 721 | 1.69% | 1.57% | 1.81% |
| Low | 1997 | Diabetes unspecified type | 621 | 1.46% | 1.34% | 1.57% |
| Low | 1997 | Heart failure unspecified right or left | 845 | 1.98% | 1.85% | 2.12% |
| Low | 1997 | Unspecified Site Cancer | 555 | 1.30% | 1.19% | 1.41% |
| Low | 1997 | Unspecified lower respiratory infectious | 1266 | 2.97% | 2.81% | 3.13% |
| Low | 1997 | Unspecified type of Stroke | 2020 | 4.74% | 4.54% | 4.94% |
| Low | 2023 | All, Ill Defined code for causes of death | 916 | 2.55% | 2.39% | 2.71% |
| Low | 2023 | Diabetes unspecified type | 360 | 1.00% | 0.90% | 1.11% |
| Low | 2023 | Exposure to unspecified factor X59 | 498 | 1.39% | 1.27% | 1.51% |
| Low | 2023 | Heart failure unspecified right or left | 1471 | 4.09% | 3.89% | 4.30% |
| Low | 2023 | Hypertension | 410 | 1.14% | 1.03% | 1.25% |
| Low | 2023 | Senility | 479 | 1.33% | 1.21% | 1.45% |
| Low | 2023 | Sepsis and FUO(Non- maternal and neonatal sepsis) | 425 | 1.18% | 1.07% | 1.29% |
| Low | 2023 | Unspecified Infectious Diseases | 391 | 1.09% | 0.98% | 1.20% |
| Low | 2023 | Unspecified Site Cancer | 420 | 1.17% | 1.06% | 1.28% |
| Low | 2023 | Unspecified lower respiratory infectious | 737 | 2.05% | 1.91% | 2.20% |
| Low | 2023 | Unspecified type of Stroke | 914 | 2.54% | 2.38% | 2.71% |
| Intermediate | 1997 | All, Ill Defined code for causes of death | 220 | 1.16% | 1.01% | 1.31% |
| Intermediate | 1997 | Atherosclerosis | 243 | 1.28% | 1.12% | 1.44% |
| Intermediate | 1997 | Heart failure unspecified right or left | 222 | 1.17% | 1.02% | 1.32% |
| Intermediate | 1997 | Non-follicular lymphoma, unspecified | 190 | 1.00% | 0.86% | 1.14% |
| Intermediate | 1997 | Unspecified Site Cancer | 247 | 1.30% | 1.14% | 1.46% |
| Intermediate | 1997 | Unspecified lower respiratory infectious | 363 | 1.91% | 1.72% | 2.11% |
| Intermediate | 1997 | Unspecified type of Stroke | 601 | 3.16% | 2.91% | 3.41% |
| Intermediate | 2023 | All, Ill Defined code for causes of death | 1376 | 2.99% | 2.83% | 3.14% |
| Intermediate | 2023 | Exposure to unspecified factor X59 | 501 | 1.09% | 0.99% | 1.18% |
| Intermediate | 2023 | Heart failure unspecified right or left | 1351 | 2.93% | 2.78% | 3.09% |
| Intermediate | 2023 | Unspecified Site Cancer | 614 | 1.33% | 1.23% | 1.44% |
| Intermediate | 2023 | Unspecified lower respiratory infectious | 702 | 1.52% | 1.41% | 1.64% |
| Intermediate | 2023 | Unspecified type of Stroke | 940 | 2.04% | 1.91% | 2.17% |
| High | 1997 | All, Ill Defined code for causes of death | 39 | 1.37% | 0.94% | 1.79% |
| High | 1997 | Non-follicular lymphoma, unspecified | 42 | 1.47% | 1.03% | 1.91% |
| High | 1997 | Unspecified Site Cancer | 44 | 1.54% | 1.09% | 1.99% |
| High | 1997 | Unspecified lower respiratory infectious | 63 | 2.21% | 1.67% | 2.75% |
| High | 1997 | Unspecified type of Stroke | 81 | 2.84% | 2.23% | 3.45% |
| High | 2023 | All, Ill Defined code for causes of death | 378 | 3.49% | 3.14% | 3.83% |
| High | 2023 | Exposure to unspecified factor X59 | 130 | 1.20% | 0.99% | 1.40% |
| High | 2023 | Heart failure unspecified right or left | 323 | 2.98% | 2.66% | 3.30% |
| High | 2023 | Unspecified Site Cancer | 130 | 1.20% | 0.99% | 1.40% |
| High | 2023 | Unspecified lower respiratory infectious | 154 | 1.42% | 1.20% | 1.64% |
| High | 2023 | Unspecified type of Stroke | 228 | 2.10% | 1.83% | 2.37% |

Table S10: Cumulative deaths before and after redistribution ranked by CoD burden in 2023 by highest educational attainment

| **Table S10.** Cumulative deaths before and after redistribution ranked by CoD burden in 2023 by highest educational attainment | | | | | | | |
| --- | --- | --- | --- | --- | --- | --- | --- |
| **Cause burden ranking in 2023** | **Cause of death** | **Deaths pre-redistribution** | | | **Deaths post-redistribution** | | |
|  |  | **High education** | **Intermediate education** | **Low education** | **High education** | **Intermediate education** | **Low education** |
| 1 | ischemic heart disease | 19 500 (19 300 - 19 800) | 126 000 (125 000 - 127 000) | 221 000 (220 000 - 222 000) | 25 500 (25 200 - 25 800) | 159 000 (158 000 - 159 000) | 280 000 (279 000 - 281 000) |
| 2 | alzheimer's disease and other dementias | 12 100 (11 900 - 12 400) | 59 800 (59 300 - 60 300) | 96 600 (96 000 - 97 200) | 12 200 (12 000 - 12 400) | 60 100 (59 600 - 60 500) | 97 100 (96 500 - 97 700) |
| 3 | tracheal, bronchus, and lung cancer | 6 620 (6 460 - 6 780) | 42 000 (41 600 - 42 400) | 43 900 (43 500 - 44 300) | 7 450 (7 290 - 7 620) | 46 200 (45 800 - 46 600) | 49 200 (48 700 - 49 600) |
| 4 | stroke | 6 220 (6 070 - 6 380) | 33 900 (33 500 - 34 300) | 53 400 (52 900 - 53 800) | 12 000 (11 800 - 12 200) | 67 900 (67 400 - 68 400) | 124 000 (124 000 - 125 000) |
| 5 | colon and rectum cancer | 6 840 (6 680 - 7 010) | 30 000 (29 600 - 30 300) | 33 000 (32 700 - 33 400) | 7 970 (7 800 - 8 150) | 36 100 (35 800 - 36 500) | 42 100 (41 700 - 42 600) |
| 6 | chronic obstructive pulmonary disease | 2 980 (2 870 - 3 080) | 26 000 (25 700 - 26 400) | 39 400 (39 000 - 39 800) | 3 530 (3 420 - 3 650) | 28 900 (28 600 - 29 200) | 44 400 (44 000 - 44 900) |
| 7 | severe acute respiratory syndrome coronavirus 2 | 2 030 (1 940 - 2 120) | 9 140 (8 950 - 9 330) | 8 690 (8 510 - 8 870) | 2 400 (2 300 - 2 500) | 10 900 (10 700 - 11 100) | 10 500 (10 300 - 10 700) |
| 8 | chronic kidney disease | 1 440 (1 360 - 1 510) | 7 740 (7 570 - 7 920) | 12 500 (12 300 - 12 700) | 2 640 (2 540 - 2 740) | 14 300 (14 100 - 14 600) | 23 900 (23 600 - 24 200) |
| 9 | atrial fibrillation and flutter | 3 370 (3 250 - 3 480) | 17 600 (17 400 - 17 900) | 31 500 (31 100 - 31 800) | 3 370 (3 250 - 3 480) | 17 700 (17 400 - 17 900) | 31 500 (31 200 - 31 900) |
| 10 | prostate cancer | 6 140 (5 990 - 6 290) | 24 500 (24 200 - 24 800) | 30 800 (30 500 - 31 100) | 6 910 (6 750 - 7 070) | 28 100 (27 800 - 28 400) | 36 000 (35 600 - 36 400) |
| 11 | lower respiratory infections | 449 (407 - 491) | 2 270 (2 170 - 2 360) | 3 640 (3 520 - 3 760) | 3 710 (3 590 - 3 830) | 19 700 (19 400 - 19 900) | 37 300 (37 000 - 37 700) |
| 12 | falls | 1 780 (1 690 - 1 860) | 8 550 (8 370 - 8 730) | 11 200 (11 000 - 11 400) | 3 330 (3 210 - 3 440) | 16 900 (16 600 - 17 100) | 25 600 (25 300 - 25 900) |
| 13 | pancreatic cancer | 4 740 (4 600 - 4 870) | 19 900 (19 600 - 20 200) | 18 700 (18 400 - 19 000) | 5 300 (5 160 - 5 440) | 22 900 (22 600 - 23 200) | 22 600 (22 300 - 22 900) |
| 14 | hypertensive heart disease | 1 200 (1 140 - 1 270) | 7 110 (6 940 - 7 270) | 12 800 (12 600 - 13 000) | 1 930 (1 840 - 2 010) | 11 100 (10 900 - 11 300) | 20 500 (20 300 - 20 800) |
| 15 | self-harm | 3 330 (3 220 - 3 450) | 15 700 (15 500 - 16 000) | 9 040 (8 850 - 9 220) | 4 340 (4 210 - 4 470) | 21 100 (20 800 - 21 400) | 12 100 (11 900 - 12 300) |
| 16 | breast cancer | 5 090 (4 950 - 5 230) | 17 300 (17 000 - 17 500) | 15 200 (15 000 - 15 400) | 5 460 (5 320 - 5 610) | 19 500 (19 200 - 19 700) | 18 400 (18 100 - 18 700) |
| 17 | diabetes mellitus | 862 (804 - 920) | 5 530 (5 390 - 5 680) | 8 480 (8 300 - 8 660) | 2 250 (2 150 - 2 340) | 15 500 (15 200 - 15 700) | 27 000 (26 700 - 27 300) |
| 18 | non-rheumatic valvular heart disease | 1 270 (1 200 - 1 340) | 6 940 (6 770 - 7 100) | 12 200 (12 000 - 12 400) | 1 810 (1 730 - 1 900) | 9 860 (9 660 - 10 100) | 17 800 (17 600 - 18 100) |
| 19 | aortic aneurysm | 1 940 (1 860 - 2 030) | 10 400 (10 200 - 10 600) | 13 400 (13 200 - 13 600) | 2 210 (2 110 - 2 300) | 11 800 (11 600 - 12 100) | 15 600 (15 300 - 15 800) |
| 20 | cirrhosis and other chronic liver diseases | 1 210 (1 150 - 1 280) | 8 930 (8 740 - 9 110) | 7 230 (7 060 - 7 390) | 1 470 (1 400 - 1 550) | 10 600 (10 400 - 10 900) | 8 700 (8 510 - 8 880) |
| 21 | parkinson's disease | 1 960 (1 880 - 2 050) | 6 310 (6 150 - 6 460) | 6 880 (6 710 - 7 040) | 1 970 (1 880 - 2 050) | 6 310 (6 150 - 6 470) | 6 880 (6 720 - 7 040) |
| 22 | leukemia | 1 430 (1 350 - 1 500) | 5 710 (5 560 - 5 860) | 5 330 (5 190 - 5 480) | 2 180 (2 090 - 2 270) | 9 090 (8 900 - 9 280) | 9 750 (9 560 - 9 940) |
| 23 | interstitial lung disease and pulmonary sarcoidosis | 973 (912 - 1 030) | 4 820 (4 680 - 4 960) | 5 390 (5 250 - 5 540) | 1 160 (1 100 - 1 230) | 5 820 (5 670 - 5 970) | 6 920 (6 750 - 7 080) |
| 24 | urinary diseases and male infertility | 609 (561 - 657) | 3 760 (3 640 - 3 880) | 7 080 (6 910 - 7 240) | 832 (775 - 888) | 4 910 (4 770 - 5 040) | 9 030 (8 850 - 9 220) |
| 25 | non-hodgkin lymphoma | 793 (738 - 848) | 3 110 (3 000 - 3 220) | 3 100 (2 990 - 3 210) | 2 080 (1 990 - 2 170) | 8 960 (8 780 - 9 150) | 10 500 (10 300 - 10 700) |
| 26 | other neurological disorders | 1 310 (1 240 - 1 380) | 4 740 (4 610 - 4 880) | 3 630 (3 510 - 3 750) | 1 380 (1 310 - 1 460) | 5 030 (4 890 - 5 170) | 3 910 (3 790 - 4 030) |
| 27 | brain and central nervous system cancer | 2 630 (2 530 - 2 730) | 8 230 (8 060 - 8 410) | 4 870 (4 730 - 5 000) | 2 790 (2 690 - 2 900) | 8 990 (8 810 - 9 180) | 5 510 (5 370 - 5 660) |
| 28 | stomach cancer | 1 420 (1 350 - 1 500) | 7 490 (7 320 - 7 660) | 9 020 (8 840 - 9 210) | 1 910 (1 830 - 2 000) | 10 100 (9 930 - 10 300) | 13 000 (12 700 - 13 200) |
| 29 | bladder cancer | 1 350 (1 280 - 1 420) | 7 000 (6 830 - 7 160) | 8 760 (8 580 - 8 940) | 1 580 (1 500 - 1 660) | 8 150 (7 970 - 8 330) | 10 400 (10 200 - 10 600) |
| 30 | liver cancer | 834 (777 - 891) | 4 130 (4 000 - 4 250) | 3 550 (3 430 - 3 660) | 945 (885 - 1 010) | 4 740 (4 610 - 4 880) | 4 320 (4 190 - 4 450) |
| 31 | multiple myeloma | 1 430 (1 360 - 1 510) | 5 580 (5 430 - 5 730) | 5 900 (5 750 - 6 050) | 1 690 (1 610 - 1 770) | 6 720 (6 560 - 6 890) | 7 310 (7 140 - 7 470) |
| 32 | kidney cancer | 1 380 (1 300 - 1 450) | 7 090 (6 920 - 7 250) | 8 100 (7 920 - 8 270) | 1 600 (1 520 - 1 680) | 8 230 (8 050 - 8 410) | 9 600 (9 410 - 9 790) |
| 33 | esophageal cancer | 1 000 (939 - 1 060) | 5 150 (5 010 - 5 290) | 5 030 (4 890 - 5 170) | 1 240 (1 170 - 1 300) | 6 380 (6 220 - 6 530) | 6 650 (6 490 - 6 810) |
| 34 | other malignant neoplasms | 1 070 (1 000 - 1 130) | 4 480 (4 350 - 4 620) | 4 680 (4 540 - 4 810) | 1 350 (1 280 - 1 420) | 5 850 (5 700 - 6 000) | 6 420 (6 260 - 6 570) |
| 35 | diarrheal diseases | 315 (280 - 350) | 1 760 (1 680 - 1 840) | 3 060 (2 950 - 3 170) | 707 (655 - 759) | 3 810 (3 690 - 3 930) | 6 590 (6 430 - 6 750) |
| 36 | cardiomyopathy and myocarditis | 397 (358 - 436) | 2 110 (2 020 - 2 200) | 1 720 (1 640 - 1 810) | 1 200 (1 130 - 1 260) | 6 540 (6 380 - 6 700) | 7 160 (6 990 - 7 320) |
| 37 | malignant skin melanoma | 1 650 (1 570 - 1 730) | 6 070 (5 920 - 6 220) | 4 510 (4 380 - 4 640) | 1 760 (1 680 - 1 840) | 6 590 (6 430 - 6 740) | 5 100 (4 960 - 5 240) |
| 38 | other neoplasms | 1 190 (1 120 - 1 260) | 4 630 (4 500 - 4 760) | 5 850 (5 700 - 6 000) | 1 240 (1 170 - 1 310) | 4 880 (4 740 - 5 020) | 6 220 (6 070 - 6 380) |
| 39 | endocrine, metabolic, blood, and immune disorders | 648 (598 - 698) | 3 420 (3 300 - 3 530) | 3 700 (3 580 - 3 820) | 791 (736 - 846) | 4 010 (3 890 - 4 140) | 4 550 (4 420 - 4 680) |
| 40 | gallbladder and biliary tract cancer | 1 210 (1 140 - 1 280) | 5 670 (5 520 - 5 820) | 6 470 (6 310 - 6 630) | 1 360 (1 290 - 1 430) | 6 500 (6 350 - 6 660) | 7 640 (7 460 - 7 810) |
| 41 | ovarian cancer | 1 850 (1 770 - 1 930) | 7 100 (6 930 - 7 260) | 6 190 (6 030 - 6 340) | 2 080 (1 990 - 2 170) | 8 410 (8 230 - 8 590) | 8 060 (7 880 - 8 230) |
| 42 | bacterial skin diseases | 298 (264 - 332) | 1 680 (1 600 - 1 760) | 2 670 (2 570 - 2 770) | 527 (482 - 572) | 2 940 (2 830 - 3 050) | 4 860 (4 720 - 4 990) |
| 43 | other cardiovascular and circulatory diseases | 611 (563 - 659) | 3 290 (3 170 - 3 400) | 4 440 (4 300 - 4 570) | 844 (787 - 901) | 4 590 (4 460 - 4 730) | 6 460 (6 300 - 6 610) |
| 44 | alcohol use disorders | 708 (656 - 760) | 6 080 (5 930 - 6 240) | 4 650 (4 520 - 4 790) | 803 (748 - 859) | 6 570 (6 410 - 6 730) | 5 040 (4 900 - 5 180) |
| 45 | paralytic ileus and intestinal obstruction | 474 (431 - 517) | 2 300 (2 210 - 2 400) | 3 590 (3 480 - 3 710) | 649 (600 - 699) | 3 200 (3 090 - 3 310) | 5 030 (4 890 - 5 170) |
| 46 | motor neuron disease | 1 140 (1 070 - 1 200) | 4 020 (3 900 - 4 150) | 2 980 (2 870 - 3 080) | 1 180 (1 120 - 1 250) | 4 240 (4 110 - 4 370) | 3 180 (3 070 - 3 290) |
| 47 | gallbladder and biliary diseases | 292 (259 - 325) | 1 910 (1 820 - 1 990) | 3 440 (3 330 - 3 550) | 370 (332 - 407) | 2 310 (2 220 - 2 410) | 4 110 (3 990 - 4 240) |
| 48 | uterine cancer | 448 (407 - 489) | 1 930 (1 840 - 2 020) | 2 140 (2 050 - 2 230) | 711 (659 - 763) | 3 210 (3 100 - 3 320) | 3 810 (3 680 - 3 930) |
| 49 | endocarditis | 274 (242 - 306) | 1 500 (1 430 - 1 580) | 2 050 (1 970 - 2 140) | 467 (425 - 509) | 2 570 (2 470 - 2 670) | 3 930 (3 800 - 4 050) |
| 50 | other digestive diseases | 221 (192 - 250) | 1 260 (1 190 - 1 320) | 1 640 (1 560 - 1 720) | 401 (362 - 440) | 2 240 (2 150 - 2 330) | 3 230 (3 120 - 3 340) |
| 51 | upper digestive system diseases | 503 (459 - 547) | 3 260 (3 140 - 3 370) | 4 990 (4 850 - 5 130) | 563 (516 - 609) | 3 570 (3 460 - 3 690) | 5 520 (5 380 - 5 670) |
| 52 | peripheral artery disease | 264 (232 - 296) | 1 840 (1 750 - 1 920) | 3 410 (3 300 - 3 520) | 359 (322 - 396) | 2 370 (2 280 - 2 470) | 4 440 (4 310 - 4 570) |
| 53 | congenital birth defects | 1 190 (1 120 - 1 260) | 2 480 (2 390 - 2 580) | 2 080 (1 990 - 2 170) | 1 350 (1 270 - 1 420) | 2 810 (2 700 - 2 910) | 2 200 (2 110 - 2 290) |
| 54 | road injuries | 792 (737 - 847) | 4 770 (4 630 - 4 900) | 3 050 (2 940 - 3 160) | 1 010 (949 - 1 070) | 5 890 (5 740 - 6 040) | 3 960 (3 840 - 4 090) |
| 55 | lip and oral cavity cancer | 475 (432 - 518) | 2 340 (2 240 - 2 430) | 2 120 (2 030 - 2 210) | 530 (485 - 575) | 2 620 (2 520 - 2 720) | 2 470 (2 370 - 2 560) |
| 56 | other musculoskeletal disorders | 383 (345 - 421) | 1 940 (1 850 - 2 020) | 2 610 (2 510 - 2 710) | 405 (366 - 445) | 2 060 (1 980 - 2 150) | 2 800 (2 700 - 2 900) |
| 57 | pancreatitis | 238 (208 - 268) | 1 710 (1 630 - 1 790) | 1 910 (1 830 - 2 000) | 294 (261 - 328) | 2 020 (1 930 - 2 110) | 2 300 (2 210 - 2 400) |
| 58 | vascular intestinal disorders | 292 (259 - 325) | 2 000 (1 910 - 2 080) | 3 420 (3 310 - 3 540) | 364 (327 - 402) | 2 420 (2 320 - 2 510) | 4 170 (4 050 - 4 300) |
| 59 | cervical cancer | 409 (369 - 449) | 1 810 (1 730 - 1 900) | 1 550 (1 480 - 1 630) | 719 (667 - 772) | 3 290 (3 180 - 3 400) | 3 370 (3 260 - 3 480) |
| 60 | soft tissue and other extraosseous sarcomas | 578 (531 - 625) | 1 910 (1 830 - 2 000) | 1 430 (1 350 - 1 500) | 613 (565 - 662) | 2 070 (1 980 - 2 160) | 1 630 (1 550 - 1 710) |
| 61 | mesothelioma | 262 (230 - 294) | 1 790 (1 710 - 1 870) | 1 470 (1 390 - 1 540) | 301 (267 - 335) | 1 970 (1 880 - 2 060) | 1 690 (1 610 - 1 770) |
| 62 | foreign body | 209 (181 - 237) | 1 080 (1 020 - 1 150) | 1 160 (1 100 - 1 230) | 264 (232 - 296) | 1 350 (1 280 - 1 420) | 1 560 (1 480 - 1 640) |
| 63 | epilepsy | 244 (213 - 275) | 1 290 (1 220 - 1 360) | 1 310 (1 240 - 1 380) | 286 (253 - 319) | 1 450 (1 370 - 1 520) | 1 450 (1 370 - 1 520) |
| 64 | interpersonal violence | 205 (177 - 233) | 1 200 (1 140 - 1 270) | 905 (846 - 964) | 316 (281 - 350) | 1 870 (1 790 - 1 960) | 1 280 (1 210 - 1 350) |
| 65 | protein-energy malnutrition | 148 (124 - 172) | 712 (660 - 764) | 1 020 (954 - 1 080) | 180 (154 - 206) | 871 (813 - 928) | 1 320 (1 250 - 1 390) |
| 66 | diverticular disease of intestines | 150 (126 - 174) | 996 (934 - 1 060) | 1 580 (1 500 - 1 660) | 185 (158 - 212) | 1 190 (1 120 - 1 260) | 1 890 (1 810 - 1 980) |
| 67 | multiple sclerosis | 411 (371 - 451) | 1 780 (1 700 - 1 870) | 1 310 (1 240 - 1 390) | 411 (371 - 451) | 1 780 (1 700 - 1 870) | 1 310 (1 240 - 1 390) |
| 68 | asthma | 161 (136 - 186) | 1 190 (1 120 - 1 260) | 2 330 (2 230 - 2 420) | 179 (153 - 205) | 1 280 (1 210 - 1 350) | 2 500 (2 400 - 2 600) |
| 69 | rheumatic heart disease | 201 (173 - 229) | 1 070 (1 010 - 1 140) | 1 880 (1 800 - 1 960) | 275 (242 - 307) | 1 490 (1 420 - 1 570) | 2 680 (2 580 - 2 790) |
| 70 | neonatal disorders | 1 360 (1 290 - 1 440) | 1 790 (1 700 - 1 870) | 243 (212 - 274) | 1 470 (1 390 - 1 540) | 1 960 (1 870 - 2 050) | 262 (231 - 294) |
| 71 | other pharynx cancer | 202 (174 - 230) | 1 230 (1 160 - 1 300) | 1 030 (965 - 1 090) | 220 (191 - 249) | 1 320 (1 250 - 1 390) | 1 130 (1 060 - 1 200) |
| 72 | inguinal, femoral, and abdominal hernia | 123 (101 - 145) | 630 (581 - 679) | 1 090 (1 020 - 1 150) | 144 (121 - 168) | 734 (681 - 788) | 1 260 (1 190 - 1 320) |
| 73 | non-melanoma skin cancer | 146 (122 - 170) | 708 (656 - 760) | 881 (823 - 939) | 165 (140 - 191) | 810 (755 - 866) | 1 040 (978 - 1 100) |
| 74 | rheumatoid arthritis | 180 (154 - 206) | 1 160 (1 090 - 1 230) | 2 170 (2 080 - 2 260) | 197 (170 - 225) | 1 240 (1 170 - 1 310) | 2 280 (2 190 - 2 370) |
| 75 | environmental heat and cold exposure | 230 (200 - 260) | 869 (811 - 927) | 692 (640 - 744) | 258 (227 - 290) | 1 010 (952 - 1 080) | 900 (842 - 959) |
| 76 | adverse effects of medical treatment | 234 (204 - 264) | 1 100 (1 040 - 1 170) | 1 790 (1 710 - 1 870) | 286 (253 - 319) | 1 390 (1 310 - 1 460) | 2 280 (2 180 - 2 370) |
| 77 | fire, heat, and hot substances | 154 (130 - 178) | 959 (898 - 1 020) | 860 (803 - 917) | 188 (161 - 215) | 1 140 (1 080 - 1 210) | 1 070 (1 010 - 1 140) |
| 78 | drowning | 317 (282 - 352) | 1 250 (1 180 - 1 320) | 919 (860 - 978) | 367 (329 - 404) | 1 460 (1 390 - 1 540) | 1 070 (1 010 - 1 140) |
| 79 | inflammatory bowel disease | 129 (107 - 151) | 814 (758 - 870) | 1 500 (1 430 - 1 580) | 180 (154 - 206) | 1 110 (1 040 - 1 170) | 2 040 (1 950 - 2 130) |
| 80 | thyroid cancer | 191 (164 - 218) | 806 (750 - 862) | 942 (882 - 1 000) | 207 (179 - 235) | 890 (832 - 949) | 1 070 (1 000 - 1 130) |
| 81 | exposure to mechanical forces | 71 (54.5 - 87.5) | 694 (642 - 746) | 487 (444 - 530) | 104 (84.5 - 125) | 870 (813 - 928) | 718 (665 - 771) |
| 82 | other unspecified infectious diseases | 140 (117 - 163) | 458 (416 - 500) | 339 (303 - 375) | 205 (177 - 234) | 764 (710 - 818) | 787 (732 - 842) |
| 83 | hemoglobinopathies and hemolytic anemias | 75 (58 - 92) | 349 (312 - 386) | 429 (388 - 470) | 113 (92.3 - 134) | 534 (488 - 579) | 781 (726 - 835) |
| 84 | larynx cancer | 88 (69.6 - 106) | 627 (578 - 676) | 784 (729 - 839) | 103 (82.7 - 122) | 698 (646 - 749) | 872 (814 - 930) |
| 85 | varicella and herpes zoster | 29 (18.4 - 39.6) | 120 (98.5 - 141) | 208 (180 - 236) | 50.9 (36.9 - 64.9) | 256 (224 - 287) | 486 (443 - 529) |
| 86 | malignant bone tumors | 157 (132 - 182) | 510 (466 - 554) | 413 (373 - 453) | 169 (143 - 194) | 559 (513 - 606) | 469 (426 - 511) |
| 87 | other transport injuries | 150 (126 - 174) | 835 (778 - 892) | 499 (455 - 543) | 175 (149 - 201) | 966 (905 - 1 030) | 596 (548 - 644) |
| 88 | other nutritional deficiencies | 18 (9.68 - 26.3) | 114 (93.1 - 135) | 179 (153 - 205) | 25.4 (15.5 - 35.2) | 159 (134 - 184) | 274 (242 - 307) |
| 89 | meningitis | 42 (29.3 - 54.7) | 163 (138 - 188) | 137 (114 - 160) | 103 (83.6 - 123) | 470 (428 - 513) | 449 (407 - 490) |
| 90 | other intestinal infectious diseases | 48 (34.4 - 61.6) | 248 (217 - 279) | 319 (284 - 354) | 77.3 (60 - 94.5) | 422 (382 - 462) | 661 (611 - 712) |
| 91 | encephalitis | 58 (43.1 - 72.9) | 195 (168 - 222) | 153 (129 - 177) | 61 (45.7 - 76.3) | 208 (179 - 236) | 166 (141 - 191) |
| 92 | eye cancer | 34 (22.6 - 45.4) | 73 (56.3 - 89.7) | 39 (26.8 - 51.2) | 89.4 (70.9 - 108) | 254 (222 - 285) | 205 (177 - 234) |
| 93 | hodgkin lymphoma | 93 (74.1 - 112) | 378 (340 - 416) | 398 (359 - 437) | 101 (81.4 - 121) | 420 (380 - 461) | 448 (407 - 490) |
| 94 | poisonings | 6 (1.2 - 10.8) | 64 (48.3 - 79.7) | 50 (36.1 - 63.9) | 55.3 (40.7 - 69.9) | 431 (390 - 471) | 291 (257 - 324) |
| 95 | drug use disorders | 9 (3.12 - 14.9) | 174 (148 - 200) | 207 (179 - 235) | 268 (236 - 300) | 3 500 (3 380 - 3 620) | 3 030 (2 920 - 3 140) |
| 96 | decubitus ulcer | 37 (25.1 - 48.9) | 217 (188 - 246) | 370 (332 - 408) | 44.5 (31.4 - 57.5) | 257 (226 - 289) | 439 (398 - 480) |
| 97 | other chronic respiratory diseases | 65 (49.2 - 80.8) | 238 (208 - 268) | 344 (308 - 380) | 78.9 (61.5 - 96.3) | 317 (282 - 351) | 469 (426 - 511) |
| 98 | tuberculosis | 59 (43.9 - 74.1) | 392 (353 - 431) | 756 (702 - 810) | 86.6 (68.3 - 105) | 530 (485 - 576) | 1 010 (945 - 1 070) |
| 99 | other unintentional injuries | 13 (5.93 - 20.1) | 85 (66.9 - 103) | 62 (46.6 - 77.4) | 43.8 (30.9 - 56.8) | 261 (229 - 292) | 229 (199 - 258) |
| 100 | appendicitis | 27 (16.8 - 37.2) | 151 (127 - 175) | 223 (194 - 252) | 37.6 (25.6 - 49.6) | 204 (176 - 232) | 300 (266 - 334) |
| 101 | primary pulmonary arterial hypertension | 38 (25.9 - 50.1) | 140 (117 - 163) | 165 (140 - 190) | 60.2 (45 - 75.4) | 260 (229 - 292) | 347 (310 - 383) |
| 102 | hiv/aids | 54 (39.6 - 68.4) | 249 (218 - 280) | 191 (164 - 218) | 79.2 (61.8 - 96.7) | 372 (334 - 410) | 262 (230 - 294) |
| 103 | nasopharynx cancer | 34 (22.6 - 45.4) | 180 (154 - 206) | 182 (156 - 208) | 46.1 (32.8 - 59.4) | 242 (211 - 272) | 255 (224 - 286) |
| 104 | neuroblastoma and other peripheral nervous cell tumors | 21 (12 - 30) | 66 (50.1 - 81.9) | 53 (38.7 - 67.3) | 54.7 (40.2 - 69.2) | 156 (132 - 181) | 94.5 (75.4 - 114) |
| 105 | other skin and subcutaneous diseases | 18 (9.68 - 26.3) | 85 (66.9 - 103) | 152 (128 - 176) | 18.1 (9.76 - 26.4) | 85.3 (67.2 - 103) | 153 (128 - 177) |
| 106 | pneumoconiosis | 1 (-0.96 - 2.96) | 128 (106 - 150) | 290 (257 - 323) | 5.37 (0.826 - 9.91) | 161 (136 - 185) | 356 (319 - 393) |
| 107 | animal contact | 17 (8.92 - 25.1) | 77 (59.8 - 94.2) | 69 (52.7 - 85.3) | 19.4 (10.8 - 28.1) | 90 (71.4 - 109) | 82.4 (64.6 - 100) |
| 108 | testicular cancer | 26 (16 - 36) | 142 (119 - 165) | 85 (66.9 - 103) | 31.1 (20.2 - 42.1) | 169 (144 - 195) | 111 (90.4 - 132) |
| 109 | upper respiratory infections | 16 (8.16 - 23.8) | 53 (38.7 - 67.3) | 66 (50.1 - 81.9) | 19.3 (10.7 - 27.9) | 65.1 (49.3 - 80.9) | 86 (67.8 - 104) |
| 110 | gynecological diseases | 9 (3.12 - 14.9) | 57 (42.2 - 71.8) | 72 (55.4 - 88.6) | 9.95 (3.77 - 16.1) | 62.8 (47.3 - 78.4) | 82.6 (64.8 - 100) |
| 111 | acute glomerulonephritis | 5 (0.617 - 9.38) | 24 (14.4 - 33.6) | 22 (12.8 - 31.2) | 5.26 (0.764 - 9.75) | 25.1 (15.3 - 34.9) | 23.3 (13.9 - 32.8) |
| 112 | eating disorders | 16 (8.16 - 23.8) | 51 (37 - 65) | 20 (11.2 - 28.8) | 16 (8.16 - 23.8) | 51 (37 - 65) | 20 (11.2 - 28.8) |
| 113 | other neglected tropical diseases | 1 (-0.96 - 2.96) | 8 (2.46 - 13.5) | 9 (3.12 - 14.9) | 8.51 (2.79 - 14.2) | 44.4 (31.4 - 57.5) | 72.8 (56.1 - 89.6) |
| 114 | maternal disorders | 25 (15.2 - 34.8) | 62 (46.6 - 77.4) | 18 (9.68 - 26.3) | 27.4 (17.1 - 37.7) | 67.8 (51.7 - 83.9) | 20.4 (11.5 - 29.2) |
| 115 | exposure to forces of nature | 23 (13.6 - 32.4) | 37 (25.1 - 48.9) | 2 (-0.772 - 4.77) | 23.5 (14 - 33) | 39.1 (26.9 - 51.4) | 2.9 (-0.436 - 6.24) |
| 116 | police conflict and executions | 4 (08 - 7.92) | 27 (16.8 - 37.2) | 20 (11.2 - 28.8) | 4.45 (0.315 - 8.58) | 29.3 (18.7 - 39.9) | 21 (12 - 29.9) |
| 117 | conflict and terrorism |  | 6 (1.2 - 10.8) | 7 (1.81 - 12.2) | 0.103 (-0.527 - 0.733) | 6.55 (1.53 - 11.6) | 7.33 (22 - 12.6) |
| 118 | electrocution | 5 (0.617 - 9.38) | 65 (49.2 - 80.8) | 39 (26.8 - 51.2) | 5.76 (16 - 10.5) | 67.8 (51.7 - 84) | 40.6 (28.1 - 53.1) |
| 119 | whooping cough | 5 (0.617 - 9.38) | 5 (0.617 - 9.38) | 1 (-0.96 - 2.96) | 57 (0.656 - 9.48) | 5.12 (0.686 - 9.56) | 1.21 (-0.946 - 3.37) |
| 120 | otitis media |  | 4 (08 - 7.92) | 3 (-0.395 - 6.39) | 0.00684 (-0.155 - 0.169) | 45 (0.107 - 8) | 38 (-0.36 - 6.52) |
| 121 | sexually transmitted infections excluding hiv |  | 3 (-0.395 - 6.39) | 1 (-0.96 - 2.96) | 7.33 (22 - 12.6) | 31.6 (20.6 - 42.6) | 59 (44 - 74.1) |
| 122 | leishmaniasis |  |  |  | 0.439 (-0.86 - 1.74) | 2.89 (-0.442 - 6.22) | 2.85 (-0.459 - 6.16) |
| 123 | food-borne trematodiases |  |  |  | 0.439 (-0.86 - 1.74) | 2.91 (-0.432 - 6.26) | 2.85 (-0.459 - 6.16) |
| 124 | acute hepatitis | 2 (-0.772 - 4.77) | 10 (3.8 - 16.2) | 3 (-0.395 - 6.39) | 3.82 (-013 - 7.64) | 16.6 (8.59 - 24.5) | 9.2 (3.26 - 15.2) |
| 125 | invasive non-typhoidal salmonella (ints) | 3 (-0.395 - 6.39) | 13 (5.93 - 20.1) | 10 (3.8 - 16.2) | 4.37 (0.275 - 8.47) | 19 (10.5 - 27.6) | 19.4 (10.8 - 28) |
| 126 | typhoid and paratyphoid |  | 1 (-0.96 - 2.96) |  | 0.132 (-0.58 - 0.845) | 1.39 (-0.92 - 3.71) | 0.421 (-0.851 - 1.69) |
| 127 | diphtheria | 1 (-0.96 - 2.96) |  | 1 (-0.96 - 2.96) | 1.12 (-0.955 - 3.19) | 0.117 (-0.553 - 0.786) | 1.26 (-0.94 - 3.46) |
| 128 | tetanus |  |  | 3 (-0.395 - 6.39) | 0.165 (-0.631 - 0.96) | 0.727 (-0.944 - 2.4) | 4.8 (0.508 - 9.1) |
| 129 | rabies |  |  | 1 (-0.96 - 2.96) | 0.204 (-0.681 - 19) | 0.218 (-0.697 - 1.13) | 1.12 (-0.954 - 3.19) |

Table S11: Deaths before and after redistribution by highest educational attainment, 1997 & 2023

| **Table S11.** Deaths before and after redistribution by highest educational attainment, 1997 & 2023 | | | | | | | |
| --- | --- | --- | --- | --- | --- | --- | --- |
| **Cause of death** | **Year** | **Deaths pre-redistribution** | | | **Deaths post-redistribution** | | |
|  |  | **High education** | **Intermediate education** | **Low education** | **High education** | **Intermediate education** | **Low education** |
| acute glomerulonephritis | 2023 |  | 4 (08 - 7.92) | 2 (-0.772 - 4.77) | 0.0461 (-0.375 - 0.467) | 4.19 (0.176 - 8.2) | 2.12 (-0.735 - 4.97) |
| acute hepatitis | 2023 |  |  |  | 0.0167 (-0.236 - 0.27) | 0.0995 (-0.519 - 0.718) | 0.0337 (-0.326 - 0.394) |
| adverse effects of medical treatment | 2023 | 4 (08 - 7.92) | 43 (30.1 - 55.9) | 26 (16 - 36) | 8.43 (2.74 - 14.1) | 59.8 (44.6 - 74.9) | 41.5 (28.9 - 54.2) |
| alcohol use disorders | 2023 | 42 (29.3 - 54.7) | 288 (255 - 321) | 109 (88.5 - 129) | 48.6 (34.9 - 62.3) | 317 (282 - 352) | 124 (102 - 146) |
| alzheimer's disease and other dementias | 2023 | 1 080 (1 020 - 1 150) | 4 420 (4 290 - 4 550) | 4 070 (3 940 - 4 190) | 1 090 (1 020 - 1 150) | 4 440 (4 310 - 4 570) | 4 080 (3 960 - 4 210) |
| animal contact | 2023 | 1 (-0.96 - 2.96) | 4 (08 - 7.92) | 2 (-0.772 - 4.77) | 1.18 (-0.949 - 3.31) | 4.8 (0.503 - 99) | 2.51 (-0.596 - 5.61) |
| aortic aneurysm | 2023 | 108 (87.6 - 128) | 422 (382 - 462) | 285 (252 - 318) | 126 (104 - 148) | 501 (457 - 544) | 345 (308 - 381) |
| appendicitis | 2023 | 1 (-0.96 - 2.96) | 11 (4.5 - 17.5) | 5 (0.617 - 9.38) | 1.73 (-0.848 - 4.31) | 14.6 (78 - 22) | 7.6 (2.19 - 13) |
| asthma | 2023 | 11 (4.5 - 17.5) | 65 (49.2 - 80.8) | 49 (35.3 - 62.7) | 12.4 (5.53 - 19.4) | 70.7 (54.2 - 87.1) | 55.3 (40.7 - 69.8) |
| atrial fibrillation and flutter | 2023 | 268 (236 - 300) | 1 100 (1 040 - 1 170) | 1 200 (1 130 - 1 270) | 268 (236 - 300) | 1 100 (1 040 - 1 170) | 1 200 (1 130 - 1 270) |
| bacterial skin diseases | 2023 | 33 (21.7 - 44.3) | 151 (127 - 175) | 140 (117 - 163) | 50.8 (36.9 - 64.8) | 234 (204 - 264) | 225 (196 - 254) |
| bladder cancer | 2023 | 68 (51.8 - 84.2) | 330 (294 - 366) | 245 (214 - 276) | 80.1 (62.6 - 97.7) | 387 (349 - 426) | 285 (252 - 318) |
| brain and central nervous system cancer | 2023 | 162 (137 - 187) | 391 (352 - 430) | 143 (120 - 166) | 173 (147 - 199) | 433 (393 - 474) | 164 (139 - 189) |
| breast cancer | 2023 | 276 (243 - 309) | 691 (639 - 743) | 391 (352 - 430) | 295 (261 - 329) | 769 (714 - 823) | 453 (411 - 494) |
| cardiomyopathy and myocarditis | 2023 | 25 (15.2 - 34.8) | 101 (81.3 - 121) | 56 (41.3 - 70.7) | 73.8 (57 - 90.7) | 300 (266 - 334) | 209 (181 - 238) |
| cervical cancer | 2023 | 15 (7.41 - 22.6) | 70 (53.6 - 86.4) | 33 (21.7 - 44.3) | 31 (20.1 - 41.9) | 131 (109 - 154) | 68 (51.9 - 84.2) |
| chronic kidney disease | 2023 | 143 (120 - 166) | 754 (700 - 808) | 863 (805 - 921) | 237 (207 - 268) | 1 200 (1 140 - 1 270) | 1 320 (1 240 - 1 390) |
| chronic obstructive pulmonary disease | 2023 | 171 (145 - 197) | 1 540 (1 460 - 1 610) | 1 380 (1 310 - 1 450) | 211 (183 - 240) | 1 700 (1 620 - 1 780) | 1 530 (1 450 - 1 610) |
| cirrhosis and other chronic liver diseases | 2023 | 82 (64.3 - 99.7) | 489 (446 - 532) | 218 (189 - 247) | 97 (77.7 - 116) | 590 (542 - 637) | 260 (229 - 292) |
| colon and rectum cancer | 2023 | 407 (367 - 447) | 1 550 (1 470 - 1 630) | 942 (882 - 1 000) | 477 (434 - 520) | 1 850 (1 770 - 1 940) | 1 160 (1 100 - 1 230) |
| conflict and terrorism | 2023 |  |  | 1 (-0.96 - 2.96) | 0.0378 (-0.343 - 0.419) | 0.103 (-0.525 - 0.731) | 13 (-0.959 - 32) |
| congenital birth defects | 2023 | 39 (26.8 - 51.2) | 86 (67.8 - 104) | 100 (80.4 - 120) | 46 (32.7 - 59.3) | 105 (85.2 - 125) | 106 (85.7 - 126) |
| decubitus ulcer | 2023 | 3 (-0.395 - 6.39) | 15 (7.41 - 22.6) | 9 (3.12 - 14.9) | 3.74 (-0502 - 7.53) | 17.8 (9.51 - 26) | 11.9 (5.17 - 18.7) |
| diabetes mellitus | 2023 | 84 (66 - 102) | 531 (486 - 576) | 490 (447 - 533) | 111 (90.2 - 131) | 647 (597 - 697) | 598 (550 - 646) |
| diarrheal diseases | 2023 | 21 (12 - 30) | 129 (107 - 151) | 142 (119 - 165) | 54.9 (40.3 - 69.4) | 265 (233 - 297) | 289 (255 - 322) |
| diphtheria | 2023 |  |  |  |  |  | 0.0263 (-0.292 - 0.344) |
| diverticular disease of intestines | 2023 | 8 (2.46 - 13.5) | 67 (51 - 83) | 43 (30.1 - 55.9) | 11.3 (4.71 - 17.9) | 78.8 (61.4 - 96.2) | 52.8 (38.5 - 67) |
| drowning | 2023 | 17 (8.92 - 25.1) | 34 (22.6 - 45.4) | 13 (5.93 - 20.1) | 20.4 (11.6 - 29.3) | 42.5 (29.7 - 55.3) | 17.1 (8.98 - 25.2) |
| drug use disorders | 2023 |  | 2 (-0.772 - 4.77) | 2 (-0.772 - 4.77) | 4.12 (0.143 - 8.1) | 20.6 (11.7 - 29.5) | 10.2 (3.94 - 16.5) |
| eating disorders | 2023 | 1 (-0.96 - 2.96) | 2 (-0.772 - 4.77) | 2 (-0.772 - 4.77) | 1 (-0.96 - 2.96) | 2 (-0.772 - 4.77) | 2 (-0.772 - 4.77) |
| electrocution | 2023 |  |  | 1 (-0.96 - 2.96) | 0.0301 (-0.31 - 0.37) | 0.0964 (-0.512 - 0.705) | 14 (-0.959 - 34) |
| encephalitis | 2023 | 8 (2.46 - 13.5) | 17 (8.92 - 25.1) | 12 (5.21 - 18.8) | 8.13 (2.54 - 13.7) | 18.1 (9.74 - 26.4) | 12.4 (5.49 - 19.3) |
| endocarditis | 2023 | 19 (10.5 - 27.5) | 94 (75 - 113) | 76 (58.9 - 93.1) | 30.6 (19.8 - 41.5) | 150 (126 - 174) | 127 (105 - 149) |
| endocrine, metabolic, blood, and immune disorders | 2023 | 38 (25.9 - 50.1) | 219 (190 - 248) | 190 (163 - 217) | 49.9 (36.1 - 63.8) | 259 (227 - 290) | 225 (196 - 254) |
| environmental heat and cold exposure | 2023 | 6 (1.2 - 10.8) | 55 (40.5 - 69.5) | 30 (19.3 - 40.7) | 8.6 (2.85 - 14.3) | 64.6 (48.8 - 80.3) | 37.7 (25.7 - 49.7) |
| epilepsy | 2023 | 14 (6.67 - 21.3) | 73 (56.3 - 89.7) | 50 (36.1 - 63.9) | 17.9 (9.58 - 26.2) | 83 (65.1 - 101) | 56.2 (41.5 - 70.8) |
| esophageal cancer | 2023 | 72 (55.4 - 88.6) | 281 (248 - 314) | 160 (135 - 185) | 86.6 (68.4 - 105) | 348 (311 - 385) | 202 (175 - 230) |
| exposure to forces of nature | 2023 | 1 (-0.96 - 2.96) |  |  | 16 (-0.958 - 39) | 0.11 (-0.54 - 0.76) | 0.0183 (-0.247 - 0.284) |
| exposure to mechanical forces | 2023 | 4 (08 - 7.92) | 40 (27.6 - 52.4) | 14 (6.67 - 21.3) | 6.83 (1.71 - 11.9) | 51 (37 - 65) | 22.3 (13 - 31.5) |
| eye cancer | 2023 | 7 (1.81 - 12.2) | 11 (4.5 - 17.5) | 3 (-0.395 - 6.39) | 10.2 (3.96 - 16.5) | 17.8 (9.56 - 26.1) | 7.41 (27 - 12.7) |
| falls | 2023 | 142 (119 - 165) | 529 (484 - 574) | 433 (392 - 474) | 287 (254 - 321) | 1 070 (1 010 - 1 130) | 966 (905 - 1 030) |
| fire, heat, and hot substances | 2023 | 5 (0.617 - 9.38) | 54 (39.6 - 68.4) | 14 (6.67 - 21.3) | 7.6 (2.2 - 13) | 63.8 (48.2 - 79.5) | 19.8 (11.1 - 28.5) |
| food-borne trematodiases | 2023 |  |  |  |  | 0.2 (-0.677 - 18) | 72e-05 (-0163 - 0165) |
| foreign body | 2023 | 22 (12.8 - 31.2) | 59 (43.9 - 74.1) | 41 (28.4 - 53.6) | 26.9 (16.7 - 37) | 77.3 (60.1 - 94.6) | 56 (41.3 - 70.7) |
| gallbladder and biliary diseases | 2023 | 26 (16 - 36) | 134 (111 - 157) | 144 (120 - 168) | 32.5 (21.3 - 43.6) | 163 (138 - 188) | 172 (147 - 198) |
| gallbladder and biliary tract cancer | 2023 | 72 (55.4 - 88.6) | 255 (224 - 286) | 137 (114 - 160) | 80.6 (63 - 98.2) | 294 (260 - 327) | 162 (137 - 187) |
| gynecological diseases | 2023 |  | 4 (08 - 7.92) | 2 (-0.772 - 4.77) | 0.0666 (-0.439 - 0.573) | 4.34 (0.258 - 8.43) | 2.29 (-0.678 - 5.25) |
| hemoglobinopathies and hemolytic anemias | 2023 | 6 (1.2 - 10.8) | 20 (11.2 - 28.8) | 13 (5.93 - 20.1) | 9.14 (3.21 - 15.1) | 32.8 (21.6 - 44) | 25.8 (15.8 - 35.7) |
| hiv/aids | 2023 |  | 8 (2.46 - 13.5) | 5 (0.617 - 9.38) | 1.28 (-0.938 - 3.49) | 12.2 (5.32 - 19) | 7.68 (2.25 - 13.1) |
| hodgkin lymphoma | 2023 | 6 (1.2 - 10.8) | 15 (7.41 - 22.6) | 12 (5.21 - 18.8) | 6.42 (1.45 - 11.4) | 16.7 (8.68 - 24.7) | 13.1 (5.97 - 20.1) |
| hypertensive heart disease | 2023 | 110 (89.4 - 131) | 449 (407 - 491) | 575 (528 - 622) | 180 (153 - 206) | 738 (684 - 791) | 905 (846 - 964) |
| inflammatory bowel disease | 2023 | 4 (08 - 7.92) | 33 (21.7 - 44.3) | 15 (7.41 - 22.6) | 7.74 (2.29 - 13.2) | 47.5 (34 - 61) | 27.2 (17 - 37.5) |
| inguinal, femoral, and abdominal hernia | 2023 | 7 (1.81 - 12.2) | 49 (35.3 - 62.7) | 45 (31.9 - 58.1) | 8.85 (32 - 14.7) | 57.1 (42.3 - 71.9) | 52.4 (38.2 - 66.6) |
| interpersonal violence | 2023 | 17 (8.92 - 25.1) | 64 (48.3 - 79.7) | 26 (16 - 36) | 24.2 (14.6 - 33.9) | 98.2 (78.7 - 118) | 36.4 (24.6 - 48.2) |
| interstitial lung disease and pulmonary sarcoidosis | 2023 | 73 (56.3 - 89.7) | 368 (330 - 406) | 268 (236 - 300) | 87.1 (68.8 - 105) | 432 (391 - 473) | 321 (286 - 356) |
| invasive non-typhoidal salmonella (ints) | 2023 |  |  |  | 0.00733 (-0.16 - 0.175) | 0.03 (-0.309 - 0.369) | 0.0543 (-0.403 - 0.511) |
| ischemic heart disease | 2023 | 797 (742 - 852) | 4 240 (4 110 - 4 360) | 3 810 (3 690 - 3 930) | 1 200 (1 130 - 1 270) | 5 990 (5 840 - 6 140) | 5 460 (5 310 - 5 600) |
| kidney cancer | 2023 | 74 (57.1 - 90.9) | 296 (262 - 330) | 170 (144 - 196) | 85.3 (67.2 - 103) | 354 (317 - 391) | 205 (177 - 233) |
| larynx cancer | 2023 | 3 (-0.395 - 6.39) | 29 (18.4 - 39.6) | 30 (19.3 - 40.7) | 3.79 (-0273 - 7.6) | 32.2 (21.1 - 43.4) | 32.2 (21.1 - 43.3) |
| leishmaniasis | 2023 |  |  |  |  | 0.2 (-0.677 - 18) | 72e-05 (-0.0163 - 0/0165) |
| leukemia | 2023 | 95 (75.9 - 114) | 326 (291 - 361) | 151 (127 - 175) | 126 (104 - 148) | 465 (423 - 508) | 244 (213 - 274) |
| lip and oral cavity cancer | 2023 | 38 (25.9 - 50.1) | 122 (100 - 144) | 76 (58.9 - 93.1) | 41.8 (29.2 - 54.5) | 135 (112 - 158) | 85.3 (67.2 - 103) |
| liver cancer | 2023 | 74 (57.1 - 90.9) | 326 (291 - 361) | 171 (145 - 197) | 81.3 (63.7 - 99) | 364 (327 - 401) | 193 (166 - 221) |
| lower respiratory infections | 2023 | 41 (28.4 - 53.6) | 153 (129 - 177) | 158 (133 - 183) | 234 (204 - 264) | 1 010 (952 - 1 080) | 1 060 (992 - 1 120) |
| malignant bone tumors | 2023 | 11 (4.5 - 17.5) | 28 (17.6 - 38.4) | 9 (3.12 - 14.9) | 11.9 (5.16 - 18.7) | 30.6 (19.8 - 41.5) | 10 (3.83 - 16.2) |
| malignant skin melanoma | 2023 | 87 (68.7 - 105) | 289 (256 - 322) | 141 (118 - 164) | 92.7 (73.8 - 112) | 312 (278 - 347) | 156 (132 - 181) |
| maternal disorders | 2023 |  | 2 (-0.772 - 4.77) | 1 (-0.96 - 2.96) | 0.0889 (-0.495 - 0.673) | 2.31 (-0.668 - 5.29) | 18 (-0.957 - 3.11) |
| meningitis | 2023 | 2 (-0.772 - 4.77) | 8 (2.46 - 13.5) | 7 (1.81 - 12.2) | 3.75 (-0467 - 7.54) | 21.5 (12.4 - 30.6) | 16.7 (8.68 - 24.7) |
| mesothelioma | 2023 | 26 (16 - 36) | 77 (59.8 - 94.2) | 47 (33.6 - 60.4) | 28.6 (18.1 - 39.1) | 86.9 (68.6 - 105) | 53.3 (39 - 67.6) |
| motor neuron disease | 2023 | 93 (74.1 - 112) | 233 (203 - 263) | 93 (74.1 - 112) | 97.8 (78.4 - 117) | 252 (221 - 283) | 104 (84 - 124) |
| multiple myeloma | 2023 | 73 (56.3 - 89.7) | 271 (239 - 303) | 158 (133 - 183) | 90.3 (71.7 - 109) | 343 (307 - 380) | 213 (184 - 242) |
| multiple sclerosis | 2023 | 14 (6.67 - 21.3) | 89 (70.5 - 107) | 41 (28.4 - 53.6) | 14 (6.67 - 21.3) | 89 (70.5 - 107) | 41 (28.4 - 53.6) |
| nasopharynx cancer | 2023 | 3 (-0.395 - 6.39) | 7 (1.81 - 12.2) | 7 (1.81 - 12.2) | 3.74 (-049 - 7.53) | 8.51 (2.79 - 14.2) | 7.94 (2.42 - 13.5) |
| neonatal disorders | 2023 | 48 (34.4 - 61.6) | 62 (46.6 - 77.4) | 8 (2.46 - 13.5) | 52.3 (38.1 - 66.5) | 66.3 (50.3 - 82.3) | 87 (2.5 - 13.6) |
| neuroblastoma and other peripheral nervous cell tumors | 2023 | 1 (-0.96 - 2.96) | 2 (-0.772 - 4.77) | 1 (-0.96 - 2.96) | 36 (-0.369 - 6.49) | 62 (1.21 - 10.8) | 2.68 (-0.529 - 5.89) |
| non-hodgkin lymphoma | 2023 | 65 (49.2 - 80.8) | 190 (163 - 217) | 116 (94.9 - 137) | 129 (107 - 151) | 406 (366 - 445) | 256 (224 - 287) |
| non-melanoma skin cancer | 2023 | 10 (3.8 - 16.2) | 54 (39.6 - 68.4) | 41 (28.4 - 53.6) | 11.3 (4.73 - 17.9) | 59 (44 - 74.1) | 45.5 (32.3 - 58.7) |
| non-rheumatic valvular heart disease | 2023 | 85 (66.9 - 103) | 398 (359 - 437) | 432 (391 - 473) | 131 (109 - 154) | 588 (540 - 636) | 638 (589 - 688) |
| other cardiovascular and circulatory diseases | 2023 | 39 (26.8 - 51.2) | 175 (149 - 201) | 131 (109 - 153) | 55.7 (41.1 - 70.3) | 248 (217 - 279) | 187 (160 - 214) |
| other chronic respiratory diseases | 2023 | 3 (-0.395 - 6.39) | 13 (5.93 - 20.1) | 11 (4.5 - 17.5) | 3.71 (-0667 - 7.48) | 15.7 (7.93 - 23.5) | 13.8 (6.49 - 21) |
| other digestive diseases | 2023 | 14 (6.67 - 21.3) | 85 (66.9 - 103) | 48 (34.4 - 61.6) | 30 (19.3 - 40.8) | 159 (134 - 183) | 106 (85.8 - 126) |
| other intestinal infectious diseases | 2023 | 2 (-0.772 - 4.77) | 9 (3.12 - 14.9) | 11 (4.5 - 17.5) | 3.45 (-0.192 - 79) | 16.9 (8.88 - 25) | 21.2 (12.2 - 30.3) |
| other malignant neoplasms | 2023 | 78 (60.7 - 95.3) | 255 (224 - 286) | 146 (122 - 170) | 92.2 (73.3 - 111) | 335 (299 - 371) | 197 (170 - 225) |
| other musculoskeletal disorders | 2023 | 26 (16 - 36) | 122 (100 - 144) | 82 (64.3 - 99.7) | 27.7 (17.4 - 38) | 129 (107 - 151) | 88.3 (69.9 - 107) |
| other neglected tropical diseases | 2023 |  |  |  | 0.471 (-0.874 - 1.82) | 2.11 (-0.738 - 4.95) | 28 (-0.748 - 4.9) |
| other neoplasms | 2023 | 81 (63.4 - 98.6) | 256 (225 - 287) | 185 (158 - 212) | 85.8 (67.7 - 104) | 274 (242 - 307) | 200 (172 - 227) |
| other neurological disorders | 2023 | 131 (109 - 153) | 424 (384 - 464) | 165 (140 - 190) | 140 (117 - 164) | 451 (409 - 492) | 182 (155 - 208) |
| other nutritional deficiencies | 2023 | 2 (-0.772 - 4.77) | 19 (10.5 - 27.5) | 14 (6.67 - 21.3) | 2.49 (-0.601 - 5.59) | 21.5 (12.4 - 30.6) | 16.4 (8.45 - 24.3) |
| other pharynx cancer | 2023 | 16 (8.16 - 23.8) | 72 (55.4 - 88.6) | 32 (20.9 - 43.1) | 17 (8.95 - 25.1) | 75.2 (58.2 - 92.3) | 34.1 (22.6 - 45.5) |
| other skin and subcutaneous diseases | 2023 | 1 (-0.96 - 2.96) | 6 (1.2 - 10.8) | 4 (08 - 7.92) | 1 (-0.96 - 2.96) | 6 (1.2 - 10.8) | 4 (082 - 7.93) |
| other transport injuries | 2023 | 3 (-0.395 - 6.39) | 23 (13.6 - 32.4) | 8 (2.46 - 13.5) | 4.83 (0.521 - 9.13) | 30.3 (19.5 - 41.1) | 10.8 (4.37 - 17.3) |
| other unintentional injuries | 2023 | 3 (-0.395 - 6.39) | 3 (-0.395 - 6.39) | 3 (-0.395 - 6.39) | 4.96 (0.597 - 9.33) | 12.1 (5.26 - 18.9) | 8.18 (2.57 - 13.8) |
| other unspecified infectious diseases | 2023 | 6 (1.2 - 10.8) | 26 (16 - 36) | 12 (5.21 - 18.8) | 9.15 (3.22 - 15.1) | 43 (30.2 - 55.9) | 25.8 (15.9 - 35.8) |
| otitis media | 2023 |  | 1 (-0.96 - 2.96) |  | 0.000726 (-0.0521 - 0.0535) | 1 (-0.96 - 2.97) | 0.00275 (-0.1 - 0.106) |
| ovarian cancer | 2023 | 87 (68.7 - 105) | 231 (201 - 261) | 93 (74.1 - 112) | 101 (81 - 120) | 287 (254 - 320) | 128 (106 - 150) |
| pancreatic cancer | 2023 | 333 (297 - 369) | 1 140 (1 070 - 1 210) | 553 (507 - 599) | 366 (329 - 404) | 1 290 (1 220 - 1 360) | 649 (599 - 699) |
| pancreatitis | 2023 | 20 (11.2 - 28.8) | 108 (87.6 - 128) | 69 (52.7 - 85.3) | 24.5 (14.8 - 34.2) | 130 (107 - 152) | 84.8 (66.8 - 103) |
| paralytic ileus and intestinal obstruction | 2023 | 41 (28.4 - 53.6) | 178 (152 - 204) | 122 (100 - 144) | 57.4 (42.5 - 72.2) | 246 (216 - 277) | 177 (151 - 203) |
| parkinson's disease | 2023 | 196 (169 - 223) | 451 (409 - 493) | 254 (223 - 285) | 196 (169 - 223) | 451 (410 - 493) | 254 (223 - 285) |
| peripheral artery disease | 2023 | 20 (11.2 - 28.8) | 112 (91.3 - 133) | 88 (69.6 - 106) | 27.5 (17.2 - 37.8) | 144 (121 - 168) | 120 (98.9 - 142) |
| pneumoconiosis | 2023 |  | 7 (1.81 - 12.2) | 1 (-0.96 - 2.96) | 0.131 (-0.578 - 0.841) | 7.43 (29 - 12.8) | 2.3 (-0.673 - 5.27) |
| poisonings | 2023 | 1 (-0.96 - 2.96) | 5 (0.617 - 9.38) | 2 (-0.772 - 4.77) | 3.41 (-0.208 - 74) | 23.6 (14.1 - 33.1) | 8.36 (2.69 - 14) |
| police conflict and executions | 2023 |  | 1 (-0.96 - 2.96) |  | 0.0233 (-0.276 - 0.322) | 1.14 (-0.953 - 3.24) | 0.0144 (-0.221 - 0.25) |
| primary pulmonary arterial hypertension | 2023 | 1 (-0.96 - 2.96) | 3 (-0.395 - 6.39) | 2 (-0.772 - 4.77) | 2.52 (-0.591 - 5.63) | 9.77 (3.64 - 15.9) | 8.38 (2.7 - 14) |
| prostate cancer | 2023 | 286 (253 - 319) | 1 110 (1 050 - 1 180) | 793 (738 - 848) | 320 (285 - 355) | 1 240 (1 170 - 1 310) | 889 (831 - 948) |
| protein-energy malnutrition | 2023 | 14 (6.67 - 21.3) | 63 (47.4 - 78.6) | 58 (43.1 - 72.9) | 16.8 (8.77 - 24.8) | 74.1 (57.2 - 91) | 71.2 (54.7 - 87.7) |
| rabies | 2023 |  |  |  |  | 7.97e-05 (-0.0174 - 0.0176) |  |
| rheumatic heart disease | 2023 | 16 (8.16 - 23.8) | 39 (26.8 - 51.2) | 26 (16 - 36) | 21.5 (12.4 - 30.6) | 62.3 (46.8 - 77.7) | 49.9 (36 - 63.7) |
| rheumatoid arthritis | 2023 | 11 (4.5 - 17.5) | 53 (38.7 - 67.3) | 41 (28.4 - 53.6) | 12.3 (5.45 - 19.2) | 58.1 (43.2 - 73.1) | 45.1 (31.9 - 58.3) |
| road injuries | 2023 | 36 (24.2 - 47.8) | 109 (88.5 - 129) | 52 (37.9 - 66.1) | 49.2 (35.4 - 62.9) | 164 (139 - 190) | 77 (59.8 - 94.2) |
| self-harm | 2023 | 220 (191 - 249) | 721 (668 - 774) | 221 (192 - 250) | 284 (251 - 317) | 991 (929 - 1 050) | 323 (288 - 358) |
| severe acute respiratory syndrome coronavirus 2 | 2023 | 220 (191 - 249) | 963 (902 - 1 020) | 922 (862 - 982) | 295 (261 - 328) | 1 310 (1 240 - 1 380) | 1 270 (1 200 - 1 330) |
| sexually transmitted infections excluding hiv | 2023 |  |  |  | 0.168 (-0.635 - 0.972) | 0.186 (-0.66 - 13) | 0.458 (-0.868 - 1.78) |
| soft tissue and other extraosseous sarcomas | 2023 | 45 (31.9 - 58.1) | 98 (78.6 - 117) | 40 (27.6 - 52.4) | 47.3 (33.8 - 60.8) | 107 (86.4 - 127) | 44.6 (31.5 - 57.7) |
| stomach cancer | 2023 | 67 (51 - 83) | 300 (266 - 334) | 151 (127 - 175) | 95.8 (76.6 - 115) | 426 (386 - 467) | 238 (208 - 269) |
| stroke | 2023 | 277 (244 - 310) | 1 360 (1 280 - 1 430) | 998 (936 - 1 060) | 431 (391 - 472) | 1 950 (1 860 - 2 040) | 1 550 (1 470 - 1 630) |
| testicular cancer | 2023 | 2 (-0.772 - 4.77) | 4 (08 - 7.92) |  | 2.11 (-0.739 - 4.95) | 5 (0.615 - 9.38) | 0.189 (-0.663 - 14) |
| tetanus | 2023 |  |  |  |  |  | 0.0263 (-0.292 - 0.344) |
| thyroid cancer | 2023 | 13 (5.93 - 20.1) | 36 (24.2 - 47.8) | 24 (14.4 - 33.6) | 13.9 (6.58 - 21.2) | 39.7 (27.3 - 52) | 27 (16.8 - 37.2) |
| tracheal, bronchus, and lung cancer | 2023 | 365 (328 - 402) | 1 990 (1 900 - 2 070) | 1 260 (1 190 - 1 330) | 413 (373 - 453) | 2 200 (2 110 - 2 300) | 1 400 (1 330 - 1 480) |
| tuberculosis | 2023 | 2 (-0.772 - 4.77) | 8 (2.46 - 13.5) | 4 (08 - 7.92) | 3.2 (-0.306 - 6.71) | 13.3 (6.15 - 20.5) | 88 (2.51 - 13.7) |
| typhoid and paratyphoid | 2023 |  |  |  |  |  | 0.0526 (-0.397 - 0.502) |
| upper digestive system diseases | 2023 | 20 (11.2 - 28.8) | 127 (105 - 149) | 108 (87.6 - 128) | 23.9 (14.3 - 33.5) | 145 (121 - 168) | 123 (102 - 145) |
| upper respiratory infections | 2023 | 2 (-0.772 - 4.77) | 1 (-0.96 - 2.96) | 2 (-0.772 - 4.77) | 2.13 (-0.731 - 4.99) | 1.91 (-0.798 - 4.62) | 2.7 (-0.519 - 5.93) |
| urinary diseases and male infertility | 2023 | 44 (31 - 57) | 302 (268 - 336) | 276 (243 - 309) | 63.2 (47.7 - 78.8) | 383 (344 - 421) | 352 (315 - 389) |
| uterine cancer | 2023 | 35 (23.4 - 46.6) | 121 (99.4 - 143) | 64 (48.3 - 79.7) | 48.5 (34.8 - 62.1) | 175 (149 - 201) | 96.1 (76.8 - 115) |
| varicella and herpes zoster | 2023 | 1 (-0.96 - 2.96) | 13 (5.93 - 20.1) | 15 (7.41 - 22.6) | 2.7 (-0.522 - 5.92) | 24.3 (14.6 - 33.9) | 30.8 (20 - 41.7) |
| vascular intestinal disorders | 2023 | 19 (10.5 - 27.5) | 99 (79.5 - 119) | 70 (53.6 - 86.4) | 23.5 (14 - 33) | 121 (99.6 - 143) | 89 (70.5 - 108) |
| whooping cough | 2023 |  |  | 1 (-0.96 - 2.96) | 0.000518 (-0.0441 - 0.0451) |  | 13 (-0.959 - 31) |
| acute glomerulonephritis | 1997 |  | 1 (-0.96 - 2.96) | 1 (-0.96 - 2.96) | 0.00206 (-0.087 - 0.0911) | 14 (-0.959 - 33) | 13 (-0.959 - 33) |
| acute hepatitis | 1997 |  |  |  | 0.0315 (-0.316 - 0.379) | 0.183 (-0.656 - 12) | 0.243 (-0.724 - 1.21) |
| adverse effects of medical treatment | 1997 | 3 (-0.395 - 6.39) | 18 (9.68 - 26.3) | 44 (31 - 57) | 3.39 (-0.219 - 7) | 21.4 (12.3 - 30.5) | 55.5 (40.9 - 70.1) |
| alcohol use disorders | 1997 | 20 (11.2 - 28.8) | 200 (172 - 228) | 286 (253 - 319) | 21.4 (12.3 - 30.5) | 212 (184 - 241) | 301 (267 - 335) |
| alzheimer's disease and other dementias | 1997 | 53 (38.7 - 67.3) | 327 (292 - 362) | 985 (923 - 1 050) | 53.2 (38.9 - 67.6) | 330 (294 - 365) | 996 (934 - 1 060) |
| animal contact | 1997 | 2 (-0.772 - 4.77) | 5 (0.617 - 9.38) | 6 (1.2 - 10.8) | 24 (-0.759 - 4.84) | 5.34 (0.812 - 9.87) | 6.52 (1.51 - 11.5) |
| aortic aneurysm | 1997 | 62 (46.6 - 77.4) | 297 (263 - 331) | 680 (629 - 731) | 66.3 (50.3 - 82.2) | 329 (293 - 364) | 763 (708 - 817) |
| appendicitis | 1997 | 2 (-0.772 - 4.77) | 4 (08 - 7.92) | 8 (2.46 - 13.5) | 28 (-0.745 - 4.91) | 4.99 (0.613 - 9.37) | 10.7 (4.26 - 17.1) |
| asthma | 1997 | 11 (4.5 - 17.5) | 41 (28.4 - 53.6) | 125 (103 - 147) | 11.2 (4.65 - 17.8) | 42.4 (29.7 - 55.2) | 128 (106 - 150) |
| atrial fibrillation and flutter | 1997 | 19 (10.5 - 27.5) | 138 (115 - 161) | 478 (435 - 521) | 19 (10.5 - 27.6) | 138 (115 - 161) | 478 (435 - 521) |
| bacterial skin diseases | 1997 | 1 (-0.96 - 2.96) | 9 (3.12 - 14.9) | 44 (31 - 57) | 1.56 (-0.889 - 4) | 13.9 (6.57 - 21.2) | 61.5 (46.1 - 76.9) |
| bladder cancer | 1997 | 31 (20.1 - 41.9) | 143 (120 - 166) | 288 (255 - 321) | 35.1 (23.5 - 46.7) | 165 (140 - 190) | 342 (305 - 378) |
| brain and central nervous system cancer | 1997 | 50 (36.1 - 63.9) | 235 (205 - 265) | 278 (245 - 311) | 52.2 (38 - 66.4) | 249 (218 - 280) | 301 (267 - 335) |
| breast cancer | 1997 | 105 (84.9 - 125) | 479 (436 - 522) | 657 (607 - 707) | 113 (92 - 134) | 532 (487 - 577) | 776 (722 - 831) |
| cardiomyopathy and myocarditis | 1997 | 8 (2.46 - 13.5) | 53 (38.7 - 67.3) | 64 (48.3 - 79.7) | 20.8 (11.8 - 29.7) | 169 (143 - 194) | 274 (241 - 306) |
| cervical cancer | 1997 | 4 (08 - 7.92) | 51 (37 - 65) | 96 (76.8 - 115) | 86 (2.5 - 13.6) | 82.1 (64.3 - 99.8) | 163 (138 - 188) |
| chronic kidney disease | 1997 | 8 (2.46 - 13.5) | 102 (82.2 - 122) | 237 (207 - 267) | 15.8 (81 - 23.6) | 162 (137 - 187) | 428 (388 - 469) |
| chronic obstructive pulmonary disease | 1997 | 55 (40.5 - 69.5) | 431 (390 - 472) | 1 160 (1 090 - 1 220) | 58.8 (43.8 - 73.8) | 471 (429 - 514) | 1 270 (1 200 - 1 340) |
| cirrhosis and other chronic liver diseases | 1997 | 18 (9.68 - 26.3) | 172 (146 - 198) | 250 (219 - 281) | 20.8 (11.8 - 29.7) | 200 (172 - 228) | 298 (264 - 332) |
| colon and rectum cancer | 1997 | 101 (81.3 - 121) | 646 (596 - 696) | 1 260 (1 190 - 1 330) | 121 (99.2 - 142) | 770 (716 - 824) | 1 550 (1 470 - 1 630) |
| congenital birth defects | 1997 | 41 (28.4 - 53.6) | 92 (73.2 - 111) | 63 (47.4 - 78.6) | 43.4 (30.5 - 56.3) | 102 (81.8 - 121) | 66.4 (50.5 - 82.4) |
| cystic echinococcosis | 1997 |  | 1 (-0.96 - 2.96) |  |  | 12 (-0.96 - 2.99) |  |
| decubitus ulcer | 1997 | 2 (-0.772 - 4.77) | 8 (2.46 - 13.5) | 20 (11.2 - 28.8) | 24 (-0.758 - 4.85) | 8.51 (2.79 - 14.2) | 21.8 (12.6 - 30.9) |
| diabetes mellitus | 1997 | 3 (-0.395 - 6.39) | 56 (41.3 - 70.7) | 159 (134 - 184) | 24.9 (15.1 - 34.7) | 260 (229 - 292) | 828 (772 - 885) |
| diarrheal diseases | 1997 | 1 (-0.96 - 2.96) | 4 (08 - 7.92) | 12 (5.21 - 18.8) | 1.27 (-0.939 - 3.48) | 5.89 (1.13 - 10.6) | 20.9 (12 - 29.9) |
| diverticular disease of intestines | 1997 | 2 (-0.772 - 4.77) | 19 (10.5 - 27.5) | 56 (41.3 - 70.7) | 2.29 (-0.677 - 5.25) | 21.2 (12.2 - 30.3) | 64 (48.3 - 79.6) |
| drowning | 1997 | 9 (3.12 - 14.9) | 40 (27.6 - 52.4) | 56 (41.3 - 70.7) | 10.2 (3.97 - 16.5) | 47.5 (34 - 61.1) | 63.4 (47.8 - 79.1) |
| drug use disorders | 1997 | 1 (-0.96 - 2.96) | 38 (25.9 - 50.1) | 36 (24.2 - 47.8) | 2.15 (-0.722 - 53) | 86.3 (68.1 - 105) | 89.7 (71.1 - 108) |
| eating disorders | 1997 |  | 1 (-0.96 - 2.96) |  |  | 1 (-0.96 - 2.96) |  |
| electrocution | 1997 |  | 6 (1.2 - 10.8) | 1 (-0.96 - 2.96) | 0.00398 (-0.12 - 0.128) | 6.13 (1.28 - 11) | 13 (-0.959 - 32) |
| encephalitis | 1997 | 2 (-0.772 - 4.77) | 4 (08 - 7.92) | 2 (-0.772 - 4.77) | 21 (-0.768 - 4.79) | 48 (0.119 - 83) | 2.38 (-0.644 - 5.4) |
| endocarditis | 1997 | 3 (-0.395 - 6.39) | 26 (16 - 36) | 70 (53.6 - 86.4) | 4.16 (0.161 - 8.15) | 33.5 (22.2 - 44.9) | 95.5 (76.4 - 115) |
| endocrine, metabolic, blood, and immune disorders | 1997 | 5 (0.617 - 9.38) | 49 (35.3 - 62.7) | 83 (65.1 - 101) | 5.95 (1.17 - 10.7) | 54 (39.6 - 68.4) | 96.1 (76.9 - 115) |
| environmental heat and cold exposure | 1997 | 3 (-0.395 - 6.39) | 7 (1.81 - 12.2) | 20 (11.2 - 28.8) | 3.33 (-0.245 - 6.91) | 9.13 (3.21 - 15) | 25.6 (15.7 - 35.6) |
| epilepsy | 1997 | 6 (1.2 - 10.8) | 33 (21.7 - 44.3) | 49 (35.3 - 62.7) | 6.8 (1.69 - 11.9) | 35.1 (23.5 - 46.7) | 52 (37.9 - 66.1) |
| esophageal cancer | 1997 | 11 (4.5 - 17.5) | 90 (71.4 - 109) | 223 (194 - 252) | 15.2 (7.54 - 22.8) | 115 (94.4 - 137) | 278 (245 - 310) |
| exposure to forces of nature | 1997 |  | 3 (-0.395 - 6.39) |  |  | 38 (-0.361 - 6.52) |  |
| exposure to mechanical forces | 1997 |  | 24 (14.4 - 33.6) | 24 (14.4 - 33.6) | 0.464 (-0.871 - 1.8) | 27.3 (17.1 - 37.6) | 31.1 (20.2 - 42.1) |
| eye cancer | 1997 |  | 2 (-0.772 - 4.77) | 1 (-0.96 - 2.96) | 0.0796 (-0.473 - 0.632) | 3.46 (-0.184 - 7.11) | 63 (1.22 - 10.8) |
| falls | 1997 | 24 (14.4 - 33.6) | 141 (118 - 164) | 246 (215 - 277) | 36 (24.2 - 47.7) | 236 (206 - 266) | 561 (515 - 608) |
| fire, heat, and hot substances | 1997 |  | 43 (30.1 - 55.9) | 40 (27.6 - 52.4) | 0.758 (-0.948 - 2.47) | 46.6 (33.2 - 60) | 46.6 (33.3 - 60) |
| food-borne trematodiases | 1997 |  |  |  | 2.66e-05 (-0.0101 - 0.0101) |  | 0.2 (-0.677 - 18) |
| foreign body | 1997 | 4 (08 - 7.92) | 27 (16.8 - 37.2) | 52 (37.9 - 66.1) | 4.59 (0.389 - 8.79) | 31.3 (20.3 - 42.2) | 62.8 (47.3 - 78.4) |
| gallbladder and biliary diseases | 1997 | 3 (-0.395 - 6.39) | 24 (14.4 - 33.6) | 94 (75 - 113) | 3.4 (-0.214 - 72) | 27.1 (16.9 - 37.4) | 104 (84.3 - 124) |
| gallbladder and biliary tract cancer | 1997 | 25 (15.2 - 34.8) | 150 (126 - 174) | 295 (261 - 329) | 27.9 (17.6 - 38.3) | 169 (143 - 194) | 342 (306 - 378) |
| gynecological diseases | 1997 | 1 (-0.96 - 2.96) | 2 (-0.772 - 4.77) | 2 (-0.772 - 4.77) | 11 (-0.96 - 2.98) | 25 (-0.755 - 4.86) | 2.12 (-0.733 - 4.98) |
| hemoglobinopathies and hemolytic anemias | 1997 | 1 (-0.96 - 2.96) | 8 (2.46 - 13.5) | 17 (8.92 - 25.1) | 15 (-0.959 - 35) | 10.5 (4.13 - 16.8) | 23 (13.6 - 32.4) |
| hiv/aids | 1997 | 5 (0.617 - 9.38) | 17 (8.92 - 25.1) | 19 (10.5 - 27.5) | 5.21 (0.733 - 9.68) | 20.5 (11.6 - 29.3) | 21.9 (12.7 - 31.1) |
| hodgkin lymphoma | 1997 | 3 (-0.395 - 6.39) | 13 (5.93 - 20.1) | 19 (10.5 - 27.5) | 3.14 (-0.334 - 6.61) | 14 (6.65 - 21.3) | 20.8 (11.9 - 29.7) |
| hypertensive heart disease | 1997 | 4 (08 - 7.92) | 37 (25.1 - 48.9) | 130 (108 - 152) | 5.28 (0.777 - 9.79) | 50 (36.1 - 63.9) | 177 (151 - 203) |
| inflammatory bowel disease | 1997 | 6 (1.2 - 10.8) | 23 (13.6 - 32.4) | 44 (31 - 57) | 6.66 (1.6 - 11.7) | 28.5 (18 - 38.9) | 59.8 (44.7 - 75) |
| inguinal, femoral, and abdominal hernia | 1997 | 3 (-0.395 - 6.39) | 14 (6.67 - 21.3) | 31 (20.1 - 41.9) | 3.16 (-0.324 - 6.65) | 15.1 (7.51 - 22.8) | 34.1 (22.6 - 45.5) |
| interpersonal violence | 1997 | 3 (-0.395 - 6.39) | 32 (20.9 - 43.1) | 47 (33.6 - 60.4) | 5.58 (0.95 - 10.2) | 50.9 (36.9 - 64.8) | 61.8 (46.4 - 77.2) |
| interstitial lung disease and pulmonary sarcoidosis | 1997 | 5 (0.617 - 9.38) | 69 (52.7 - 85.3) | 142 (119 - 165) | 5.39 (0.841 - 9.94) | 78 (60.7 - 95.3) | 176 (150 - 202) |
| invasive non-typhoidal salmonella (ints) | 1997 |  |  |  | 0.000244 (-0.0304 - 0.0309) | 0.00231 (-0.092 - 0.0966) | 0.00461 (-0.128 - 0.138) |
| ischemic heart disease | 1997 | 539 (493 - 585) | 4 140 (4 020 - 4 270) | 10 700 (10 500 - 10 900) | 616 (567 - 664) | 4 730 (4 590 - 4 860) | 12 500 (12 200 - 12 700) |
| kidney cancer | 1997 | 25 (15.2 - 34.8) | 222 (193 - 251) | 384 (346 - 422) | 29.6 (19 - 40.3) | 249 (218 - 280) | 441 (400 - 483) |
| larynx cancer | 1997 |  | 17 (8.92 - 25.1) | 31 (20.1 - 41.9) | 0.279 (-0.756 - 1.31) | 18.5 (10 - 26.9) | 34.5 (22.9 - 46) |
| leishmaniasis | 1997 |  |  |  | 2.66e-05 (-0101 - 0101) |  | 0.2 (-0.677 - 18) |
| leukemia | 1997 | 34 (22.6 - 45.4) | 145 (121 - 169) | 218 (189 - 247) | 48.3 (34.6 - 61.9) | 197 (169 - 224) | 377 (339 - 415) |
| lip and oral cavity cancer | 1997 | 6 (1.2 - 10.8) | 43 (30.1 - 55.9) | 72 (55.4 - 88.6) | 6.61 (1.57 - 11.6) | 47.4 (33.9 - 60.9) | 82 (64.3 - 99.8) |
| liver cancer | 1997 | 7 (1.81 - 12.2) | 54 (39.6 - 68.4) | 102 (82.2 - 122) | 7.86 (2.37 - 13.4) | 60.7 (45.4 - 76) | 122 (100 - 143) |
| lower respiratory infections | 1997 | 7 (1.81 - 12.2) | 40 (27.6 - 52.4) | 87 (68.7 - 105) | 73.9 (57.1 - 90.8) | 435 (394 - 476) | 1 460 (1 380 - 1 530) |
| malaria | 1997 | 1 (-0.96 - 2.96) |  |  | 1 (-0.96 - 2.96) |  |  |
| malignant bone tumors | 1997 | 2 (-0.772 - 4.77) | 9 (3.12 - 14.9) | 22 (12.8 - 31.2) | 2.14 (-0.729 - 5) | 9.95 (3.77 - 16.1) | 23.9 (14.3 - 33.5) |
| malignant skin melanoma | 1997 | 27 (16.8 - 37.2) | 134 (111 - 157) | 162 (137 - 187) | 28.5 (18.1 - 39) | 143 (120 - 167) | 179 (153 - 205) |
| maternal disorders | 1997 | 1 (-0.96 - 2.96) | 1 (-0.96 - 2.96) | 1 (-0.96 - 2.96) | 15 (-0.959 - 35) | 1.13 (-0.954 - 3.21) | 1.13 (-0.953 - 3.22) |
| meningitis | 1997 | 1 (-0.96 - 2.96) | 3 (-0.395 - 6.39) | 5 (0.617 - 9.38) | 27 (-0.75 - 4.89) | 8.42 (2.73 - 14.1) | 17.4 (9.26 - 25.6) |
| mesothelioma | 1997 | 3 (-0.395 - 6.39) | 46 (32.7 - 59.3) | 64 (48.3 - 79.7) | 3.57 (-0.133 - 7.27) | 49.3 (35.6 - 63.1) | 70.6 (54.1 - 87.1) |
| motor neuron disease | 1997 | 20 (11.2 - 28.8) | 77 (59.8 - 94.2) | 128 (106 - 150) | 20.3 (11.5 - 29.1) | 79.4 (61.9 - 96.8) | 131 (109 - 154) |
| multiple myeloma | 1997 | 23 (13.6 - 32.4) | 134 (111 - 157) | 295 (261 - 329) | 24.9 (15.2 - 34.7) | 152 (128 - 176) | 344 (308 - 380) |
| multiple sclerosis | 1997 | 6 (1.2 - 10.8) | 42 (29.3 - 54.7) | 53 (38.7 - 67.3) | 6 (1.2 - 10.8) | 42 (29.3 - 54.7) | 53 (38.7 - 67.3) |
| nasopharynx cancer | 1997 |  | 11 (4.5 - 17.5) | 9 (3.12 - 14.9) | 0.124 (-0.566 - 0.813) | 12.1 (5.26 - 18.9) | 11.9 (5.11 - 18.6) |
| neonatal disorders | 1997 | 38 (25.9 - 50.1) | 74 (57.1 - 90.9) | 7 (1.81 - 12.2) | 39.9 (27.5 - 52.3) | 77.1 (59.9 - 94.3) | 72 (1.83 - 12.2) |
| neuroblastoma and other peripheral nervous cell tumors | 1997 | 1 (-0.96 - 2.96) | 5 (0.617 - 9.38) | 5 (0.617 - 9.38) | 19 (-0.956 - 3.14) | 9.25 (3.29 - 15.2) | 7.2 (1.94 - 12.5) |
| non-hodgkin lymphoma | 1997 | 6 (1.2 - 10.8) | 32 (20.9 - 43.1) | 56 (41.3 - 70.7) | 51.7 (37.6 - 65.8) | 250 (219 - 281) | 458 (416 - 500) |
| non-melanoma skin cancer | 1997 | 1 (-0.96 - 2.96) | 19 (10.5 - 27.5) | 17 (8.92 - 25.1) | 1.23 (-0.944 - 3.39) | 20.6 (11.7 - 29.5) | 21.1 (12.1 - 30.1) |
| non-rheumatic valvular heart disease | 1997 | 9 (3.12 - 14.9) | 97 (77.7 - 116) | 329 (293 - 365) | 11.5 (4.82 - 18.1) | 119 (97.8 - 141) | 407 (368 - 447) |
| other cardiovascular and circulatory diseases | 1997 | 15 (7.41 - 22.6) | 88 (69.6 - 106) | 208 (180 - 236) | 18.6 (10.1 - 27) | 124 (102 - 146) | 306 (272 - 340) |
| other chronic respiratory diseases | 1997 | 2 (-0.772 - 4.77) | 8 (2.46 - 13.5) | 13 (5.93 - 20.1) | 25 (-0.755 - 4.86) | 10.1 (3.84 - 16.3) | 20.3 (11.5 - 29.1) |
| other digestive diseases | 1997 | 2 (-0.772 - 4.77) | 17 (8.92 - 25.1) | 41 (28.4 - 53.6) | 3.23 (-0.29 - 6.76) | 27.3 (17 - 37.5) | 67.7 (51.6 - 83.9) |
| other intestinal infectious diseases | 1997 | 2 (-0.772 - 4.77) | 3 (-0.395 - 6.39) | 9 (3.12 - 14.9) | 27 (-0.749 - 4.9) | 4.83 (0.52 - 9.13) | 15.7 (7.93 - 23.5) |
| other malignant neoplasms | 1997 | 26 (16 - 36) | 93 (74.1 - 112) | 175 (149 - 201) | 28.7 (18.2 - 39.1) | 120 (98.6 - 142) | 227 (197 - 257) |
| other musculoskeletal disorders | 1997 | 6 (1.2 - 10.8) | 47 (33.6 - 60.4) | 70 (53.6 - 86.4) | 6.23 (1.34 - 11.1) | 48.9 (35.2 - 62.6) | 75.1 (58.1 - 92.1) |
| other neglected tropical diseases | 1997 |  |  |  | 0.00029 (-0.0331 - 0.0337) | 0.541 (-0.9 - 1.98) | 0.935 (-0.96 - 2.83) |
| other neoplasms | 1997 | 12 (5.21 - 18.8) | 84 (66 - 102) | 189 (162 - 216) | 12.3 (5.42 - 19.2) | 86.2 (68 - 104) | 195 (168 - 223) |
| other neurological disorders | 1997 | 7 (1.81 - 12.2) | 51 (37 - 65) | 93 (74.1 - 112) | 7.62 (2.21 - 13) | 54.2 (39.7 - 68.6) | 97.1 (77.8 - 116) |
| other nutritional deficiencies | 1997 |  | 1 (-0.96 - 2.96) | 1 (-0.96 - 2.96) | 0.0101 (-0.187 - 0.208) | 1.69 (-0.857 - 4.24) | 3.76 (-0384 - 7.57) |
| other pharynx cancer | 1997 | 5 (0.617 - 9.38) | 18 (9.68 - 26.3) | 32 (20.9 - 43.1) | 5.22 (0.745 - 9.7) | 19.6 (10.9 - 28.2) | 35.6 (23.9 - 47.3) |
| other skin and subcutaneous diseases | 1997 |  | 1 (-0.96 - 2.96) | 6 (1.2 - 10.8) | 0.000312 (-0.0343 - 0.0349) | 1 (-0.96 - 2.97) | 61 (1.21 - 10.8) |
| other transport injuries | 1997 | 7 (1.81 - 12.2) | 49 (35.3 - 62.7) | 37 (25.1 - 48.9) | 7.67 (2.24 - 13.1) | 52.3 (38.2 - 66.5) | 40.9 (28.4 - 53.5) |
| other unintentional injuries | 1997 |  | 2 (-0.772 - 4.77) | 1 (-0.96 - 2.96) | 0.357 (-0.814 - 1.53) | 4.86 (0.537 - 9.17) | 62 (1.21 - 10.8) |
| other unspecified infectious diseases | 1997 | 1 (-0.96 - 2.96) | 2 (-0.772 - 4.77) | 8 (2.46 - 13.5) | 1.8 (-0.828 - 4.44) | 6.76 (1.66 - 11.9) | 23.8 (14.2 - 33.3) |
| otitis media | 1997 |  |  |  | 3.53e-05 (-0116 - 0117) | 0.000697 (-0.051 - 0.0524) | 0.00209 (-0.0876 - 0.0918) |
| ovarian cancer | 1997 | 35 (23.4 - 46.6) | 190 (163 - 217) | 361 (324 - 398) | 39.8 (27.5 - 52.2) | 222 (192 - 251) | 437 (396 - 478) |
| pancreatic cancer | 1997 | 64 (48.3 - 79.7) | 402 (363 - 441) | 724 (671 - 777) | 74.4 (57.5 - 91.3) | 465 (422 - 507) | 869 (811 - 926) |
| pancreatitis | 1997 | 2 (-0.772 - 4.77) | 30 (19.3 - 40.7) | 60 (44.8 - 75.2) | 2.37 (-0.648 - 5.39) | 33.3 (22 - 44.6) | 67 (50.9 - 83) |
| paralytic ileus and intestinal obstruction | 1997 | 7 (1.81 - 12.2) | 31 (20.1 - 41.9) | 109 (88.5 - 129) | 8.17 (2.57 - 13.8) | 39.9 (27.5 - 52.2) | 135 (112 - 158) |
| parkinson's disease | 1997 | 16 (8.16 - 23.8) | 87 (68.7 - 105) | 167 (142 - 192) | 16 (8.16 - 23.8) | 87 (68.7 - 105) | 167 (142 - 192) |
| peripheral artery disease | 1997 | 5 (0.617 - 9.38) | 26 (16 - 36) | 109 (88.5 - 129) | 5.71 (12 - 10.4) | 32.3 (21.1 - 43.4) | 130 (108 - 153) |
| pneumoconiosis | 1997 |  |  | 12 (5.21 - 18.8) | 0.0138 (-0.217 - 0.244) | 0.243 (-0.723 - 1.21) | 14.7 (7.22 - 22.3) |
| poisonings | 1997 |  | 2 (-0.772 - 4.77) | 4 (08 - 7.92) | 0.879 (-0.959 - 2.72) | 14.1 (6.76 - 21.5) | 12.5 (5.54 - 19.4) |
| police conflict and executions | 1997 |  | 1 (-0.96 - 2.96) |  | 0.000111 (-0.0205 - 0.0207) | 12 (-0.959 - 3) | 0.0173 (-0.24 - 0.275) |
| primary pulmonary arterial hypertension | 1997 | 2 (-0.772 - 4.77) | 9 (3.12 - 14.9) | 11 (4.5 - 17.5) | 26 (-0.752 - 4.88) | 10.2 (3.91 - 16.4) | 13.2 (6.1 - 20.4) |
| prostate cancer | 1997 | 132 (109 - 155) | 616 (567 - 665) | 1 220 (1 150 - 1 290) | 148 (124 - 172) | 707 (655 - 759) | 1 420 (1 350 - 1 490) |
| protein-energy malnutrition | 1997 |  | 5 (0.617 - 9.38) | 24 (14.4 - 33.6) | 0.102 (-0.525 - 0.729) | 6.55 (1.53 - 11.6) | 28.8 (18.3 - 39.3) |
| rheumatic heart disease | 1997 | 4 (08 - 7.92) | 33 (21.7 - 44.3) | 97 (77.7 - 116) | 4.6 (0.394 - 8.8) | 39.1 (26.8 - 51.3) | 117 (96 - 138) |
| rheumatoid arthritis | 1997 | 4 (08 - 7.92) | 23 (13.6 - 32.4) | 112 (91.3 - 133) | 4.13 (0.148 - 8.12) | 24.7 (15 - 34.5) | 117 (95.7 - 138) |
| road injuries | 1997 | 22 (12.8 - 31.2) | 242 (212 - 272) | 155 (131 - 179) | 26.5 (16.4 - 36.6) | 275 (243 - 308) | 195 (168 - 223) |
| self-harm | 1997 | 78 (60.7 - 95.3) | 486 (443 - 529) | 406 (367 - 445) | 105 (85.3 - 126) | 665 (614 - 715) | 551 (505 - 597) |
| sexually transmitted infections excluding hiv | 1997 |  |  |  | 1 (-0.96 - 2.96) | 0.00746 (-0.162 - 0.177) | 4.17 (0.168 - 8.17) |
| soft tissue and other extraosseous sarcomas | 1997 | 9 (3.12 - 14.9) | 48 (34.4 - 61.6) | 63 (47.4 - 78.6) | 9.47 (3.44 - 15.5) | 50.9 (36.9 - 64.9) | 71.2 (54.7 - 87.8) |
| stomach cancer | 1997 | 34 (22.6 - 45.4) | 251 (220 - 282) | 584 (537 - 631) | 43.9 (30.9 - 56.9) | 319 (284 - 353) | 738 (685 - 791) |
| stroke | 1997 | 139 (116 - 162) | 1 050 (989 - 1 120) | 2 670 (2 570 - 2 770) | 245 (214 - 275) | 1 850 (1 760 - 1 930) | 5 270 (5 120 - 5 410) |
| testicular cancer | 1997 |  | 7 (1.81 - 12.2) | 7 (1.81 - 12.2) | 0.189 (-0.663 - 14) | 8.42 (2.73 - 14.1) | 8.2 (2.59 - 13.8) |
| thyroid cancer | 1997 | 5 (0.617 - 9.38) | 15 (7.41 - 22.6) | 39 (26.8 - 51.2) | 5.22 (0.739 - 9.69) | 17.2 (94 - 25.3) | 43.1 (30.2 - 55.9) |
| tracheal, bronchus, and lung cancer | 1997 | 137 (114 - 160) | 962 (901 - 1 020) | 1 670 (1 590 - 1 750) | 151 (127 - 175) | 1 040 (981 - 1 110) | 1 850 (1 760 - 1 930) |
| tuberculosis | 1997 | 2 (-0.772 - 4.77) | 26 (16 - 36) | 57 (42.2 - 71.8) | 3.41 (-0.212 - 72) | 28.5 (18 - 38.9) | 68 (51.8 - 84.2) |
| upper digestive system diseases | 1997 | 4 (08 - 7.92) | 77 (59.8 - 94.2) | 213 (184 - 242) | 4.65 (0.424 - 8.88) | 81.9 (64.1 - 99.6) | 227 (197 - 256) |
| upper respiratory infections | 1997 |  | 3 (-0.395 - 6.39) |  | 0.00676 (-0.154 - 0.168) | 3.24 (-0.287 - 6.77) | 0.523 (-0.895 - 1.94) |
| urinary diseases and male infertility | 1997 | 5 (0.617 - 9.38) | 65 (49.2 - 80.8) | 232 (202 - 262) | 6.35 (1.41 - 11.3) | 77.9 (60.6 - 95.2) | 279 (246 - 312) |
| uterine cancer | 1997 | 7 (1.81 - 12.2) | 34 (22.6 - 45.4) | 77 (59.8 - 94.2) | 10 (3.83 - 16.3) | 60.2 (45 - 75.5) | 139 (116 - 162) |
| varicella and herpes zoster | 1997 |  | 2 (-0.772 - 4.77) | 2 (-0.772 - 4.77) | 0.0457 (-0.373 - 0.465) | 2.86 (-0.453 - 6.18) | 3.76 (-0388 - 7.57) |

Table S12: Garbage level in Sweden, all ages, by region, 1997 to 2023

| **Table S12:** Garbage level in Sweden, all ages, by region, 1997 to 2023 | | | | |
| --- | --- | --- | --- | --- |
| Region | Year | GC deaths | Total deaths | Fraction of deaths coded to GCs |
| Blekinge län | 1997 | 540 | 1819 | 0.30 |
| Blekinge län | 1998 | 488 | 1750 | 0.28 |
| Blekinge län | 1999 | 555 | 1791 | 0.31 |
| Blekinge län | 2000 | 545 | 1729 | 0.32 |
| Blekinge län | 2001 | 507 | 1679 | 0.30 |
| Blekinge län | 2002 | 546 | 1748 | 0.31 |
| Blekinge län | 2003 | 531 | 1737 | 0.31 |
| Blekinge län | 2004 | 481 | 1603 | 0.30 |
| Blekinge län | 2005 | 471 | 1679 | 0.28 |
| Blekinge län | 2006 | 487 | 1716 | 0.28 |
| Blekinge län | 2007 | 500 | 1700 | 0.29 |
| Blekinge län | 2008 | 524 | 1740 | 0.30 |
| Blekinge län | 2009 | 533 | 1799 | 0.30 |
| Blekinge län | 2010 | 486 | 1677 | 0.29 |
| Blekinge län | 2011 | 526 | 1711 | 0.31 |
| Blekinge län | 2012 | 515 | 1760 | 0.29 |
| Blekinge län | 2013 | 453 | 1669 | 0.27 |
| Blekinge län | 2014 | 444 | 1661 | 0.27 |
| Blekinge län | 2015 | 455 | 1687 | 0.27 |
| Blekinge län | 2016 | 488 | 1767 | 0.28 |
| Blekinge län | 2017 | 450 | 1778 | 0.25 |
| Blekinge län | 2018 | 433 | 1722 | 0.25 |
| Blekinge län | 2019 | 408 | 1666 | 0.24 |
| Blekinge län | 2020 | 459 | 1793 | 0.26 |
| Blekinge län | 2021 | 459 | 1790 | 0.26 |
| Blekinge län | 2022 | 488 | 1830 | 0.27 |
| Blekinge län | 2023 | 462 | 1821 | 0.25 |
| Dalarnas län | 1997 | 1049 | 3582 | 0.29 |
| Dalarnas län | 1998 | 1026 | 3486 | 0.29 |
| Dalarnas län | 1999 | 1021 | 3426 | 0.30 |
| Dalarnas län | 2000 | 1009 | 3406 | 0.30 |
| Dalarnas län | 2001 | 957 | 3443 | 0.28 |
| Dalarnas län | 2002 | 965 | 3568 | 0.27 |
| Dalarnas län | 2003 | 909 | 3421 | 0.27 |
| Dalarnas län | 2004 | 886 | 3319 | 0.27 |
| Dalarnas län | 2005 | 925 | 3337 | 0.28 |
| Dalarnas län | 2006 | 855 | 3229 | 0.26 |
| Dalarnas län | 2007 | 974 | 3234 | 0.30 |
| Dalarnas län | 2008 | 905 | 3178 | 0.28 |
| Dalarnas län | 2009 | 861 | 3253 | 0.26 |
| Dalarnas län | 2010 | 910 | 3165 | 0.29 |
| Dalarnas län | 2011 | 857 | 3085 | 0.28 |
| Dalarnas län | 2012 | 1016 | 3326 | 0.31 |
| Dalarnas län | 2013 | 853 | 3148 | 0.27 |
| Dalarnas län | 2014 | 763 | 3047 | 0.25 |
| Dalarnas län | 2015 | 835 | 3178 | 0.26 |
| Dalarnas län | 2016 | 832 | 3157 | 0.26 |
| Dalarnas län | 2017 | 785 | 3093 | 0.25 |
| Dalarnas län | 2018 | 751 | 3121 | 0.24 |
| Dalarnas län | 2019 | 760 | 3034 | 0.25 |
| Dalarnas län | 2020 | 822 | 3310 | 0.25 |
| Dalarnas län | 2021 | 742 | 3056 | 0.24 |
| Dalarnas län | 2022 | 790 | 3091 | 0.26 |
| Dalarnas län | 2023 | 804 | 3171 | 0.25 |
| Gävleborgs län | 1997 | 933 | 3396 | 0.27 |
| Gävleborgs län | 1998 | 1136 | 3623 | 0.31 |
| Gävleborgs län | 1999 | 1072 | 3557 | 0.30 |
| Gävleborgs län | 2000 | 1111 | 3566 | 0.31 |
| Gävleborgs län | 2001 | 971 | 3370 | 0.29 |
| Gävleborgs län | 2002 | 1070 | 3630 | 0.29 |
| Gävleborgs län | 2003 | 1026 | 3560 | 0.29 |
| Gävleborgs län | 2004 | 964 | 3364 | 0.29 |
| Gävleborgs län | 2005 | 1036 | 3483 | 0.30 |
| Gävleborgs län | 2006 | 993 | 3419 | 0.29 |
| Gävleborgs län | 2007 | 1047 | 3431 | 0.31 |
| Gävleborgs län | 2008 | 994 | 3389 | 0.29 |
| Gävleborgs län | 2009 | 980 | 3343 | 0.29 |
| Gävleborgs län | 2010 | 983 | 3264 | 0.30 |
| Gävleborgs län | 2011 | 993 | 3267 | 0.30 |
| Gävleborgs län | 2012 | 993 | 3334 | 0.30 |
| Gävleborgs län | 2013 | 888 | 3201 | 0.28 |
| Gävleborgs län | 2014 | 891 | 3221 | 0.28 |
| Gävleborgs län | 2015 | 1026 | 3374 | 0.30 |
| Gävleborgs län | 2016 | 891 | 3156 | 0.28 |
| Gävleborgs län | 2017 | 873 | 3267 | 0.27 |
| Gävleborgs län | 2018 | 822 | 3084 | 0.27 |
| Gävleborgs län | 2019 | 815 | 3110 | 0.26 |
| Gävleborgs län | 2020 | 768 | 3413 | 0.23 |
| Gävleborgs län | 2021 | 819 | 3272 | 0.25 |
| Gävleborgs län | 2022 | 821 | 3384 | 0.24 |
| Gävleborgs län | 2023 | 770 | 3218 | 0.24 |
| Gotlands län | 1997 | 183 | 631 | 0.29 |
| Gotlands län | 1998 | 178 | 645 | 0.28 |
| Gotlands län | 1999 | 157 | 640 | 0.25 |
| Gotlands län | 2000 | 207 | 663 | 0.31 |
| Gotlands län | 2001 | 218 | 652 | 0.33 |
| Gotlands län | 2002 | 196 | 655 | 0.30 |
| Gotlands län | 2003 | 148 | 600 | 0.25 |
| Gotlands län | 2004 | 175 | 658 | 0.27 |
| Gotlands län | 2005 | 173 | 621 | 0.28 |
| Gotlands län | 2006 | 174 | 627 | 0.28 |
| Gotlands län | 2007 | 199 | 579 | 0.34 |
| Gotlands län | 2008 | 191 | 615 | 0.31 |
| Gotlands län | 2009 | 190 | 593 | 0.32 |
| Gotlands län | 2010 | 184 | 633 | 0.29 |
| Gotlands län | 2011 | 146 | 610 | 0.24 |
| Gotlands län | 2012 | 178 | 630 | 0.28 |
| Gotlands län | 2013 | 165 | 660 | 0.25 |
| Gotlands län | 2014 | 159 | 555 | 0.29 |
| Gotlands län | 2015 | 164 | 629 | 0.26 |
| Gotlands län | 2016 | 157 | 640 | 0.25 |
| Gotlands län | 2017 | 148 | 621 | 0.24 |
| Gotlands län | 2018 | 172 | 608 | 0.28 |
| Gotlands län | 2019 | 142 | 625 | 0.23 |
| Gotlands län | 2020 | 129 | 654 | 0.20 |
| Gotlands län | 2021 | 133 | 632 | 0.21 |
| Gotlands län | 2022 | 157 | 626 | 0.25 |
| Gotlands län | 2023 | 153 | 666 | 0.23 |
| Hallands län | 1997 | 831 | 2698 | 0.31 |
| Hallands län | 1998 | 767 | 2623 | 0.29 |
| Hallands län | 1999 | 797 | 2735 | 0.29 |
| Hallands län | 2000 | 834 | 2757 | 0.30 |
| Hallands län | 2001 | 780 | 2661 | 0.29 |
| Hallands län | 2002 | 843 | 2782 | 0.30 |
| Hallands län | 2003 | 798 | 2707 | 0.29 |
| Hallands län | 2004 | 710 | 2628 | 0.27 |
| Hallands län | 2005 | 736 | 2754 | 0.27 |
| Hallands län | 2006 | 770 | 2706 | 0.28 |
| Hallands län | 2007 | 789 | 2782 | 0.28 |
| Hallands län | 2008 | 787 | 2711 | 0.29 |
| Hallands län | 2009 | 847 | 2771 | 0.31 |
| Hallands län | 2010 | 843 | 2784 | 0.30 |
| Hallands län | 2011 | 848 | 2857 | 0.30 |
| Hallands län | 2012 | 811 | 2865 | 0.28 |
| Hallands län | 2013 | 840 | 2849 | 0.29 |
| Hallands län | 2014 | 776 | 2890 | 0.27 |
| Hallands län | 2015 | 765 | 2799 | 0.27 |
| Hallands län | 2016 | 822 | 2935 | 0.28 |
| Hallands län | 2017 | 818 | 3026 | 0.27 |
| Hallands län | 2018 | 750 | 2952 | 0.25 |
| Hallands län | 2019 | 671 | 2811 | 0.24 |
| Hallands län | 2020 | 729 | 3061 | 0.24 |
| Hallands län | 2021 | 695 | 2988 | 0.23 |
| Hallands län | 2022 | 794 | 3229 | 0.25 |
| Hallands län | 2023 | 846 | 3204 | 0.26 |
| Jämtlands län | 1997 | 562 | 1703 | 0.33 |
| Jämtlands län | 1998 | 523 | 1714 | 0.31 |
| Jämtlands län | 1999 | 525 | 1745 | 0.30 |
| Jämtlands län | 2000 | 515 | 1644 | 0.31 |
| Jämtlands län | 2001 | 522 | 1682 | 0.31 |
| Jämtlands län | 2002 | 499 | 1589 | 0.31 |
| Jämtlands län | 2003 | 468 | 1608 | 0.29 |
| Jämtlands län | 2004 | 484 | 1656 | 0.29 |
| Jämtlands län | 2005 | 468 | 1607 | 0.29 |
| Jämtlands län | 2006 | 447 | 1584 | 0.28 |
| Jämtlands län | 2007 | 487 | 1566 | 0.31 |
| Jämtlands län | 2008 | 423 | 1477 | 0.29 |
| Jämtlands län | 2009 | 468 | 1518 | 0.31 |
| Jämtlands län | 2010 | 453 | 1478 | 0.31 |
| Jämtlands län | 2011 | 455 | 1534 | 0.30 |
| Jämtlands län | 2012 | 454 | 1460 | 0.31 |
| Jämtlands län | 2013 | 495 | 1515 | 0.33 |
| Jämtlands län | 2014 | 399 | 1497 | 0.27 |
| Jämtlands län | 2015 | 443 | 1503 | 0.29 |
| Jämtlands län | 2016 | 416 | 1427 | 0.29 |
| Jämtlands län | 2017 | 431 | 1469 | 0.29 |
| Jämtlands län | 2018 | 391 | 1435 | 0.27 |
| Jämtlands län | 2019 | 375 | 1400 | 0.27 |
| Jämtlands län | 2020 | 355 | 1528 | 0.23 |
| Jämtlands län | 2021 | 350 | 1396 | 0.25 |
| Jämtlands län | 2022 | 390 | 1445 | 0.27 |
| Jämtlands län | 2023 | 369 | 1450 | 0.25 |
| Jönköpings län | 1997 | 981 | 3363 | 0.29 |
| Jönköpings län | 1998 | 1015 | 3640 | 0.28 |
| Jönköpings län | 1999 | 1076 | 3640 | 0.30 |
| Jönköpings län | 2000 | 1045 | 3633 | 0.29 |
| Jönköpings län | 2001 | 1120 | 3667 | 0.31 |
| Jönköpings län | 2002 | 1058 | 3620 | 0.29 |
| Jönköpings län | 2003 | 1050 | 3518 | 0.30 |
| Jönköpings län | 2004 | 980 | 3430 | 0.29 |
| Jönköpings län | 2005 | 934 | 3416 | 0.27 |
| Jönköpings län | 2006 | 978 | 3607 | 0.27 |
| Jönköpings län | 2007 | 975 | 3495 | 0.28 |
| Jönköpings län | 2008 | 987 | 3407 | 0.29 |
| Jönköpings län | 2009 | 906 | 3329 | 0.27 |
| Jönköpings län | 2010 | 952 | 3489 | 0.27 |
| Jönköpings län | 2011 | 949 | 3490 | 0.27 |
| Jönköpings län | 2012 | 990 | 3467 | 0.29 |
| Jönköpings län | 2013 | 921 | 3345 | 0.28 |
| Jönköpings län | 2014 | 869 | 3355 | 0.26 |
| Jönköpings län | 2015 | 847 | 3395 | 0.25 |
| Jönköpings län | 2016 | 841 | 3248 | 0.26 |
| Jönköpings län | 2017 | 874 | 3502 | 0.25 |
| Jönköpings län | 2018 | 877 | 3434 | 0.26 |
| Jönköpings län | 2019 | 808 | 3182 | 0.25 |
| Jönköpings län | 2020 | 768 | 3681 | 0.21 |
| Jönköpings län | 2021 | 699 | 3368 | 0.21 |
| Jönköpings län | 2022 | 844 | 3394 | 0.25 |
| Jönköpings län | 2023 | 858 | 3538 | 0.24 |
| Kalmar län | 1997 | 867 | 2911 | 0.30 |
| Kalmar län | 1998 | 842 | 2941 | 0.29 |
| Kalmar län | 1999 | 811 | 2939 | 0.28 |
| Kalmar län | 2000 | 839 | 2881 | 0.29 |
| Kalmar län | 2001 | 868 | 2953 | 0.29 |
| Kalmar län | 2002 | 842 | 3009 | 0.28 |
| Kalmar län | 2003 | 879 | 2876 | 0.31 |
| Kalmar län | 2004 | 842 | 2861 | 0.29 |
| Kalmar län | 2005 | 806 | 2870 | 0.28 |
| Kalmar län | 2006 | 788 | 2859 | 0.28 |
| Kalmar län | 2007 | 809 | 2835 | 0.29 |
| Kalmar län | 2008 | 814 | 2826 | 0.29 |
| Kalmar län | 2009 | 814 | 2795 | 0.29 |
| Kalmar län | 2010 | 759 | 2704 | 0.28 |
| Kalmar län | 2011 | 785 | 2779 | 0.28 |
| Kalmar län | 2012 | 731 | 2835 | 0.26 |
| Kalmar län | 2013 | 715 | 2705 | 0.26 |
| Kalmar län | 2014 | 660 | 2651 | 0.25 |
| Kalmar län | 2015 | 706 | 2775 | 0.25 |
| Kalmar län | 2016 | 678 | 2778 | 0.24 |
| Kalmar län | 2017 | 643 | 2768 | 0.23 |
| Kalmar län | 2018 | 683 | 2734 | 0.25 |
| Kalmar län | 2019 | 609 | 2518 | 0.24 |
| Kalmar län | 2020 | 633 | 2818 | 0.22 |
| Kalmar län | 2021 | 582 | 2635 | 0.22 |
| Kalmar län | 2022 | 682 | 2813 | 0.24 |
| Kalmar län | 2023 | 659 | 2900 | 0.23 |
| Kronobergs län | 1997 | 505 | 1842 | 0.27 |
| Kronobergs län | 1998 | 520 | 1922 | 0.27 |
| Kronobergs län | 1999 | 506 | 1918 | 0.26 |
| Kronobergs län | 2000 | 593 | 1963 | 0.30 |
| Kronobergs län | 2001 | 579 | 1991 | 0.29 |
| Kronobergs län | 2002 | 529 | 1864 | 0.28 |
| Kronobergs län | 2003 | 526 | 1918 | 0.27 |
| Kronobergs län | 2004 | 462 | 1797 | 0.26 |
| Kronobergs län | 2005 | 536 | 1913 | 0.28 |
| Kronobergs län | 2006 | 518 | 1873 | 0.28 |
| Kronobergs län | 2007 | 547 | 1853 | 0.30 |
| Kronobergs län | 2008 | 512 | 1857 | 0.28 |
| Kronobergs län | 2009 | 486 | 1821 | 0.27 |
| Kronobergs län | 2010 | 504 | 1824 | 0.28 |
| Kronobergs län | 2011 | 443 | 1759 | 0.25 |
| Kronobergs län | 2012 | 510 | 1868 | 0.27 |
| Kronobergs län | 2013 | 455 | 1894 | 0.24 |
| Kronobergs län | 2014 | 450 | 1747 | 0.26 |
| Kronobergs län | 2015 | 433 | 1848 | 0.23 |
| Kronobergs län | 2016 | 459 | 1782 | 0.26 |
| Kronobergs län | 2017 | 508 | 1923 | 0.26 |
| Kronobergs län | 2018 | 484 | 1921 | 0.25 |
| Kronobergs län | 2019 | 415 | 1819 | 0.23 |
| Kronobergs län | 2020 | 427 | 2094 | 0.20 |
| Kronobergs län | 2021 | 404 | 1879 | 0.22 |
| Kronobergs län | 2022 | 406 | 1902 | 0.21 |
| Kronobergs län | 2023 | 467 | 1852 | 0.25 |
| Norrbottens län | 1997 | 700 | 2693 | 0.26 |
| Norrbottens län | 1998 | 749 | 2751 | 0.27 |
| Norrbottens län | 1999 | 827 | 2890 | 0.29 |
| Norrbottens län | 2000 | 759 | 2780 | 0.27 |
| Norrbottens län | 2001 | 729 | 2730 | 0.27 |
| Norrbottens län | 2002 | 742 | 2857 | 0.26 |
| Norrbottens län | 2003 | 792 | 2833 | 0.28 |
| Norrbottens län | 2004 | 756 | 2814 | 0.27 |
| Norrbottens län | 2005 | 736 | 2805 | 0.26 |
| Norrbottens län | 2006 | 743 | 2734 | 0.27 |
| Norrbottens län | 2007 | 740 | 2790 | 0.27 |
| Norrbottens län | 2008 | 741 | 2865 | 0.26 |
| Norrbottens län | 2009 | 687 | 2646 | 0.26 |
| Norrbottens län | 2010 | 685 | 2687 | 0.25 |
| Norrbottens län | 2011 | 733 | 2802 | 0.26 |
| Norrbottens län | 2012 | 728 | 2854 | 0.26 |
| Norrbottens län | 2013 | 659 | 2761 | 0.24 |
| Norrbottens län | 2014 | 733 | 2819 | 0.26 |
| Norrbottens län | 2015 | 732 | 2822 | 0.26 |
| Norrbottens län | 2016 | 697 | 2815 | 0.25 |
| Norrbottens län | 2017 | 737 | 2861 | 0.26 |
| Norrbottens län | 2018 | 689 | 2844 | 0.24 |
| Norrbottens län | 2019 | 666 | 2838 | 0.23 |
| Norrbottens län | 2020 | 692 | 3019 | 0.23 |
| Norrbottens län | 2021 | 644 | 2897 | 0.22 |
| Norrbottens län | 2022 | 726 | 2979 | 0.24 |
| Norrbottens län | 2023 | 724 | 3002 | 0.24 |
| Örebro län | 1997 | 860 | 3262 | 0.26 |
| Örebro län | 1998 | 860 | 3239 | 0.27 |
| Örebro län | 1999 | 962 | 3326 | 0.29 |
| Örebro län | 2000 | 880 | 3260 | 0.27 |
| Örebro län | 2001 | 877 | 3246 | 0.27 |
| Örebro län | 2002 | 829 | 3353 | 0.25 |
| Örebro län | 2003 | 870 | 3210 | 0.27 |
| Örebro län | 2004 | 763 | 3183 | 0.24 |
| Örebro län | 2005 | 738 | 3102 | 0.24 |
| Örebro län | 2006 | 739 | 3050 | 0.24 |
| Örebro län | 2007 | 710 | 3002 | 0.24 |
| Örebro län | 2008 | 790 | 3026 | 0.26 |
| Örebro län | 2009 | 797 | 3183 | 0.25 |
| Örebro län | 2010 | 742 | 2929 | 0.25 |
| Örebro län | 2011 | 782 | 3025 | 0.26 |
| Örebro län | 2012 | 794 | 3005 | 0.26 |
| Örebro län | 2013 | 765 | 3095 | 0.25 |
| Örebro län | 2014 | 697 | 2819 | 0.25 |
| Örebro län | 2015 | 736 | 2941 | 0.25 |
| Örebro län | 2016 | 721 | 3002 | 0.24 |
| Örebro län | 2017 | 686 | 2916 | 0.24 |
| Örebro län | 2018 | 716 | 2957 | 0.24 |
| Örebro län | 2019 | 653 | 2843 | 0.23 |
| Örebro län | 2020 | 631 | 3027 | 0.21 |
| Örebro län | 2021 | 655 | 2873 | 0.23 |
| Örebro län | 2022 | 740 | 2988 | 0.25 |
| Örebro län | 2023 | 700 | 3117 | 0.22 |
| Östergötlands län | 1997 | 1323 | 4412 | 0.30 |
| Östergötlands län | 1998 | 1251 | 4256 | 0.29 |
| Östergötlands län | 1999 | 1297 | 4490 | 0.29 |
| Östergötlands län | 2000 | 1303 | 4401 | 0.30 |
| Östergötlands län | 2001 | 1255 | 4391 | 0.29 |
| Östergötlands län | 2002 | 1300 | 4473 | 0.29 |
| Östergötlands län | 2003 | 1252 | 4401 | 0.28 |
| Östergötlands län | 2004 | 1222 | 4294 | 0.28 |
| Östergötlands län | 2005 | 1233 | 4377 | 0.28 |
| Östergötlands län | 2006 | 1096 | 4220 | 0.26 |
| Östergötlands län | 2007 | 1090 | 4358 | 0.25 |
| Östergötlands län | 2008 | 1266 | 4382 | 0.29 |
| Östergötlands län | 2009 | 1194 | 4302 | 0.28 |
| Östergötlands län | 2010 | 1153 | 4164 | 0.28 |
| Östergötlands län | 2011 | 1149 | 4131 | 0.28 |
| Östergötlands län | 2012 | 1192 | 4290 | 0.28 |
| Östergötlands län | 2013 | 1104 | 4292 | 0.26 |
| Östergötlands län | 2014 | 1061 | 4138 | 0.26 |
| Östergötlands län | 2015 | 1006 | 4157 | 0.24 |
| Östergötlands län | 2016 | 995 | 4250 | 0.23 |
| Östergötlands län | 2017 | 1098 | 4382 | 0.25 |
| Östergötlands län | 2018 | 1080 | 4321 | 0.25 |
| Östergötlands län | 2019 | 1033 | 4136 | 0.25 |
| Östergötlands län | 2020 | 1053 | 4515 | 0.23 |
| Östergötlands län | 2021 | 903 | 4348 | 0.21 |
| Östergötlands län | 2022 | 1129 | 4373 | 0.26 |
| Östergötlands län | 2023 | 1097 | 4320 | 0.25 |
| Skåne län | 1997 | 3427 | 11981 | 0.29 |
| Skåne län | 1998 | 3369 | 12050 | 0.28 |
| Skåne län | 1999 | 3465 | 12297 | 0.28 |
| Skåne län | 2000 | 3448 | 12076 | 0.29 |
| Skåne län | 2001 | 3505 | 12189 | 0.29 |
| Skåne län | 2002 | 3575 | 12211 | 0.29 |
| Skåne län | 2003 | 3569 | 11869 | 0.30 |
| Skåne län | 2004 | 3168 | 11719 | 0.27 |
| Skåne län | 2005 | 3327 | 11845 | 0.28 |
| Skåne län | 2006 | 3149 | 11640 | 0.27 |
| Skåne län | 2007 | 3389 | 11952 | 0.28 |
| Skåne län | 2008 | 3342 | 11927 | 0.28 |
| Skåne län | 2009 | 3360 | 11525 | 0.29 |
| Skåne län | 2010 | 3571 | 11877 | 0.30 |
| Skåne län | 2011 | 3468 | 11603 | 0.30 |
| Skåne län | 2012 | 3796 | 12305 | 0.31 |
| Skåne län | 2013 | 3544 | 12149 | 0.29 |
| Skåne län | 2014 | 3388 | 11770 | 0.29 |
| Skåne län | 2015 | 3474 | 11810 | 0.29 |
| Skåne län | 2016 | 3287 | 11956 | 0.27 |
| Skåne län | 2017 | 3354 | 12073 | 0.28 |
| Skåne län | 2018 | 3383 | 12239 | 0.28 |
| Skåne län | 2019 | 3244 | 11742 | 0.28 |
| Skåne län | 2020 | 3310 | 12720 | 0.26 |
| Skåne län | 2021 | 3338 | 12394 | 0.27 |
| Skåne län | 2022 | 3670 | 12840 | 0.29 |
| Skåne län | 2023 | 3481 | 12662 | 0.27 |
| Södermanlands län | 1997 | 763 | 2814 | 0.27 |
| Södermanlands län | 1998 | 787 | 2840 | 0.28 |
| Södermanlands län | 1999 | 755 | 2963 | 0.25 |
| Södermanlands län | 2000 | 761 | 2856 | 0.27 |
| Södermanlands län | 2001 | 812 | 2966 | 0.27 |
| Södermanlands län | 2002 | 797 | 2891 | 0.28 |
| Södermanlands län | 2003 | 743 | 2797 | 0.27 |
| Södermanlands län | 2004 | 711 | 2783 | 0.26 |
| Södermanlands län | 2005 | 775 | 2780 | 0.28 |
| Södermanlands län | 2006 | 769 | 2842 | 0.27 |
| Södermanlands län | 2007 | 777 | 2883 | 0.27 |
| Södermanlands län | 2008 | 823 | 3006 | 0.27 |
| Södermanlands län | 2009 | 806 | 2959 | 0.27 |
| Södermanlands län | 2010 | 784 | 2838 | 0.28 |
| Södermanlands län | 2011 | 802 | 2884 | 0.28 |
| Södermanlands län | 2012 | 873 | 2937 | 0.30 |
| Södermanlands län | 2013 | 858 | 2837 | 0.30 |
| Södermanlands län | 2014 | 776 | 2772 | 0.28 |
| Södermanlands län | 2015 | 831 | 2923 | 0.28 |
| Södermanlands län | 2016 | 751 | 2956 | 0.25 |
| Södermanlands län | 2017 | 726 | 2972 | 0.24 |
| Södermanlands län | 2018 | 797 | 3011 | 0.26 |
| Södermanlands län | 2019 | 744 | 2891 | 0.26 |
| Södermanlands län | 2020 | 682 | 3202 | 0.21 |
| Södermanlands län | 2021 | 729 | 3080 | 0.24 |
| Södermanlands län | 2022 | 752 | 3101 | 0.24 |
| Södermanlands län | 2023 | 762 | 3201 | 0.24 |
| Stockholms län | 1997 | 4634 | 15723 | 0.29 |
| Stockholms län | 1998 | 4504 | 15749 | 0.29 |
| Stockholms län | 1999 | 4732 | 16060 | 0.29 |
| Stockholms län | 2000 | 4471 | 15817 | 0.28 |
| Stockholms län | 2001 | 4485 | 15781 | 0.28 |
| Stockholms län | 2002 | 4718 | 16080 | 0.29 |
| Stockholms län | 2003 | 4561 | 15953 | 0.29 |
| Stockholms län | 2004 | 4300 | 15720 | 0.27 |
| Stockholms län | 2005 | 4453 | 15531 | 0.29 |
| Stockholms län | 2006 | 4528 | 15686 | 0.29 |
| Stockholms län | 2007 | 4522 | 15812 | 0.29 |
| Stockholms län | 2008 | 4527 | 15576 | 0.29 |
| Stockholms län | 2009 | 4403 | 15179 | 0.29 |
| Stockholms län | 2010 | 4743 | 15638 | 0.30 |
| Stockholms län | 2011 | 4604 | 15567 | 0.30 |
| Stockholms län | 2012 | 4502 | 15559 | 0.29 |
| Stockholms län | 2013 | 4377 | 15255 | 0.29 |
| Stockholms län | 2014 | 4387 | 15666 | 0.28 |
| Stockholms län | 2015 | 4462 | 15867 | 0.28 |
| Stockholms län | 2016 | 4282 | 15953 | 0.27 |
| Stockholms län | 2017 | 4172 | 15878 | 0.26 |
| Stockholms län | 2018 | 4357 | 16231 | 0.27 |
| Stockholms län | 2019 | 4247 | 15405 | 0.28 |
| Stockholms län | 2020 | 3958 | 18493 | 0.21 |
| Stockholms län | 2021 | 3734 | 15660 | 0.24 |
| Stockholms län | 2022 | 4236 | 16224 | 0.26 |
| Stockholms län | 2023 | 4292 | 16077 | 0.27 |
| unknown | 1997 | 2320 | 7893 | 0.29 |
| unknown | 2012 | 6 | 33 | 0.18 |
| unknown | 2013 | 8 | 32 | 0.25 |
| unknown | 2014 | 8 | 37 | 0.22 |
| unknown | 2015 | 13 | 44 | 0.30 |
| unknown | 2016 | 4 | 26 | 0.15 |
| unknown | 2017 | 14 | 51 | 0.27 |
| unknown | 2018 | 14 | 40 | 0.35 |
| unknown | 2019 | 10 | 31 | 0.32 |
| unknown | 2020 | 11 | 55 | 0.20 |
| unknown | 2021 | 29 | 69 | 0.42 |
| Uppsala län | 1997 | 627 | 2379 | 0.26 |
| Uppsala län | 1998 | 687 | 2491 | 0.28 |
| Uppsala län | 1999 | 733 | 2429 | 0.30 |
| Uppsala län | 2000 | 707 | 2420 | 0.29 |
| Uppsala län | 2001 | 672 | 2527 | 0.27 |
| Uppsala län | 2002 | 680 | 2537 | 0.27 |
| Uppsala län | 2003 | 698 | 2483 | 0.28 |
| Uppsala län | 2004 | 661 | 2470 | 0.27 |
| Uppsala län | 2005 | 656 | 2473 | 0.27 |
| Uppsala län | 2006 | 600 | 2450 | 0.24 |
| Uppsala län | 2007 | 751 | 2740 | 0.27 |
| Uppsala län | 2008 | 765 | 2637 | 0.29 |
| Uppsala län | 2009 | 753 | 2664 | 0.28 |
| Uppsala län | 2010 | 779 | 2675 | 0.29 |
| Uppsala län | 2011 | 764 | 2733 | 0.28 |
| Uppsala län | 2012 | 790 | 2756 | 0.29 |
| Uppsala län | 2013 | 702 | 2730 | 0.26 |
| Uppsala län | 2014 | 728 | 2714 | 0.27 |
| Uppsala län | 2015 | 713 | 2756 | 0.26 |
| Uppsala län | 2016 | 682 | 2748 | 0.25 |
| Uppsala län | 2017 | 743 | 2829 | 0.26 |
| Uppsala län | 2018 | 758 | 2870 | 0.26 |
| Uppsala län | 2019 | 732 | 2776 | 0.26 |
| Uppsala län | 2020 | 745 | 3102 | 0.24 |
| Uppsala län | 2021 | 694 | 2966 | 0.23 |
| Uppsala län | 2022 | 798 | 3108 | 0.26 |
| Uppsala län | 2023 | 783 | 3044 | 0.26 |
| Värmlands län | 1997 | 1107 | 3525 | 0.31 |
| Värmlands län | 1998 | 1037 | 3386 | 0.31 |
| Värmlands län | 1999 | 1109 | 3559 | 0.31 |
| Värmlands län | 2000 | 1109 | 3506 | 0.32 |
| Värmlands län | 2001 | 1003 | 3392 | 0.30 |
| Värmlands län | 2002 | 922 | 3363 | 0.27 |
| Värmlands län | 2003 | 915 | 3372 | 0.27 |
| Värmlands län | 2004 | 896 | 3219 | 0.28 |
| Värmlands län | 2005 | 894 | 3297 | 0.27 |
| Värmlands län | 2006 | 890 | 3312 | 0.27 |
| Värmlands län | 2007 | 902 | 3176 | 0.28 |
| Värmlands län | 2008 | 949 | 3311 | 0.29 |
| Värmlands län | 2009 | 948 | 3352 | 0.28 |
| Värmlands län | 2010 | 944 | 3231 | 0.29 |
| Värmlands län | 2011 | 845 | 3046 | 0.28 |
| Värmlands län | 2012 | 937 | 3198 | 0.29 |
| Värmlands län | 2013 | 859 | 3209 | 0.27 |
| Värmlands län | 2014 | 798 | 3042 | 0.26 |
| Värmlands län | 2015 | 878 | 3251 | 0.27 |
| Värmlands län | 2016 | 786 | 3200 | 0.25 |
| Värmlands län | 2017 | 789 | 3134 | 0.25 |
| Värmlands län | 2018 | 794 | 3132 | 0.25 |
| Värmlands län | 2019 | 769 | 3009 | 0.26 |
| Värmlands län | 2020 | 750 | 3160 | 0.24 |
| Värmlands län | 2021 | 815 | 3255 | 0.25 |
| Värmlands län | 2022 | 811 | 3124 | 0.26 |
| Värmlands län | 2023 | 845 | 3244 | 0.26 |
| Västerbottens län | 1997 | 758 | 2602 | 0.29 |
| Västerbottens län | 1998 | 767 | 2622 | 0.29 |
| Västerbottens län | 1999 | 719 | 2704 | 0.27 |
| Västerbottens län | 2000 | 767 | 2669 | 0.29 |
| Västerbottens län | 2001 | 693 | 2597 | 0.27 |
| Västerbottens län | 2002 | 698 | 2755 | 0.25 |
| Västerbottens län | 2003 | 719 | 2645 | 0.27 |
| Västerbottens län | 2004 | 637 | 2561 | 0.25 |
| Västerbottens län | 2005 | 678 | 2569 | 0.26 |
| Västerbottens län | 2006 | 637 | 2553 | 0.25 |
| Västerbottens län | 2007 | 676 | 2530 | 0.27 |
| Västerbottens län | 2008 | 672 | 2585 | 0.26 |
| Västerbottens län | 2009 | 713 | 2630 | 0.27 |
| Västerbottens län | 2010 | 760 | 2611 | 0.29 |
| Västerbottens län | 2011 | 664 | 2617 | 0.25 |
| Västerbottens län | 2012 | 745 | 2709 | 0.28 |
| Västerbottens län | 2013 | 665 | 2726 | 0.24 |
| Västerbottens län | 2014 | 612 | 2575 | 0.24 |
| Västerbottens län | 2015 | 686 | 2662 | 0.26 |
| Västerbottens län | 2016 | 640 | 2611 | 0.25 |
| Västerbottens län | 2017 | 548 | 2601 | 0.21 |
| Västerbottens län | 2018 | 638 | 2745 | 0.23 |
| Västerbottens län | 2019 | 590 | 2622 | 0.23 |
| Västerbottens län | 2020 | 564 | 2611 | 0.22 |
| Västerbottens län | 2021 | 638 | 2765 | 0.23 |
| Västerbottens län | 2022 | 669 | 2898 | 0.23 |
| Västerbottens län | 2023 | 645 | 2668 | 0.24 |
| Västernorrlands län | 1997 | 882 | 3281 | 0.27 |
| Västernorrlands län | 1998 | 838 | 3246 | 0.26 |
| Västernorrlands län | 1999 | 870 | 3179 | 0.27 |
| Västernorrlands län | 2000 | 876 | 3154 | 0.28 |
| Västernorrlands län | 2001 | 862 | 3202 | 0.27 |
| Västernorrlands län | 2002 | 866 | 3156 | 0.27 |
| Västernorrlands län | 2003 | 860 | 3173 | 0.27 |
| Västernorrlands län | 2004 | 776 | 3010 | 0.26 |
| Västernorrlands län | 2005 | 782 | 3084 | 0.25 |
| Västernorrlands län | 2006 | 835 | 3036 | 0.28 |
| Västernorrlands län | 2007 | 810 | 3028 | 0.27 |
| Västernorrlands län | 2008 | 795 | 2970 | 0.27 |
| Västernorrlands län | 2009 | 815 | 3078 | 0.26 |
| Västernorrlands län | 2010 | 823 | 3036 | 0.27 |
| Västernorrlands län | 2011 | 828 | 2959 | 0.28 |
| Västernorrlands län | 2012 | 802 | 2946 | 0.27 |
| Västernorrlands län | 2013 | 794 | 2903 | 0.27 |
| Västernorrlands län | 2014 | 773 | 2911 | 0.27 |
| Västernorrlands län | 2015 | 818 | 3019 | 0.27 |
| Västernorrlands län | 2016 | 718 | 2878 | 0.25 |
| Västernorrlands län | 2017 | 682 | 2946 | 0.23 |
| Västernorrlands län | 2018 | 689 | 2817 | 0.24 |
| Västernorrlands län | 2019 | 672 | 2772 | 0.24 |
| Västernorrlands län | 2020 | 650 | 3083 | 0.21 |
| Västernorrlands län | 2021 | 659 | 2898 | 0.23 |
| Västernorrlands län | 2022 | 667 | 2972 | 0.22 |
| Västernorrlands län | 2023 | 637 | 2834 | 0.22 |
| Västmanlands län | 1997 | 801 | 2681 | 0.30 |
| Västmanlands län | 1998 | 857 | 2743 | 0.31 |
| Västmanlands län | 1999 | 856 | 2762 | 0.31 |
| Västmanlands län | 2000 | 776 | 2782 | 0.28 |
| Västmanlands län | 2001 | 800 | 2781 | 0.29 |
| Västmanlands län | 2002 | 907 | 2933 | 0.31 |
| Västmanlands län | 2003 | 809 | 2701 | 0.30 |
| Västmanlands län | 2004 | 815 | 2765 | 0.29 |
| Västmanlands län | 2005 | 883 | 2825 | 0.31 |
| Västmanlands län | 2006 | 815 | 2778 | 0.29 |
| Västmanlands län | 2007 | 831 | 2661 | 0.31 |
| Västmanlands län | 2008 | 815 | 2641 | 0.31 |
| Västmanlands län | 2009 | 776 | 2544 | 0.31 |
| Västmanlands län | 2010 | 776 | 2524 | 0.31 |
| Västmanlands län | 2011 | 777 | 2573 | 0.30 |
| Västmanlands län | 2012 | 757 | 2641 | 0.29 |
| Västmanlands län | 2013 | 709 | 2507 | 0.28 |
| Västmanlands län | 2014 | 675 | 2545 | 0.27 |
| Västmanlands län | 2015 | 716 | 2610 | 0.27 |
| Västmanlands län | 2016 | 754 | 2719 | 0.28 |
| Västmanlands län | 2017 | 685 | 2673 | 0.26 |
| Västmanlands län | 2018 | 757 | 2812 | 0.27 |
| Västmanlands län | 2019 | 679 | 2651 | 0.26 |
| Västmanlands län | 2020 | 638 | 2932 | 0.22 |
| Västmanlands län | 2021 | 641 | 2741 | 0.23 |
| Västmanlands län | 2022 | 688 | 2884 | 0.24 |
| Västmanlands län | 2023 | 666 | 2770 | 0.24 |
| Västra Götalands län | 1997 | 2388 | 8085 | 0.30 |
| Västra Götalands län | 1998 | 4488 | 15806 | 0.28 |
| Västra Götalands län | 1999 | 4607 | 15984 | 0.29 |
| Västra Götalands län | 2000 | 4444 | 15523 | 0.29 |
| Västra Götalands län | 2001 | 4571 | 15870 | 0.29 |
| Västra Götalands län | 2002 | 4527 | 15973 | 0.28 |
| Västra Götalands län | 2003 | 4404 | 15597 | 0.28 |
| Västra Götalands län | 2004 | 4071 | 15201 | 0.27 |
| Västra Götalands län | 2005 | 4052 | 15379 | 0.26 |
| Västra Götalands län | 2006 | 3970 | 15323 | 0.26 |
| Västra Götalands län | 2007 | 4120 | 15391 | 0.27 |
| Västra Götalands län | 2008 | 4234 | 15397 | 0.27 |
| Västra Götalands län | 2009 | 4135 | 14872 | 0.28 |
| Västra Götalands län | 2010 | 4237 | 15261 | 0.28 |
| Västra Götalands län | 2011 | 4177 | 14886 | 0.28 |
| Västra Götalands län | 2012 | 4136 | 15250 | 0.27 |
| Västra Götalands län | 2013 | 4125 | 15056 | 0.27 |
| Västra Götalands län | 2014 | 3838 | 14686 | 0.26 |
| Västra Götalands län | 2015 | 4095 | 14994 | 0.27 |
| Västra Götalands län | 2016 | 3846 | 15106 | 0.25 |
| Västra Götalands län | 2017 | 3891 | 15373 | 0.25 |
| Västra Götalands län | 2018 | 3791 | 15299 | 0.25 |
| Västra Götalands län | 2019 | 3710 | 15022 | 0.25 |
| Västra Götalands län | 2020 | 3652 | 16078 | 0.23 |
| Västra Götalands län | 2021 | 3655 | 15242 | 0.24 |
| Västra Götalands län | 2022 | 3995 | 15604 | 0.26 |
| Västra Götalands län | 2023 | 4093 | 15717 | 0.26 |

Table S13: Redistribution proportions of GCs to well-defined target causes of death

| Table S13: Redistribution proportions of GCs to well-defined target causes of death | | | | |
| --- | --- | --- | --- | --- |
| GC | Total GC deaths | Well-defined target | Redistributed deaths | Proportion redistributed |
| Abdomen and Pelvis Cancer | 5019 | colorectal cancer other | 401.0 | 8.0 |
| Abdomen and Pelvis Cancer | 5019 | esophageal cancer | 618.0 | 12.3 |
| Abdomen and Pelvis Cancer | 5019 | kidney cancer | 480.6 | 9.6 |
| Abdomen and Pelvis Cancer | 5019 | other cancer | 339.8 | 6.8 |
| Abdomen and Pelvis Cancer | 5019 | other non-hodgkin lymphoma | 862.5 | 17.2 |
| Abdomen and Pelvis Cancer | 5019 | ovarian cancer | 563.8 | 11.2 |
| Acute Respiratory Failure | 824 | chronic ischemic heart disease | 48.0 | 5.8 |
| Acute Respiratory Failure | 824 | influenza | 45.5 | 5.5 |
| Acute Respiratory Failure | 824 | ischemic stroke | 42.2 | 5.1 |
| Acute Respiratory Failure | 824 | tracheal, bronchus, and lung cancer | 71.6 | 8.7 |
| Acute Respiratory Failure | 824 | unspecified bronchitis and bronchiectasis | 116.7 | 14.2 |
| Acute kidney failure | 8669 | acute myocardial infarction | 629.1 | 7.3 |
| Acute kidney failure | 8669 | chronic ischemic heart disease | 625.2 | 7.2 |
| Acute kidney failure | 8669 | chronic kidney disease due to hypertension | 494.6 | 5.7 |
| Acute kidney failure | 8669 | influenza | 456.1 | 5.3 |
| Acute kidney failure | 8669 | ischemic stroke | 561.2 | 6.5 |
| Adrenal Site Cancer unspecified part of adrenal gland | 296 | neuroblastoma and other peripheral nervous cell tumors | 115.5 | 39.0 |
| Adrenal Site Cancer unspecified part of adrenal gland | 296 | other cancer | 133.1 | 45.0 |
| Adrenal Site Cancer unspecified part of adrenal gland | 296 | penis cancer | 14.8 | 5.0 |
| Adrenal Site Cancer unspecified part of adrenal gland | 296 | vulva cancer | 26.1 | 8.8 |
| Adrenal Unspecified Site Cancer in medulla or cortex | 27 | neuroblastoma and other peripheral nervous cell tumors | 25.5 | 94.4 |
| Adrenal Unspecified Site Cancer-parent cause | 210 | other cancer | 153.5 | 73.1 |
| Adrenal Unspecified Site Cancer-parent cause | 210 | penis cancer | 15.3 | 7.3 |
| Adrenal Unspecified Site Cancer-parent cause | 210 | vagina cancer | 12.4 | 5.9 |
| Adrenal Unspecified Site Cancer-parent cause | 210 | vulva cancer | 26.0 | 12.4 |
| Alcoholic hepatic failure | 2115 | alcohol use disorders | 109.0 | 5.2 |
| Alcoholic hepatic failure | 2115 | cirrhosis and other chronic liver diseases due to alcohol use | 348.9 | 16.5 |
| Alcoholic hepatic failure | 2115 | cirrhosis and other chronic liver diseases due to hepatitis b | 347.0 | 16.4 |
| Alcoholic hepatic failure | 2115 | cirrhosis and other chronic liver diseases due to hepatitis c | 347.7 | 16.4 |
| Alcoholic hepatic failure | 2115 | cirrhosis and other chronic liver diseases due to other causes | 347.2 | 16.4 |
| Alcoholic hepatic failure | 2115 | cirrhosis due to nash | 347.2 | 16.4 |
| All, Ill Defined code for causes of death | 46434 | acute myocardial infarction | 6041.9 | 13.0 |
| All, Ill Defined code for causes of death | 46434 | chronic ischemic heart disease | 5923.8 | 12.8 |
| All, Ill Defined code for causes of death | 46434 | tracheal, bronchus, and lung cancer | 3021.3 | 6.5 |
| Amyloidosis | 1500 | chronic kidney disease due to hypertension | 116.3 | 7.8 |
| Amyloidosis | 1500 | multiple myeloma | 819.8 | 54.7 |
| Amyloidosis | 1500 | other cardiomyopathy | 358.5 | 23.9 |
| Anemia Unspecified | 3879 | aplastic anemias | 281.9 | 7.3 |
| Anemia Unspecified | 3879 | chronic kidney disease due to diabetes mellitus type 2 | 198.7 | 5.1 |
| Anemia Unspecified | 3879 | chronic kidney disease due to hypertension | 325.8 | 8.4 |
| Anemia Unspecified | 3879 | chronic kidney disease due to other and unspecified causes | 2221.0 | 57.3 |
| Arterial Embolism | 1968 | acute myocardial infarction | 382.0 | 19.4 |
| Arterial Embolism | 1968 | aortic aneurysm | 140.2 | 7.1 |
| Arterial Embolism | 1968 | chronic ischemic heart disease | 337.7 | 17.2 |
| Arterial Embolism | 1968 | ischemic stroke | 443.7 | 22.5 |
| Assault by unspecified means | 114 | all causes | 11.0 | 9.6 |
| Assault by unspecified means | 114 | physical violence by firearm | 20.2 | 17.7 |
| Assault by unspecified means | 114 | physical violence by other means | 47.5 | 41.7 |
| Assault by unspecified means | 114 | physical violence by sharp object | 34.3 | 30.1 |
| Assigned death to tobacco | 106 | tracheal, bronchus, and lung cancer | 96.2 | 90.8 |
| Assigned death to tobacco | 106 | unspecified bronchitis and bronchiectasis | 8.0 | 7.5 |
| Atherosclerosis | 26743 | acute myocardial infarction | 6707.1 | 25.1 |
| Atherosclerosis | 26743 | aortic aneurysm | 1406.4 | 5.3 |
| Atherosclerosis | 26743 | chronic ischemic heart disease | 6367.0 | 23.8 |
| Atherosclerosis | 26743 | ischemic stroke | 9286.3 | 34.7 |
| CKD due to diabetes Unspecified type | 3999 | chronic kidney disease due to diabetes mellitus type 1 | 474.8 | 11.9 |
| CKD due to diabetes Unspecified type | 3999 | chronic kidney disease due to diabetes mellitus type 2 | 3524.2 | 88.1 |
| CNS Abscess | 223 | diabetes mellitus type 1 | 13.3 | 5.9 |
| CNS Abscess | 223 | diabetes mellitus type 2 | 45.9 | 20.6 |
| CNS Abscess | 223 | endocarditis | 19.0 | 8.5 |
| CNS Abscess | 223 | falls | 72.4 | 32.5 |
| CNS Abscess | 223 | motor vehicle road injuries | 23.2 | 10.4 |
| CNS Abscess | 223 | pyoderma | 18.8 | 8.4 |
| CNS Fluid Diseases | 29 | acute myocardial infarction | 23.7 | 81.9 |
| CNS Fluid Diseases | 29 | hypertensive heart disease | 3.3 | 11.4 |
| Cardiac rhythm disorders | 6951 | acute myocardial infarction | 2099.2 | 30.2 |
| Cardiac rhythm disorders | 6951 | chronic ischemic heart disease | 2198.1 | 31.6 |
| Cardiac rhythm disorders | 6951 | ischemic stroke | 850.7 | 12.2 |
| Cerebral Cysts | 22 | brain and central nervous system cancer | 1.7 | 7.6 |
| Cerebral Cysts | 22 | falls | 2.7 | 12.5 |
| Cerebral Cysts | 22 | self-harm by hanging, strangulation, and suffocation | 2.9 | 13.3 |
| Cerebral Cysts | 22 | tracheal, bronchus, and lung cancer | 6.9 | 31.6 |
| Cerebral Palsy | 839 | colorectal cancer other | 42.7 | 5.1 |
| Cerebral Palsy | 839 | congenital others | 48.8 | 5.8 |
| Cerebral Palsy | 839 | epilepsy | 60.6 | 7.2 |
| Cerebral Palsy | 839 | influenza | 81.7 | 9.7 |
| Cerebral Palsy | 839 | other lower respiratory infections | 43.4 | 5.2 |
| Cerebral Palsy | 839 | paralytic ileus and intestinal obstruction | 67.5 | 8.0 |
| Chronic lymphocytic leukemia by age | 5551 | chronic lymphoid leukemia | 5550.0 | 100.0 |
| Chronic respiratory failure | 566 | acute myocardial infarction | 43.0 | 7.6 |
| Chronic respiratory failure | 566 | chronic ischemic heart disease | 51.1 | 9.0 |
| Chronic respiratory failure | 566 | influenza | 78.7 | 13.9 |
| Chronic respiratory failure | 566 | intracerebral hemorrhage | 29.5 | 5.2 |
| Chronic respiratory failure | 566 | ischemic stroke | 87.8 | 15.5 |
| Chronic respiratory failure | 566 | tracheal, bronchus, and lung cancer | 32.4 | 5.7 |
| Chronic respiratory failure | 566 | unspecified bronchitis and bronchiectasis | 54.3 | 9.6 |
| Diabetes unspecified type | 31213 | all causes | 3417.0 | 10.9 |
| Diabetes unspecified type | 31213 | diabetes mellitus type 1 | 1585.5 | 5.1 |
| Diabetes unspecified type | 31213 | diabetes mellitus type 2 | 26210.5 | 84.0 |
| Exposure to unspecified factor X59 | 25718 | falls | 23132.7 | 89.9 |
| External Causes UDI, type unspecified | 2324 | falls | 1211.4 | 52.1 |
| External Causes UDI, type unspecified | 2324 | fire, heat, and hot substances | 195.5 | 8.4 |
| External Causes UDI, type unspecified | 2324 | self-harm by drowning | 207.9 | 8.9 |
| External Causes UDI, type unspecified | 2324 | self-harm by jumping | 184.6 | 7.9 |
| Eye Unspecified Site Cancer | 370 | other eye cancers | 369.0 | 99.7 |
| Female pelvic inflammatory diseases | 102 | all causes | 13.0 | 12.7 |
| Female pelvic inflammatory diseases | 102 | chlamydial infection | 8.2 | 8.0 |
| Female pelvic inflammatory diseases | 102 | other sexually transmitted infections | 79.0 | 77.5 |
| Fistula | 203 | bladder cancer | 15.1 | 7.4 |
| Fistula | 203 | colorectal cancer other | 10.7 | 5.3 |
| Fistula | 203 | decubitus ulcer | 15.1 | 7.4 |
| Fistula | 203 | diverticular disease of intestines | 15.1 | 7.4 |
| Fistula | 203 | gonococcal infection | 10.4 | 5.1 |
| Fistula | 203 | other cancer | 10.5 | 5.2 |
| Fistula | 203 | other unintentional injuries | 15.1 | 7.4 |
| Fistula | 203 | post procedural or drug treatment disorders | 30.3 | 14.9 |
| Fistula | 203 | urinary tract infections | 15.1 | 7.4 |
| Fistula | 203 | urolithiasis | 15.1 | 7.4 |
| Fluid, Electrolyte, Acid Base Disorders | 2767 | chronic kidney disease due to hypertension | 165.0 | 6.0 |
| Fluid, Electrolyte, Acid Base Disorders | 2767 | diabetes mellitus type 2 | 204.0 | 7.4 |
| Fluid, Electrolyte, Acid Base Disorders | 2767 | influenza | 140.4 | 5.1 |
| Fluid, Electrolyte, Acid Base Disorders | 2767 | ischemic stroke | 295.9 | 10.7 |
| Fluid, Electrolyte, Acid Base Disorders | 2767 | other diarrheal diseases | 141.7 | 5.1 |
| HIV correction for Actinomycosis | 7 | non-genital herpes infection | 3.3 | 47.3 |
| HIV correction for Aspergillosis | 91 | hiv/aids resulting in other diseases | 17.1 | 18.8 |
| HIV correction for Aspergillosis | 91 | non-genital herpes infection | 10.2 | 11.2 |
| HIV correction for Candidiasis | 268 | listeriosis | 25.1 | 9.4 |
| HIV correction for Candidiasis | 268 | non-genital herpes infection | 60.5 | 22.6 |
| HIV correction for Coccidioidomycosis | 1 | acute myocardial infarction | 0.1 | 14.7 |
| HIV correction for Coccidioidomycosis | 1 | chronic ischemic heart disease | 0.1 | 12.4 |
| HIV correction for Coccidioidomycosis | 1 | ischemic stroke | 0.2 | 15.6 |
| HIV correction for Coccidioidomycosis | 1 | unspecified dementia | 0.1 | 7.1 |
| HIV correction for Cryptococcosis | 5 | all causes | 1.0 | 20.0 |
| HIV correction for Cryptococcosis | 5 | hiv/aids resulting in other diseases | 3.0 | 60.0 |
| HIV correction for Histoplasmosis | 1 | anthrax | 0.1 | 7.7 |
| HIV correction for Histoplasmosis | 1 | brucellosis | 0.1 | 7.7 |
| HIV correction for Histoplasmosis | 1 | cytomegaloviral diseases | 0.1 | 7.7 |
| HIV correction for Histoplasmosis | 1 | leptospirosis | 0.1 | 7.7 |
| HIV correction for Histoplasmosis | 1 | listeriosis | 0.1 | 7.7 |
| HIV correction for Histoplasmosis | 1 | lyme disease | 0.1 | 7.7 |
| HIV correction for Histoplasmosis | 1 | mononucleosis | 0.1 | 7.7 |
| HIV correction for Histoplasmosis | 1 | mumps | 0.1 | 7.7 |
| HIV correction for Histoplasmosis | 1 | non-genital herpes infection | 0.1 | 7.7 |
| HIV correction for Histoplasmosis | 1 | other drug-resistant infectious diseases | 0.1 | 7.7 |
| HIV correction for Histoplasmosis | 1 | pasteurellosis | 0.1 | 7.7 |
| HIV correction for Histoplasmosis | 1 | rubella | 0.1 | 7.7 |
| HIV correction for Histoplasmosis | 1 | tularemia | 0.1 | 7.7 |
| HIV correction for Immunodeficiency antibody | 68 | endo other than secret cause | 16.5 | 24.2 |
| HIV correction for Immunodeficiency antibody | 68 | hiv/aids resulting in other diseases | 9.8 | 14.5 |
| HIV correction for Immunodeficiency antibody | 68 | lipoprotein metabolism and other lipidaemias disorders | 6.2 | 9.2 |
| HIV correction for Immunodeficiency antibody | 68 | obesity | 4.6 | 6.8 |
| HIV correction for Immunodeficiency cell | 46 | cystic fibrosis | 2.4 | 5.2 |
| HIV correction for Immunodeficiency cell | 46 | endo other than secret cause | 26.3 | 57.1 |
| HIV correction for Immunodeficiency cell | 46 | hiv/aids resulting in other diseases | 2.7 | 6.0 |
| HIV correction for Immunodeficiency cell | 46 | lipoprotein metabolism and other lipidaemias disorders | 3.5 | 7.6 |
| HIV correction for Immunodeficiency cell | 46 | obesity | 6.3 | 13.7 |
| HIV correction for Immunodeficiency other | 413 | acute myocardial infarction | 30.8 | 7.4 |
| HIV correction for Immunodeficiency other | 413 | chronic ischemic heart disease | 31.8 | 7.7 |
| HIV correction for Immunodeficiency other | 413 | endo other than secret cause | 34.7 | 8.4 |
| HIV correction for Immunodeficiency other | 413 | hiv/aids resulting in other diseases | 42.1 | 10.2 |
| HIV correction for Immunodeficiency other | 413 | ischemic stroke | 24.5 | 5.9 |
| HIV correction for Kaposi's sarcoma | 33 | soft tissue and other extraosseous sarcomas | 30.9 | 93.5 |
| HIV correction for Nocardiosis | 4 | acute myocardial infarction | 0.3 | 8.7 |
| HIV correction for Nocardiosis | 4 | chronic ischemic heart disease | 0.3 | 8.0 |
| HIV correction for Nocardiosis | 4 | severe acute respiratory syndrome coronavirus 2 | 0.3 | 7.3 |
| HIV correction for Nocardiosis | 4 | unspecified dementia | 0.2 | 5.2 |
| HIV correction for Other Mycobacterial infection | 145 | acute myocardial infarction | 11.7 | 8.1 |
| HIV correction for Other Mycobacterial infection | 145 | chronic ischemic heart disease | 9.9 | 6.8 |
| HIV correction for Other Mycobacterial infection | 145 | ischemic stroke | 8.4 | 5.8 |
| HIV correction for Other Mycobacterial infection | 145 | non-genital herpes infection | 10.8 | 7.5 |
| HIV correction for Pneumocystosis | 36 | acute myocardial infarction | 2.6 | 7.1 |
| HIV correction for Pneumocystosis | 36 | chronic ischemic heart disease | 2.0 | 5.7 |
| HIV correction for Pneumocystosis | 36 | hiv/aids resulting in other diseases | 6.0 | 16.6 |
| HIV correction for Toxoplasmosis | 8 | hiv/aids resulting in other diseases | 0.4 | 5.0 |
| HIV correction for Toxoplasmosis | 8 | listeriosis | 2.4 | 29.5 |
| HIV correction for Unspecified mycosis | 109 | listeriosis | 7.7 | 7.1 |
| HIV correction for Unspecified mycosis | 109 | non-genital herpes infection | 17.0 | 15.6 |
| HIV correction for Urogenital Candidiasis | 1 | genital candidiasis | 0.9 | 86.4 |
| HIV correction for Zygomycosis | 5 | non-genital herpes infection | 0.5 | 10.1 |
| HIV correction for Zygomycosis | 5 | pasteurellosis | 0.6 | 11.6 |
| Haemophilus influenza infection, unspecified site | 12 | cellulitis | 5.4 | 45.1 |
| Haemophilus influenza infection, unspecified site | 12 | h influenzae type b meningitis | 0.8 | 6.3 |
| Haemophilus influenza infection, unspecified site | 12 | h influenzae type b pneumonia | 3.7 | 30.6 |
| Haemophilus influenza infection, unspecified site | 12 | upper respiratory infections | 2.2 | 18.0 |
| Head and Neck Cancer | 557 | brain and central nervous system cancer | 65.3 | 11.7 |
| Head and Neck Cancer | 557 | breast cancer | 105.2 | 18.9 |
| Head and Neck Cancer | 557 | non-melanoma skin cancer (squamous-cell carcinoma) | 30.0 | 5.4 |
| Head and Neck Cancer | 557 | other cancer | 36.9 | 6.6 |
| Head and Neck Cancer | 557 | thyroid cancer | 39.5 | 7.1 |
| Head and Neck Cancer | 557 | tracheal, bronchus, and lung cancer | 242.6 | 43.6 |
| Heart failure unspecified right or left | 87520 | acute myocardial infarction | 20223.7 | 23.1 |
| Heart failure unspecified right or left | 87520 | chronic ischemic heart disease | 22800.9 | 26.1 |
| Heart failure unspecified right or left | 87520 | hypertensive heart disease | 7395.5 | 8.5 |
| Heart failure unspecified right or left | 87520 | ischemic stroke | 5007.4 | 5.7 |
| Heart failure unspecified right or left | 87520 | non-rheumatic calcific aortic valve disease | 5960.3 | 6.8 |
| Heart failure unspecified right or left | 87520 | unspecified bronchitis and bronchiectasis | 4866.6 | 5.6 |
| Hepatic Failure | 2183 | breast cancer | 112.8 | 5.2 |
| Hepatic Failure | 2183 | colorectal cancer other | 159.5 | 7.3 |
| Hepatic Failure | 2183 | gallbladder and biliary tract cancer | 117.0 | 5.4 |
| Hepatic Failure | 2183 | pancreatic cancer | 224.5 | 10.3 |
| Hepatitis Unspecified | 100 | cirrhosis and other chronic liver diseases due to alcohol use | 19.0 | 19.0 |
| Hepatitis Unspecified | 100 | cirrhosis and other chronic liver diseases due to hepatitis b | 19.2 | 19.2 |
| Hepatitis Unspecified | 100 | cirrhosis and other chronic liver diseases due to hepatitis c | 19.2 | 19.2 |
| Hepatitis Unspecified | 100 | cirrhosis and other chronic liver diseases due to other causes | 19.2 | 19.2 |
| Hepatitis Unspecified | 100 | cirrhosis due to nash | 18.9 | 18.9 |
| Hypertension | 14675 | acute myocardial infarction | 2656.2 | 18.1 |
| Hypertension | 14675 | chronic ischemic heart disease | 3217.6 | 21.9 |
| Hypertension | 14675 | chronic kidney disease due to hypertension | 1073.6 | 7.3 |
| Hypertension | 14675 | hypertensive heart disease | 1918.2 | 13.1 |
| Hypertension | 14675 | intracerebral hemorrhage | 2076.8 | 14.2 |
| Hypertension | 14675 | ischemic stroke | 2982.5 | 20.3 |
| Intermediate cause for CNS | 1679 | acute myocardial infarction | 136.5 | 8.1 |
| Intermediate cause for CNS | 1679 | brain and central nervous system cancer | 144.7 | 8.6 |
| Intermediate cause for CNS | 1679 | chronic ischemic heart disease | 113.1 | 6.7 |
| Intermediate cause for CNS | 1679 | intracerebral hemorrhage | 230.3 | 13.7 |
| Intermediate cause for CNS | 1679 | ischemic stroke | 273.3 | 16.3 |
| Intermediate cause for CNS | 1679 | subarachnoid hemorrhage | 103.8 | 6.2 |
| Left heart failure | 6877 | acute myocardial infarction | 1860.9 | 27.1 |
| Left heart failure | 6877 | chronic ischemic heart disease | 1758.9 | 25.6 |
| Left heart failure | 6877 | hypertensive heart disease | 641.4 | 9.3 |
| Left heart failure | 6877 | non-rheumatic calcific aortic valve disease | 511.1 | 7.4 |
| Left heart failure | 6877 | unspecified bronchitis and bronchiectasis | 429.8 | 6.2 |
| Liver Abscess | 162 | amoebiasis | 9.1 | 5.6 |
| Liver Abscess | 162 | other digestive diseases except secreute causes | 128.6 | 79.4 |
| Liver Abscess | 162 | other salmonella infections | 12.3 | 7.6 |
| Lymphoid leukemia unspecified by age | 354 | other leukemia | 344.0 | 97.2 |
| MDS not classified | 23 | acute myeloid leukemia | 19.1 | 83.0 |
| MDS not classified | 23 | other leukemia | 3.9 | 17.0 |
| Mental Disorders | 4850 | alzheimer disease | 290.1 | 6.0 |
| Mental Disorders | 4850 | falls | 377.8 | 7.8 |
| Mental Disorders | 4850 | influenza | 1264.7 | 26.1 |
| Mental Disorders | 4850 | other lower respiratory infections | 779.0 | 16.1 |
| Mental Disorders | 4850 | unspecified dementia | 453.8 | 9.4 |
| Mental Disorders | 4850 | urinary tract infections | 317.0 | 6.5 |
| Myocardial Degeneration | 372 | alcoholic cardiomyopathy | 40.3 | 10.8 |
| Myocardial Degeneration | 372 | myocarditis | 124.2 | 33.4 |
| Myocardial Degeneration | 372 | other cardiomyopathy | 207.5 | 55.8 |
| Non-follicular lymphoma, unspecified | 11676 | other non-hodgkin lymphoma | 11570.1 | 99.1 |
| Osteomyelitis | 466 | diabetes mellitus type 2 | 186.7 | 40.1 |
| Osteomyelitis | 466 | falls | 68.9 | 14.8 |
| Osteomyelitis | 466 | peripheral artery disease | 37.8 | 8.1 |
| Osteomyelitis | 466 | pyoderma | 72.2 | 15.5 |
| Osteomyelitis | 466 | systemic and discoid lupus erythematous | 34.8 | 7.5 |
| Peritonitis & Acute Abdomen | 1366 | colorectal cancer other | 170.6 | 12.5 |
| Peritonitis & Acute Abdomen | 1366 | esophageal diseases | 91.1 | 6.7 |
| Peritonitis & Acute Abdomen | 1366 | gallbladder and biliary diseases | 75.7 | 5.5 |
| Peritonitis & Acute Abdomen | 1366 | paralytic ileus and intestinal obstruction | 157.0 | 11.5 |
| Peritonitis & Acute Abdomen | 1366 | peptic ulcer disease | 182.4 | 13.4 |
| Peritonitis & Acute Abdomen | 1366 | rectum cancer | 71.1 | 5.2 |
| Peritonitis & Acute Abdomen | 1366 | vascular intestinal disorders | 80.8 | 5.9 |
| Pleurisy, Pyothorax | 1113 | influenza | 182.2 | 16.4 |
| Pleurisy, Pyothorax | 1113 | other lower respiratory infections | 93.8 | 8.4 |
| Pleurisy, Pyothorax | 1113 | other non-hodgkin lymphoma | 96.7 | 8.7 |
| Pleurisy, Pyothorax | 1113 | tracheal, bronchus, and lung cancer | 429.8 | 38.6 |
| Pneumoconiosis associated with tuberculosis | 4 | asbestosis | 1.5 | 36.4 |
| Pneumoconiosis associated with tuberculosis | 4 | respiratory tuberculosis | 1.3 | 32.4 |
| Pneumoconiosis associated with tuberculosis | 4 | silicosis | 0.9 | 21.9 |
| Pneumoconiosis associated with tuberculosis | 4 | tuberculosis of bones and joints | 0.2 | 6.2 |
| Pneumonitis | 3875 | ischemic stroke | 1169.2 | 30.2 |
| Primary or secondary Liver Cancer Unspecified | 9378 | bladder cancer | 529.5 | 5.6 |
| Primary or secondary Liver Cancer Unspecified | 9378 | breast cancer | 809.8 | 8.6 |
| Primary or secondary Liver Cancer Unspecified | 9378 | colorectal cancer other | 1469.7 | 15.7 |
| Primary or secondary Liver Cancer Unspecified | 9378 | kidney cancer | 530.3 | 5.7 |
| Primary or secondary Liver Cancer Unspecified | 9378 | pancreatic cancer | 1263.6 | 13.5 |
| Primary or secondary Liver Cancer Unspecified | 9378 | prostate cancer | 1926.6 | 20.5 |
| Primary or secondary Liver Cancer Unspecified | 9378 | rectum cancer | 654.8 | 7.0 |
| Primary or secondary Liver Cancer Unspecified | 9378 | stomach cancer | 691.7 | 7.4 |
| Pulmonary Embolism | 13117 | acute myocardial infarction | 835.7 | 6.4 |
| Pulmonary Embolism | 13117 | chronic ischemic heart disease | 701.9 | 5.4 |
| Pulmonary Embolism | 13117 | falls | 1100.9 | 8.4 |
| Pulmonary Embolism | 13117 | ischemic stroke | 661.5 | 5.0 |
| Pulmonary Embolism | 13117 | other cardiovascular and circulatory diseases except varicose andphlebitis | 1133.0 | 8.6 |
| Pulmonary Embolism | 13117 | phlebitis and thrombophlebitis | 799.5 | 6.1 |
| Pulmonary Embolism | 13117 | tracheal, bronchus, and lung cancer | 777.5 | 5.9 |
| Self-harm by unspecified means | 48 | self-harm by drowning | 5.3 | 11.0 |
| Self-harm by unspecified means | 48 | self-harm by firearm | 6.7 | 13.9 |
| Self-harm by unspecified means | 48 | self-harm by hanging, strangulation, and suffocation | 23.0 | 47.9 |
| Self-harm by unspecified means | 48 | self-harm by jumping | 4.9 | 10.1 |
| Self-harm by unspecified means | 48 | self-harm by non pesticide substance and gas | 7.1 | 14.8 |
| Self-poisoning unspecified | 3367 | self-harm by non pesticide substance and gas | 3323.5 | 98.7 |
| Senility | 31491 | acute myocardial infarction | 5529.8 | 17.6 |
| Senility | 31491 | chronic ischemic heart disease | 7872.7 | 25.0 |
| Senility | 31491 | ischemic stroke | 2142.8 | 6.8 |
| Sepsis (Non- maternal and neonatal sepsis) | 20024 | influenza | 1020.8 | 5.1 |
| Sepsis (Non- maternal and neonatal sepsis) | 20024 | ischemic stroke | 1071.8 | 5.4 |
| Sepsis (Non- maternal and neonatal sepsis) | 20024 | urinary tract infections | 1271.0 | 6.3 |
| Shock, Cardiac Arrest, Coma | 8962 | acute myocardial infarction | 1475.1 | 16.5 |
| Shock, Cardiac Arrest, Coma | 8962 | chronic ischemic heart disease | 1539.1 | 17.2 |
| Shock, Cardiac Arrest, Coma | 8962 | ischemic stroke | 670.2 | 7.5 |
| Undetermined intent Drowning | 873 | drowning | 254.5 | 29.2 |
| Undetermined intent Drowning | 873 | physical violence by other means | 72.2 | 8.3 |
| Undetermined intent Drowning | 873 | self-harm by drowning | 274.8 | 31.5 |
| Undetermined intent Drowning | 873 | self-harm by jumping | 271.6 | 31.1 |
| Undetermined intent shooting by Handgun Firearm | 20 | physical violence by firearm | 6.7 | 33.7 |
| Undetermined intent shooting by Handgun Firearm | 20 | self-harm by firearm | 8.5 | 42.5 |
| Undetermined intent shooting by Handgun Firearm | 20 | unintentional firearm injuries | 4.8 | 23.9 |
| Undetermined intent shooting by rifle and larger firearm | 21 | physical violence by firearm | 6.2 | 29.7 |
| Undetermined intent shooting by rifle and larger firearm | 21 | self-harm by firearm | 6.6 | 31.4 |
| Undetermined intent shooting by rifle and larger firearm | 21 | unintentional firearm injuries | 8.2 | 38.9 |
| Undetermined intent shooting by unspecified firearm | 32 | physical violence by firearm | 8.4 | 26.3 |
| Undetermined intent shooting by unspecified firearm | 32 | self-harm by firearm | 12.1 | 38.0 |
| Undetermined intent shooting by unspecified firearm | 32 | unintentional firearm injuries | 11.4 | 35.7 |
| Undetermined intent Poisoning by antiepileptic and psychotropic drugs | 1505 | opioid use disorders | 81.7 | 5.4 |
| Undetermined intent Poisoning by antiepileptic and psychotropic drugs | 1505 | physical violence by other means | 110.8 | 7.4 |
| Undetermined intent Poisoning by antiepileptic and psychotropic drugs | 1505 | self-harm by drowning | 127.8 | 8.5 |
| Undetermined intent Poisoning by antiepileptic and psychotropic drugs | 1505 | self-harm by firearm | 115.0 | 7.6 |
| Undetermined intent Poisoning by antiepileptic and psychotropic drugs | 1505 | self-harm by hanging, strangulation, and suffocation | 537.9 | 35.7 |
| Undetermined intent Poisoning by antiepileptic and psychotropic drugs | 1505 | self-harm by jumping | 95.6 | 6.4 |
| Undetermined intent Poisoning by antiepileptic and psychotropic drugs | 1505 | self-harm by non pesticide substance and gas | 254.9 | 16.9 |
| Undetermined intent Poisoning by autonomic nervous system drugs | 21 | physical violence by other means | 6.0 | 28.4 |
| Undetermined intent Poisoning by autonomic nervous system drugs | 21 | poisoning by pesticides | 11.2 | 53.3 |
| Undetermined intent Poisoning by autonomic nervous system drugs | 21 | self-harm by drowning | 2.1 | 9.8 |
| Undetermined intent Poisoning by autonomic nervous system drugs | 21 | self-harm by jumping | 1.8 | 8.5 |
| Undetermined intent Poisoning by multiple or unspecified drug | 7941 | all causes | 2453.0 | 30.9 |
| Undetermined intent Poisoning by multiple or unspecified drug | 7941 | opioid use disorders | 4053.8 | 51.0 |
| Undetermined intent Poisoning by multiple or unspecified drug | 7941 | other drug use disorders | 765.6 | 9.6 |
| Undetermined intent Poisoning by narcotics and psychodysleptics drugs | 1429 | amphetamine use disorders | 176.4 | 12.3 |
| Undetermined intent Poisoning by narcotics and psychodysleptics drugs | 1429 | opioid use disorders | 373.7 | 26.2 |
| Undetermined intent Poisoning by narcotics and psychodysleptics drugs | 1429 | other drug use disorders | 221.9 | 15.5 |
| Undetermined intent Poisoning by narcotics and psychodysleptics drugs | 1429 | physical violence by other means | 86.8 | 6.1 |
| Undetermined intent Poisoning by narcotics and psychodysleptics drugs | 1429 | self-harm by hanging, strangulation, and suffocation | 231.5 | 16.2 |
| Undetermined intent Poisoning by narcotics and psychodysleptics drugs | 1429 | self-harm by non pesticide substance and gas | 87.5 | 6.1 |
| Undetermined intent Poisoning by no opioid analgesics | 181 | physical violence by other means | 67.3 | 37.2 |
| Undetermined intent Poisoning by no opioid analgesics | 181 | poisoning by pesticides | 27.8 | 15.3 |
| Undetermined intent Poisoning by no opioid analgesics | 181 | self-harm by drowning | 45.2 | 25.0 |
| Undetermined intent Poisoning by no opioid analgesics | 181 | self-harm by jumping | 40.8 | 22.5 |
| Undetermined intent Poisoning by other gases and vapors | 66 | physical violence by other means | 4.9 | 7.5 |
| Undetermined intent Poisoning by other gases and vapors | 66 | poisoning by carbon monoxide | 9.1 | 13.7 |
| Undetermined intent Poisoning by other gases and vapors | 66 | self-harm by drowning | 24.4 | 36.9 |
| Undetermined intent Poisoning by other gases and vapors | 66 | self-harm by jumping | 27.6 | 41.9 |
| Undetermined intent Poisoning by pesticides | 7 | physical violence by other means | 4.1 | 58.5 |
| Undetermined intent Poisoning by pesticides | 7 | poisoning by pesticides | 2.4 | 34.2 |
| Undetermined intent Poisoning by pesticides | 7 | self-harm by poisoning pesticides | 0.5 | 7.2 |
| Undetermined intent Poisoning by solvents and halogenated hydrocarbons | 17 | physical violence by other means | 7.0 | 41.4 |
| Undetermined intent Poisoning by solvents and halogenated hydrocarbons | 17 | poisoning by carbon monoxide | 6.6 | 38.7 |
| Undetermined intent Poisoning by solvents and halogenated hydrocarbons | 17 | self-harm by drowning | 1.8 | 10.5 |
| Undetermined intent Poisoning by solvents and halogenated hydrocarbons | 17 | self-harm by jumping | 1.6 | 9.3 |
| Undetermined intent Poisoning by unspecified chemicals and noxious substances | 22 | other drug use disorders | 2.3 | 10.4 |
| Undetermined intent Poisoning by unspecified chemicals and noxious substances | 22 | physical violence by other means | 5.9 | 26.8 |
| Undetermined intent Poisoning by unspecified chemicals and noxious substances | 22 | self-harm by drowning | 7.2 | 32.9 |
| Undetermined intent Poisoning by unspecified chemicals and noxious substances | 22 | self-harm by jumping | 6.6 | 29.9 |
| Undetermined intent Poisoning by unspecified drugs and biological drugs | 2039 | physical violence by other means | 137.3 | 6.7 |
| Undetermined intent Poisoning by unspecified drugs and biological drugs | 2039 | poisoning by carbon monoxide | 230.2 | 11.3 |
| Undetermined intent Poisoning by unspecified drugs and biological drugs | 2039 | poisoning by pesticides | 242.9 | 11.9 |
| Undetermined intent Poisoning by unspecified drugs and biological drugs | 2039 | self-harm by drowning | 146.4 | 7.2 |
| Undetermined intent Poisoning by unspecified drugs and biological drugs | 2039 | self-harm by firearm | 131.9 | 6.5 |
| Undetermined intent Poisoning by unspecified drugs and biological drugs | 2039 | self-harm by hanging, strangulation, and suffocation | 704.9 | 34.6 |
| Undetermined intent Poisoning by unspecified drugs and biological drugs | 2039 | self-harm by jumping | 119.4 | 5.9 |
| Undetermined intent Poisoning by unspecified drugs and biological drugs | 2039 | self-harm by non pesticide substance and gas | 298.1 | 14.6 |
| Undetermined intent Strangulation | 136 | physical violence by other means | 20.3 | 14.9 |
| Undetermined intent Strangulation | 136 | pulmonary aspiration and foreign body in airway | 22.2 | 16.3 |
| Undetermined intent Strangulation | 136 | self-harm by drowning | 27.1 | 19.9 |
| Undetermined intent Strangulation | 136 | self-harm by hanging, strangulation, and suffocation | 28.6 | 21.0 |
| Undetermined intent Strangulation | 136 | self-harm by jumping | 37.8 | 27.8 |
| Undetermined intent of Blunt Objects | 27 | other exposure to mechanical forces | 9.0 | 33.4 |
| Undetermined intent of Blunt Objects | 27 | physical violence by other means | 11.6 | 43.1 |
| Undetermined intent of Blunt Objects | 27 | self-harm by drowning | 3.7 | 13.5 |
| Undetermined intent of Blunt Objects | 27 | self-harm by jumping | 2.7 | 10.0 |
| Undetermined intent of Crashing | 326 | cyclist road injuries | 19.7 | 6.0 |
| Undetermined intent of Crashing | 326 | motor vehicle road injuries | 55.9 | 17.1 |
| Undetermined intent of Crashing | 326 | motorcyclist road injuries | 42.8 | 13.1 |
| Undetermined intent of Crashing | 326 | pedestrian road injuries | 82.4 | 25.3 |
| Undetermined intent of Crashing | 326 | physical violence by other means | 49.8 | 15.3 |
| Undetermined intent of Crashing | 326 | self-harm by drowning | 24.9 | 7.6 |
| Undetermined intent of Crashing | 326 | self-harm by jumping | 35.8 | 11.0 |
| Undetermined intent of Explosion | 12 | other exposure to mechanical forces | 0.7 | 5.7 |
| Undetermined intent of Explosion | 12 | other unintentional injuries | 3.0 | 24.9 |
| Undetermined intent of Explosion | 12 | physical violence by other means | 3.7 | 31.1 |
| Undetermined intent of Explosion | 12 | self-harm by drowning | 1.5 | 12.7 |
| Undetermined intent of Explosion | 12 | self-harm by jumping | 3.1 | 25.6 |
| Undetermined intent of Hot Objects | 2 | fire, heat, and hot substances | 0.4 | 19.3 |
| Undetermined intent of Hot Objects | 2 | physical violence by other means | 1.0 | 50.0 |
| Undetermined intent of Hot Objects | 2 | self-harm by drowning | 0.4 | 18.4 |
| Undetermined intent of Hot Objects | 2 | self-harm by jumping | 0.2 | 12.3 |
| Undetermined intent of Moving Objects | 204 | pedestrian road injuries | 107.2 | 52.5 |
| Undetermined intent of Moving Objects | 204 | physical violence by other means | 71.0 | 34.8 |
| Undetermined intent of Moving Objects | 204 | self-harm by drowning | 12.3 | 6.0 |
| Undetermined intent of Moving Objects | 204 | self-harm by jumping | 13.6 | 6.7 |
| Undetermined intent of Sharp Objects | 57 | self-harm by drowning | 24.1 | 42.3 |
| Undetermined intent of Sharp Objects | 57 | self-harm by jumping | 31.5 | 55.3 |
| Undetermined intent of fall | 369 | falls | 175.1 | 47.5 |
| Undetermined intent of fall | 369 | other unintentional injuries | 23.6 | 6.4 |
| Undetermined intent of fall | 369 | physical violence by other means | 52.4 | 14.2 |
| Undetermined intent of fall | 369 | self-harm by drowning | 51.4 | 13.9 |
| Undetermined intent of fall | 369 | self-harm by jumping | 66.5 | 18.0 |
| Undetermined intent of fire and flames | 350 | fire, heat, and hot substances | 107.3 | 30.7 |
| Undetermined intent of fire and flames | 350 | other unintentional injuries | 125.2 | 35.8 |
| Undetermined intent of fire and flames | 350 | physical violence by other means | 89.4 | 25.5 |
| Undetermined intent of fire and flames | 350 | self-harm by fire, heat, and hot substances | 28.1 | 8.0 |
| Unspecified Intestine Diseases | 2188 | diverticular disease of intestines | 383.0 | 17.5 |
| Unspecified Intestine Diseases | 2188 | esophageal diseases | 253.3 | 11.6 |
| Unspecified Intestine Diseases | 2188 | paralytic ileus and intestinal obstruction | 933.2 | 42.6 |
| Unspecified Intestine Diseases | 2188 | ulcerative colitis | 251.5 | 11.5 |
| Unspecified Bacterial Diseases | 1515 | clostridium difficile | 441.6 | 29.1 |
| Unspecified Bacterial Diseases | 1515 | other diarrheal diseases | 833.3 | 55.0 |
| Unspecified Bacterial Diseases | 1515 | respiratory tuberculosis | 89.8 | 5.9 |
| Unspecified Blood Diseases | 154 | endo other than secret cause | 33.8 | 22.0 |
| Unspecified Blood Diseases | 154 | interstitial lung disease and pulmonary sarcoidosis | 100.5 | 65.3 |
| Unspecified Brain Diseases | 770 | epilepsy | 62.1 | 8.1 |
| Unspecified Brain Diseases | 770 | ischemic stroke | 576.5 | 74.9 |
| Unspecified Brain Diseases | 770 | other degenerative diseases of nervous system | 75.7 | 9.8 |
| Unspecified Bronchitis and Bronchiectasis | 724 | unspecified bronchitis and bronchiectasis | 675.0 | 93.2 |
| Unspecified CNS Diseases | 213 | alcohol use disorders | 18.1 | 8.5 |
| Unspecified CNS Diseases | 213 | ischemic stroke | 127.1 | 59.7 |
| Unspecified CNS Diseases | 213 | motor neuron disease | 22.7 | 10.6 |
| Unspecified CNS Diseases | 213 | other degenerative diseases of nervous system | 28.2 | 13.2 |
| Unspecified CNS Infection | 112 | encephalitis japanese | 8.3 | 7.4 |
| Unspecified CNS Infection | 112 | h influenzae type b meningitis | 18.2 | 16.3 |
| Unspecified CNS Infection | 112 | other meningitis | 36.7 | 32.7 |
| Unspecified CNS Infection | 112 | pneumococcal meningitis | 29.9 | 26.7 |
| Unspecified CNS Infection | 112 | viral meningitis | 18.9 | 16.9 |
| Unspecified Cardiomyopathy | 6524 | alcoholic cardiomyopathy | 1886.7 | 28.9 |
| Unspecified Cardiomyopathy | 6524 | other cardiomyopathy | 4637.3 | 71.1 |
| Unspecified Chromosomal Diseases | 50 | down syndrome | 12.0 | 24.0 |
| Unspecified Chromosomal Diseases | 50 | other chromosomal abnormalities other | 13.4 | 26.8 |
| Unspecified Chromosomal Diseases | 50 | trisomy 13 and 18 | 21.7 | 43.4 |
| Unspecified Congenital Diseases | 35 | alcohol use disorders | 4.0 | 11.6 |
| Unspecified Congenital Diseases | 35 | congenital others | 6.4 | 18.2 |
| Unspecified Congenital Diseases | 35 | moderately complex congenital heart disease | 2.1 | 6.0 |
| Unspecified Congenital Diseases | 35 | other chromosomal abnormalities other | 3.6 | 10.2 |
| Unspecified Congenital Diseases | 35 | trisomy 13 and 18 | 3.0 | 8.5 |
| Unspecified Digestive Diseases | 282 | diverticular disease of intestines | 15.2 | 5.4 |
| Unspecified Digestive Diseases | 282 | gallbladder and biliary diseases | 35.8 | 12.7 |
| Unspecified Digestive Diseases | 282 | other diarrheal diseases | 29.4 | 10.4 |
| Unspecified Digestive Diseases | 282 | pancreatitis | 16.8 | 6.0 |
| Unspecified Digestive Diseases | 282 | paralytic ileus and intestinal obstruction | 39.5 | 14.0 |
| Unspecified Digestive Diseases | 282 | peptic ulcer disease | 49.4 | 17.5 |
| Unspecified Digestive Diseases | 282 | ulcerative colitis | 17.1 | 6.1 |
| Unspecified Digestive Diseases | 282 | vascular intestinal disorders | 37.0 | 13.1 |
| Unspecified Eating Disorders | 183 | other nutritional deficiencies | 105.0 | 57.4 |
| Unspecified Eating Disorders | 183 | protein-energy malnutrition | 70.0 | 38.3 |
| Unspecified Endocrine Cancer | 27 | other cancer | 16.4 | 60.7 |
| Unspecified Endocrine Cancer | 27 | thyroid cancer | 5.6 | 20.7 |
| Unspecified Endocrine Cancer | 27 | vulva cancer | 3.3 | 12.2 |
| Unspecified Endocrine Diseases | 2 | alcohol use disorders | 1.2 | 58.7 |
| Unspecified Endocrine Diseases | 2 | diabetes mellitus type 1 | 0.1 | 6.5 |
| Unspecified Endocrine Diseases | 2 | diabetes mellitus type 2 | 0.4 | 21.9 |
| Unspecified Endocrine Diseases | 2 | endo other than secret cause | 0.2 | 10.9 |
| Unspecified Female Genital Cancer | 2011 | cervical cancer | 696.7 | 34.6 |
| Unspecified Female Genital Cancer | 2011 | other cancer | 241.1 | 12.0 |
| Unspecified Female Genital Cancer | 2011 | ovarian cancer | 928.6 | 46.2 |
| Unspecified Female Genital Cancer | 2011 | vulva cancer | 121.1 | 6.0 |
| Unspecified GI Cancer | 7733 | colorectal cancer other | 808.0 | 10.4 |
[truncated: 9,891 more chars]
